# Dramatic cropland expansion in Myanmar following political reforms threatens biodiversity

**DOI:** 10.1038/s41598-018-34974-8

**Published:** 2018-11-08

**Authors:** Yuchen Zhang, Graham W. Prescott, Rebecca E. Tay, Borame L. Dickens, Edward L. Webb, Saw Htun, Robert J. Tizard, Madhu Rao, Luis Roman Carrasco

**Affiliations:** 1 Department of Biological Sciences, National University of Singapore, 14 Science Drive 4, Singapore, 117543 Republic of Singapore; 2 Wildlife Conservation Society Myanmar Program, Aye Yeik Mon 1st Street, Ward 3, Building C1 Hlaing Township, 11051 Yangon, Myanmar; 3 Wildlife Conservation Society Asia Program, 2300 Southern Boulevard, Bronx, New York, New York 10460 USA

## Abstract

Effective conservation planning needs to consider the threats of cropland expansion to biodiversity. We used Myanmar as a case study to devise a modeling framework to identify which Key Biodiversity Areas (KBAs) are most vulnerable to cropland expansion in a context of increasingly resolved armed conflict. We studied 13 major crops with the potential to expand into KBAs. We used mixed-effects models and an agricultural versus forest rent framework to model current land use and conversion of forests to cropland for each crop. We found that the current cropland distribution is explained by higher agricultural value, lower transportation costs and lower elevation. We also found that protected areas and socio-political instability are effective in slowing down deforestation with conflicts in Myanmar damaging farmland and displacing farmers elsewhere. Under plausible economic development and socio-political stability scenarios, the models forecast 48.5% of land to be converted. We identified export crops such as maize, and pigeon pea as key deforestation drivers. This cropland expansion would pose a major threat to Myanmar’s freshwater KBAs. We highlight the importance of considering rapid land-use transitions in the tropics to devise robust conservation plans.

## Introduction

Cropland expansion is a major global driver of deforestation^1^. With global crop demand projected to increase from between 25% to 70% (by mass) by 2050^2–4^ and increasing world market integration, demand for new land is likely to be concentrated in tropical developing nations. Countries that are particularly vulnerable have large areas suitable for agriculture covered by forests and are in the early stages of their forest transition^5^. Examples of regions that have recently passed through this stage include Peninsular Malaysia, Indonesian Papua, and Papua New Guinea, where oil palm expansion was responsible for the majority of deforestation between 1990–2010^6^. While deforestation rates remain high in these countries, other highly-forested countries and regions where cropland expansion has been hindered by conflict, like Myanmar, Congo and Colombia, may go through the same process of rapid, agriculture-driven deforestation^7^.

A modelling framework is required to respond to agricultural pressures that are both temporally and spatially heterogeneous. Ignoring such variability could lead to reduced cost-effectiveness, efficiency or even complete failure^8^ in conservation planning. Previous conservation work has tended to assume that conservation threats do not change over time^9^. In particular, the establishment of protected areas (PAs) is commonly identified using static information, and assumes that the system is at equilibrium. Given the rapid pace of tropical land use transition, analyses that assume static threats may not be applicable to this region. Threats with changing intensity over time and space should therefore be assessed by carrying out temporal and spatial predictions, before identifying corresponding conservation actions^10^. This is especially relevant in the context of increasing global crop demand. It is important to foresee where cropland expansion may occur and the implications for this expansion on forests and biodiversity^11,12^.

Another source of uncertainty is the joint presence of weak institutions and armed conflict within some tropical nations. Conflict can potentially increase or decrease the risk of forest loss. The political, economic, and social environment of a country can be major and complex determinants of deforestation via levels of authoritarianism, market openness and internal conflict^6,13^. For example, authoritarian governments have historically engineered large-scale deforestation-for instance the Amazon Settlement and transmigration schemes in Brazil and Indonesia respectively^13,14^-but have also inhibited environmentally destructive investment and economic development in these countries^6,15^. Indeed the experience of both Brazil and Indonesia after the fall of dictatorship was of accelerated deforestation for pasture, soy, and oil palm^6,15^. Conflicts lead to exploitation of forest resources for funding of rebels in some cases such as Colombia^16^, but can also deter large-scale commercial deforestation in others such as in the case of Rwanda where land could not be used for refugee settlements^17^.

Uncertainty surrounding which specific forests and biodiversity would be affected by cropland expansion and political forces may undermine conservation efforts. Understanding and predicting them is thus of key interest to policy makers and conservation planners. Studies about conservation planning that address threats that change over time have recently emerged^9,10,18^, but very few of them consider cropland expansion in the context of conservation planning. We devised a modelling framework that explores cropland threats to forests and biodiversity, assessing changes in cropland expansion through socio-economic factors such as agricultural rent and armed conflict. We applied this modelling framework to Myanmar as a case study. Myanmar is undergoing unprecedented political and economic reforms while harbouring some of the largest expanse of remaining tropical forests in continental SE Asia^19^. Cropland expansion was a key deforestation driver from 1990–2016^20^. A review of emerging forest conservation issues in Myanmar identified rapid expansion of commercial agriculture in the wake of political and economic liberalisation as one of the major threats to Myanmar’s forests by the mid-2020s^21^. So far, 132 Key Biodiversity Areas (KBAs) have been identified by the Wildlife Conservation Society in Myanmar. These KBAs are areas that hold significant populations of species of high conservation concern^22^. Only 37 of these KBAs are legally protected. Myanmar represents a case study of a country undergoing significant political change. This requires models that can capture the dynamic threats to forests and biodiversity to inform future PA network design efforts.

In this study, we modelled cropland expansion in Myanmar based on a von Thünen agricultural versus forest rent framework. The von Thünen framework posits that forest is converted to cropland if the rent from land clearance for cropland is higher than forest rent^23^ (see Methods). We additionally expanded the model to include three other factors that might explain the current cropland and forest distributions in Myanmar: protected area (PA) status, elevation, and social stability to account for armed conflict. We then translated the extended von Thünen framework into generalized linear mixed-effects models by modelling current land use as a function of the factors identified through the von Thünen framework: agricultural value, transportation cost, elevation, socio-political stability, and PA status (see Methods). We standardized all continuous variables in the model by deducting the mean and dividing by the standard deviation. We modelled the distribution of thirteen crops: four export crops (maize, rice, chickpea, and pigeon pea), two food crops for domestic consumption (cassava and potato), three non-oilseed industrial crops (rubber, sugarcane, and tobacco), and four oilseed crops (groundnut, sesame, sunflower, and oil palm)^24,25^ (see Methods). The best models capturing the relationship between the predictors and the observed cropland distribution were then employed to produce cropland expansion projections under several economic development scenarios for all crops. These scenarios were motivated by 10 year outlooks of the OECD-FAO and the International Energy Agency on agriculture and transportation costs^26–28^. These scenarios ranged from a business as usual baseline (“baseline scenario”), increase in existing yields to potential yields (“potential yield scenario”), increase of agricultural value by 50% (“agricultural value scenario”), increase in transport costs by 35% (“transportation cost scenario”), attainment of socio-political stability (a “stability scenario” in which the border states undergoing civil war have the same level of conflict as the core states and regions of Myanmar) and a combination of increase of agricultural value and socio-political stability (“combined scenario”, Table 1). The cropland expansion projections under different scenarios were used to ascertain the KBAs at greater risk from cropland expansion.

**Table 1:** Description of economic development scenarios used to investigate and compare possible cropland expansion in Myanmar under each scenario.

Scenario	Assumption	Crop yield	Justification
Baseline	all the explanatory variables remain at current values	actual yield	
Potential yield	agricultural value is calculated based on crop potential yield	potential yield	Economic reforms and investment in agriculture might lead to increased crop yield
Agricultural value	agricultural value increases by 50% in real terms	potential yield	The FAO’s Food Price Index, which tracked the price of agricultural food commodities, increased by 50% in real terms over the past ten years^26^ and we assume this trend will hold in the next 10 years
Transportation cost	transport costs increase by 35% in real terms	potential yield	Based on the prediction of oil price change by the International Energy Agency in the next 10 years^27,28^
Stability	civil war ends, and socio-political stability is uniform across Myanmar	potential yield	Ceasefire agreements have been signed^54^. Peace may allow the development of agriculture leading to deforestation.
Combined	agricultural value increases by 50% in real terms, and an achievement of socio-political stability	potential yield	

This study has achieved three objectives: (i) developed a modelling framework to understand the factors that explain the current cropland and forest distribution in Myanmar, (ii) projected spatial patterns of cropland expansion in Myanmar for multiple crops under different economic development scenarios; and (iii) identified the KBAs with the highest vulnerability to cropland expansion.

## Results

### Analysis of current land use

**Figure 1:**
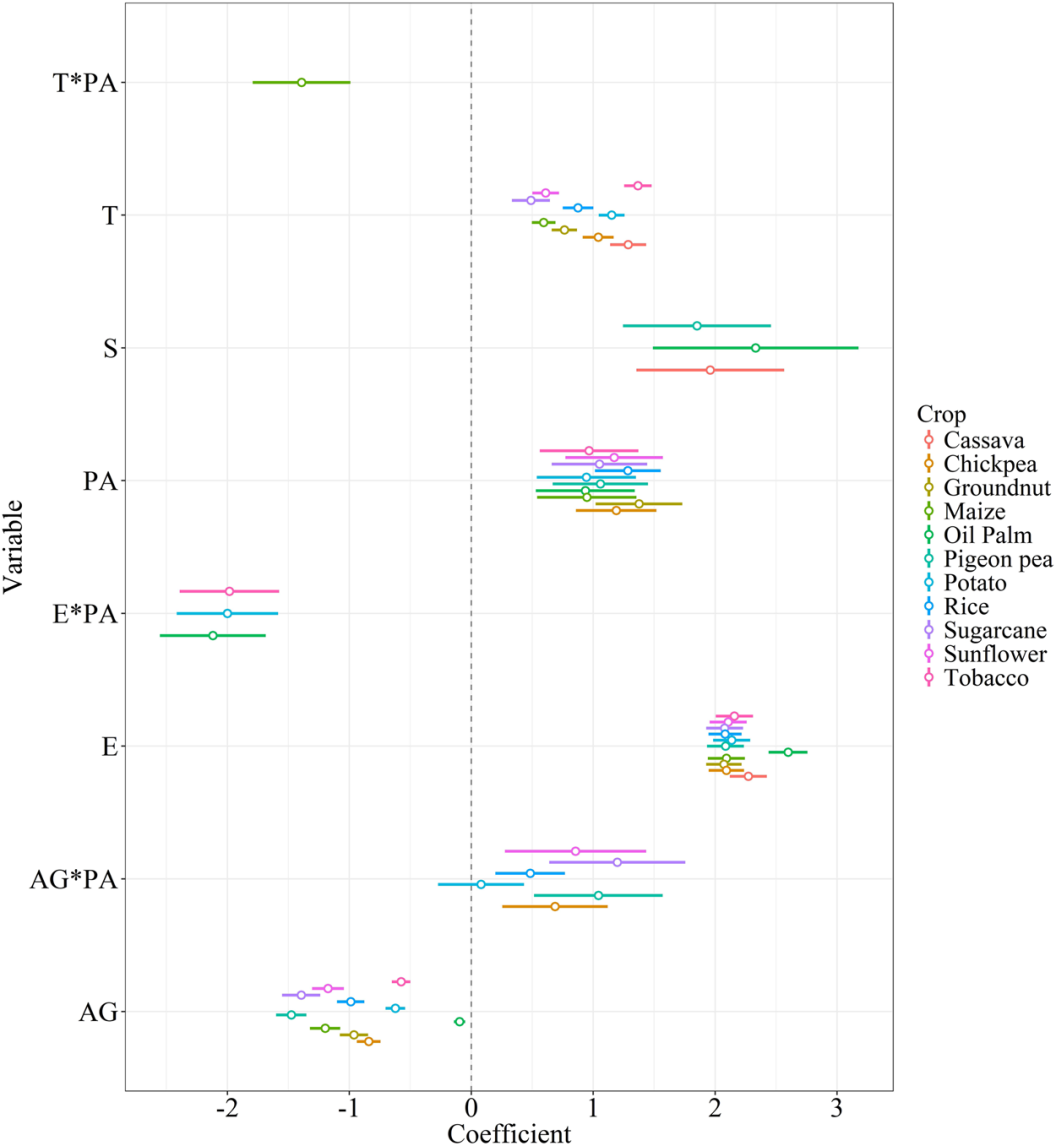
Plot of explanatory variables effect sizes with confidence intervals for the selected best generalized linear mixed-effects models for 11 crops to explain the current land use distribution in Myanmar. The dependent variable was the probability of forest presence. AG = Agricultural value, T = Transportation cost, E = Elevation, S = Socio-political stability, PA = PA status. “*” denotes interaction terms.

We selected the generalized linear mixed-effects model that best explained the current land use distribution of each of the 13 crops considered. However, no results were available for sesame due to a quasi-perfect separation issue and for rubber due to heteroscedasticity problems. We found that the drivers of land use were consistent among all crops considered in the selected best models (Fig. 1 shows the results for the best models for each crop). Elevation had a higher effect size for all crops (model slope coefficient β ranged from 2.072 for groundnut to 2.598 for oil palm) than transportation cost (β ranged from 0.489 for sugarcane to 1.367 for tobacco) and agricultural value (β ranged from −1.476 for pigeon pea to −0.096 for oil palm). As we expected from the von Thünen framework, land is less likely to be used as cropland if located at higher elevations or located in areas that presented higher transportation costs. Agricultural value had negative effect sizes, indicating that land is more likely to be used as cropland at higher agricultural values.

Socio-political stability (β ranged from 1.852 for pigeon pea to 2.333 for oil palm) had a higher effect size than PA status (β ranged from 0.936 for oil palm to 1.376 for groundnut) and both had positive coefficients (Fig. 1). This means that forests were more likely to be converted to cropland if they were located outside regions that have experienced civil war or outside PAs. These results were robust to the use of alternative land use distribution maps (see Supplementary Fig. S6).

### Scenarios for cropland expansion projections

**Figure 2:**
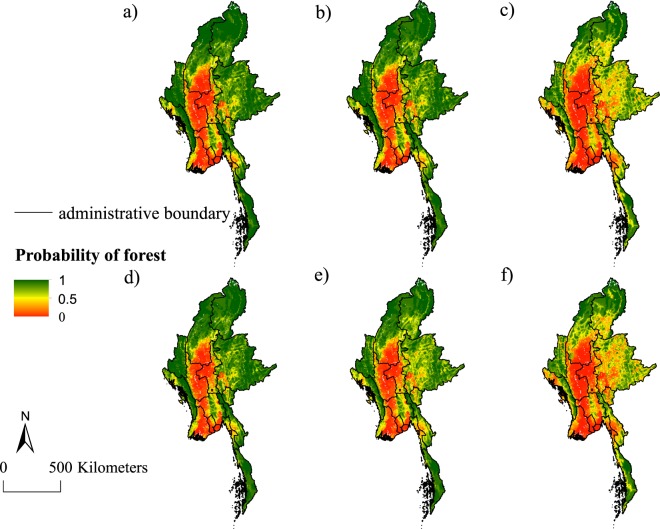
Averaged projected land use distribution across 11 crops (sesame and rubber were excluded). (**a**) Baseline scenario; (**b**) potential yield scenario; (**c**) agricultural value scenario (**d**) transportation cost scenario; (**e**) stability scenario; and (**f**) combined scenario.

Across all scenarios, cropland was projected to remain concentrated near the centre of Myanmar, but to expand to the edges of the country as these areas had high potential yield (Fig. 2). Exceptions were the land use distribution projected for crops for domestic markets (potato and cassava), which did not present very apparent differences in land use spatial patterns (see Supplementary Fig. S4).

Under the agricultural value scenario, in which agricultural value doubles, there was larger scale cropland expansion than under the baseline scenario. Cropland expansion occurred mainly in the eastern part of Myanmar (Fig. 2). This trend was stronger for export crops and oilseed crops compared to the other two categories of crops (see Supplementary Figs S3, S4 and S5). However, there was almost no expansion in the case of oil palm due to the small effect size of agricultural value (see Supplementary Fig. S5).

Under the transportation cost scenario, in which transport costs increases by 35%, cropland contracted around current cropland sites (Fig. 2). We observed a similar trend for all crops for domestic market and oilseed crops, but, compared to the baseline scenario, there were no apparent differences and even a slight expansion from maize due to its high potential yield and small effect size of transportation cost (Fig. 1, see Supplementary Figs S3 and S4).

Under the stability scenario, which approximates an end to civil war, specifically assuming that border regions with civil war become as peaceful as the regions not undergoing civil war, cropland increased mainly in border provinces that are currently undergoing civil conflict (Fig. 2).

The combined scenario, a combination of the stability and agricultural value scenarios in which agricultural value increases and the civil war ends, led to the largest amount of cropland expansion in Myanmar in all border provinces, but more extensively in the eastern part (Fig. 2).

Comparison of cropland expansion from individual crops under the combined scenario showed that export crops, especially maize and pigeon pea, led to the most extensive cropland expansion. This was in contrast to oil palm that showed less extensive expansion, due to low potential yields, even though the Myanmar government is actively promoting its plantation (see Supplementary Figs S3, S4 and S5).

### Assessment of threats to KBAs from cropland expansion

**Figure 3:**
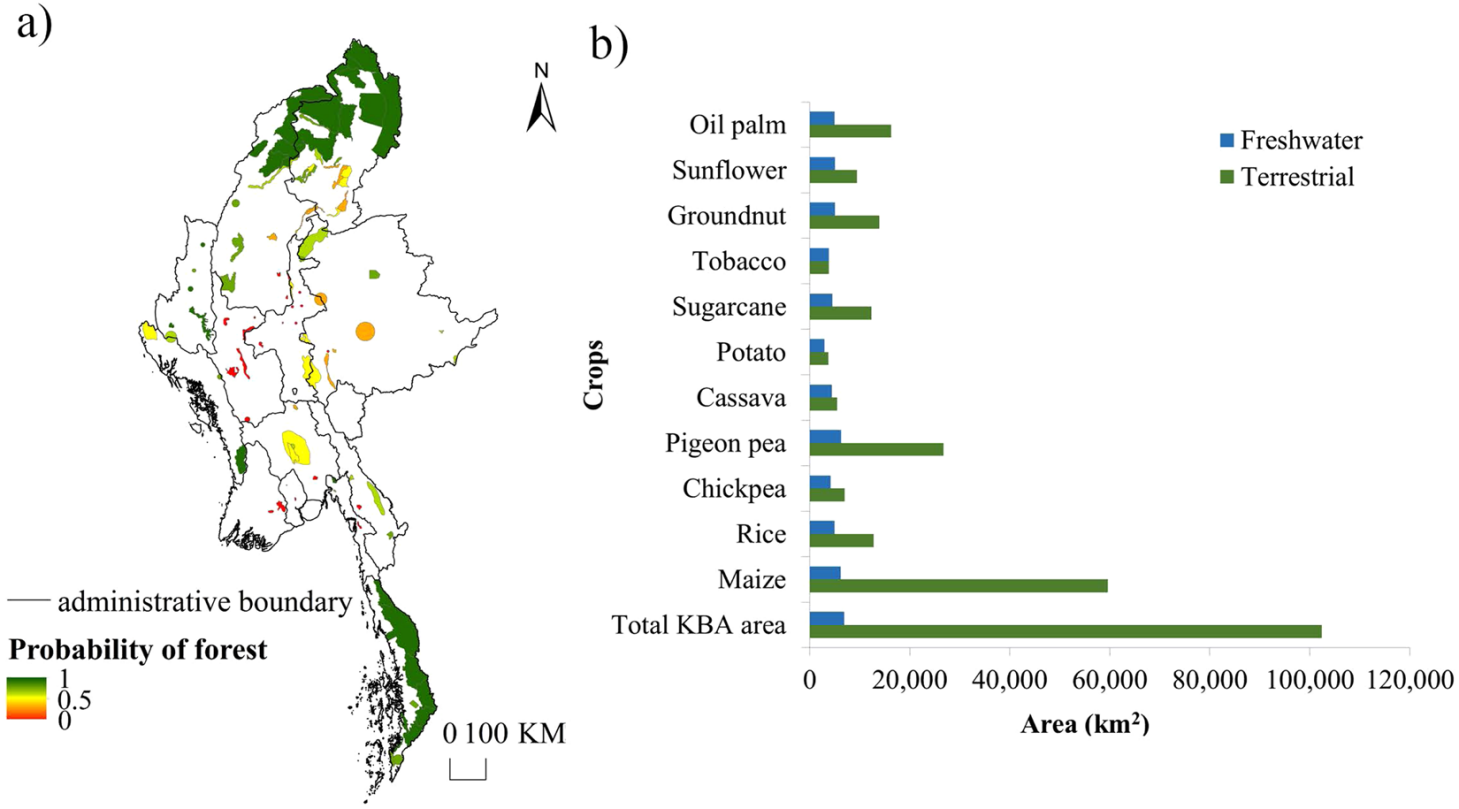
(**a**) Average probability of forest presence within freshwater and terrestrial KBAs projected under the combined scenario across 11 crops, (**b**) Area of freshwater and terrestrial KBAs affected by projected cropland expansion (with less than 50% probability of forest presence) from each crop under the combined scenario (sesame and rubber were excluded).

Results from overlaying our projected cropland expansion maps with KBAs showed that 28 KBAs were under high probability of deforestation by all crops (more than 50% of the area is predicted to be converted to cropland). Conversely, only 8 of these KBAs were within PAs (see Supplementary Table S4). Another 17,784 km^2^ of land would need to be protected to cover all highly vulnerable KBAs. This would result in PA coverage increasing from 10% to 13% of Myanmar’s total land area, which would still not meet Myanmar’s 17% Aichi Biodiversity Target 11. Most of the affected KBAs were in the central regions of Myanmar, with only a few located near the national border with China (Fig. 3). We found freshwater KBAs, including riparian zones, to be at greater vulnerability to deforestation than terrestrial KBAs (see Supplementary Table S4). Only 2 out of 24 freshwater KBAs were within PAs whilst 31 out of 74 terrestrial KBAs were protected.

## Discussion

The current land use distribution in Myanmar is counter-intuitive from an agronomic perspective. Cropland is disproportionately located in areas with relatively low potential yield and thus lower potential agricultural value^29^. In Myanmar, high potential yield land is mostly located in states and regions that have experienced civil conflict, suggesting that it has prevented cropland expansion. Large-scale forced displacement, physical damage to farmland and infrastructure, and insecure land tenure have inhibited agricultural development in these regions^30^. Another factor promoting deforestation following peace is the strategic allocation of agricultural concessions by the military government in contested territory following cease-fire agreements, in order to personally enrich members of the military, deny resource access to insurgents, and make the territory legible to the state^31^. An example of this is the allocation of a cassava agribusiness concession to a military-allied crony businessman in the Hukaung Valley which led to deforestation in a formerly protected area^31,32^. Therefore, we would expect higher levels of deforestation following the attainment of peace and economic development, especially in the border areas. While our data analysis and results only applies to Myanmar, the same kind of scenario modelling could be usefully applied to identify forested and key biodiversity areas at risk from future cropland expansion in countries where conflict has impeded agricultural development such as Democratic Republic of Congo, Colombia and Rwanda^7^. Mechanisms of how conflicts increase or decrease deforestation are hard to generalize and heavily dependent upon local context. Nonetheless, the end of conflict usually creates an inflection point in deforestation dynamics. It is at this junction where conservation interventions are particularly crucial to attain a sustainable forest transitions.

In terms of effects of individual crops on cropland expansion in Myanmar, we found that export crops, especially maize and pigeon pea, had the greatest potential influence on projected forest conversion (see Supplementary Table S3). These are crops for which Myanmar has a comparative production advantage and are extensively exported. We found these export crops are generally sensitive to price changes, such as the observed increase in sown acreage of pulses in Myanmar after price increases in the 1990s^24^. Furthermore, because those export crops are mainly non-perishable crops, their distribution is not as sensitive to the time taken to bring them to markets as opposed to crops for domestic consumption and oilseed crops. In contrast, the models projected low levels of oil palm expansion. This was because most regions in Myanmar are not suitable for oil palm cultivation according to oil palm potential yield maps^29^. This result is in contrast to Myanmar’s government efforts that have tried to curb the volume of oil palm imports to stabilize edible oil prices. However, because Myanmar has no comparative advantage in palm oil production, it will be unlikely to meet the domestic demand by increasing their area of production^24^. Most regions in Myanmar have very low palm oil potential yields^29^ and more benefits could be earned if sustainable forest management were practiced instead.

Projected cropland expansion poses serious threats to KBAs, especially freshwater ones. All freshwater KBAs in Myanmar, except for the Tanai River KBA, the Uyu River KBA, and the Upper Chindwin KBA, were projected to be at higher risk of deforestation (see Supplementary Table S4). Freshwater KBAs are associated with riparian zones and fertile drainages, which makes them suitable for conversion to cropland. In addition, freshwater KBAs are smaller, and only two of them are within PAs in Myanmar. Our results indicate that KBAs are at risk without further protection. Policy makers could direct conservation investment to KBAs that present the highest risk of conversion. This approach would work provided PAs can be expected to withstand the high pressures for cropland expansion. Considering that PAs under high pressure may eventually suffer PADDD^33^, an alternative strategy could be to focus on KBAs with relatively low risk with a lower agricultural rent to forgo. Policy makers could also target those medium risk KBAs considering the trade-off between conservation cost and threat levels. Myanmar has been criticized for its *ad hoc* approach to establishing PAs^34^. Conversely, we found that land within PAs in Myanmar is significantly protected from cropland expansion. This agrees with previous case studies reporting that some PAs in Myanmar (Chattin Wildlife Sanctuary and Popa Mountain Park) have successfully conserved forests^35,36^.

There are many other countries similar to Myanmar that are facing or might face political and economic transitions in the future. For instance, Colombia and Central African Republic. One possible strategy to reconcile cropland expansion and biodiversity conservation in these countries is land sparing. Land sparing can be achieved through cropland intensification, which will be more feasible if Myanmar becomes more peaceful and foreign investment increases. However, cropland intensification alone may not spare land for nature possibly because of the rebound effects by which higher agricultural rent may incentivize farmers to convert more land^37^. Therefore, land intensification in these countries should be combined with other conservation actions. For instance, payments or technical assistance conditioned to habitat conservation^38^. Land use zoning and spatially strategic deployment of technology and infrastructure are other alternatives to avoid rebound effects from cropland intensification^38^. A combination of these two strategies may be necessary. For example, KBAs could be zoned for conservation while intensification of cropland is planned far from KBAs by directing yield-increasing measures only to those areas zoned as cropland. Land sharing through agroforestry could also be used as an alternative strategy. This would especially relevant in countries with a long history of agroforestry such as Myanmar and Indonesia^39^.

Our analyses are based on a few assumptions about markets, crop yields, and the political environment. We assume that individual farmers behave rationally with perfect information and always act to maximize their profits. We additionally assume that maximum potential yields are attainable. However, these assumptions may be violated in the context of Myanmar. Individual economic opportunity, including agricultural development, has been severely constrained in Myanmar by the legacy of military dictatorship, a centrally planned economy, international sanctions, and insecurity of land tenure^40^. Moreover, weak governance may prioritize the economic advancement of the political and military elites at the expense of individual farmers. This would result in the formulation of policies that may not be rational from an agronomic or economic perspective. Realization of potential yields in Myanmar is currently constrained by a lack of infrastructure for transport and irrigation, access to best crop varieties, and technology^40^. Because of these assumptions, our findings are illustrative of the directions in which cropland expansion could proceed^41^. However, it is worth noting that previous research generating global cropland expansion projections under the Shared Socioeconomic Pathways has also found that the areas near the central region have higher cropland expansion potential than the Northern border areas of Myanmar^42^, thus agreeing with our results. Although in our scenarios we used changes that could realistically occur in the future based on 10 year outlooks from OECD-FAO and the International Energy Agency^26–28^, there may be lags between the viability to convert area to cropland and the actual conversion. In addition, it is uncertain when conflicts will end completely and socio-stability will be achieved. Therefore, we cannot predict exactly when conversion will occur. The assumptions of our analyses will be more applicable to Myanmar in potential medium to long term. This would correspond to the end-point of a transition to a free-market democracy that is fully integrated into the global economy. Therefore, our analyses should not be regarded as accurate predictions of land-use transitions during the current period that presents high uncertainty but rather an exploration of a wide range of possible dynamics.

Although some of the input layers in our analysis like KBAs and PA maps were developed specifically for Myanmar, due to data paucity, we had to resort to coarser global cropland and yield maps which are, to our knowledge, the best sources of data available. In the absence of accurate and spatially explicit data on conflict intensity, we used a binary dummy variable for states with non-Bamar ethnic majorities as a proxy for armed conflict. This matches the distribution of armed conflict in Myanmar, which has largely seen armed groups representing Myanmar’s ethnic minorities fighting for greater political autonomy. However, this dummy variable also correlates with other factors such as governance culture and economic development levels. In our ‘stability’ scenario, we assume that under an end to civil war the states which have experienced armed conflicts will more closely resemble the Bamar-majority regions that have largely avoided conflict regionally, due to the well-documented effects of peace on development and deforestation. However, underlying differences in culture and governance may persist in the event of peace (e.g. if ethnic minority governments are given more autonomy over natural resources as part of a peace agreement) which may affect the pattern of future cropland distribution and is not explicitly captured by our model.

In addition, although we modelled the likely location and extent of future cropland expansion under realistic scenarios, panel data regression would have allowed to better relating land-use change with time, helping understand the actual pace of change. However, multiple observations of land use distributions across time were lacking. As such, our modelling assumes that the inferred relationship between the predictors and the observed snapshot spatial distribution of land use can be used to understand how land-use will change when those predictors change in the future using the 10 year outlooks from OECD-FAO and the International Energy Agency^26–28^. Future research would thus benefit from increasing mapping efforts in Myanmar over multiple periods.

Modelling threats to biodiversity that change over time and space is necessary to better schedule conservation decisions in the future. It enables conservation resources to be allocated optimally and timely between regions. Our modelling framework for Myanmar could serve as a useful tool for countries transitioning between states of armed conflict and market liberalization. Application of our framework has demonstrated that dramatic cropland expansion is expected in one of the countries with the largest remaining forest tracts in SE Asia. This threat is especially acute in freshwater KBAs. PA design would need to consider these rapidly changing landscapes to facilitate a sustainable forest transition in Myanmar.

## Methods

### Study area

With 44.2% of forest cover, Myanmar presents one of the largest unexploited tracts of land highly suitable for agriculture^43^. Located within one of the most globally threatened hotspots (the Indo-Burma hotspot), Myanmar’s remaining extensive forests and biodiversity makes it a high conservation priority^44^.

Cropland in Myanmar is currently concentrated in the drier central regions^45^. Long-standing civil conflict in some of the peripheral provinces of Myanmar such as Shan and Kachin has led to abandonment of cropland and internal migration^30^. Under military dictatorship (1962–2011), Myanmar was politically and economically isolated and under-developed. Since the 2010 general election, the agricultural sector is likely to benefit from increased access to international markets and private investments. This is expected to improve technology and infrastructure leading to cropland expansion at the expense of forests and biodiversity^41^.

Only 10% of the land in Myanmar is currently within PAs, and they tend to be small in size, vulnerable to encroachment by cropland, and do not adequately cover all bioregions within the country^46^.

### The von Thünen framework

Within spatial economic theory, the von Thünen framework assumes that increasing distance to the market and concomitant transport costs counter the rents generated by agriculture, making less likely the allocation of cropland far from the markets^47^. The von Thünen framework is able to produce adequate projections of cropland expansion. It is very flexible, making it suitable to be applied to dynamic conservation problems^37,48^. The von Thünen framework thus allows for a unifying theoretical framework to model different policy interventions.

When farming and forestry are considered as potential land uses, the von Thünen framework posits that forest is converted to cropland if the rent differential between land clearance for agriculture and forest rent (*DIFF*) is positive^23^. To account for armed conflict within the traditional von Thünen model, we adopted an approach in which other salient processes that may play a role on cropland expansion in Myanmar were included. These are chiefly social stability (*S*), protected areas (*PA*) and elevation (*E*). We hypothesized that these processes would affect production costs. Key assumptions included an increase in investment failure and costliness with higher social instability. We also considered financial penalties when PA encroachment occurred and the effect of elevation influencing transportation costs. The extended von Thünen framework therefore becomes:

1 $${\rm{DIFF}}={\rm{AG}}-{T}-{\rm{TRS}}-{f}({S},{\rm{PA}},{E})$$

where *AG*: agricultural value ($/hectare), which is revenue minus production costs that do not include transport costs; *T*: transportation cost ($/hectare); *TRS***:** timber rent under sustainable forest management ($/hectare). *f()* is an unknown function that relates *S*, *PA* and *E* with production costs and needs to be estimated. We then translated the extended von Thünen model into a linear mixed-effects model (hierarchical model) to infer the influence of the different elements of the framework into the observed distribution of cropland (see “Statistical models” section).

### Data collection

We compiled spatial information on land use in Myanmar and factors that could influence land use with Geographic Information Systems. We performed an evenly spaced point sampling at a 0.05 degree resolution (the area of a similar grid cell along the equator is around 3,025 hectares) in ArcMap (version 10.2). This generated 23,313 points covering the entirety of Myanmar. All data for the variables entering the analyses were extracted in ArcMap and exported to R (version 3.4.1)^49^ for analysis (Supplementary Table S2 contains the variables considered, their source and spatial resolution. Dataset 1 and Dataset 2 contain the data used in the analysis for regression and projection respectively and Supplementary code contains the R code used). We standardized all the continuous variables considered by deducting each variable’s mean to each observation and dividing this by the standard deviation of that variable to allow direct comparison among model effect sizes.

#### Crops considered

We chose crops based on the highest harvested area, yield, price, or those actively promoted by government policies in Myanmar^24^. We selected a total of 13 crops under four categories: export crops (maize, rice, chickpea, and pigeon pea), food crops for domestic consumption (cassava and potato), non-oilseed industrial crops (rubber, sugarcane, and tobacco), and oilseed crops (groundnut, sesame, sunflower, and oil palm)^24,25^.

#### Variables considered

Our dependent variable was the current land use distribution in Myanmar, coded as a binary variable with forestland coded as ones and cropland as zeros. We classified the other land use types as miscellaneous and did not include them in the analysis (Supplementary Table S1 shows the reclassification of each land category). Several cropland distribution maps were available and here we present the analysis based on cropland distribution for the GLC2009. This dataset was chosen because it captures the land use distribution before the economic reforms and ceasefire agreements that happened in 2011 in Myanmar^45^. To test the robustness of our analyses to cropland map used, we repeated the statistical analyses with an alternative cropland dataset from the International Institute for Applied Systems Analysis-International Food Policy Research Institute cropland percentage map^50^ (see Supplementary methods). Our independent variables were those motivated by the extended von Thünen framework: agricultural value, transportation cost, elevation, socio-political stability and PAs. We coded socio-political stability as *division* and *state* referring to non-civil conflict and non-conflict regions respectively. These refer to regions with Bamar and non-Bamar ethnic majorities respectively. This is because non-Bamar ethnic majority regions present long-standing struggles for political autonomy among minority groups. PAs were coded either as non–PA with zeros vs. PA with ones. The other variables were continuous variables (see Supplementary methods).

### Statistical models

To model the factors that best project the current distribution of forested versus cropland, we fitted generalized linear mixed-effects models using the lme4 package in R^51^. These models resulted from translating the extended von Thünen framework into linear models. We modelled our dependent variable, land use (*L*), as a function of the following fixed effects: agricultural value (*AG*), transportation cost (*T*), elevation (*E*), socio-political stability (*S*), and PA status (*PA*):

2 $$L=\alpha +{\beta }_{1}{\rm{AG}}+{\beta }_{2}T+{\beta }_{3}S+{\beta }_{4}{\rm{PA}}+{\beta }_{5}E+{c}_{m}+\varepsilon \,{\rm{where}}\,{c}_{m}\, \sim \,N(0,{\sigma }_{1}^{2});\,{\varepsilon }_{i}\, \sim \,N(0,{\sigma }^{2})$$

where *α* is the intercept; *β*_1_ to *β*_5_ are the coefficients for the explanatory variables; *c*_*m*_ is the random intercept for administrative unit *m* that is assumed to follow a normal distribution with mean zero and variance *σ*_1_^2^ and *ε* is the error term. Fixed effects are defined here as constant slopes, and random effects are defined as varying intercepts that represent group-level variables that are assumed to follow a normal distribution. Because of lack of spatially explicit data on timber revenue, rents from sustainable forest management (*TRS*) were assumed constant across space and not included in the statistical model.

We calculated the agricultural value from each crop *i* in each point *j* earned from actual yields as:

3 $${{\rm{AG}}}_{{\rm{ij}}}={\rm{TR}}+{Y}_{{\rm{ij}}}\times ({P}_{{\rm{i}}}-{C}_{{\rm{i}}})$$

where *TR*: timber rent from land clearance ($/hectare). *Y*: yield (tonnes/hectare); *P*: farm gate price ($/tonne); *C*: production cost ($/tonne) (see Supplementary methods).

We calculated the transportation costs as a function of accessibility based on the actual road infrastructure network as:

4 $${T}_{{\rm{ij}}}={Y}_{{\rm{ij}}}\times ({a}_{{\rm{j}}}\times {f}_{{\rm{i}}})$$

where *a*: accessibility (mins to travel to the nearest city); *f*: freight rate ($/tonne∙minute) (see Supplementary methods).

We did this for each of the 13 crops selected. While the dependent variable was consistently the land use distribution in Myanmar, values of agricultural value and transportation cost changed when the model was used for different crops.

As the response variable in each case was binary, we employed a log of odds (logit) transformation. As a result, following back-transformation, the dependent variable became the probability of a point having forest presence. We included random effects to account for potential spatial auto-correlation, as well as factors related to governance, socio-political and cultural dynamics corresponding to different administrative units. We optimized the random effect structure with three different levels of administrative units as random intercepts and average distances to the rest of observations as random slope. Models were compared using the Akaike information criterion (AIC), before performing model selection on fixed effects^52^.

We used an information theoretic approach for model selection. For each crop we proposed a set of models with different combinations of fixed effects and interactions terms (totally 99 models per crop and only models that could converge were considered). We then selected models with the smallest AIC^53^ (Supplementary Table S3 summarizes all the models considered). We removed multi-collinearity by looking at the variance inflation factors for the fixed effects and assessed heteroscedasticity according to binned plots.

### Scenarios

We used the selected best empirical models for each crop (the model presenting the lowest AIC) to project crop expansion under several scenarios. Under each scenario, we averaged the projected maps across all crops to get the final land use projection maps, which is a model ensemble. These scenarios assume potential yields are realized. Scenario projections for sesame and rubber were excluded due to the lack of potential yield data. The scenarios and their rationale are described in Table 1.

### Assessment of cropland expansion threats to KBAs

To assess the threat to KBAs, we overlaid the probability map of forest presence of the model ensemble in the combined scenario with the spatial locations of KBAs in Myanmar. This scenario was expected to be the worst-case scenario for forests. We calculated the average probability of all locations within each KBA having forest presence and used a cutoff of 50% to identify KBAs with high risk of cropland expansion.

## Electronic supplementary material

Appendix S1

Dataset 1

Dataset 2

## Data Availability

All data generated or analysed during this study are included in this published article (and its Supplementary Information files).
