# Supplementary material for: Dramatic cropland expansion in Myanmar following political reforms threatens biodiversity
Source: Sci Rep. 2018 Nov 8;8:16558. doi: 10.1038/s41598-018-34974-8 (PMC6224574; doi:10.1038/s41598-018-34974-8)
Supplement: Supplementary file 1 — Appendix S1 [file 41598_2018_34974_MOESM1_ESM.docx]

**Title:** Dramatic cropland expansion in Myanmar following political reforms threatens biodiversity

**Author list:** Zhang, Y., Prescott, G. W., Tay, R. E., Dickens, B. L., Webb, E. L., Htun, S., Tizard, R. J., Rao, M., Carrasco, L.R.

**Supplementary Methods**

**Data collection**

All economic values were converted to international dollars of 2014 using purchasing power parity exchange rates and deflator tables ^1,2^. One international dollar has the same purchasing power in the domestic economy of a country as one US dollar has in the economy of US.

*Land use types*

Land use in Myanmar was based on the GLC2009 datasets ^3^ and we classified it into cropland, forest, and miscellaneous land (Table S1 and Fig. S1). We also validated this classification against the existing forest cover map, Global Mobile Services (GMS) 2009 ^4^. Out of the 23,313 points, 315 points were categorized as miscellaneous land and were not included in the analysis, which resulted in a binary land use variable.

*Agricultural value (AG)*

We calculated the agricultural value from each crop i in each cell j earned from actual yields as:

$\text{AG}_{\text{ij}}=\text{TR+}Y_{\text{ij}}\times\left(P_{\text{i}}-C_{\text{i}}\right)$ (S1)

where TR: timber rent from land clearance ($/hectare). *Y*: yield (tonnes/hectare); *P*: farm gate price ($/tonne); *C*: production cost ($/tonne); The actual yield of crops was obtained from a global dataset ^5,6^. When developing model projections potential yields were used instead. Potential yield for agricultural value calculation of all crops, except for oil palm, sesame, and rubber, was obtained from the Global Agro-Ecological Zone (GAEZ) data portal ^7^. It was the 30-year average of agro-climatically attainable yield for rain-fed crop from 1961-1990. We calculated potential yield of oil palm based on the potential yield of palm oil obtained from GAEZ ^7^ and an oil extraction rate of 20.65% ^8^. Potential yield of sesame and rubber were not available.

Farm gate prices were from Food and Agriculture Organization of the United Nations (FAO)’ producer price indices (PPI) for the year 2012, which was the most recent available year ^9^. Farm gate price *P* of rubber and tobacco were from FAO’s PPI for the year 2007 and sugarcane from PPI for the year 2003 ^9^. We assumed the price of oil palm fresh fruit bunches to be the same as that in Indonesia in 2008 ^10^.

We estimated the timber rent from land clearance (TR) as follows:

$\text{TR}=\text{AT}\times\left(\text{PT}-\text{CT}\right)-\text{TT}$ (S2)

where AT: amount of timber harvested from land clearance (m^3^/ha); PT: price of timber ($/m^3^); CT: cost of timber harvesting ($/m^3^). TT: timber transportation cost ($/ha). We estimated the amount of timber harvested from land clearance (AT) based on the Global Forest Resources Assessment in 2010 ^11^, which was the growing stock of commercial species. Price of timber products was a five-year average of that from 2000-2014; data were from FAOSTAT as was the export unit price of tropical industrial round wood (non-coniferous species). Cost of harvesting timber (CT) was from research done by the Statutory Research Tasks Unit for Nature & the Environment ^12^. Timber transportation cost (TT) was calculated with the same method of calculating crop transportation cost.

The production cost of each crop usually included the costs of seed, fertilizer, manure, pesticide, labour and land tax. For those crops with benefit to cost ratios (BCR) available (rice, sunflower, groundnut, maize, pigeon pea, maize, sunflower, and potato), we used the BCRs to get their production cost and capital cost was included. For the remaining crops with no BCR data available, we had to calculate their production cost by summing up individual cost of each source. Unlike in the original von Thünen model, we were unable to include capital costs due to data paucity. For rubber and oil palm, because they could not be harvested in the same year as when they were planted and their plantation cycle is longer, we had to calculate their equivalent annual agricultural value directly.

BCR represents the ratio of gross rent to production cost to produce crops. For instance the BCR of rice is 1.94 ^13^, which means that for every 1.94 dollars of gross revenues from producing rice, 1 dollar goes to its production cost. For rice, sesame, groundnut, pigeon pea, maize, sunflower, and potato, the BCRs were 1.94, 2.11, 1.63, 1.82, 1.97, 1.44, and 1.29. These were used to calculate the corresponding production cost for each crop with the formula ^13^:

$C_{\text{ij}}=\frac{Y_{\text{ij}}\times P_{\text{i}}}{\text{BCR}_{\text{i}}}$ (S3)

BCRs of these crops were obtained through literature review ^13-15^. Some were directly available (rice and sunflower), for those that were not and only total revenue and production costs were available in the literature, we calculated the BCR using the formula:

$\text{BCR}=\frac{total revenue}{total production \cos t}$ (S4)

We made several assumptions to get the BCR of crops: 1) BCR of sesame was an average of that in the three seasons in Myanmar: cool season, pre-monsoon season and monsoon season. 2) For groundnut, we calculated its BCR in the cool season as a weighted average of that in two different land types in Myanmar: yar mye and kaing mye, where the weights were the extension of each of the land type areas. Then we used the average of groundnut BCR in the cool season and that in the monsoon season as the final BCR of groundnut ^13^. 3) BCR of sunflower was that of open pollinated sunflower because the majority of the sunflowers planted in Myanmar belong to this type. 4) BCR of potato was an average of the three seasons in Myanmar mentioned above and BCR of pigeon pea was an average of three groups of sampled farmers ^14^. 5) For the BCR of maize, we estimated it as a weighted average of that in low capital households and in high capital households ^15^. The weights used were the percentage of low and high capital households in Myanmar ^16^. Low capital households were assumed as the households in the poorest and poor categories and high capital households were assumed to be those in the upper class category ^16^.

For the other crops without clearly documented BCR in Myanmar, we calculated their cultivation costs based on the level of inputs used in neighbouring Southeast Asian countries and using unit costs from Myanmar whenever possible. We employed the formula below:

$C=mp$ (S5)

where *C*: cultivation cost in Myanmar ($/tonne/hectare); *m*: amount of materials and labour input in neighbouring countries ($/tonne∙hectare); *p*: price of material and labour input in Myanmar ($/unit).

Due to lack of data, we assumed that: 1) Farmers paid the same price for seeds as that by which they sell their crops; 2) The main fertilizer used in Myanmar were urea, triple superphosphate (TSP), and muriate of potash (MOP) ^14^. The ratio of these three kinds of fertilizers was estimated based on the literature; 3) We estimated costs of material and labour input from a FAO survey in Myanmar ^13^. Price of urea and TSP was averaged to be 480 kytes/kg and 173 kytes/kg respectively. MOP price was taken from World Bank Datasets and the import price in 2014 was used because MOP in Myanmar was 100% imported ^14^; 4) The price of manure used in the calculations was 7 kyats/kg; 5) We assumed animal labour to be paired oxen and the payment was 2500 kyats/day and human labour wage was 600 kyats/day; 6) Land tax in Myanmar was assumed to be 7.4 kyats per hectare and pesticides cost for all crops was assumed to be 2000 kyats per hectare. Then we obtained the average yield of a particular crop in Myanmar from FAOSTAT yield for the year 2013. By dividing land tax and pesticide cost per hectare, land tax and pesticide cost per tonne per hectare was obtained.

The detailed calculations of production cost for crops with no BCR ratio available were:

*Chickpea:* we estimated cultivation cost of chickpea from that in India, including cost of seeds, fertilizer, human labour, land tax, and pesticides. Amount of materials and labour inputs was based on a survey done by Reddy et al. ^17^ in India. Ratio of urea, TSP, and MOP was 1: 3:1 ^18^

*Sugarcane:* we estimated cultivation cost of sugarcane according to data on India sugarcane cultivation cost in year 2012-2013 ^19^. It included costs of seeds, fertilizer, manure, human labour, animal labour, land tax, and pesticides. Ratio of the three kinds of fertilizers used in the calculation was 4.5:1.5:1 ^18^.

*Cassava:* we used cultivation cost of cassava in India to estimate that in Myanmar based on a survey ^20^ and by the Central Tuber Crops Research Institute ^21^. Because cassava cultivation usually uses stem cutting rather than seeds, only costs of fertilizer, manure, human labour, animal labour, land tax, and pesticides were included. In this case, we assumed animal labour working time to be same as human labour working time for land preparation. Ratio of the three kinds of fertilizers was 45:40:65 kg/hectare ^21^.

*Tobacco:* we estimated cultivation cost of tobacco according to an existing survey ^22^ in Pakistan. It included cost of seed, fertilizer, manure, human labour, animal labour, land tax, and pesticides. Ratio of urea, TSP, and MOP was assumed to be 5:15:15 ^23^.

*Rubber and oil palm:* For both oil palm and rubber, they have distinct plantation cycles and can only be harvested after the first several years. Thus, for these two crops, we calculated net present values of the total cultivation costs and revenue earned over the entire plantation cycle with a 12% discount rate ^24,25^, and then used the estimated net benefits to get their equivalent annual agricultural value per tonne per hectare.

We assumed the plantation cycle of rubber to be 25 years and it could be harvested only from the 6^th^ year. Thus, we took rubber production cost into account only from the 6^th^ year and revenue also began to be generated from that time. In addition, we included nursey cost of seedlings only for the first year. Additional revenue was earned in the beginning of first year by selling timber products from forest clearance for rubber plantation. We assumed costs of removing grass, fertilizer, and labour to be constant over the entire plantation cycle. Cultivation cost of rubber was estimated based on a rubber plantation project proposal by GBP International in Myanmar ^26^.

We assumed the plantation cycle of oil palm also to be 25 years and it could be harvested from the 3^rd^ year. Thus, revenue from harvesting oil palm began to be generated from this time. We estimated cultivation cost of oil palm from work done by Corley and Tinker ^27^. Cost of seeds, nursery, land preparation (based on human and animal labour cost), and land establishment (based on human and animal labour cost) only occurred in the first year, and timber revenue from forest clearing to plant oil palm was earned only in the beginning of the first year. We assumed operational cost (based on human and animal labour cost) to be different before and after the 3^rd^ year. Fertilizer cost, land tax, and pesticides cost were assumed to be constant over the plantation cycle. We estimated ratio of urea, TSP, and MOP to be 96.6:11.66:142 kg/ha ^27^.

*Transportation cost (T)*

We calculated the transportation costs as a function of accessibility based on the actual road infrastructure network as follows:

$T_{\text{ij}}=Y_{\text{ij}}\times\left(a_{\text{j}}\times f_{\text{i}}\right)$ (S6)

where *a*: accessibility (mins); *f*: freight rate ($/tonne∙minute). Lower transportation cost means there is better market access, making the replacement of forests with agriculture more profitable ^28^. Thus, we assumed cropland expansion in Myanmar would be influenced by transportation cost. Accessibility was the estimated travel time to the nearest large city with at least 50,000 people ^29^. Freight rate was the road freight and was the sum of drivers’ wages and diesel fuel cost.

We made several assumptions to calculate freight rate: 1) driver’s monthly wages were estimated to be $100 in 2010 USD, which was the average wage for blue collar unskilled workers in Myanmar ^30^; 2) trucks used were assumed to be Howo 6 x 4 dump truck, with carrying capacity of 19.32 m^3^, curb weight was 12 tons, and fuel consumption when it was empty was 0.28l/km ^31^; 3) the density of the crops were from online databases ^32,33^; 4) the average speed of the truck was assumed to be 45km/hr ^34^; 5) fuel consumption of the truck when not empty was calculated according to the fuel consumption model proposed by Posada-Henao ^35^ for C3 type truck with curb weight below 28 tons and speed below 45km/hr. Average road slope was conservatively assumed to be 7%, which was the maximum slope that the model could take; 6) diesel costs were estimated to be $3.627 in 2014 ^36^; 7) vehicles make a trip to agricultural zones with no cargo and return to cities fully loaded.

*Elevation (E)*

We obtained elevation data from NASA’s Global Digital Elevation Model and measured as height above sea level in meters ^37^.

*Socio-political stability (S)*

We obtained socio-political stability data from the database of Global Administrative Areas ^38^. We coded it as a binary variable that took the values *state* and *division*.

*Protected area (PA)*

We obtained the distribution of PAs from the Wildlife Conservation Society ^39^ and we also coded it as a binary variable (non-PA coded as zeros vs. PA coded as ones).

**Uncertainty analysis on land use distribution**

*Modelling of land use distribution with IIASA-IFPRI dataset*

In order to verify how different factors explained current land use distribution in Myanmar, for the statistical models in the empirical approach, we did model uncertainty analysis by using an alternative land use dataset reclassified from cropland percentage map. In this alternative dataset, land use in Myanmar was from the International Institute for Applied Systems Analysis-International Food Policy Research Institute (IIASA-IFPRI) cropland percentage map developed in 2015, which gives the percentage of cropland at each location ^40^. For a particular cell, if its cropland percentage was above a certain threshold, we classified its land use type as cropland, otherwise forest. We tested thresholds of 1%, 10%, 30%, and 50% and compared their corresponding land use distribution maps with the existing forest cover map GMS2009 ^4,41^. The threshold that resulted in the best match was chosen, which was a threshold of 30%. This also generated a binary dataset: land with crop percentage above 30% was classified as cropland and coded as zeros, otherwise was classified as forest and coded as ones. Only the binary dependent variable land use was changed in the alternative dataset while all the other variables and model fitting methods remained unchanged.

**Supplementary Results**

*Modelling of land use distribution with IIASA-IFPRI dataset*

Compared to land use distribution modelling under land use from GLC2009 map, modelling under land use from IIASA-IFPRI map showed similar effects on all the explanatory variables (Fig. 1 and Fig. S6). Among the three continuous variables, elevation had the strongest effects on probability of forest presence. Between the two categorical variables, socio-political stability had a stronger influence on probability of forest presence compared to PA status. Agricultural rents had a negative coefficient, so a higher agricultural rent led to the lower possibility of forest existence. All the other four variables were positively correlated with probability of forest presence, showing that land remaining to be forests was associated with higher transportation costs, being located at higher elevations within areas of civil conflict or PA. The only exceptions were cassava and potato, while the former one had a too negative effect size for interaction between transportation cost and PA status, the latter one had socio-political stability negatively correlated with probability of forest presence.**Literature Cited**

1 Economy Watch. *GDP deflator data for all countries. Stanley St Labs*, <<http://www.economywatch.com/economic-statistics/economic-indicators/GDP_Deflator/>> (2015).

2 Economy Watch. *Implied PPP conversion rate data for all countries. Stanley St Labs*, <<http://www.economywatch.com/economic-statistics/economic-indicators/Implied_PPP_Conversion_Rate/>> (2015).

3 Arino, O. *et al.* (European Space Agency (ESA) & Université catholique de Louvain (UCL), 2012).

4 GMS Core Environment Program. in *Greater Mekong subregion altas of the environment* (Asian development bank Manila, Philippines, 2009).

5 Monfreda, C., Ramankutty, N. & Foley, J. A. Farming the planet: 2. Geographic distribution of crop areas, yields, physiological types, and net primary production in the year 2000. *Global Biogeochemical Cycles* **22**, doi:10.1029/2007GB002947 (2008).

6 Ramankutty, N., Evan, A. T., Monfreda, C. & Foley, J. A. Farming the planet: 1. Geographic distribution of global agricultural lands in the year 2000. *Global Biogeochemical Cycles* **22**, doi:10.1029/2007GB002952 (2008 ).

7 FAO. *Global Agro-ecological Zones (GAZE v3.0). IIASA, Laxenburg, Austria and FOA, Rome, Italy*, <<http://www.fao.org/nr/gaez/newsevents/detail/en/c/141573/>> (2012).

8 Butler, R. A., Koh, L. P. & Ghazoul, J. REDD in the red: palm oil could undermine carbon payment schemes. *Conservation Letters* **2**, 67-73 (2008).

9 FAO. *FAOSTAT database. FAO*, <<http://faostat3.fao.org/home/E>> (2015).

10 Feintrenie, L., Chong, W. K. & Levang, P. Why do farmers prefer oil palm? Lessons learnt from Bungo District, Indonesia. *Small-scale Forestry* **9**, 379-396 (2010).

11 FAO. *Global forest resources assessment 2015*. (Food and Agriculture Organization of the United Nations 2015).

12 Arets, E. J. M. M. & Veeneklaas, F. R. Costs and benefits of a more sustainable production of tropical timber. WOt-technical report No.10. Report No. 2352-2739, 59 (Wageningen, 2014).

13 Favre, R. & Myint, U. K. *An analysis of the Myanmar edible oil crops sub-sector*. 1 edn, (Rural Infrastructure and Agro-Industries Division, Food and Agriculture Organization of the United Nations, 2009).

14 Kyi, A. *Enhancing the sustainable development of diverse agriculture through CGPRT crops in Myanmar: current status of CGPRT crop agriculture and identification of its development constraints* (UNESCAP-CAPSA, 2005).

15 Woods, K. in *Land grabbing, conflict and agrarian‐environmental transformations: perspectives from East and Southeast Asia Iune 5-6.* (BRICS Initiatives for Critical Agrarian Studies (BICAS),MOSAIC Research Project,Land Deal Politics Initiative (LDPI),RCSD Chiang Mai University & Transnational Institute).

16 Social Policy and Poverty Research Group. Characteristics of poorests, poor, and near-poor households in rural Myanmar. (Myanmar, 2011).

17 Reddy, A. A., Mathur, V. C., Yadav, S. S. & Yadav, M. in *Chickpea breeding and management* (eds S. S. Yadav, R. J. Redden, W. Chen, & B. Sharma) Ch. 14, 292-321 (CAB International, 2007).

18 Prasad, R. (ed National academy of agricultural sciences) (National academy of agricultural sciences, New Delhi, 2009).

19 Directorate of Economics and Statistics. (ed Directorate of Economics and Statistics) (Directorate of Economics and Statistics, India, 2013).

20 Howeler, R. in *Partnership in Modern Science to Develop a Strong Cassava Commercial Sector in Africa and Appropriate Varieties by 2020* (Bellagio, Italy, 2006).

21 Edison, S., Anantharaman, M. & Srinivas, T. *Status of cassva in India: an overall view*. (Central Tuber Crops Research Institute, 2006).

22 Rahman, Q. L. U., Sajjad, M., Khar, N., Shahenshah & Nazir, M. Costs and net returns of tobacco production in district Swabi (Khyber Pakhtunkawa) Pakistan. *Interdisciplinary journal of contemporary research in business* **3**, 160-171 (2011).

23 MegaTech International. *Fertilizer. MegaTech International,*, <<http://www.cement-plant.com/npk-npks-mix-blended-fertilizer-plant.html>> (2016).

24 Asian Development Bank. Proposed loan: Republic of the Union of Myanmar: Maubin–Phyapon road rehabilitation project Report No. 47086, (Philippines, 2014).

25 Asian Development Bank. Proposed loan: Republic of the Union of Myanmar: power distribution improvement project. Report No. 46390, (Philippines, 2013).

26 GBP International. *Investment opportunities in Myanmar. GBP International*, <<http://www.gbp-international.com/fileadmin/user_upload/media/News/Investments_Myanmar.pdf?PHPSESSID=a8680clk0bk4hatu8khco0e3o3>> (2012).

27 Corley, R. H. V. & Tinker, P. B. *The oil palm*. 4 edn, 592 (Blackwell, 2008).

28 Angelsen, A. Policies for reduced deforestation and their impact on agricultural production. *PNAS* **107**, 19639–19644 (2010).

29 Nelson, A. (Office for Official Publications of the European Communities, Luxembourg, 2008).

30 Internatial Development Group. *Country report-Myanmar*. (Building and Construction Authority, 2013).

31 Made-in-China.com. *Sinotruk Howo H7 25 Ton/6X4/dump truck, Focus Technology Co., Ltd*, <<http://wanjintruck.en.made-in-china.com/product/MepnSOtKAXUI/China-Sinotruk-HOWO-H7-25-Ton-6X4-Dump-Truck.html>> (2015).

32 AVCalc LLC. *Aqua-calc.com*, <<http://www.aqua-calc.com/page/density-table>> (2005).

33 Key Technology. *Bulk density averages. Key Technology*, <<http://go.key.net/rs/key/images/Bulk%20Density%20Averages%20100630.pdf>> (2015).

34 Murray, T. J. & Boevey, T. M. C. in *Proceedings of the 55th Annual Congress, South African Sugar Technologists' Association.* 135-138 (South African Sugar Technologists' Association, Durban and Mount Edgecombe).

35 Posada-Henao, J. J. in *5th International Symposium on Highway Geometric Design* (Vancouver, Canada, 2015).

36 Max Energy Co. Ltd. *Disel price list. Max Energy Co. Ltd.*, <<http://maxenergy.com.mm/>> (2014).

37 NASA and METI. *Advanced Spaceborne Thermal Emission and Reflection Radiometer (ASTER) Global Digital Elevation Model Version 2 (GDEM V2)*, <<https://search.earthdata.nasa.gov/search/granules?p=C197265171-LPDAAC_ECS&m=-1.546875!45.28125!1!1!0!0%2C2&q=ELEVATION&ok=ELEVATION>> (2011).

38 Global Administrative Areas. (2015).

39 WCS. *Myanmar biodiversity conservation investment vision* (Wildlife Conservation Society, 2013).

40 Fritz, S. S., L.; McCallum, I.; You, L.; Bun, A.; Moltchanova, E.; Duerauer, M.; Albrecht, F.; Schill, C.; Perger, C.;. Mapping global cropland and field size. *Global Change Biology* **21**, 1980-1992, doi:10.1111/gcb.12838 (2015).

41 Vancutsem, C., Marinho, E., Kayitakire, F., See, L. & Fritz, S. Harmonizing and combining existing land cover/land use datasets for cropland area monitoring at the African continental scale. *Remote Sens* **5**, 19-41, doi:10.3390/rs5010019 (2013).

**Supplementary Tables and Figures**

Supplementary Table S1. Reclassification of Global Land Cover 2009. Class 0 represents cropland, class 1 represents forests, and class NA represents miscellaneous land, which was not included in the model.

| GLC2009 | Description | Class |
| --- | --- | --- |
| 11 | Post-flooding or irrigated croplands (or aquatic) | 0 |
| 14 | Rainfed croplands | 0 |
| 20 | Mosaic cropland (50-70%) / vegetation (grassland/shrubland/forest) (20-50%) | 0 |
| 30 | Mosaic vegetation (grassland/shrubland/forest) (50-70%) / cropland (20-50%) | 0 |
| 40 | Closed to open (>15%) broadleaved evergreen or semi-deciduous forest (>5m) | 1 |
| 50 | Closed (>40%) broadleaved deciduous forest (>5m) | 1 |
| 60 | Open (15-40%) broadleaved deciduous forest/woodland (>5m) | 1 |
| 70 | Closed (>40%) needle leaved evergreen forest (>5m) | 1 |
| 100 | Closed to open (>15%) mixed broadleaved and needle leaved forest (>5m) | 1 |
| 110 | Mosaic forest or shrubland (50-70%) / grassland (20-50%) | 1 |
| 120 | Mosaic grassland (50-70%) / forest or shrubland (20-50%) | 1 |
| 130 | Closed to open (>15%) (broadleaved or needle leaved, evergreen or deciduous) shrubland (<5m) | 1 |
| 140 | Closed to open (>15%) herbaceous vegetation (grassland, savannas or lichens/mosses) | NA |
| 150 | Sparse (<15%) vegetation | NA |
| 160 | Closed to open (>15%) broadleaved forest regularly flooded (semi-permanently or temporarily) - Fresh or brackish water | 1 |
| 170 | Closed (>40%) broadleaved forest or shrubland permanently flooded - Saline or brackish water | 1 |
| 190 | Artificial surfaces and associated areas (Urban areas >50%) | NA |
| 200 | Bare areas | NA |
| 210 | Water bodies | NA |
| 220 | Permanent snow and ice | NA |

Supplementary Table S2. Summary of spatial layers used in the statistical models

| Spatial layer | Source | Extent | Resolution (degree) | Data type |
| --- | --- | --- | --- | --- |
| Land use distribution | GLC2009 ^3^ | Global | 0.0028 | Raster |
| Actual yield | ^6^ | Global | 0.083 | Raster |
| Potential yield | ^7^ | Global | 0.083 | Raster |
| Accessibility | ^29^ | Global | 0.0083 | Raster |
| Elevation | ^37^ | Global | 0.0083 | Raster |
| Socio-political stability | ^38^ | Global | NA | Polygon |
| PA status | ^39^ | Myanmar | NA | Polygon |
| KBA distribution | ^39^ | Myanmar | NA | Polygon |

Supplementary Table S3. Mixed effects model selection table for all the 11 crops showing models tested during the analysis to investigate factors influencing current land use patterns in Myanmar and their AIC for each crop (Sesame and rubber were excluded due to quasi-perfect separation issue and heteroscedasticity issue respectively). AG=Agricultural value, T=Transportation cost, E=Elevation, S=Socio-political stability, PA=PA status. Blank indicates that the model did not converge.

| Model formula | Maize | Rice | Chickpea | Pigeon pea | Cassava | Potato | Sugarcane | Tobacco | Groundnut | Sunflower | Oil Palm |
| --- | --- | --- | --- | --- | --- | --- | --- | --- | --- | --- | --- |
| Null | 16,369 | 16,369 | 16,369 | 16,369 | 16,369 | 16,369 | 16,369 | 16,369 | 16,369 | 16,369 | 16,369 |
| AG | 15,351 | 15,405 | 15,561 | 15,244 | 16,115 | 15,928 | 15,139 | 16,338 | 15,368 | 15,301 | 16,360 |
| T | 15,655 | 15,484 | 15,520 | 15,466 | 15,676 | 15,434 | 15,540 | 15,427 | 15,446 | 15,578 |  |
| E | 14,897 | 14,897 | 14,897 | 14,897 | 14,897 | 14,897 | 14,897 | 14,897 | 14,897 | 14,897 | 14,897 |
| S | 16,308 | 16,308 | 16,308 | 16,308 | 16,308 | 16,308 | 16,308 | 16,308 | 16,308 | 16,308 | 16,308 |
| PA | 16,294 | 16,294 | 16,294 | 16,294 | 16,294 | 16,294 | 16,294 | 16,294 | 16,294 | 16,294 | 16,294 |
| AG+T | 15,089 | 15,084 | 15,085 | 15,055 | 15,350 | 15,055 | 15,060 | 15,087 | 15,046 | 15,086 |  |
| AG+E |  | 14,278 | 14,429 | 14,272 | 14,771 | 14,672 | 14,202 | 14,879 | 14,338 | 14,284 | 14,884 |
| AG+S | 15,282 | 15,328 | 15,452 | 15,170 | 16,043 | 15,871 | 15,063 | 16,279 | 15,303 | 15,222 | 16,299 |
| AG+PA | 15,294 | 15,347 | 15,513 | 15,196 | 16,054 | 15,876 | 15,082 | 16,267 | 15,320 | 15,252 | 16,284 |
| T+E | 14,570 | 14,466 | 14,480 |  | 14,564 | 14,404 | 14,497 | 14,390 | 14,420 | 14,523 |  |
| T+S | 15,584 | 15,417 | 15,444 | 15,391 | 15,619 | 15,352 | 15,469 | 15,347 | 15,365 | 15,514 |  |
| T+PA | 15,581 | 15,422 | 15,452 | 15,398 | 15,614 | 15,366 | 15,478 | 15,358 | 15,379 | 15,508 |  |
| E+S | 14,868 | 14,868 | 14,868 | 14,868 | 14,868 | 14,868 | 14,868 | 14,868 | 14,868 | 14,868 | 14,868 |
| E+PA | 14,844 | 14,844 | 14,844 | 14,844 | 14,844 | 14,844 | 14,844 | 14,844 | 14,844 | 14,844 | 14,844 |
| S+PA | 16,230 | 16,230 | 16,230 | 16,230 | 16,230 | 16,230 | 16,230 | 16,230 | 16,230 | 16,230 | 16,230 |
| AG+T+S | 15,014 | 15,009 | 15,007 | 14,978 | 15,282 | 14,980 | 14,984 | 15,011 | 14,970 | 15,009 |  |
| AG+E+S | 14,281 |  |  |  | 14,739 | 14,648 |  | 14,852 | 14,313 | 14,246 | 14,855 |
| AG+T+E |  |  |  |  |  | 14,151 | 14,168 | 14,160 | 14,145 | 14,173 |  |
| T+E+S | 14,536 | 14,435 | 14,446 | 14,390 | 14,537 |  |  |  | 14,382 | 14,495 |  |
| AG+T+PA | 15,029 | 15,027 | 15,031 | 15,001 | 15,294 | 15,000 | 15,004 | 15,030 | 14,991 | 15,028 |  |
| AG+E+PA |  |  |  |  | 14,723 | 14,627 | 14,153 | 14,828 | 14,295 |  | 14,831 |
| AG+S+PA | 15,221 | 15,265 | 15,439 | 15,118 | 15,980 | 15,817 | 15,002 | 16,205 | 15,252 | 15,170 | 16,221 |
| T+E+PA |  |  |  |  |  |  | 14,446 | 14,333 | 14,365 | 14,467 |  |
| T+S+PA | 15,507 | 15,352 | 15,371 | 15,317 | 15,554 | 15,279 | 15,403 | 15,273 | 15,294 | 15,439 |  |
| AG+T+E+S |  |  |  |  |  |  |  |  | 14,113 |  |  |
| AG+T+E+PA | 14,122 |  |  |  |  |  |  | 14,112 | 14,098 |  |  |
| AG+T+S+PA | 14,950 | 14,947 |  |  | 15,223 | 14,921 | 14,923 | 14,950 | 14,911 | 14,947 |  |
| AG+E+S+PA |  |  |  |  | 14,690 | 14,602 |  |  | 14,268 |  | 14,800 |
| T+E+S+PA |  | 14,381 |  |  |  | 14,307 |  |  |  | 14,437 |  |
| AG+T+E+S+PA |  |  |  |  |  |  |  |  |  |  |  |
| AG+T+E+PA+AG*PA |  | 14,134 | 14,128 |  |  |  | 14,109 |  |  | 14,120 |  |
| AG+T+E+PA+T*PA | 14,104 |  |  |  |  |  |  |  |  |  |  |
| AG+T+E+PA+E*PA |  |  |  |  |  | 14,053 |  | 14,062 |  |  |  |
| AG+T+S+PA+AG*PA | 14,951 |  |  |  |  |  |  |  |  |  |  |
| AG+T+S+PA+T*PA |  |  |  | 14,903 |  |  |  |  |  |  |  |
| AG+E+S+PA+AG*PA |  | 14,278 |  | 14,187 | 14,692 |  |  |  |  |  |  |
| AG+E+S+PA+E*PA |  |  |  |  |  |  |  | 14,744 |  |  | 14,744 |
| AG+E+S+PA+S*PA |  | 14,272 |  | 14,684 |  |  |  |  |  |  |  |
| T+E+S+PA+T*PA |  |  |  |  |  | 14,307 | 14,390 |  | 14,326 |  |  |
| T+E+S+PA+E*PA | 14,419 |  |  |  |  |  |  |  |  |  |  |
| T+E+S+PA+S*PA |  |  |  |  |  |  | 14,404 |  |  |  |  |
| AG+T+E+PA+AG*PA+E*PA |  |  |  |  |  | 14,054 |  |  |  |  |  |
| T+E+S+PA+T*PA+S*PA |  |  |  |  |  |  |  | 14,286 |  |  |  |

Supplementary Table S4. List of freshwater and terrestrial KBAs in Myanmar showing probability of forest presence within each KBA projected under the combined scenario for 11 crops ((sesame and rubber were excluded due to lack of potential yield data). The abbreviations are as follows: T=terrestrial KBAs, F=freshwater KBAs

| ID | KBA name | Area (km^2^) | Type | PA status | Maize | Rice | Chickpea | Pigeon pea | Cassava | Potato | Sugarcane | Tobacco | Groundnut | Sunflower | Oil palm | Average |
| --- | --- | --- | --- | --- | --- | --- | --- | --- | --- | --- | --- | --- | --- | --- | --- | --- |
| 1 | Alaungdaw Kathapa N.P | 1,433 | T | Yes | 0.01 | 0.82 | 0.91 | 0.71 | 0.91 | 0.83 | 0.86 | 0.81 | 0.61 | 0.90 | 0.85 | 0.75 |
| 2 | Ayeyarwady River (Bagan Section) | 342 | F | No | 0.00 | 0.00 | 0.00 | 0.00 | 0.00 | 0.01 | 0.00 | 0.00 | 0.00 | 0.00 | 0.00 | 0.00 |
| 3 | Ayeyarwady River (Bhamo to Shwegu Section) | 200 | F | No | 0.10 | 0.51 | 0.60 | 0.01 | 0.52 | 0.71 | 0.63 | 0.73 | 0.57 | 0.58 | 0.26 | 0.47 |
| 4 | Ayeyarwady River (Moda Section) | 303 | F | No | 0.10 | 0.25 | 0.32 | 0.01 | 0.59 | 0.44 | 0.33 | 0.46 | 0.27 | 0.29 | 0.30 | 0.31 |
| 5 | Ayeyarwady River (Myitkyina to Sinbo Section) | 578 | F | No | 0.01 | 0.19 | 0.54 | 0.00 | 0.39 | 0.66 | 0.45 | 0.72 | 0.18 | 0.19 | 0.30 | 0.33 |
| 6 | Ayeyarwady River (Sinbyugyun to Minbu Section) | 540 | F | No | 0.00 | 0.00 | 0.01 | 0.00 | 0.05 | 0.01 | 0.00 | 0.01 | 0.00 | 0.01 | 0.01 | 0.01 |
| 7 | Ayeyarwady River (Singu Section) | 75 | F | No | 0.00 | 0.04 | 0.09 | 0.02 | 0.20 | 0.30 | 0.10 | 0.13 | 0.01 | 0.07 | 0.30 | 0.12 |
| 8 | Bumhpabum W.S | 2,939 | T | Yes | 0.33 | 1.00 | 1.00 | 0.99 | 1.00 | 1.00 | 1.00 | 1.00 | 1.00 | 1.00 | 1.00 | 0.94 |
| 9 | Bwe Pa | 152 | T | No | 0.67 | 0.96 | 0.95 | 0.86 | 0.90 | 0.95 | 0.94 | 0.97 | 0.96 | 0.88 | 0.90 | 0.90 |
| 10 | Central Bago Yoma | 3,951 | T | No | 0.19 | 0.40 | 0.64 | 0.06 | 0.74 | 0.70 | 0.49 | 0.64 | 0.40 | 0.51 | 0.59 | 0.49 |
| 12 | Chatthin W.S | 284 | T | Yes | 0.00 | 0.27 | 0.39 | 0.17 | 0.35 | 0.42 | 0.36 | 0.43 | 0.09 | 0.38 | 0.22 | 0.28 |
| 13 | Chaungmon-Wachaung | 516 | T | No | 0.83 | 0.96 | 0.97 | 0.56 | 0.99 | 0.97 | 0.96 | 0.97 | 0.96 | 0.97 | 0.81 | 0.90 |
| 14 | Dawna Range | 1,264 | T | No | 0.44 | 0.63 | 0.77 | 0.07 | 0.62 | 0.82 | 0.69 | 0.78 | 0.63 | 0.66 | 0.49 | 0.60 |
| 15 | Fen-shui-ling Valley | 146 | T | No | 1.00 | 1.00 | 1.00 | 1.00 | 1.00 | 1.00 | 1.00 | 1.00 | 1.00 | 1.00 | 1.00 | 1.00 |
| 16 | Gyobin | 161 | T | No | 0.00 | 0.01 | 0.05 | 0.00 | 0.21 | 0.08 | 0.02 | 0.06 | 0.01 | 0.03 | 0.09 | 0.05 |
| 17 | Hkakaborazi N.P | 4,313 | T | Yes | 0.91 | 1.00 | 1.00 | 1.00 | 0.98 | 1.00 | 1.00 | 0.99 | 1.00 | 1.00 | 0.94 | 0.98 |
| 18 | Hpa-an | 115 | T | No | 0.00 | 0.02 | 0.17 | 0.00 | 0.04 | 0.25 | 0.06 | 0.17 | 0.02 | 0.09 | 0.02 | 0.08 |
| 19 | Hponkanrazi W.S | 2,803 | T | Yes | 0.78 | 1.00 | 1.00 | 0.97 | 0.95 | 0.99 | 1.00 | 0.99 | 1.00 | 1.00 | 0.89 | 0.96 |
| 20 | Htamanthi W.S | 2,542 | T | Yes | 0.07 | 0.96 | 0.96 | 0.83 | 0.99 | 0.97 | 0.95 | 0.98 | 0.94 | 0.96 | 0.99 | 0.87 |
| 21 | Htaung Pru | 285 | T | No | 0.53 | 0.74 | 0.86 | 0.17 | 0.94 | 0.85 | 0.80 | 0.86 | 0.74 | 0.83 | 0.48 | 0.71 |
| 22 | Hukaung Valley W.S | 6,483 | T | Yes | 0.16 | 1.00 | 1.00 | 0.95 | 0.98 | 1.00 | 1.00 | 1.00 | 0.99 | 1.00 | 0.98 | 0.91 |
| 23 | Indawgyi W.S | 737 | T | Yes | 0.12 | 0.83 | 0.88 | 0.37 | 0.64 | 0.90 | 0.87 | 0.91 | 0.71 | 0.86 | 0.68 | 0.71 |
| 24 | Inlay Wetland W.S | 554 | F | Yes | 0.01 | 0.26 | 0.41 | 0.05 | 0.07 | 0.20 | 0.43 | 0.18 | 0.08 | 0.36 | 0.04 | 0.19 |
| 25 | Kamaing | 588 | T | No | 0.01 | 0.56 | 0.86 | 0.19 | 0.62 | 0.79 | 0.63 | 0.80 | 0.37 | 0.36 | 0.48 | 0.52 |
| 26 | Karathuri | 238 | T | No | 0.81 | 0.90 | 0.92 | 0.57 | 0.96 | 0.92 | 0.90 | 0.92 | 0.90 | 0.91 | 0.72 | 0.86 |
| 27 | Kawthaung District Lowlands | 414 | T | No | 0.78 | 0.90 | 0.83 | 0.43 | 0.93 | 0.81 | 0.84 | 0.82 | 0.88 | 0.84 | 0.55 | 0.78 |
| 28 | Kennedy Peak | 108 | T | No | 0.73 | 0.92 | 0.92 | 0.82 | 0.62 | 0.93 | 0.91 | 0.93 | 0.91 | 0.92 | 0.71 | 0.85 |
| 29 | Kyaukpantaung W.S | 129 | T | Yes | 0.28 | 0.97 | 0.99 | 0.71 | 0.96 | 0.99 | 0.97 | 0.98 | 0.96 | 0.97 | 0.87 | 0.88 |
| 33 | Lenya N.P | 1,846 | T | Yes | 0.24 | 0.99 | 0.99 | 0.91 | 0.99 | 0.99 | 0.99 | 0.99 | 0.98 | 0.99 | 0.96 | 0.91 |
| 34 | Loimwe P.A | 43 | T | Yes | 0.16 | 0.59 | 0.45 | 0.01 | 0.77 | 0.86 | 0.78 | 0.89 | 0.36 | 0.30 | 0.75 | 0.54 |
| 35 | Mahamyaing W.S | 1,204 | T | Yes | 0.07 | 0.90 | 0.92 | 0.73 | 0.97 | 0.93 | 0.90 | 0.93 | 0.82 | 0.92 | 0.94 | 0.82 |
| 36 | Mahanandar Kan | 78 | F | No | 0.00 | 0.01 | 0.02 | 0.00 | 0.07 | 0.06 | 0.01 | 0.02 | 0.00 | 0.01 | 0.02 | 0.02 |
| 38 | Mawlamyine | 90 | T | No | 0.00 | 0.04 | 0.27 | 0.00 | 0.06 | 0.40 | 0.11 | 0.29 | 0.05 | 0.16 | 0.02 | 0.13 |
| 39 | Mehon (Doke-hta Wady River) | 881 | F | No | 0.15 | 0.36 | 0.28 | 0.01 | 0.46 | 0.50 | 0.40 | 0.48 | 0.31 | 0.33 | 0.30 | 0.33 |
| 40 | Minzontaung W.S | 17 | T | Yes | 0.00 | 0.00 | 0.00 | 0.00 | 0.00 | 0.01 | 0.00 | 0.00 | 0.00 | 0.00 | 0.00 | 0.00 |
| 41 | Momeik-Mabein | 2,821 | T | No | 0.39 | 0.57 | 0.68 | 0.07 | 0.67 | 0.84 | 0.71 | 0.83 | 0.58 | 0.64 | 0.27 | 0.57 |
| 42 | Moyungyi Wetland W.S | 103 | F | Yes | 0.00 | 0.05 | 0.15 | 0.04 | 0.06 | 0.37 | 0.16 | 0.29 | 0.01 | 0.13 | 0.21 | 0.13 |
| 43 | Myaleik Taung | 37 | T | No | 0.00 | 0.03 | 0.01 | 0.00 | 0.21 | 0.14 | 0.04 | 0.07 | 0.01 | 0.02 | 0.20 | 0.07 |
| 44 | Myinmoletkhat | 8,131 | T | No | 0.83 | 0.91 | 0.97 | 0.73 | 0.98 | 0.97 | 0.94 | 0.97 | 0.92 | 0.94 | 0.90 | 0.91 |
| 45 | Myitkyina-Nandebad-Talawgyi | 554 | T | No | 0.08 | 0.24 | 0.56 | 0.00 | 0.40 | 0.67 | 0.48 | 0.74 | 0.22 | 0.27 | 0.29 | 0.36 |
| 46 | Myittha Lakes | 37 | F | No | 0.00 | 0.00 | 0.02 | 0.00 | 0.05 | 0.04 | 0.01 | 0.02 | 0.00 | 0.01 | 0.04 | 0.02 |
| 47 | Nadi Kan | 37 | F | No | 0.00 | 0.02 | 0.01 | 0.00 | 0.06 | 0.19 | 0.05 | 0.19 | 0.02 | 0.01 | 0.04 | 0.05 |
| 48 | Nam Sam Chaung (Kachin State) | 458 | T | No | 0.09 | 0.39 | 0.63 | 0.01 | 0.51 | 0.74 | 0.60 | 0.78 | 0.37 | 0.39 | 0.33 | 0.44 |
| 49 | Nam San Valley (Shan State) | 2,003 | T | No | 0.09 | 0.23 | 0.18 | 0.00 | 0.29 | 0.44 | 0.32 | 0.42 | 0.22 | 0.21 | 0.11 | 0.23 |
| 50 | Natmataung N.P | 1,100 | T | Yes | 0.40 | 1.00 | 0.99 | 0.97 | 0.98 | 0.92 | 1.00 | 0.94 | 0.99 | 0.99 | 0.83 | 0.91 |
| 51 | Nat-yekan | 160 | T | No | 0.68 | 0.80 | 0.80 | 0.30 | 0.97 | 0.92 | 0.86 | 0.91 | 0.79 | 0.77 | 0.95 | 0.79 |
| 52 | Ngawun/ Lenya N.P (Extension) | 1,851 | T | Yes | 0.08 | 0.99 | 0.97 | 0.93 | 0.99 | 0.99 | 0.98 | 0.99 | 0.98 | 0.98 | 0.91 | 0.89 |
| 53 | Ngwe Taung | 733 | T | No | 0.45 | 0.65 | 0.73 | 0.05 | 0.68 | 0.73 | 0.68 | 0.74 | 0.65 | 0.67 | 0.42 | 0.59 |
| 54 | Ninety-six Inns | 587 | T | No | 0.03 | 0.26 | 0.44 | 0.00 | 0.33 | 0.56 | 0.40 | 0.60 | 0.24 | 0.24 | 0.20 | 0.30 |
| 55 | North Zarmayi | 99 | T | No | 0.29 | 0.63 | 0.84 | 0.08 | 0.91 | 0.87 | 0.73 | 0.84 | 0.63 | 0.68 | 0.80 | 0.66 |
| 56 | Northern Rakhine Yoma | 1,303 | T | No | 0.40 | 0.60 | 0.63 | 0.01 | 0.53 | 0.60 | 0.58 | 0.64 | 0.59 | 0.61 | 0.09 | 0.48 |
| 57 | Pachan | 608 | T | No | 0.63 | 0.79 | 0.77 | 0.30 | 0.90 | 0.73 | 0.76 | 0.74 | 0.80 | 0.77 | 0.33 | 0.68 |
| 58 | Panlaung Pyadalin Cave W.S | 349 | T | Yes | 0.04 | 0.54 | 0.57 | 0.08 | 0.30 | 0.61 | 0.58 | 0.56 | 0.36 | 0.60 | 0.22 | 0.41 |
| 59 | Paunglong Catchment Area | 2,550 | T | No | 0.17 | 0.33 | 0.31 | 0.02 | 0.52 | 0.57 | 0.42 | 0.54 | 0.33 | 0.29 | 0.40 | 0.35 |
| 60 | Peleik Inn | 37 | F | No | 0.00 | 0.00 | 0.01 | 0.00 | 0.01 | 0.02 | 0.00 | 0.01 | 0.00 | 0.00 | 0.01 | 0.01 |
| 61 | Rakhine Yoma Elephant Range | 1,713 | T | Yes | 0.02 | 1.00 | 0.99 | 0.90 | 0.97 | 0.99 | 0.99 | 1.00 | 1.00 | 1.00 | 0.89 | 0.89 |
| 62 | Saramati Taung | 1,065 | T | No | 1.00 | 1.00 | 1.00 | 1.00 | 1.00 | 1.00 | 1.00 | 1.00 | 1.00 | 1.00 | 1.00 | 1.00 |
| 63 | Shwe U Daung W.S | 183 | T | Yes | 0.27 | 0.82 | 0.82 | 0.48 | 0.79 | 0.92 | 0.89 | 0.91 | 0.73 | 0.82 | 0.80 | 0.75 |
| 64 | Shwesettaw W.S | 497 | T | Yes | 0.00 | 0.11 | 0.12 | 0.05 | 0.14 | 0.23 | 0.14 | 0.17 | 0.04 | 0.14 | 0.16 | 0.12 |
| 65 | Tanai River | 636 | F | No | 0.12 | 0.72 | 0.93 | 0.02 | 0.87 | 0.93 | 0.88 | 0.94 | 0.76 | 0.76 | 0.78 | 0.70 |
| 66 | Taninthayi N.P | 3,663 | T | Yes | 0.11 | 0.96 | 0.96 | 0.86 | 0.96 | 0.98 | 0.96 | 0.98 | 0.91 | 0.96 | 0.94 | 0.87 |
| 67 | Taninthayi N.R | 1,619 | T | Yes | 0.04 | 0.89 | 0.96 | 0.80 | 0.96 | 0.98 | 0.93 | 0.97 | 0.88 | 0.93 | 0.93 | 0.84 |
| 68 | Taung Kan at Sedawgyi | 37 | F | No | 0.00 | 0.03 | 0.12 | 0.00 | 0.27 | 0.15 | 0.04 | 0.08 | 0.02 | 0.04 | 0.19 | 0.09 |
| 69 | Thaungdut | 325 | T | No | 0.68 | 0.84 | 0.83 | 0.28 | 0.98 | 0.85 | 0.84 | 0.87 | 0.81 | 0.83 | 0.91 | 0.79 |
| 70 | Upper Mogaung Chaung Basin | 188 | T | No | 0.03 | 0.14 | 0.58 | 0.00 | 0.35 | 0.61 | 0.40 | 0.67 | 0.13 | 0.16 | 0.30 | 0.31 |
| 71 | Uyu River | 844 | F | No | 0.24 | 0.59 | 0.71 | 0.04 | 0.91 | 0.75 | 0.67 | 0.80 | 0.55 | 0.58 | 0.80 | 0.60 |
| 72 | Yemyet Inn | 42 | F | No | 0.00 | 0.00 | 0.02 | 0.00 | 0.05 | 0.02 | 0.00 | 0.01 | 0.00 | 0.00 | 0.02 | 0.01 |
| 73 | Zeihmu Range | 81 | T | No | 0.31 | 0.87 | 0.88 | 0.53 | 0.60 | 0.86 | 0.83 | 0.89 | 0.88 | 0.70 | 0.65 | 0.73 |
| 74 | Ayeyarwady River (Bhamo) | 102 | F | No | 0.00 | 0.00 | 0.00 | 0.00 | 0.41 | 0.63 | 0.48 | 0.65 | 0.35 | 0.33 | 0.24 | 0.28 |
| 75 | Ayeyarwady River (Shwegu) | 373 | F | No | 0.03 | 0.11 | 0.20 | 0.01 | 0.56 | 0.34 | 0.19 | 0.35 | 0.11 | 0.13 | 0.16 | 0.20 |
| 76 | Babulon Htan | 1,896 | T | No | 0.93 | 0.97 | 0.97 | 0.66 | 0.88 | 0.99 | 0.97 | 0.99 | 0.98 | 0.98 | 0.91 | 0.93 |
| 81 | Hlawga Park | 6 | T | Yes | 0.00 | 0.00 | 0.07 | 0.00 | 0.07 | 0.13 | 0.02 | 0.08 | 0.01 | 0.03 | 0.06 | 0.04 |
| 82 | Hlawga Reservior | 23 | T | No | 0.00 | 0.01 | 0.11 | 0.00 | 0.11 | 0.21 | 0.03 | 0.13 | 0.01 | 0.05 | 0.12 | 0.07 |
| 83 | Hukaung Valley W.S (Extension) | 11,348 | T | Yes | 0.09 | 0.99 | 0.99 | 0.91 | 0.96 | 0.99 | 0.99 | 0.99 | 0.96 | 0.99 | 0.96 | 0.89 |
| 84 | Indawgyi grassland and Indaw chaung wetland | 258 | F | No | 0.05 | 0.41 | 0.57 | 0.07 | 0.41 | 0.65 | 0.52 | 0.68 | 0.38 | 0.42 | 0.30 | 0.41 |
| 85 | Irrawaddy Dolphin P.A | 326 | F | No | 0.00 | 0.35 | 0.48 | 0.26 | 0.35 | 0.72 | 0.51 | 0.57 | 0.09 | 0.49 | 0.75 | 0.42 |
| 88 | Kahilu W.S | 127 | T | Yes | 0.05 | 0.59 | 0.85 | 0.19 | 0.42 | 0.90 | 0.74 | 0.87 | 0.49 | 0.78 | 0.48 | 0.58 |
| 90 | Kelatha W.S | 25 | T | Yes | 0.01 | 0.65 | 0.85 | 0.23 | 0.17 | 0.96 | 0.88 | 0.94 | 0.26 | 0.84 | 0.64 | 0.59 |
| 92 | Kyaikhtiyoe W.S | 137 | T | Yes | 0.25 | 1.00 | 0.99 | 0.86 | 0.96 | 0.99 | 0.99 | 1.00 | 1.00 | 0.99 | 0.90 | 0.90 |
| 93 | Lwoilin/ Ginga mountain | 548 | T | No | 0.54 | 0.78 | 0.75 | 0.32 | 0.72 | 0.86 | 0.84 | 0.89 | 0.73 | 0.71 | 0.64 | 0.71 |
| 94 | Maletto Inn | 386 | F | No | 0.01 | 0.04 | 0.09 | 0.00 | 0.11 | 0.11 | 0.06 | 0.10 | 0.04 | 0.07 | 0.08 | 0.06 |
| 95 | Mali Hka Area | 5,129 | T | No | 0.96 | 0.99 | 0.99 | 0.87 | 0.96 | 0.99 | 0.99 | 1.00 | 0.99 | 0.99 | 0.98 | 0.97 |
| 99 | May Hka Area | 10,090 | T | No | 0.90 | 0.94 | 0.92 | 0.85 | 0.84 | 0.96 | 0.95 | 0.96 | 0.93 | 0.93 | 0.89 | 0.91 |
| 101 | Mone Chaung | 15 | F | No | 0.00 | 0.00 | 0.00 | 0.00 | 0.09 | 0.02 | 0.01 | 0.01 | 0.00 | 0.01 | 0.02 | 0.02 |
| 103 | Mulayit W.S | 214 | T | Yes | 0.09 | 0.89 | 0.95 | 0.63 | 0.85 | 0.97 | 0.92 | 0.96 | 0.88 | 0.87 | 0.78 | 0.80 |
| 108 | North Lenyar | 2,650 | T | No | 0.76 | 0.93 | 0.95 | 0.66 | 0.98 | 0.95 | 0.94 | 0.95 | 0.92 | 0.94 | 0.71 | 0.88 |
| 109 | North Zarmayi Elephant PA | 710 | T | No | 0.05 | 0.26 | 0.62 | 0.02 | 0.72 | 0.70 | 0.42 | 0.61 | 0.26 | 0.39 | 0.62 | 0.43 |
| 111 | Parsar P.A | 117 | T | Yes | 0.39 | 0.63 | 0.65 | 0.02 | 0.70 | 0.88 | 0.78 | 0.91 | 0.62 | 0.64 | 0.43 | 0.60 |
| 112 | Pauk Area | 195 | T | No | 0.00 | 0.01 | 0.03 | 0.00 | 0.11 | 0.04 | 0.01 | 0.02 | 0.01 | 0.01 | 0.03 | 0.02 |
| 114 | Phokyar Elephant Camp | 100 | T | No | 0.04 | 0.20 | 0.45 | 0.01 | 0.59 | 0.53 | 0.29 | 0.45 | 0.20 | 0.33 | 0.37 | 0.32 |
| 115 | Pidaung W.S | 150 | T | Yes | 0.17 | 0.95 | 0.97 | 0.66 | 0.71 | 0.98 | 0.98 | 0.99 | 0.79 | 0.98 | 0.88 | 0.82 |
| 116 | Popa Mountain Park | 98 | T | Yes | 0.00 | 0.20 | 0.22 | 0.10 | 0.18 | 0.20 | 0.25 | 0.14 | 0.05 | 0.26 | 0.18 | 0.16 |
| 123 | Shinmataung | 24 | T | No | 0.00 | 0.01 | 0.03 | 0.00 | 0.06 | 0.05 | 0.01 | 0.02 | 0.00 | 0.01 | 0.04 | 0.02 |
| 124 | Taunggyi B.S | 70 | T | Yes | 0.01 | 0.35 | 0.45 | 0.08 | 0.08 | 0.08 | 0.49 | 0.09 | 0.10 | 0.41 | 0.02 | 0.20 |
| 129 | U-do | 5 | T | No | 0.00 | 0.00 | 0.01 | 0.00 | 0.00 | 0.01 | 0.00 | 0.01 | 0.00 | 0.00 | 0.00 | 0.00 |
| 130 | Upper Chindwin (Kaunghein-Padumone) | 45 | F | No | 0.00 | 0.00 | 0.00 | 0.00 | 0.99 | 1.00 | 0.99 | 1.00 | 0.97 | 1.00 | 1.00 | 0.63 |
| 131 | Yaybawmee | 3,213 | T | No | 0.70 | 0.95 | 0.96 | 0.49 | 0.98 | 0.97 | 0.96 | 0.98 | 0.94 | 0.94 | 0.93 | 0.89 |
| 132 | Yelegale | 83 | T | No | 0.00 | 0.01 | 0.05 | 0.00 | 0.06 | 0.08 | 0.02 | 0.06 | 0.01 | 0.03 | 0.05 | 0.03 |

Supplementary Fig. S1. a) Reclassified global land cover 2009 map; and b) Reclassified IIASA-IFPRI cropland percentage map showing the cropland and forest distribution in Myanmar


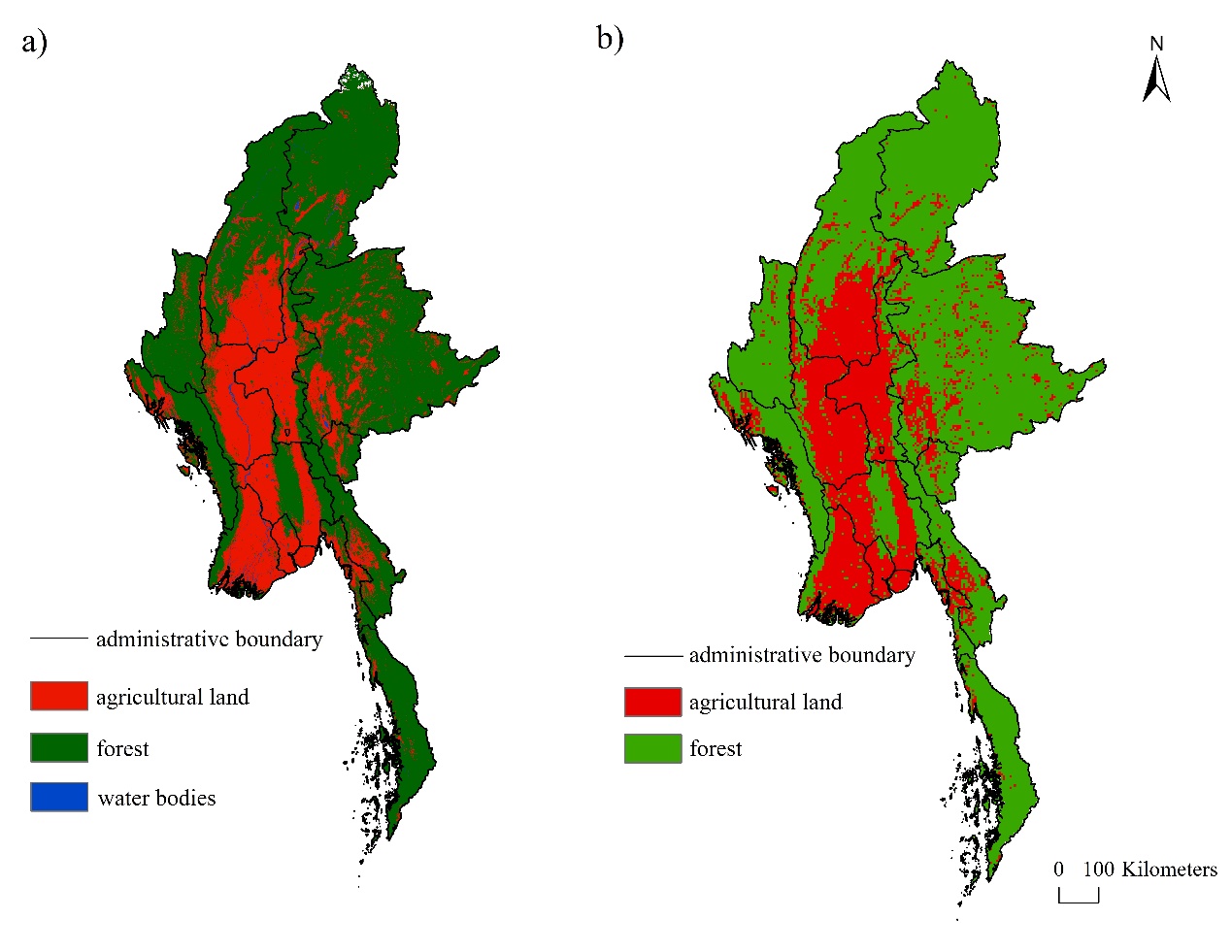


Supplementary Fig. S2. Map of Myanmar showing locations of freshwater and terrestrial KBAs and different administrative divisions.


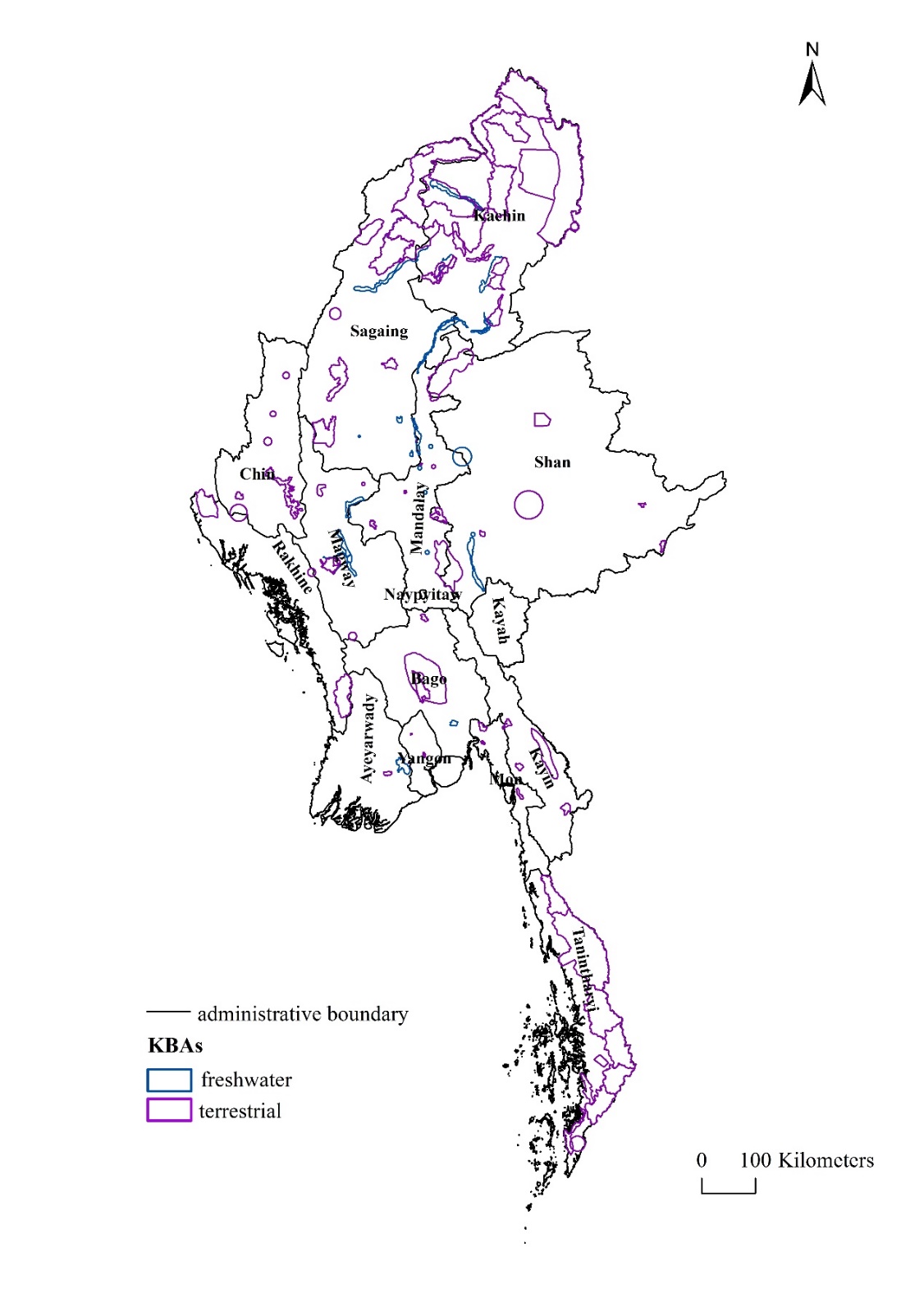


Supplementary Fig. S3. Projected land use distribution by export crop: a) maize; b) rice; c) chickpea; and d) pigeon pea. Crops are under the following scenarios: i) under the baseline scenario; ii) when potential yield is realized; iii) under agricultural value scenario; iv) under transportation cost scenario; v) under scenario stability scenario; and vi) under combined scenario. There are projections under stability scenario and combined scenario only for pigeon pea because only its distribution is significantly influenced by socio-political stability according to the selected best mixed-effects models. However, distribution of pigeon pea is not significantly influenced by transportation cost, and thus there is no projection under transportation cost scenario for it.


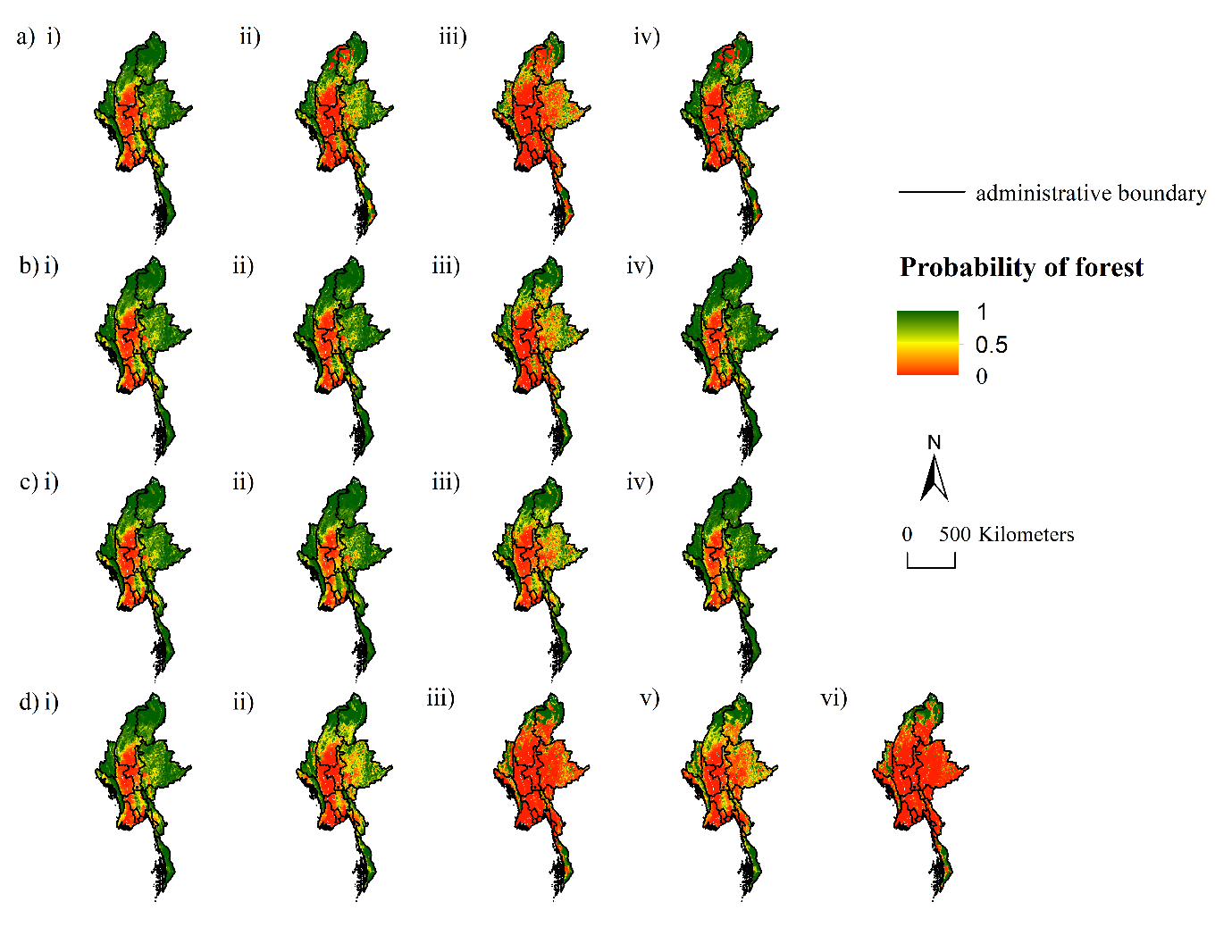


Supplementary Fig. S4. Projected land use distribution by food crop for domestic markets a) cassava; b) potato; and for industrial crops c) sugarcane; d) tobacco. These crops are under the following scenarios: i) under the baseline scenario; ii) when potential yield is realized; iii) under agricultural value scenario; iv) under transportation cost scenario; v) under stability scenario; and vi) under combined scenario. There are projections under stability scenario only for cassava because only its distribution is significantly influenced by socio-political stability according to the selected best mixed–effects models. However, because its distribution is not significantly influenced by agricultural value, it has no projections under agricultural value scenario and projection under combined scenario will be the same as under stability scenario.


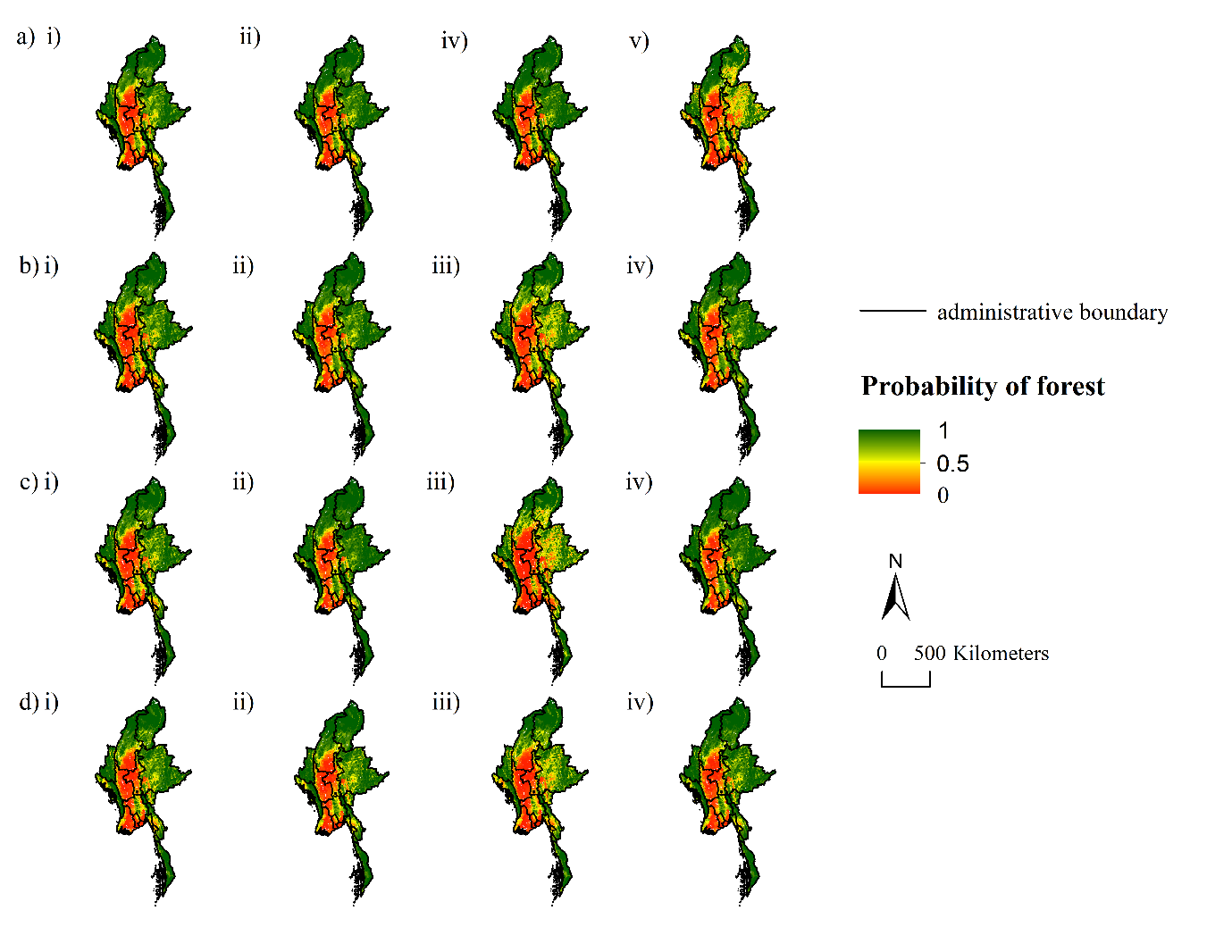


Supplementary Fig. S5. Projected land use distribution by oilseed crop a) sunflower b) groundnut and c) oil palm under the following scenarios: i) under the baseline scenario ii) when potential yield is realized iii) under agricultural value scenario; iv) under transportation cost scenario v) under stability scenario vi) under combined scenario. There are projections under stability scenario and combined scenario only for oil palm because only its distribution is significantly influenced by socio-political stability according to the selected best mixed-effects models. However, distribution of oil palm is not significantly influenced by transportation cost, and thus there is no projection under transportation cost scenario for it.


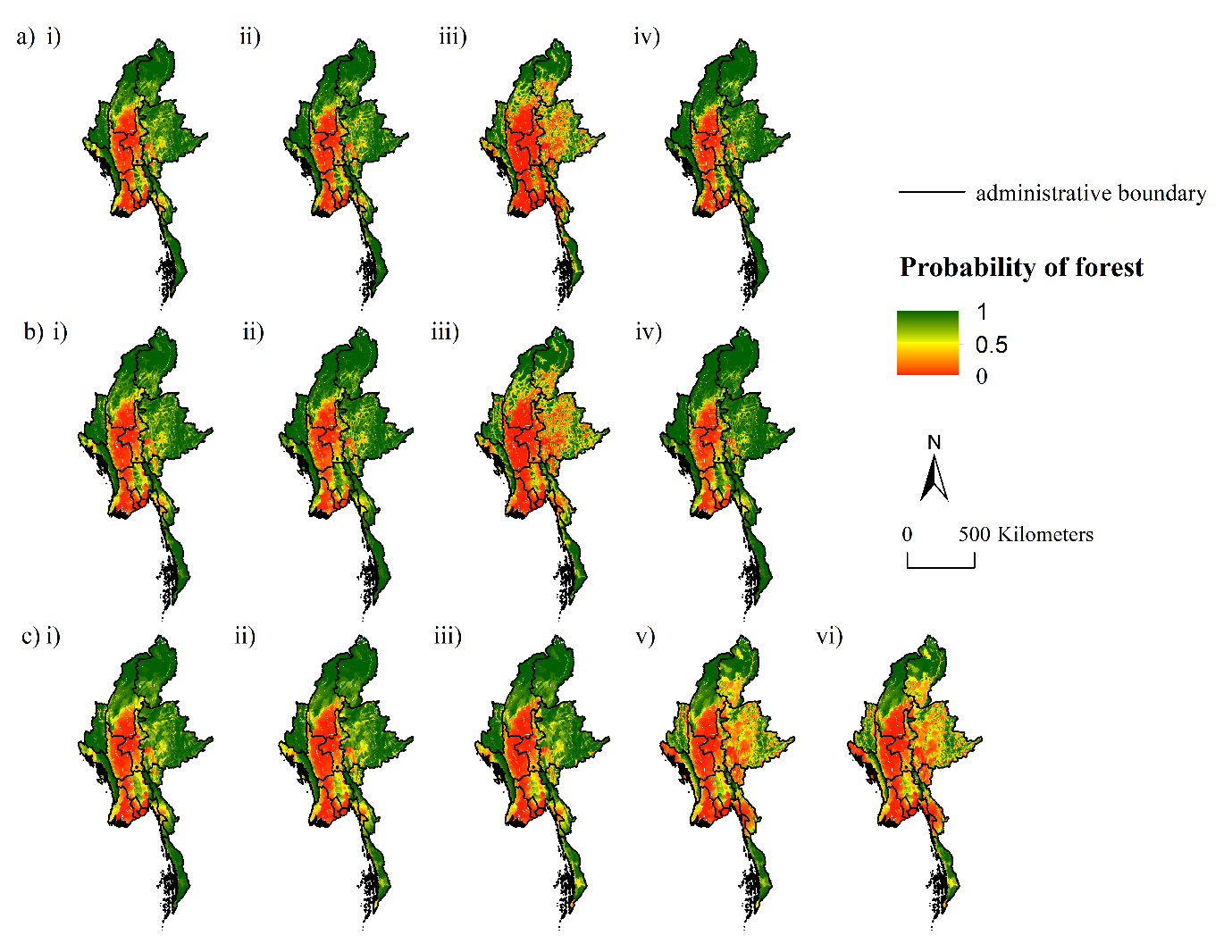


Supplementary Fig. S6. Plot of explanatory variable effect sizes with confidence intervals for the selected best generalized linear mixed-effects models for 13 crops to explain current land use distribution in Myanmar with land use from the IIASA IFPRI dataset used to generate the dependent variable. AG=Agricultural value, T=Transportation cost, E=Elevation, S=Socio-political stability and PA=PA status.


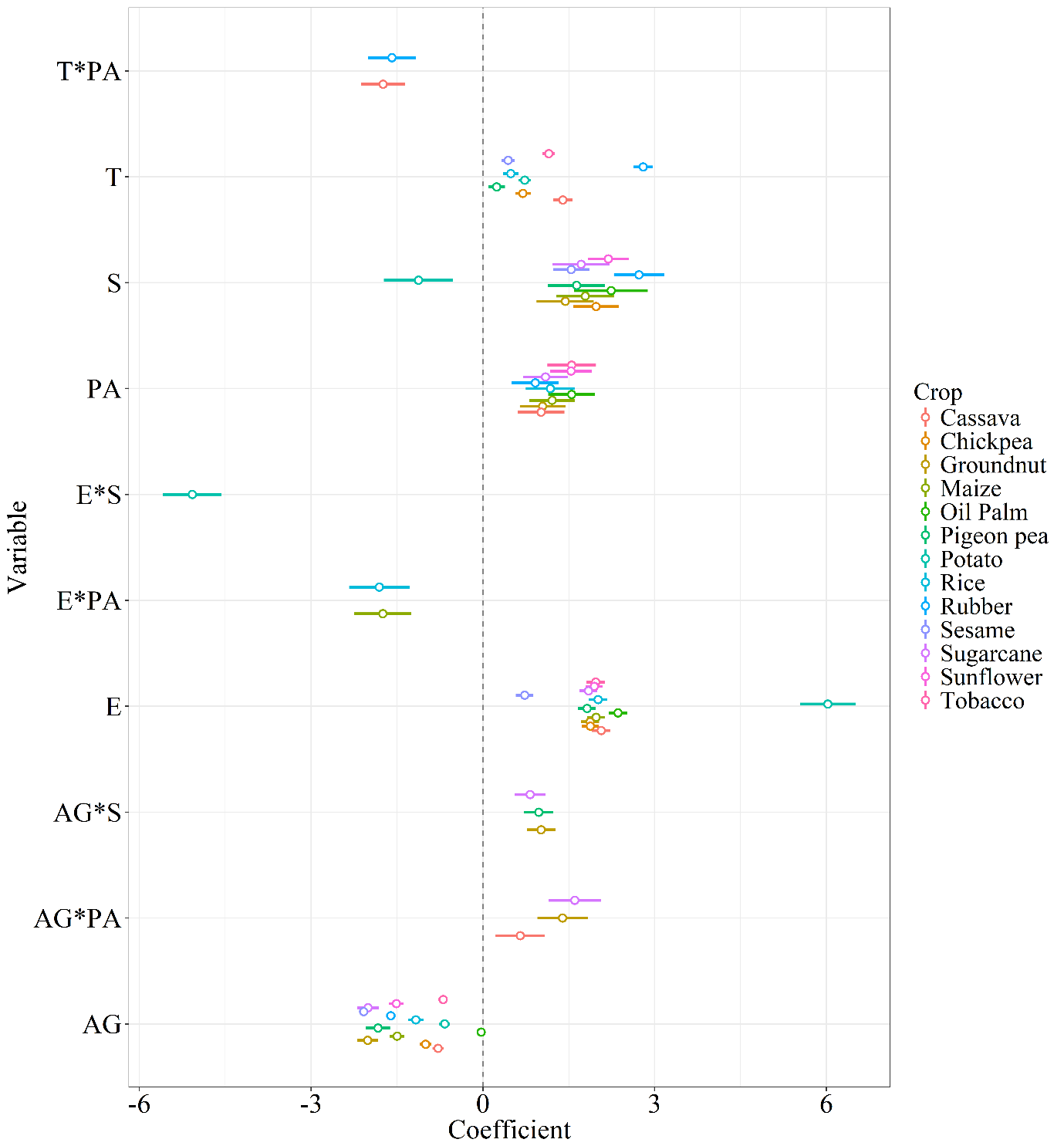


**Supplementary Code**

#############################################################################

###################model land use with characteristics of maize#######################

setwd("D:\\Myanmar project\\Resubmission 2\\Code and data (submit)")

data_all_raman<-read.csv("Appendix_S3_Supplementary_Data.csv",header=T)

data_maizer<-data_all_raman[c(1:23313),]

data_maizer_subset<-data_maizer[data_maizer$land_use!=2,]

#convert to factors data_maizer_subset$land_use <- as.factor(data_maizer_subset$land_use) data_maizer_subset$region_code1 <- as.factor(data_maizer_subset$region_code1) data_maizer_subset$region_code2 <- as.factor(data_maizer_subset$region_code2) data_maizer_subset$region_code3 <- as.factor(data_maizer_subset$region_code3) data_maizer_subset$resid<-as.factor(data_maizer_subset$resid) data_maizer_subset$PA<- as.factor(data_maizer_subset$PA)

colnames(data_maizer_subset) <- c("landuse", "agri", "trans", "elevation",

"region_type","region_code1","region_code2","region_code3","rdist", "resid","PA","ID")

library(lme4)

##check collinearity

library(car)

model_vif <- glm(landuse ~ scale(agri)+scale(trans)+scale(elevation) +region_type+PA, family=binomial, data=data_maizer_subset) summary(model_vif)

vif(model_vif)

###try to fix the random effects first

model_r1 <- glmer(landuse ~ scale(agri)+scale(trans)+scale(elevation) +region_type+PA+

(1|region_code1), family=binomial, data=data_maizer_subset) summary(model_r1)

model_r2 <- glmer(landuse ~ scale(agri)+scale(trans) +scale(elevation)+region_type+PA+

(1|region_code2), family=binomial, data=data_maizer_subset) summary(model_r2)

model_r3 <- glmer(landuse ~ scale(agri)+scale(trans)+scale(elevation) +region_type+PA+

(1|region_code3), family=binomial, data=data_maizer_subset) summary(model_r3)

AIC(model_r1, model_r2, model_r3)

#choose region_code3 with smallest AIC

model_r4 <- glmer(landuse ~ scale(agri)+scale(trans)+scale(elevation) +region_type+PA+ (rdist|region_code3), family=binomial, data=data_maizer_subset) summary(model_r4)

AIC(model_r3,model_r4)

##choose model_r4 with samllest AIC

###one variable### model_null<-glmer(landuse~1+(rdist|region_code3), family=binomial, data=data_maizer_subset) summary(model_null)

model1<-glmer(landuse~scale(agri)+(rdist|region_code3), family=binomial, data=data_maizer_subset) summary(model1)

model2<-glmer(landuse~scale(trans)+(rdist|region_code3), family=binomial, data=data_maizer_subset) summary(model2)

model3<-glmer(landuse~scale(elevation)+(rdist|region_code3), family=binomial, data=data_maizer_subset) summary(model3)

model4<-glmer(landuse~region_type+(rdist|region_code3), family=binomial, data=data_maizer_subset) summary(model4)

model5<-glmer(landuse~PA+(rdist|region_code3), family=binomial, data=data_maizer_subset) summary(model5)

###two variables###

model6<-glmer(landuse~scale(agri)+scale(trans)+(rdist|region_code3), family=binomial, data=data_maizer_subset) summary(model6)

model7<-glmer(landuse~scale(agri)+scale(elevation)+(rdist|region_code3), family=binomial, data=data_maizer_subset) summary(model7)

model8<-glmer(landuse~scale(agri)+region_type+(rdist|region_code3), family=binomial, data=data_maizer_subset) summary(model8)

model9<-glmer(landuse~scale(trans)+region_type+(rdist|region_code3), family=binomial, data=data_maizer_subset) summary(model9)

model10<-glmer(landuse~scale(elevation)+region_type+(rdist|region_code3), family=binomial, data=data_maizer_subset) summary(model10)

model11<-glmer(landuse~scale(trans)+scale(elevation)+(rdist|region_code3), family=binomial, data=data_maizer_subset) summary(model11)

model12<-glmer(landuse~scale(agri)+PA+(rdist|region_code3), family=binomial, data=data_maizer_subset) summary(model12) model13<-glmer(landuse~scale(trans)+PA+(rdist|region_code3), family=binomial, data=data_maizer_subset) summary(model13)

model14<-glmer(landuse~scale(elevation)+PA+(rdist|region_code3), family=binomial, data=data_maizer_subset) summary(model14)

model15<-glmer(landuse~region_type+PA+(rdist|region_code3), family=binomial, data=data_maizer_subset) summary(model15)

###three variables###

model16<-glmer(landuse~scale(agri)+scale(trans)+region_type+(rdist|region_code3), family=binomial, data=data_maizer_subset) summary(model16)

model17<-glmer(landuse~scale(agri)+scale(elevation)+region_type+(rdist|region_code3), family=binomial, data=data_maizer_subset) summary(model17)

model18<-glmer(landuse~scale(agri)+scale(trans)+scale(elevation)+(rdist|region_code3), family=binomial, data=data_maizer_subset) summary(model18)

model19<-glmer(landuse~scale(trans)+scale(elevation)+region_type+(rdist|region_code3), family=binomial, data=data_maizer_subset) summary(model19) model20<-glmer(landuse~scale(agri)+scale(trans)+PA+(rdist|region_code3), family=binomial, data=data_maizer_subset) summary(model20)

model21<-glmer(landuse~scale(agri)+scale(elevation)+PA+(rdist|region_code3), family=binomial, data=data_maizer_subset) summary(model21)

model22<-glmer(landuse~scale(agri)+region_type+PA+(rdist|region_code3), family=binomial, data=data_maizer_subset) summary(model22)

model23<-glmer(landuse~scale(trans)+scale(elevation)+PA+(rdist|region_code3), family=binomial, data=data_maizer_subset) summary(model23)

model24<-glmer(landuse~scale(trans)+region_type+PA+(rdist|region_code3), family=binomial, data=data_maizer_subset) summary(model24)

model25<-glmer(landuse~scale(elevation)+region_type+PA+(rdist|region_code3), family=binomial, data=data_maizer_subset) summary(model25)

###four variables###

model26<-

glmer(landuse~scale(agri)+scale(trans)+scale(elevation)+region_type+(rdist|region_code3), family=binomial, data=data_maizer_subset) summary(model26) model27<-glmer(landuse~scale(agri)+scale(trans)+scale(elevation)+PA+(rdist|region_code3), family=binomial, data=data_maizer_subset) summary(model27)

model28<-glmer(landuse~scale(agri)+scale(trans)+PA+region_type+(rdist|region_code3), family=binomial, data=data_maizer_subset) summary(model28)

model29<-glmer(landuse~scale(agri)+PA+scale(elevation)+region_type+(rdist|region_code3), family=binomial, data=data_maizer_subset) summary(model29)

model30<-glmer(landuse~PA+scale(trans)+scale(elevation)+region_type+(rdist|region_code3), family=binomial, data=data_maizer_subset) summary(model30)

###five variables###

model31<-glmer(landuse ~ scale(agri)+scale(trans)+scale(elevation)

+region_type+PA+(rdist|region_code3), family=binomial, data=data_maizer_subset)

###one interaction###

model32<-

glmer(landuse~scale(agri)+scale(trans)+scale(elevation)+region_type+scale(agri):region_type+(r dist|region_code3), family=binomial, data=data_maizer_subset) summary(model32)

model33<-

glmer(landuse~scale(agri)+scale(trans)+scale(elevation)+region_type+scale(trans):region_type+

(rdist|region_code3), family=binomial, data=data_maizer_subset) summary(model33)

model34<-

glmer(landuse~scale(agri)+scale(trans)+scale(elevation)+region_type+scale(elevation):region_ty pe+(rdist|region_code3), family=binomial, data=data_maizer_subset) summary(model34)

model35<-

glmer(landuse~scale(agri)+scale(trans)+scale(elevation)+PA+scale(agri):PA+(rdist|region_code

3), family=binomial, data=data_maizer_subset) summary(model35)

model36<-

glmer(landuse~scale(agri)+scale(trans)+region_type+PA+scale(agri):PA+(rdist|region_code3), family=binomial, data=data_maizer_subset) summary(model36)

model37<-

glmer(landuse~scale(agri)+scale(elevation)+region_type+PA+scale(agri):PA+(rdist|region_code

3), family=binomial, data=data_maizer_subset) summary(model37)

model38<-

glmer(landuse~scale(agri)+scale(trans)+scale(elevation)+PA+scale(trans):PA+(rdist|region_code

3), family=binomial, data=data_maizer_subset) summary(model38)

model39<-

glmer(landuse~scale(agri)+scale(trans)+region_type+PA+scale(trans):PA+(rdist|region_code3), family=binomial, data=data_maizer_subset) summary(model39)

model40<-

glmer(landuse~scale(trans)+scale(elevation)+region_type+PA+scale(trans):PA+(rdist|region_co de3), family=binomial, data=data_maizer_subset) summary(model40)

model41<-

glmer(landuse~scale(agri)+scale(trans)+scale(elevation)+PA+scale(elevation):PA+(rdist|region_ code3), family=binomial, data=data_maizer_subset) summary(model41)

model42<-

glmer(landuse~scale(agri)+scale(elevation)+region_type+PA+scale(elevation):PA+(rdist|region_ code3), family=binomial, data=data_maizer_subset) summary(model42)

model43<-

glmer(landuse~scale(trans)+scale(elevation)+region_type+PA+scale(elevation):PA+(rdist|region

_code3), family=binomial, data=data_maizer_subset) summary(model43)

model44<-

glmer(landuse~scale(agri)+scale(trans)+region_type+PA+region_type:PA+(rdist|region_code3), family=binomial, data=data_maizer_subset) summary(model44)

model45<-

glmer(landuse~scale(agri)+scale(elevation)+region_type+PA+region_type:PA+(rdist|region_cod e3), family=binomial, data=data_maizer_subset) summary(model45)

model46<-

glmer(landuse~scale(trans)+scale(elevation)+region_type+PA+region_type:PA+(rdist|region_co de3), family=binomial, data=data_maizer_subset) summary(model46)

###two interactions###

## with region

model47<-

glmer(landuse~scale(agri)+scale(trans)+scale(elevation)+region_type+scale(agri):region_type+s cale(trans):region_type+(rdist|region_code3), family=binomial, data=data_maizer_subset) summary(model47)

model48<-

glmer(landuse~scale(agri)+scale(trans)+scale(elevation)+region_type+scale(agri):region_type+s cale(elevation):region_type+(rdist|region_code3), family=binomial, data=data_maizer_subset) summary(model48)

model49<-

glmer(landuse~scale(agri)+scale(trans)+scale(elevation)+region_type+scale(trans):region_type+ scale(elevation):region_type+(rdist|region_code3), family=binomial, data=data_maizer_subset) summary(model49)

##with PA model50<-

glmer(landuse~scale(agri)+scale(trans)+scale(elevation)+PA+scale(agri):PA+scale(trans):PA+(r dist|region_code3), family=binomial, data=data_maizer_subset)

summary(model50)

model51<-

glmer(landuse~scale(agri)+scale(trans)+region_type+PA+scale(agri):PA+scale(trans):PA+(rdist| region_code3), family=binomial, data=data_maizer_subset) summary(model51)

model52<-

glmer(landuse~scale(agri)+scale(trans)+scale(elevation)+PA+scale(agri):PA+scale(elevation):P

A+(rdist|region_code3), family=binomial, data=data_maizer_subset) summary(model52)

model53<-

glmer(landuse~scale(agri)+scale(elevation)+region_type+PA+scale(agri):PA+scale(elevation):P

A+(rdist|region_code3), family=binomial, data=data_maizer_subset) summary(model53)

model54<-

glmer(landuse~scale(agri)+scale(trans)+region_type+PA+scale(agri):PA+region_type:PA+(rdist| region_code3), family=binomial, data=data_maizer_subset) summary(model54)

model55<-

glmer(landuse~scale(agri)+scale(elevation)+region_type+PA+scale(agri):PA+region_type:PA+( rdist|region_code3), family=binomial, data=data_maizer_subset) summary(model55)

model56<-

glmer(landuse~scale(agri)+scale(trans)+scale(elevation)+PA+scale(trans):PA+scale(elevation):P

A+(rdist|region_code3), family=binomial, data=data_maizer_subset) summary(model56)

model57<glmer(landuse~scale(trans)+scale(elevation)+region_type+PA+scale(trans):PA+scale(elevation): PA+(rdist|region_code3), family=binomial, data=data_maizer_subset) summary(model57)

model58<-

glmer(landuse~scale(agri)+scale(trans)+region_type+PA+scale(trans):PA+region_type:PA+(rdis t|region_code3), family=binomial, data=data_maizer_subset) summary(model58)

model59<-

glmer(landuse~scale(trans)+scale(elevation)+region_type+PA+scale(trans):PA+region_type:PA

+(rdist|region_code3), family=binomial, data=data_maizer_subset) summary(model59)

##combine two sets model60<-

glmer(landuse~scale(agri)+scale(trans)+region_type+PA+scale(agri):region_type+scale(agri):P

A+(rdist|region_code3), family=binomial, data=data_maizer_subset) summary(model60)

model61<-

glmer(landuse~scale(agri)+scale(elevation)+region_type+PA+scale(agri):region_type+scale(agri

):PA+(rdist|region_code3), family=binomial, data=data_maizer_subset) summary(model61)

model62<-

glmer(landuse~scale(agri)+scale(trans)+region_type+PA+scale(agri):region_type+scale(trans):P

A+(rdist|region_code3), family=binomial, data=data_maizer_subset) summary(model62)

model63<-

glmer(landuse~scale(agri)+scale(elevation)+region_type+PA+scale(agri):region_type+scale(elev ation):PA+(rdist|region_code3), family=binomial, data=data_maizer_subset) summary(model63)

model64<-

glmer(landuse~scale(agri)+scale(trans)+region_type+PA+scale(agri):region_type+region_type:P

A+(rdist|region_code3), family=binomial, data=data_maizer_subset) summary(model64)

model65<-

glmer(landuse~scale(agri)+scale(elevation)+region_type+PA+scale(agri):region_type+region_ty pe:PA+(rdist|region_code3), family=binomial, data=data_maizer_subset) summary(model65)

model66<-

glmer(landuse~scale(agri)+scale(trans)+region_type+PA+scale(trans):region_type+scale(agri):P

A+(rdist|region_code3), family=binomial, data=data_maizer_subset) summary(model66)

model67<glmer(landuse~scale(agri)+scale(trans)+region_type+PA+scale(trans):region_type+scale(trans): PA+(rdist|region_code3), family=binomial, data=data_maizer_subset) summary(model67)

model68<-

glmer(landuse~scale(elevation)+scale(trans)+region_type+PA+scale(trans):region_type+scale(tr ans):PA+(rdist|region_code3), family=binomial, data=data_maizer_subset) summary(model68)

model69<-

glmer(landuse~scale(trans)+scale(elevation)+region_type+PA+scale(trans):region_type+scale(el evation):PA+(rdist|region_code3), family=binomial, data=data_maizer_subset) summary(model69)

model70<glmer(landuse~scale(agri)+scale(trans)+region_type+PA+scale(trans):region_type+region_type: PA+(rdist|region_code3), family=binomial, data=data_maizer_subset) summary(model70)

model71<-

glmer(landuse~scale(trans)+scale(elevation)+region_type+PA+scale(trans):region_type+region_ type:PA+(rdist|region_code3), family=binomial, data=data_maizer_subset) summary(model71)

model72<-

glmer(landuse~scale(agri)+scale(elevation)+region_type+PA+scale(elevation):region_type+scal e(agri):PA+(rdist|region_code3), family=binomial, data=data_maizer_subset) summary(model72)

model73<-

glmer(landuse~scale(trans)+scale(elevation)+region_type+PA+scale(elevation):region_type+sca le(trans):PA+(rdist|region_code3), family=binomial, data=data_maizer_subset) summary(model73)

model74<-

glmer(landuse~scale(agri)+scale(elevation)+region_type+PA+scale(elevation):region_type+scal e(elevation):PA+(rdist|region_code3), family=binomial, data=data_maizer_subset) summary(model74)

model75<-

glmer(landuse~scale(trans)+scale(elevation)+region_type+PA+scale(elevation):region_type+sca le(elevation):PA+(rdist|region_code3), family=binomial, data=data_maizer_subset) summary(model75)

model76<-

glmer(landuse~scale(agri)+scale(elevation)+region_type+PA+scale(elevation):region_type+regi on_type:PA+(rdist|region_code3), family=binomial, data=data_maizer_subset) summary(model76)

model77<-

glmer(landuse~scale(trans)+scale(elevation)+region_type+PA+scale(elevation):region_type+reg ion_type:PA+(rdist|region_code3), family=binomial, data=data_maizer_subset) summary(model77)

###three interactions###

model78<-

glmer(landuse~scale(agri)+scale(trans)+scale(elevation)+region_type+scale(agri):region_type+s cale(elevation):region_type+scale(trans):region_type+(rdist|region_code3), family=binomial, data=data_maizer_subset) summary(model78)

model79<-

glmer(landuse~scale(agri)+scale(trans)+region_type+PA+scale(agri):region_type+scale(agri):P A+scale(trans):region_type+(rdist|region_code3), family=binomial, data=data_maizer_subset) summary(model79)

model80<-

glmer(landuse~scale(agri)+scale(trans)+region_type+PA+scale(agri):region_type+scale(trans):P A+scale(trans):region_type+(rdist|region_code3), family=binomial, data=data_maizer_subset) summary(model80)

model81<-

glmer(landuse~scale(agri)+scale(trans)+region_type+PA+scale(agri):region_type+region_type:P A+scale(trans):region_type+(rdist|region_code3), family=binomial, data=data_maizer_subset) summary(model81)

model82<-

glmer(landuse~scale(agri)+scale(elevation)+region_type+PA+scale(agri):region_type+scale(agri

):PA+scale(elevation):region_type+(rdist|region_code3), family=binomial, data=data_maizer_subset) summary(model82)

model83<-

glmer(landuse~scale(agri)+scale(elevation)+region_type+PA+scale(agri):region_type+scale(elev ation):PA+scale(elevation):region_type+(rdist|region_code3), family=binomial, data=data_maizer_subset) summary(model83)

model84<-

glmer(landuse~scale(agri)+scale(elevation)+region_type+PA+scale(agri):region_type+region_ty pe:PA+scale(elevation):region_type+(rdist|region_code3), family=binomial, data=data_maizer_subset) summary(model84)

model85<-

glmer(landuse~scale(trans)+scale(elevation)+region_type+PA+scale(trans):region_type+scale(tr ans):PA+scale(elevation):region_type+(rdist|region_code3), family=binomial, data=data_maizer_subset) summary(model85)

model86<-

glmer(landuse~scale(trans)+scale(elevation)+region_type+PA+scale(trans):region_type+scale(el evation):PA+scale(elevation):region_type+(rdist|region_code3), family=binomial, data=data_maizer_subset) summary(model86)

model87<-

glmer(landuse~scale(trans)+scale(elevation)+region_type+PA+scale(trans):region_type+region_ type:PA+scale(elevation):region_type+(rdist|region_code3), family=binomial, data=data_maizer_subset)

summary(model87)

###four interactions### model88<-

glmer(landuse~scale(agri)+scale(trans)+region_type+PA+scale(agri):region_type+scale(trans):re gion_type+scale(agri):PA+scale(trans):PA+(rdist|region_code3), family=binomial, data=data_maizer_subset) summary(model88)

model89<-

glmer(landuse~scale(agri)+scale(trans)+region_type+PA+scale(agri):region_type+scale(trans):re gion_type+scale(agri):PA+region_type:PA+(rdist|region_code3), family=binomial, data=data_maizer_subset) summary(model89)

model90<-

glmer(landuse~scale(agri)+scale(trans)+region_type+PA+scale(agri):region_type+scale(trans):re gion_type+scale(trans):PA+region_type:PA+(rdist|region_code3), family=binomial, data=data_maizer_subset) summary(model90)

model91<-

glmer(landuse~scale(agri)+scale(elevation)+region_type+PA+scale(agri):region_type+scale(elev ation):region_type+scale(agri):PA+scale(elevation):PA+(rdist|region_code3), family=binomial, data=data_maizer_subset) summary(model91)

model92<-

glmer(landuse~scale(agri)+scale(elevation)+region_type+PA+scale(agri):region_type+scale(elev ation):region_type+scale(agri):PA+region_type:PA+(rdist|region_code3), family=binomial, data=data_maizer_subset) summary(model92)

model93<-

glmer(landuse~scale(agri)+scale(elevation)+region_type+PA+scale(agri):region_type+scale(elev ation):region_type+region_type:PA+scale(elevation):PA+(rdist|region_code3), family=binomial, data=data_maizer_subset) summary(model93)

model94<-

glmer(landuse~scale(trans)+scale(elevation)+region_type+PA+scale(trans):region_type+scale(el evation):region_type+scale(trans):PA+scale(elevation):PA+(rdist|region_code3), family=binomial, data=data_maizer_subset) summary(model94)

model95<-

glmer(landuse~scale(trans)+scale(elevation)+region_type+PA+scale(trans):region_type+scale(el evation):region_type+scale(trans):PA+region_type:PA+(rdist|region_code3), family=binomial, data=data_maizer_subset) summary(model95)

model96<-

glmer(landuse~scale(trans)+scale(elevation)+region_type+PA+scale(trans):region_type+scale(el evation):region_type+region_type:PA+scale(elevation):PA+(rdist|region_code3), family=binomial, data=data_maizer_subset) summary(model96)

###try five interactions model97<-

glmer(landuse~scale(agri)+scale(trans)+region_type+PA+scale(agri):region_type+scale(trans):re gion_type+scale(agri):PA+scale(trans):PA+region_type:PA+(rdist|region_code3), family=binomial, data=data_maizer_subset) summary(model97)

model98<-

glmer(landuse~scale(agri)+scale(elevation)+region_type+PA+scale(agri):region_type+scale(elev ation):region_type+scale(agri):PA+scale(elevation):PA+region_type:PA+(rdist|region_code3), family=binomial, data=data_maizer_subset) summary(model98)

model99<-

glmer(landuse~scale(trans)+scale(elevation)+region_type+PA+scale(trans):region_type+scale(el evation):region_type+scale(trans):PA+scale(elevation):PA+region_type:PA+(rdist|region_code3

), family=binomial, data=data_maizer_subset) summary(model99)

##select the best model models_sel_maizer<- model.sel(model_null,model1,model2,model3,model4,model5,model6, model8, model9, model10, model11,model12, model13, model14,

model15,model16,model17,model19,model20,model22,model24,model27,model28,model36, model43,model38,rank="AIC") sink(file="models_sel_maizer.txt")

models_sel_maizer

sink()

##dignostic plots library(arm) binnedplot(predict(model38, type="response"), resid(model38, type="response"))

library(effects) plot(allEffects(model38)) allEffects(model38)

sink(file="alleffects(model38).txt") allEffects(model38) sink()

sink("summary(model38).txt") summary(model38)

sink()

####################prediction with actual yield ###############################

#1. Model baseline scenario, just put the actual yield in

pred_maizer <- predict(model38, data_maizer_subset, re.form=NULL, type="response") head(pred_maizer)

write.csv(pred_maizer, "pred_maizer.csv")

##########################prediction with potential yield ###################### ##get potential yield data

data_all<-read.csv("Appendix_S4_Supplementary_Data.csv",header=T)

data_maize<-data_all[c(1:23313),]

data_maize_subset<-data_maize[data_maize$land_use!=2,]

data_maize_subset$land_use <- as.factor(data_maize_subset$land_use) data_maize_subset$region_code1 <- as.factor(data_maize_subset$region_code1) data_maize_subset$region_code2 <- as.factor(data_maize_subset$region_code2) data_maize_subset$region_code3 <- as.factor(data_maize_subset$region_code3) data_maize_subset$resid<-as.factor(data_maize_subset$resid) data_maize_subset$PA<- as.factor(data_maize_subset$PA)

colnames(data_maize_subset) <- c("landuse", "agri", "trans", "elevation",

"region_type","region_code1","region_code2","region_code3","rdist", "resid","PA","ID") head(data_maize_subset)

#2. Scenario: actual yield being replaced by potential yield data

pred_maize <- predict(model38, data_maize_subset, re.form=NULL, type="response") head(pred_maize)

write.csv(pred_maize, "pred_maize.csv")

#3. Scenario A: agr increase 50%

maize_y <- cbind(data_maize_subset$landuse, (data_maize_subset$agri)*1.5 , data_maize_subset[,3:12])

colnames(maize_y) <- c("landuse", "agri", "trans","elevation","region_type","region_code1","region_code2","region_code3","rdist","resid","PA","ID")

summary(maize_y)

pred_maize<-predict(model38, maize_y, re.form=NULL, type="response"))

write.csv(pred_maize, "pred_maize_agri.csv")

#4. Scenario B: transport costs increase by 35%

maize_trans_y <- cbind(data_maize_subset[,1:2], data_maize_subset[,3]*1.35, data_maize_subset[,4:12]) colnames(maize_trans_y) <- c("landuse", "agri",

"trans","elevation","region_type","region_code1","region_code2","region_code3","rdist","resid",

"PA","ID") summary(maize_trans_y)

pred_trans_maize<-predict(model38, maize_trans_y, re.form=NULL, type="response")

write.csv(pred_trans_maize, "pred_maize_trans.csv")

#5. Scenario C: political stability has total conversion

region_maize <- data_maize_subset

summary(region_maize) region_maize[,5] <- "Division"

region_maize[,5] <- as.factor(region_maize[,5])

pred_region_maize <- predict(model38, region_maize, re.form=NULL, type="response") head(pred_region_maize)

write.csv(pred_region_maize, "pred_maize_stability.csv")

#6. Scenario D: worst case. agri increase 50%, and stability has total conversion maize_worstcase<-maize_y

maize_worstcase[,5]<-"Division"

pred_worstcase_maize<-predict(model38, maize_worstcase, re.form=NULL, type="response")

write.csv(pred_worstcase_maize, "pred_maize_worstcase.csv")

rm(list=ls())

##############################################################################

###################model land use with characteristics of rice ########################

setwd("D:\\Myanmar project\\Resubmission 2\\Code and data (submit)")

data_all_raman<-read.csv("Appendix_S3_Supplementary_Data.csv",header=T)

data_ricer<-data_all_raman[c(23314:46626),]

data_ricer_subset<-data_ricer[data_ricer$land_use!=2,]

#convert to factors data_ricer_subset$land_use <- as.factor(data_ricer_subset$land_use) data_ricer_subset$region_code1 <- as.factor(data_ricer_subset$region_code1) data_ricer_subset$region_code2 <- as.factor(data_ricer_subset$region_code2) data_ricer_subset$region_code3 <- as.factor(data_ricer_subset$region_code3) data_ricer_subset$resid<-as.factor(data_ricer_subset$resid) data_ricer_subset$PA<- as.factor(data_ricer_subset$PA) colnames(data_ricer_subset) <- c("landuse", "agri", "trans", "elevation",

"region_type","region_code1","region_code2","region_code3","rdist", "resid","PA","ID") head(data_ricer_subset)

library(lme4)

##check collinearity

library(car)

model_vif <- glm(landuse ~ scale(agri)+scale(trans)+scale(elevation) +region_type+PA, family=binomial, data=data_ricer_subset) summary(model_vif)

vif(model_vif)

###try to fix the random effects first

model_r1 <- glmer(landuse ~ scale(agri)+scale(trans)+scale(elevation) +region_type+PA+ (1|region_code1), family=binomial, data=data_ricer_subset) summary(model_r1)

model_r2 <- glmer(landuse ~ scale(agri)+scale(trans) +scale(elevation)+region_type+PA+

(1|region_code2), family=binomial, data=data_ricer_subset) summary(model_r2)

model_r3 <- glmer(landuse ~ scale(agri)+scale(trans)+scale(elevation) +region_type+PA+

(1|region_code3), family=binomial, data=data_ricer_subset) summary(model_r3)

AIC(model_r1, model_r2, model_r3)

#choose region_code3 with smallest AIC

model_r4 <- glmer(landuse ~ scale(agri)+scale(trans)+scale(elevation) +region_type+PA+

(rdist|region_code3), family=binomial, data=data_ricer_subset) summary(model_r4)

AIC(model_r3,model_r4)

##choose model_r4 with samllest AIC

###one variable###

model_null<-glmer(landuse~1+(rdist|region_code3), family=binomial, data=data_ricer_subset) summary(model_null)

model1<-glmer(landuse~scale(agri)+(rdist|region_code3), family=binomial, data=data_ricer_subset) summary(model1)

model2<-glmer(landuse~scale(trans)+(rdist|region_code3), family=binomial, data=data_ricer_subset) summary(model2)

model3<-glmer(landuse~scale(elevation)+(rdist|region_code3), family=binomial, data=data_ricer_subset) summary(model3)

model4<-glmer(landuse~region_type+(rdist|region_code3), family=binomial, data=data_ricer_subset) summary(model4)

model5<-glmer(landuse~PA+(rdist|region_code3), family=binomial, data=data_ricer_subset) summary(model5)

###two variables###

model6<-glmer(landuse~scale(agri)+scale(trans)+(rdist|region_code3), family=binomial, data=data_ricer_subset) summary(model6) model7<-glmer(landuse~scale(agri)+scale(elevation)+(rdist|region_code3), family=binomial, data=data_ricer_subset) summary(model7)

model8<-glmer(landuse~scale(agri)+region_type+(rdist|region_code3), family=binomial, data=data_ricer_subset) summary(model8)

model9<-glmer(landuse~scale(trans)+region_type+(rdist|region_code3), family=binomial, data=data_ricer_subset) summary(model9)

model10<-glmer(landuse~scale(elevation)+region_type+(rdist|region_code3), family=binomial, data=data_ricer_subset) summary(model10)

model11<-glmer(landuse~scale(trans)+scale(elevation)+(rdist|region_code3), family=binomial, data=data_ricer_subset) summary(model11)

model12<-glmer(landuse~scale(agri)+PA+(rdist|region_code3), family=binomial, data=data_ricer_subset) summary(model12)

model13<-glmer(landuse~scale(trans)+PA+(rdist|region_code3), family=binomial, data=data_ricer_subset) summary(model13)

model14<-glmer(landuse~scale(elevation)+PA+(rdist|region_code3), family=binomial, data=data_ricer_subset)

summary(model14)

model15<-glmer(landuse~region_type+PA+(rdist|region_code3), family=binomial, data=data_ricer_subset) summary(model15)

###three variables###

model16<-glmer(landuse~scale(agri)+scale(trans)+region_type+(rdist|region_code3), family=binomial, data=data_ricer_subset) summary(model16)

model17<-glmer(landuse~scale(agri)+scale(elevation)+region_type+(rdist|region_code3), family=binomial, data=data_ricer_subset) summary(model17)

model18<-glmer(landuse~scale(agri)+scale(trans)+scale(elevation)+(rdist|region_code3), family=binomial, data=data_ricer_subset) summary(model18)

model19<-glmer(landuse~scale(trans)+scale(elevation)+region_type+(rdist|region_code3), family=binomial, data=data_ricer_subset) summary(model19)

model20<-glmer(landuse~scale(agri)+scale(trans)+PA+(rdist|region_code3), family=binomial, data=data_ricer_subset) summary(model20) model21<-glmer(landuse~scale(agri)+scale(elevation)+PA+(rdist|region_code3), family=binomial, data=data_ricer_subset) summary(model21)

model22<-glmer(landuse~scale(agri)+region_type+PA+(rdist|region_code3), family=binomial, data=data_ricer_subset) summary(model22)

model23<-glmer(landuse~scale(trans)+scale(elevation)+PA+(rdist|region_code3), family=binomial, data=data_ricer_subset) summary(model23)

model24<-glmer(landuse~scale(trans)+region_type+PA+(rdist|region_code3), family=binomial, data=data_ricer_subset) summary(model24)

model25<-glmer(landuse~scale(elevation)+region_type+PA+(rdist|region_code3), family=binomial, data=data_ricer_subset) summary(model25)

###four variables###

model26<-

glmer(landuse~scale(agri)+scale(trans)+scale(elevation)+region_type+(rdist|region_code3), family=binomial, data=data_ricer_subset) summary(model26)

model27<-glmer(landuse~scale(agri)+scale(trans)+scale(elevation)+PA+(rdist|region_code3), family=binomial, data=data_ricer_subset) summary(model27) model28<-glmer(landuse~scale(agri)+scale(trans)+PA+region_type+(rdist|region_code3), family=binomial, data=data_ricer_subset) summary(model28)

model29<-glmer(landuse~scale(agri)+PA+scale(elevation)+region_type+(rdist|region_code3), family=binomial, data=data_ricer_subset) summary(model29)

model30<-glmer(landuse~PA+scale(trans)+scale(elevation)+region_type+(rdist|region_code3), family=binomial, data=data_ricer_subset) summary(model30)

###five variables###

model31<-glmer(landuse ~ scale(agri)+scale(trans)+scale(elevation)

+region_type+PA+(rdist|region_code3), family=binomial, data=data_ricer_subset)

###one interaction###

model32<-

glmer(landuse~scale(agri)+scale(trans)+scale(elevation)+region_type+scale(agri):region_type+(r dist|region_code3), family=binomial, data=data_ricer_subset) summary(model32)

model33<-

glmer(landuse~scale(agri)+scale(trans)+scale(elevation)+region_type+scale(trans):region_type+

(rdist|region_code3), family=binomial, data=data_ricer_subset) summary(model33)

model34<-

glmer(landuse~scale(agri)+scale(trans)+scale(elevation)+region_type+scale(elevation):region_ty pe+(rdist|region_code3), family=binomial, data=data_ricer_subset)

summary(model34)

model35<-

glmer(landuse~scale(agri)+scale(trans)+scale(elevation)+PA+scale(agri):PA+(rdist|region_code

3), family=binomial, data=data_ricer_subset) summary(model35)

model36<-

glmer(landuse~scale(agri)+scale(trans)+region_type+PA+scale(agri):PA+(rdist|region_code3), family=binomial, data=data_ricer_subset) summary(model36)

model37<-

glmer(landuse~scale(agri)+scale(elevation)+region_type+PA+scale(agri):PA+(rdist|region_code

3), family=binomial, data=data_ricer_subset) summary(model37)

model38<-

glmer(landuse~scale(agri)+scale(trans)+scale(elevation)+PA+scale(trans):PA+(rdist|region_code

3), family=binomial, data=data_ricer_subset) summary(model38)

model39<-

glmer(landuse~scale(agri)+scale(trans)+region_type+PA+scale(trans):PA+(rdist|region_code3), family=binomial, data=data_ricer_subset) summary(model39)

model40<-

glmer(landuse~scale(trans)+scale(elevation)+region_type+PA+scale(trans):PA+(rdist|region_co de3), family=binomial, data=data_ricer_subset) summary(model40)

model41<-

glmer(landuse~scale(agri)+scale(trans)+scale(elevation)+PA+scale(elevation):PA+(rdist|region_ code3), family=binomial, data=data_ricer_subset) summary(model41)

model42<-

glmer(landuse~scale(agri)+scale(elevation)+region_type+PA+scale(elevation):PA+(rdist|region_ code3), family=binomial, data=data_ricer_subset) summary(model42)

model43<-

glmer(landuse~scale(trans)+scale(elevation)+region_type+PA+scale(elevation):PA+(rdist|region

_code3), family=binomial, data=data_ricer_subset) summary(model43)

model44<-

glmer(landuse~scale(agri)+scale(trans)+region_type+PA+region_type:PA+(rdist|region_code3), family=binomial, data=data_ricer_subset) summary(model44)

model45<-

glmer(landuse~scale(agri)+scale(elevation)+region_type+PA+region_type:PA+(rdist|region_cod e3), family=binomial, data=data_ricer_subset) summary(model45)

model46<-

glmer(landuse~scale(trans)+scale(elevation)+region_type+PA+region_type:PA+(rdist|region_co de3), family=binomial, data=data_ricer_subset) summary(model46)

###two interactions###

## with region

model47<-

glmer(landuse~scale(agri)+scale(trans)+scale(elevation)+region_type+scale(agri):region_type+s cale(trans):region_type+(rdist|region_code3), family=binomial, data=data_ricer_subset) summary(model47)

model48<-

glmer(landuse~scale(agri)+scale(trans)+scale(elevation)+region_type+scale(agri):region_type+s cale(elevation):region_type+(rdist|region_code3), family=binomial, data=data_ricer_subset) summary(model48)

model49<-

glmer(landuse~scale(agri)+scale(trans)+scale(elevation)+region_type+scale(trans):region_type+ scale(elevation):region_type+(rdist|region_code3), family=binomial, data=data_ricer_subset) summary(model49)

##with PA model50<-

glmer(landuse~scale(agri)+scale(trans)+scale(elevation)+PA+scale(agri):PA+scale(trans):PA+(r dist|region_code3), family=binomial, data=data_ricer_subset) summary(model50)

model51<-

glmer(landuse~scale(agri)+scale(trans)+region_type+PA+scale(agri):PA+scale(trans):PA+(rdist| region_code3), family=binomial, data=data_ricer_subset) summary(model51)

model52<-

glmer(landuse~scale(agri)+scale(trans)+scale(elevation)+PA+scale(agri):PA+scale(elevation):P

A+(rdist|region_code3), family=binomial, data=data_ricer_subset)

summary(model52)

model53<-

glmer(landuse~scale(agri)+scale(elevation)+region_type+PA+scale(agri):PA+scale(elevation):P

A+(rdist|region_code3), family=binomial, data=data_ricer_subset) summary(model53)

model54<-

glmer(landuse~scale(agri)+scale(trans)+region_type+PA+scale(agri):PA+region_type:PA+(rdist| region_code3), family=binomial, data=data_ricer_subset) summary(model54)

model55<-

glmer(landuse~scale(agri)+scale(elevation)+region_type+PA+scale(agri):PA+region_type:PA+( rdist|region_code3), family=binomial, data=data_ricer_subset) summary(model55)

model56<-

glmer(landuse~scale(agri)+scale(trans)+scale(elevation)+PA+scale(trans):PA+scale(elevation):P

A+(rdist|region_code3), family=binomial, data=data_ricer_subset) summary(model56)

model57<glmer(landuse~scale(trans)+scale(elevation)+region_type+PA+scale(trans):PA+scale(elevation): PA+(rdist|region_code3), family=binomial, data=data_ricer_subset) summary(model57)

model58<-

glmer(landuse~scale(agri)+scale(trans)+region_type+PA+scale(trans):PA+region_type:PA+(rdis t|region_code3), family=binomial, data=data_ricer_subset) summary(model58)

model59<-

glmer(landuse~scale(trans)+scale(elevation)+region_type+PA+scale(trans):PA+region_type:PA

+(rdist|region_code3), family=binomial, data=data_ricer_subset) summary(model59)

##combine two sets model60<-

glmer(landuse~scale(agri)+scale(trans)+region_type+PA+scale(agri):region_type+scale(agri):P

A+(rdist|region_code3), family=binomial, data=data_ricer_subset) summary(model60)

model61<-

glmer(landuse~scale(agri)+scale(elevation)+region_type+PA+scale(agri):region_type+scale(agri

):PA+(rdist|region_code3), family=binomial, data=data_ricer_subset) summary(model61)

model62<-

glmer(landuse~scale(agri)+scale(trans)+region_type+PA+scale(agri):region_type+scale(trans):P

A+(rdist|region_code3), family=binomial, data=data_ricer_subset) summary(model62)

model63<-

glmer(landuse~scale(agri)+scale(elevation)+region_type+PA+scale(agri):region_type+scale(elev ation):PA+(rdist|region_code3), family=binomial, data=data_ricer_subset) summary(model63)

model64<-

glmer(landuse~scale(agri)+scale(trans)+region_type+PA+scale(agri):region_type+region_type:P

A+(rdist|region_code3), family=binomial, data=data_ricer_subset) summary(model64)

model65<-

glmer(landuse~scale(agri)+scale(elevation)+region_type+PA+scale(agri):region_type+region_ty pe:PA+(rdist|region_code3), family=binomial, data=data_ricer_subset) summary(model65)

model66<-

glmer(landuse~scale(agri)+scale(trans)+region_type+PA+scale(trans):region_type+scale(agri):P

A+(rdist|region_code3), family=binomial, data=data_ricer_subset) summary(model66)

model67<glmer(landuse~scale(agri)+scale(trans)+region_type+PA+scale(trans):region_type+scale(trans): PA+(rdist|region_code3), family=binomial, data=data_ricer_subset) summary(model67)

model68<-

glmer(landuse~scale(elevation)+scale(trans)+region_type+PA+scale(trans):region_type+scale(tr ans):PA+(rdist|region_code3), family=binomial, data=data_ricer_subset) summary(model68)

model69<-

glmer(landuse~scale(trans)+scale(elevation)+region_type+PA+scale(trans):region_type+scale(el evation):PA+(rdist|region_code3), family=binomial, data=data_ricer_subset) summary(model69)

model70<glmer(landuse~scale(agri)+scale(trans)+region_type+PA+scale(trans):region_type+region_type: PA+(rdist|region_code3), family=binomial, data=data_ricer_subset) summary(model70)

model71<-

glmer(landuse~scale(trans)+scale(elevation)+region_type+PA+scale(trans):region_type+region_ type:PA+(rdist|region_code3), family=binomial, data=data_ricer_subset)

summary(model71)

model72<-

glmer(landuse~scale(agri)+scale(elevation)+region_type+PA+scale(elevation):region_type+scal e(agri):PA+(rdist|region_code3), family=binomial, data=data_ricer_subset) summary(model72)

model73<-

glmer(landuse~scale(trans)+scale(elevation)+region_type+PA+scale(elevation):region_type+sca le(trans):PA+(rdist|region_code3), family=binomial, data=data_ricer_subset) summary(model73)

model74<-

glmer(landuse~scale(agri)+scale(elevation)+region_type+PA+scale(elevation):region_type+scal e(elevation):PA+(rdist|region_code3), family=binomial, data=data_ricer_subset) summary(model74)

model75<-

glmer(landuse~scale(trans)+scale(elevation)+region_type+PA+scale(elevation):region_type+sca le(elevation):PA+(rdist|region_code3), family=binomial, data=data_ricer_subset) summary(model75)

model76<-

glmer(landuse~scale(agri)+scale(elevation)+region_type+PA+scale(elevation):region_type+regi on_type:PA+(rdist|region_code3), family=binomial, data=data_ricer_subset) summary(model76)

model77<-

glmer(landuse~scale(trans)+scale(elevation)+region_type+PA+scale(elevation):region_type+reg ion_type:PA+(rdist|region_code3), family=binomial, data=data_ricer_subset) summary(model77)

###three interactions###

model78<-

glmer(landuse~scale(agri)+scale(trans)+scale(elevation)+region_type+scale(agri):region_type+s cale(elevation):region_type+scale(trans):region_type+(rdist|region_code3), family=binomial, data=data_ricer_subset) summary(model78)

model79<-

glmer(landuse~scale(agri)+scale(trans)+region_type+PA+scale(agri):region_type+scale(agri):P A+scale(trans):region_type+(rdist|region_code3), family=binomial, data=data_ricer_subset) summary(model79)

model80<-

glmer(landuse~scale(agri)+scale(trans)+region_type+PA+scale(agri):region_type+scale(trans):P A+scale(trans):region_type+(rdist|region_code3), family=binomial, data=data_ricer_subset) summary(model80)

model81<-

glmer(landuse~scale(agri)+scale(trans)+region_type+PA+scale(agri):region_type+region_type:P A+scale(trans):region_type+(rdist|region_code3), family=binomial, data=data_ricer_subset) summary(model81)

model82<-

glmer(landuse~scale(agri)+scale(elevation)+region_type+PA+scale(agri):region_type+scale(agri

):PA+scale(elevation):region_type+(rdist|region_code3), family=binomial, data=data_ricer_subset) summary(model82)

model83<-

glmer(landuse~scale(agri)+scale(elevation)+region_type+PA+scale(agri):region_type+scale(elev ation):PA+scale(elevation):region_type+(rdist|region_code3), family=binomial, data=data_ricer_subset)

summary(model83)

model84<-

glmer(landuse~scale(agri)+scale(elevation)+region_type+PA+scale(agri):region_type+region_ty pe:PA+scale(elevation):region_type+(rdist|region_code3), family=binomial, data=data_ricer_subset) summary(model84)

model85<-

glmer(landuse~scale(trans)+scale(elevation)+region_type+PA+scale(trans):region_type+scale(tr ans):PA+scale(elevation):region_type+(rdist|region_code3), family=binomial, data=data_ricer_subset) summary(model85)

model86<-

glmer(landuse~scale(trans)+scale(elevation)+region_type+PA+scale(trans):region_type+scale(el evation):PA+scale(elevation):region_type+(rdist|region_code3), family=binomial, data=data_ricer_subset) summary(model86)

model87<-

glmer(landuse~scale(trans)+scale(elevation)+region_type+PA+scale(trans):region_type+region_ type:PA+scale(elevation):region_type+(rdist|region_code3), family=binomial, data=data_ricer_subset) summary(model87)

###four interactions### model88<-

glmer(landuse~scale(agri)+scale(trans)+region_type+PA+scale(agri):region_type+scale(trans):re gion_type+scale(agri):PA+scale(trans):PA+(rdist|region_code3), family=binomial, data=data_ricer_subset) summary(model88)

model89<-

glmer(landuse~scale(agri)+scale(trans)+region_type+PA+scale(agri):region_type+scale(trans):re gion_type+scale(agri):PA+region_type:PA+(rdist|region_code3), family=binomial, data=data_ricer_subset) summary(model89)

model90<-

glmer(landuse~scale(agri)+scale(trans)+region_type+PA+scale(agri):region_type+scale(trans):re gion_type+scale(trans):PA+region_type:PA+(rdist|region_code3), family=binomial, data=data_ricer_subset) summary(model90)

model91<-

glmer(landuse~scale(agri)+scale(elevation)+region_type+PA+scale(agri):region_type+scale(elev ation):region_type+scale(agri):PA+scale(elevation):PA+(rdist|region_code3), family=binomial, data=data_ricer_subset) summary(model91)

model92<-

glmer(landuse~scale(agri)+scale(elevation)+region_type+PA+scale(agri):region_type+scale(elev ation):region_type+scale(agri):PA+region_type:PA+(rdist|region_code3), family=binomial, data=data_ricer_subset) summary(model92)

model93<-

glmer(landuse~scale(agri)+scale(elevation)+region_type+PA+scale(agri):region_type+scale(elev ation):region_type+region_type:PA+scale(elevation):PA+(rdist|region_code3), family=binomial, data=data_ricer_subset) summary(model93)

model94<-

glmer(landuse~scale(trans)+scale(elevation)+region_type+PA+scale(trans):region_type+scale(el evation):region_type+scale(trans):PA+scale(elevation):PA+(rdist|region_code3), family=binomial, data=data_ricer_subset)

summary(model94)

model95<-

glmer(landuse~scale(trans)+scale(elevation)+region_type+PA+scale(trans):region_type+scale(el evation):region_type+scale(trans):PA+region_type:PA+(rdist|region_code3), family=binomial, data=data_ricer_subset) summary(model95)

model96<-

glmer(landuse~scale(trans)+scale(elevation)+region_type+PA+scale(trans):region_type+scale(el evation):region_type+region_type:PA+scale(elevation):PA+(rdist|region_code3), family=binomial, data=data_ricer_subset) summary(model96)

###try five interactions### model97<-

glmer(landuse~scale(agri)+scale(trans)+region_type+PA+scale(agri):region_type+scale(trans):re gion_type+scale(agri):PA+scale(trans):PA+region_type:PA+(rdist|region_code3), family=binomial, data=data_ricer_subset) summary(model97)

model98<-

glmer(landuse~scale(agri)+scale(elevation)+region_type+PA+scale(agri):region_type+scale(elev ation):region_type+scale(agri):PA+scale(elevation):PA+region_type:PA+(rdist|region_code3), family=binomial, data=data_ricer_subset) summary(model98)

model99<-

glmer(landuse~scale(trans)+scale(elevation)+region_type+PA+scale(trans):region_type+scale(el evation):region_type+scale(trans):PA+scale(elevation):PA+region_type:PA+(rdist|region_code3

), family=binomial, data=data_ricer_subset) summary(model99)

##select the best model library(MuMIn)

models_sel_ricer<-

model.sel(model_null,model1,model2,model3,model4,model5,model6,model7, model8, model9, model10, model11,model12, model13, model14,

model15,model16,model19,model20,model22,model24,model28,model30,model35,model37,mo del45,rank="AIC")

sink(file="models_sel_ricer.txt")

models_sel_ricer

sink()

##dignostic plots library(arm) binnedplot(predict(model35, type="response"), resid(model35, type="response"))

qqnorm(resid(model35)) qqnorm(ranef(model35))

library(effects) plot(allEffects(model35)) allEffects(model35)

sink(file="alleffects(model35).txt") allEffects(model35)

sink()

sink("summary(model35).txt") summary(model35)

sink()

####################prediction with actual yield ###############################

#1. Model baseline scenario, just put the actual yield in

pred_ricer <- predict(model35, data_ricer_subset, re.form=NULL, type="response") head(pred_ricer)

write.csv(pred_ricer, "pred_ricer.csv")

##########################prediction with potential yield ######################

##get potential yield data## data_all<-read.csv("Appendix_S4_Supplementary_Data.csv",header=T)

data_rice<-data_all[c(23314:46626),]

data_rice_subset<-data_rice[data_rice$land_use!=2,]

data_rice_subset$land_use <- as.factor(data_rice_subset$land_use) data_rice_subset$region_code1 <- as.factor(data_rice_subset$region_code1) data_rice_subset$region_code2 <- as.factor(data_rice_subset$region_code2) data_rice_subset$region_code3 <- as.factor(data_rice_subset$region_code3) data_rice_subset$resid<-as.factor(data_rice_subset$resid) data_rice_subset$PA<- as.factor(data_rice_subset$PA)

colnames(data_rice_subset) <- c("landuse", "agri", "trans", "elevation",

"region_type","region_code1","region_code2","region_code3","rdist", "resid","PA","ID") head(data_rice_subset)

#2. Scenario: actual yield being replaced by potential yield data

pred_rice <- predict(model35, data_rice_subset, re.form=NULL, type="response") head(pred_rice)

write.csv(pred_rice, "pred_rice.csv")

#3. Scenario A: agr increases 50%

rice_y <- cbind(data_rice_subset$landuse, (data_rice_subset$agri)*1.5 , data_rice_subset[,3:12]) colnames(rice_y) <- c("landuse", "agri",

"trans","elevation","region_type","region_code1","region_code2","region_code3","rdist","resid",

"PA","ID") summary(rice_y1)

pred_rice<-predict(model35, rice_y, re.form=NULL, type="response")

write.csv(pred_rice, "pred_rice_agri.csv")

#4. Scenario B: tranport cost increases 35%

rice_trans_y <- cbind(data_rice_subset[,1:2], data_rice_subset[,3]*1.35,data_rice_subset[,4:12]) colnames(rice_trans_y) <- c("landuse", "agri",

"trans","elevation","region_type","region_code1","region_code2","region_code3","rdist","resid",

"PA","ID") summary(rice_trans_y)

pred_trans_rice<-predict(model35, rice_trans_y, re.form=NULL, type="response")

write.csv(pred_trans_rice, "pred_rice_trans.csv")

#5. Scenario C: political stability has total conversion

region_rice <- data_rice_subset summary(region_rice) region_rice[,5] <- "Division" region_rice[,5] <- as.factor(region_rice[,5]) pred_region_rice <- predict(model35, region_rice, re.form=NULL, type="response") head(pred_region_rice)

write.csv(pred_region_rice, "pred_rice_stability.csv")

#6. Scenario D: worst case. agri increase 50%, stability total conversion rice_worstcase<-rice_y

rice_worstcase[,5]<-"Division"

pred_worstcase_rice<-predict(model35, rice_worstcase, re.form=NULL, type="response")

write.csv(pred_worstcase_rice, "pred_rice_worstcase.csv")

rm(list=ls())

##############################################################################

#################model land use with characteristics of chickpea####################### setwd("D:\\Myanmar project\\Resubmission 2\\Code and data (submit)")

data_all_raman<-read.csv("Appendix_S3_Supplementary_Data.csv",header=T)

data_chickpear<-data_all_raman[c(46627:69939),]

data_chickpear_subset<-data_chickpear[data_chickpear$land_use!=2,]

#convert to factors data_chickpear_subset$land_use <- as.factor(data_chickpear_subset$land_use) data_chickpear_subset$region_code1 <- as.factor(data_chickpear_subset$region_code1) data_chickpear_subset$region_code2 <- as.factor(data_chickpear_subset$region_code2) data_chickpear_subset$region_code3 <- as.factor(data_chickpear_subset$region_code3) data_chickpear_subset$resid<-as.factor(data_chickpear_subset$resid) data_chickpear_subset$PA<- as.factor(data_chickpear_subset$PA) colnames(data_chickpear_subset) <- c("landuse", "agri", "trans", "elevation",

"region_type","region_code1","region_code2","region_code3","rdist", "resid","PA","ID") head(data_chickpear_subset)

library(lme4)

##check collinearity

library(car)

model_vif <- glm(landuse ~ scale(agri)+scale(trans)+scale(elevation) +region_type+PA, family=binomial, data=data_chickpear_subset) summary(model_vif)

vif(model_vif)

###try to fix the random effects first

model_r1 <- glmer(landuse ~ scale(agri)+scale(trans)+scale(elevation) +region_type+PA+

(1|region_code1), family=binomial, data=data_chickpear_subset) summary(model_r1)

model_r2 <- glmer(landuse ~ scale(agri)+scale(trans) +scale(elevation)+region_type+PA+

(1|region_code2), family=binomial, data=data_chickpear_subset) summary(model_r2)

model_r3 <- glmer(landuse ~ scale(agri)+scale(trans)+scale(elevation) +region_type+PA+

(1|region_code3), family=binomial, data=data_chickpear_subset) summary(model_r3)

AIC(model_r1, model_r2, model_r3)

#choose region_code3 with smallest AIC

model_r4 <- glmer(landuse ~ scale(agri)+scale(trans)+scale(elevation) +region_type+PA+

(rdist|region_code3), family=binomial, data=data_chickpear_subset) summary(model_r4)

AIC(model_r3,model_r4)

##choose model_r4 with samllest AIC

###one variable### model_null<-glmer(landuse~1+(rdist|region_code3), family=binomial, data=data_chickpear_subset) summary(model_null)

model1<-glmer(landuse~scale(agri)+(rdist|region_code3), family=binomial, data=data_chickpear_subset) summary(model1)

model2<-glmer(landuse~scale(trans)+(rdist|region_code3), family=binomial, data=data_chickpear_subset) summary(model2)

model3<-glmer(landuse~scale(elevation)+(rdist|region_code3), family=binomial, data=data_chickpear_subset) summary(model3)

model4<-glmer(landuse~region_type+(rdist|region_code3), family=binomial, data=data_chickpear_subset) summary(model4)

model5<-glmer(landuse~PA+(rdist|region_code3), family=binomial, data=data_chickpear_subset) summary(model5)

###two variables###

model6<-glmer(landuse~scale(agri)+scale(trans)+(rdist|region_code3), family=binomial, data=data_chickpear_subset) summary(model6)

model7<-glmer(landuse~scale(agri)+scale(elevation)+(rdist|region_code3), family=binomial, data=data_chickpear_subset) summary(model7)

model8<-glmer(landuse~scale(agri)+region_type+(rdist|region_code3), family=binomial, data=data_chickpear_subset) summary(model8)

model9<-glmer(landuse~scale(trans)+region_type+(rdist|region_code3), family=binomial, data=data_chickpear_subset) summary(model9)

model10<-glmer(landuse~scale(elevation)+region_type+(rdist|region_code3), family=binomial, data=data_chickpear_subset) summary(model10)

model11<-glmer(landuse~scale(trans)+scale(elevation)+(rdist|region_code3), family=binomial, data=data_chickpear_subset) summary(model11)

model12<-glmer(landuse~scale(agri)+PA+(rdist|region_code3), family=binomial, data=data_chickpear_subset) summary(model12) model13<-glmer(landuse~scale(trans)+PA+(rdist|region_code3), family=binomial, data=data_chickpear_subset) summary(model13)

model14<-glmer(landuse~scale(elevation)+PA+(rdist|region_code3), family=binomial, data=data_chickpear_subset) summary(model14)

model15<-glmer(landuse~region_type+PA+(rdist|region_code3), family=binomial, data=data_chickpear_subset) summary(model15)

###three variables###

model16<-glmer(landuse~scale(agri)+scale(trans)+region_type+(rdist|region_code3), family=binomial, data=data_chickpear_subset) summary(model16)

model17<-glmer(landuse~scale(agri)+scale(elevation)+region_type+(rdist|region_code3), family=binomial, data=data_chickpear_subset) summary(model17)

model18<-glmer(landuse~scale(agri)+scale(trans)+scale(elevation)+(rdist|region_code3), family=binomial, data=data_chickpear_subset) summary(model18)

model19<-glmer(landuse~scale(trans)+scale(elevation)+region_type+(rdist|region_code3), family=binomial, data=data_chickpear_subset) summary(model19) model20<-glmer(landuse~scale(agri)+scale(trans)+PA+(rdist|region_code3), family=binomial, data=data_chickpear_subset) summary(model20)

model21<-glmer(landuse~scale(agri)+scale(elevation)+PA+(rdist|region_code3), family=binomial, data=data_chickpear_subset) summary(model21)

model22<-glmer(landuse~scale(agri)+region_type+PA+(rdist|region_code3), family=binomial, data=data_chickpear_subset) summary(model22)

model23<-glmer(landuse~scale(trans)+scale(elevation)+PA+(rdist|region_code3), family=binomial, data=data_chickpear_subset) summary(model23)

model24<-glmer(landuse~scale(trans)+region_type+PA+(rdist|region_code3), family=binomial, data=data_chickpear_subset) summary(model24)

model25<-glmer(landuse~scale(elevation)+region_type+PA+(rdist|region_code3), family=binomial, data=data_chickpear_subset) summary(model25)

###four variables###

model26<-

glmer(landuse~scale(agri)+scale(trans)+scale(elevation)+region_type+(rdist|region_code3), family=binomial, data=data_chickpear_subset) summary(model26) model27<-glmer(landuse~scale(agri)+scale(trans)+scale(elevation)+PA+(rdist|region_code3), family=binomial, data=data_chickpear_subset) summary(model27)

model28<-glmer(landuse~scale(agri)+scale(trans)+PA+region_type+(rdist|region_code3), family=binomial, data=data_chickpear_subset) summary(model28)

model29<-glmer(landuse~scale(agri)+PA+scale(elevation)+region_type+(rdist|region_code3), family=binomial, data=data_chickpear_subset) summary(model29)

model30<-glmer(landuse~PA+scale(trans)+scale(elevation)+region_type+(rdist|region_code3), family=binomial, data=data_chickpear_subset) summary(model30)

###five variables###

model31<-glmer(landuse ~ scale(agri)+scale(trans)+scale(elevation)

+region_type+PA+(rdist|region_code3), family=binomial, data=data_chickpear_subset)

###one interaction###

model32<-

glmer(landuse~scale(agri)+scale(trans)+scale(elevation)+region_type+scale(agri):region_type+(r dist|region_code3), family=binomial, data=data_chickpear_subset) summary(model32)

model33<-

glmer(landuse~scale(agri)+scale(trans)+scale(elevation)+region_type+scale(trans):region_type+

(rdist|region_code3), family=binomial, data=data_chickpear_subset) summary(model33)

model34<-

glmer(landuse~scale(agri)+scale(trans)+scale(elevation)+region_type+scale(elevation):region_ty pe+(rdist|region_code3), family=binomial, data=data_chickpear_subset) summary(model34)

model35<-

glmer(landuse~scale(agri)+scale(trans)+scale(elevation)+PA+scale(agri):PA+(rdist|region_code

3), family=binomial, data=data_chickpear_subset) summary(model35)

model36<-

glmer(landuse~scale(agri)+scale(trans)+region_type+PA+scale(agri):PA+(rdist|region_code3), family=binomial, data=data_chickpear_subset) summary(model36)

model37<-

glmer(landuse~scale(agri)+scale(elevation)+region_type+PA+scale(agri):PA+(rdist|region_code

3), family=binomial, data=data_chickpear_subset) summary(model37)

model38<-

glmer(landuse~scale(agri)+scale(trans)+scale(elevation)+PA+scale(trans):PA+(rdist|region_code

3), family=binomial, data=data_chickpear_subset) summary(model38)

model39<-

glmer(landuse~scale(agri)+scale(trans)+region_type+PA+scale(trans):PA+(rdist|region_code3), family=binomial, data=data_chickpear_subset) summary(model39)

model40<-

glmer(landuse~scale(trans)+scale(elevation)+region_type+PA+scale(trans):PA+(rdist|region_co de3), family=binomial, data=data_chickpear_subset) summary(model40)

model41<-

glmer(landuse~scale(agri)+scale(trans)+scale(elevation)+PA+scale(elevation):PA+(rdist|region_ code3), family=binomial, data=data_chickpear_subset) summary(model41)

model42<-

glmer(landuse~scale(agri)+scale(elevation)+region_type+PA+scale(elevation):PA+(rdist|region_ code3), family=binomial, data=data_chickpear_subset) summary(model42)

model43<-

glmer(landuse~scale(trans)+scale(elevation)+region_type+PA+scale(elevation):PA+(rdist|region

_code3), family=binomial, data=data_chickpear_subset) summary(model43)

model44<-

glmer(landuse~scale(agri)+scale(trans)+region_type+PA+region_type:PA+(rdist|region_code3), family=binomial, data=data_chickpear_subset) summary(model44)

model45<-

glmer(landuse~scale(agri)+scale(elevation)+region_type+PA+region_type:PA+(rdist|region_cod e3), family=binomial, data=data_chickpear_subset) summary(model45)

model46<-

glmer(landuse~scale(trans)+scale(elevation)+region_type+PA+region_type:PA+(rdist|region_co de3), family=binomial, data=data_chickpear_subset) summary(model46)

###two interactions###

## with region

model47<-

glmer(landuse~scale(agri)+scale(trans)+scale(elevation)+region_type+scale(agri):region_type+s cale(trans):region_type+(rdist|region_code3), family=binomial, data=data_chickpear_subset) summary(model47)

model48<-

glmer(landuse~scale(agri)+scale(trans)+scale(elevation)+region_type+scale(agri):region_type+s cale(elevation):region_type+(rdist|region_code3), family=binomial, data=data_chickpear_subset) summary(model48)

model49<-

glmer(landuse~scale(agri)+scale(trans)+scale(elevation)+region_type+scale(trans):region_type+ scale(elevation):region_type+(rdist|region_code3), family=binomial, data=data_chickpear_subset) summary(model49)

##with PA model50<-

glmer(landuse~scale(agri)+scale(trans)+scale(elevation)+PA+scale(agri):PA+scale(trans):PA+(r dist|region_code3), family=binomial, data=data_chickpear_subset)

summary(model50)

model51<-

glmer(landuse~scale(agri)+scale(trans)+region_type+PA+scale(agri):PA+scale(trans):PA+(rdist| region_code3), family=binomial, data=data_chickpear_subset) summary(model51)

model52<-

glmer(landuse~scale(agri)+scale(trans)+scale(elevation)+PA+scale(agri):PA+scale(elevation):P

A+(rdist|region_code3), family=binomial, data=data_chickpear_subset) summary(model52)

model53<-

glmer(landuse~scale(agri)+scale(elevation)+region_type+PA+scale(agri):PA+scale(elevation):P

A+(rdist|region_code3), family=binomial, data=data_chickpear_subset) summary(model53)

model54<-

glmer(landuse~scale(agri)+scale(trans)+region_type+PA+scale(agri):PA+region_type:PA+(rdist| region_code3), family=binomial, data=data_chickpear_subset) summary(model54)

model55<-

glmer(landuse~scale(agri)+scale(elevation)+region_type+PA+scale(agri):PA+region_type:PA+( rdist|region_code3), family=binomial, data=data_chickpear_subset) summary(model55)

model56<-

glmer(landuse~scale(agri)+scale(trans)+scale(elevation)+PA+scale(trans):PA+scale(elevation):P

A+(rdist|region_code3), family=binomial, data=data_chickpear_subset) summary(model56)

model57<glmer(landuse~scale(trans)+scale(elevation)+region_type+PA+scale(trans):PA+scale(elevation): PA+(rdist|region_code3), family=binomial, data=data_chickpear_subset) summary(model57)

model58<-

glmer(landuse~scale(agri)+scale(trans)+region_type+PA+scale(trans):PA+region_type:PA+(rdis t|region_code3), family=binomial, data=data_chickpear_subset) summary(model58)

model59<-

glmer(landuse~scale(trans)+scale(elevation)+region_type+PA+scale(trans):PA+region_type:PA

+(rdist|region_code3), family=binomial, data=data_chickpear_subset) summary(model59)

##combine two sets model60<-

glmer(landuse~scale(agri)+scale(trans)+region_type+PA+scale(agri):region_type+scale(agri):P

A+(rdist|region_code3), family=binomial, data=data_chickpear_subset) summary(model60)

model61<-

glmer(landuse~scale(agri)+scale(elevation)+region_type+PA+scale(agri):region_type+scale(agri

):PA+(rdist|region_code3), family=binomial, data=data_chickpear_subset) summary(model61)

model62<-

glmer(landuse~scale(agri)+scale(trans)+region_type+PA+scale(agri):region_type+scale(trans):P

A+(rdist|region_code3), family=binomial, data=data_chickpear_subset) summary(model62)

model63<-

glmer(landuse~scale(agri)+scale(elevation)+region_type+PA+scale(agri):region_type+scale(elev ation):PA+(rdist|region_code3), family=binomial, data=data_chickpear_subset) summary(model63)

model64<-

glmer(landuse~scale(agri)+scale(trans)+region_type+PA+scale(agri):region_type+region_type:P

A+(rdist|region_code3), family=binomial, data=data_chickpear_subset) summary(model64)

model65<-

glmer(landuse~scale(agri)+scale(elevation)+region_type+PA+scale(agri):region_type+region_ty pe:PA+(rdist|region_code3), family=binomial, data=data_chickpear_subset) summary(model65)

model66<-

glmer(landuse~scale(agri)+scale(trans)+region_type+PA+scale(trans):region_type+scale(agri):P

A+(rdist|region_code3), family=binomial, data=data_chickpear_subset) summary(model66)

model67<glmer(landuse~scale(agri)+scale(trans)+region_type+PA+scale(trans):region_type+scale(trans): PA+(rdist|region_code3), family=binomial, data=data_chickpear_subset) summary(model67)

model68<-

glmer(landuse~scale(elevation)+scale(trans)+region_type+PA+scale(trans):region_type+scale(tr ans):PA+(rdist|region_code3), family=binomial, data=data_chickpear_subset) summary(model68)

model69<-

glmer(landuse~scale(trans)+scale(elevation)+region_type+PA+scale(trans):region_type+scale(el evation):PA+(rdist|region_code3), family=binomial, data=data_chickpear_subset) summary(model69)

model70<glmer(landuse~scale(agri)+scale(trans)+region_type+PA+scale(trans):region_type+region_type: PA+(rdist|region_code3), family=binomial, data=data_chickpear_subset) summary(model70)

model71<-

glmer(landuse~scale(trans)+scale(elevation)+region_type+PA+scale(trans):region_type+region_ type:PA+(rdist|region_code3), family=binomial, data=data_chickpear_subset) summary(model71)

model72<-

glmer(landuse~scale(agri)+scale(elevation)+region_type+PA+scale(elevation):region_type+scal e(agri):PA+(rdist|region_code3), family=binomial, data=data_chickpear_subset) summary(model72)

model73<-

glmer(landuse~scale(trans)+scale(elevation)+region_type+PA+scale(elevation):region_type+sca le(trans):PA+(rdist|region_code3), family=binomial, data=data_chickpear_subset) summary(model73)

model74<-

glmer(landuse~scale(agri)+scale(elevation)+region_type+PA+scale(elevation):region_type+scal e(elevation):PA+(rdist|region_code3), family=binomial, data=data_chickpear_subset) summary(model74)

model75<-

glmer(landuse~scale(trans)+scale(elevation)+region_type+PA+scale(elevation):region_type+sca le(elevation):PA+(rdist|region_code3), family=binomial, data=data_chickpear_subset) summary(model75)

model76<-

glmer(landuse~scale(agri)+scale(elevation)+region_type+PA+scale(elevation):region_type+regi on_type:PA+(rdist|region_code3), family=binomial, data=data_chickpear_subset) summary(model76)

model77<-

glmer(landuse~scale(trans)+scale(elevation)+region_type+PA+scale(elevation):region_type+reg ion_type:PA+(rdist|region_code3), family=binomial, data=data_chickpear_subset) summary(model77)

###three interactions###

model78<-

glmer(landuse~scale(agri)+scale(trans)+scale(elevation)+region_type+scale(agri):region_type+s cale(elevation):region_type+scale(trans):region_type+(rdist|region_code3), family=binomial, data=data_chickpear_subset) summary(model78)

model79<-

glmer(landuse~scale(agri)+scale(trans)+region_type+PA+scale(agri):region_type+scale(agri):P A+scale(trans):region_type+(rdist|region_code3), family=binomial, data=data_chickpear_subset) summary(model79)

model80<-

glmer(landuse~scale(agri)+scale(trans)+region_type+PA+scale(agri):region_type+scale(trans):P A+scale(trans):region_type+(rdist|region_code3), family=binomial, data=data_chickpear_subset) summary(model80)

model81<-

glmer(landuse~scale(agri)+scale(trans)+region_type+PA+scale(agri):region_type+region_type:P A+scale(trans):region_type+(rdist|region_code3), family=binomial, data=data_chickpear_subset) summary(model81)

model82<-

glmer(landuse~scale(agri)+scale(elevation)+region_type+PA+scale(agri):region_type+scale(agri

):PA+scale(elevation):region_type+(rdist|region_code3), family=binomial, data=data_chickpear_subset) summary(model82)

model83<-

glmer(landuse~scale(agri)+scale(elevation)+region_type+PA+scale(agri):region_type+scale(elev ation):PA+scale(elevation):region_type+(rdist|region_code3), family=binomial, data=data_chickpear_subset) summary(model83)

model84<-

glmer(landuse~scale(agri)+scale(elevation)+region_type+PA+scale(agri):region_type+region_ty pe:PA+scale(elevation):region_type+(rdist|region_code3), family=binomial, data=data_chickpear_subset) summary(model84)

model85<-

glmer(landuse~scale(trans)+scale(elevation)+region_type+PA+scale(trans):region_type+scale(tr ans):PA+scale(elevation):region_type+(rdist|region_code3), family=binomial, data=data_chickpear_subset) summary(model85)

model86<-

glmer(landuse~scale(trans)+scale(elevation)+region_type+PA+scale(trans):region_type+scale(el evation):PA+scale(elevation):region_type+(rdist|region_code3), family=binomial, data=data_chickpear_subset) summary(model86)

model87<-

glmer(landuse~scale(trans)+scale(elevation)+region_type+PA+scale(trans):region_type+region_ type:PA+scale(elevation):region_type+(rdist|region_code3), family=binomial, data=data_chickpear_subset)

summary(model87)

###four interactions### model88<-

glmer(landuse~scale(agri)+scale(trans)+region_type+PA+scale(agri):region_type+scale(trans):re gion_type+scale(agri):PA+scale(trans):PA+(rdist|region_code3), family=binomial, data=data_chickpear_subset) summary(model88)

model89<-

glmer(landuse~scale(agri)+scale(trans)+region_type+PA+scale(agri):region_type+scale(trans):re gion_type+scale(agri):PA+region_type:PA+(rdist|region_code3), family=binomial, data=data_chickpear_subset) summary(model89)

model90<-

glmer(landuse~scale(agri)+scale(trans)+region_type+PA+scale(agri):region_type+scale(trans):re gion_type+scale(trans):PA+region_type:PA+(rdist|region_code3), family=binomial, data=data_chickpear_subset) summary(model90)

model91<-

glmer(landuse~scale(agri)+scale(elevation)+region_type+PA+scale(agri):region_type+scale(elev ation):region_type+scale(agri):PA+scale(elevation):PA+(rdist|region_code3), family=binomial, data=data_chickpear_subset) summary(model91)

model92<-

glmer(landuse~scale(agri)+scale(elevation)+region_type+PA+scale(agri):region_type+scale(elev ation):region_type+scale(agri):PA+region_type:PA+(rdist|region_code3), family=binomial, data=data_chickpear_subset) summary(model92)

model93<-

glmer(landuse~scale(agri)+scale(elevation)+region_type+PA+scale(agri):region_type+scale(elev ation):region_type+region_type:PA+scale(elevation):PA+(rdist|region_code3), family=binomial, data=data_chickpear_subset) summary(model93)

model94<-

glmer(landuse~scale(trans)+scale(elevation)+region_type+PA+scale(trans):region_type+scale(el evation):region_type+scale(trans):PA+scale(elevation):PA+(rdist|region_code3), family=binomial, data=data_chickpear_subset) summary(model94)

model95<-

glmer(landuse~scale(trans)+scale(elevation)+region_type+PA+scale(trans):region_type+scale(el evation):region_type+scale(trans):PA+region_type:PA+(rdist|region_code3), family=binomial, data=data_chickpear_subset) summary(model95)

model96<-

glmer(landuse~scale(trans)+scale(elevation)+region_type+PA+scale(trans):region_type+scale(el evation):region_type+region_type:PA+scale(elevation):PA+(rdist|region_code3), family=binomial, data=data_chickpear_subset) summary(model96)

###try five interactions### model97<-

glmer(landuse~scale(agri)+scale(trans)+region_type+PA+scale(agri):region_type+scale(trans):re gion_type+scale(agri):PA+scale(trans):PA+region_type:PA+(rdist|region_code3), family=binomial, data=data_chickpear_subset) summary(model97)

model98<-

glmer(landuse~scale(agri)+scale(elevation)+region_type+PA+scale(agri):region_type+scale(elev ation):region_type+scale(agri):PA+scale(elevation):PA+region_type:PA+(rdist|region_code3), family=binomial, data=data_chickpear_subset) summary(model98)

model99<-

glmer(landuse~scale(trans)+scale(elevation)+region_type+PA+scale(trans):region_type+scale(el evation):region_type+scale(trans):PA+scale(elevation):PA+region_type:PA+(rdist|region_code3

), family=binomial, data=data_chickpear_subset) summary(model99)

##select the best model library(MuMIn)

models_sel_chickpear<-

model.sel(model_null,model1,model2,model3,model4,model5,model6,model7, model8, model9, model10, model11,model12, model13, model14, model15,model16,model19,model20,model22,model24,model35,rank="AIC") sink(file="models_sel_chickpear.txt")

models_sel_chickpear

sink()

##dignostic plots library(arm) binnedplot(predict(model35, type="response"), resid(model35, type="response"))

library(effects) plot(allEffects(model35)) allEffects(model35)

sink(file="alleffects(model35).txt") allEffects(model35) sink()

sink("summary(model35).txt") summary(model35) sink()

####################prediction with actual yield ###############################

#1. Model baseline scenario, just put the actual yield in pred_chickpear <- predict(model35, data_chickpear_subset, re.form=NULL, type="response") head(pred_chickpear)

write.csv(pred_chickpear, "pred_chickpear.csv")

##########################prediction with potential yield ######################

##get potential yield data data_all<-read.csv("Appendix_S4_Supplementary_Data.csv",header=T)

data_chickpea<-data_all[c(46627:69939),]

data_chickpea_subset<-data_chickpea[data_chickpea$land_use!=2,]

data_chickpea_subset$land_use <- as.factor(data_chickpea_subset$land_use) data_chickpea_subset$region_code1 <- as.factor(data_chickpea_subset$region_code1) data_chickpea_subset$region_code2 <- as.factor(data_chickpea_subset$region_code2) data_chickpea_subset$region_code3 <- as.factor(data_chickpea_subset$region_code3) data_chickpea_subset$resid<-as.factor(data_chickpea_subset$resid) data_chickpea_subset$PA<- as.factor(data_chickpea_subset$PA)

colnames(data_chickpea_subset) <- c("landuse", "agri", "trans", "elevation",

"region_type","region_code1","region_code2","region_code3","rdist", "resid","PA","ID") head(data_chickpea_subset)

#2. scenario: actual yield being replaced by potential yield data pred_chickpea <- predict(model35, data_chickpea_subset, re.form=NULL, type="response") head(pred_chickpea)

write.csv(pred_chickpea, "pred_chickpea.csv")

#3. Scenario A: agr increase 50%

chickpea_y <- cbind(data_chickpea_subset$landuse, (data_chickpea_subset$agri)*1.5 , data_chickpea_subset[,3:12])

colnames(chickpea_y) <- c("landuse", "agri", "trans","elevation","region_type","region_code1","region_code2","region_code3","rdist","resid","PA","ID")

summary(chickpea_y)

pred_chickpea<-predict(model35, chickpea_y, re.form=NULL, type="response"))

write.csv(pred_chickpea, "pred_chickpea_agri.csv")

#4. Scenario B: transport costs increase by 35%

chickpea_trans_y <- cbind(data_chickpea_subset[,1:2], data_chickpea_subset[,3]*1.35, data_chickpea_subset[,4:12]) colnames(chickpea_trans_y) <- c("landuse", "agri",

"trans","elevation","region_type","region_code1","region_code2","region_code3","rdist","resid",

"PA","ID") summary(chickpea_trans_y)

pred_trans_chickpea<-predict(model35, chickpea_trans_y, re.form=NULL, type="response")

write.csv(pred_trans_chickpea, "pred_chickpea_trans.csv")

#5. Scenario C: political stability has total conversion

region_chickpea <- data_chickpea_subset

summary(region_chickpea) region_chickpea[,5] <- "Division"

region_chickpea[,5] <- as.factor(region_chickpea[,5])

pred_region_chickpea <- predict(model35, region_chickpea, re.form=NULL, type="response") head(pred_region_chickpea)

write.csv(pred_region_chickpea, "pred_chickpea_stability.csv")

#6. Scenario D: worst case. agri increase 50%, and stability has total conversion chickpea_worstcase<-chickpea_y

chickpea_worstcase[,5]<-"Division"

pred_worstcase_chickpea<-predict(model35, chickpea_worstcase, re.form=NULL, type="response")

write.csv(pred_worstcase_chickpea, "pred_chickpea_worstcase.csv")

rm(list=ls())

############################################################################## #################model land use with characteristics of pigeonpea######################

setwd("D:\\Myanmar project\\Resubmission 2\\Code and data (submit)")

data_all_raman<-read.csv("Appendix_S3_Supplementary_Data.csv",header=T)

data_pigeonpear<-data_all_raman[c(69940:93252),]

data_pigeonpear_subset<-data_pigeonpear[data_pigeonpear$land_use!=2,]

#convert to factors data_pigeonpear_subset$land_use <- as.factor(data_pigeonpear_subset$land_use) data_pigeonpear_subset$region_code1 <- as.factor(data_pigeonpear_subset$region_code1) data_pigeonpear_subset$region_code2 <- as.factor(data_pigeonpear_subset$region_code2) data_pigeonpear_subset$region_code3 <- as.factor(data_pigeonpear_subset$region_code3) data_pigeonpear_subset$resid<-as.factor(data_pigeonpear_subset$resid) data_pigeonpear_subset$PA<- as.factor(data_pigeonpear_subset$PA) colnames(data_pigeonpear_subset) <- c("landuse", "agri", "trans", "elevation",

"region_type","region_code1","region_code2","region_code3","rdist", "resid","PA","ID") head(data_pigeonpear_subset)

library(lme4)

##check collinearity

library(car)

model_vif <- glm(landuse ~ scale(agri)+scale(trans)+scale(elevation) +region_type+PA, family=binomial, data=data_pigeonpear_subset) summary(model_vif)

vif(model_vif)

###try to fix the random effects first

model_r1 <- glmer(landuse ~ scale(agri)+scale(trans)+scale(elevation) +region_type+PA+

(1|region_code1), family=binomial, data=data_pigeonpear_subset) summary(model_r1)

model_r2 <- glmer(landuse ~ scale(agri)+scale(trans) +scale(elevation)+region_type+PA+

(1|region_code2), family=binomial, data=data_pigeonpear_subset) summary(model_r2)

model_r3 <- glmer(landuse ~ scale(agri)+scale(trans)+scale(elevation) +region_type+PA+

(1|region_code3), family=binomial, data=data_pigeonpear_subset) summary(model_r3)

AIC(model_r1, model_r2, model_r3)

#choose region_code3 with smallest AIC

model_r4 <- glmer(landuse ~ scale(agri)+scale(trans)+scale(elevation) +region_type+PA+

(rdist|region_code3), family=binomial, data=data_pigeonpear_subset) summary(model_r4)

AIC(model_r3,model_r4)

##choose model_r4 with samllest AIC

###one variable###

model_null<-glmer(landuse~1+(rdist|region_code3), family=binomial, data=data_pigeonpear_subset) summary(model_null) model1<-glmer(landuse~scale(agri)+(rdist|region_code3), family=binomial, data=data_pigeonpear_subset) summary(model1)

model2<-glmer(landuse~scale(trans)+(rdist|region_code3), family=binomial, data=data_pigeonpear_subset) summary(model2)

model3<-glmer(landuse~scale(elevation)+(rdist|region_code3), family=binomial, data=data_pigeonpear_subset) summary(model3)

model4<-glmer(landuse~region_type+(rdist|region_code3), family=binomial, data=data_pigeonpear_subset) summary(model4)

model5<-glmer(landuse~PA+(rdist|region_code3), family=binomial, data=data_pigeonpear_subset) summary(model5)

###two variables###

model6<-glmer(landuse~scale(agri)+scale(trans)+(rdist|region_code3), family=binomial, data=data_pigeonpear_subset) summary(model6)

model7<-glmer(landuse~scale(agri)+scale(elevation)+(rdist|region_code3), family=binomial, data=data_pigeonpear_subset) summary(model7)

model8<-glmer(landuse~scale(agri)+region_type+(rdist|region_code3), family=binomial, data=data_pigeonpear_subset) summary(model8)

model9<-glmer(landuse~scale(trans)+region_type+(rdist|region_code3), family=binomial, data=data_pigeonpear_subset) summary(model9)

model10<-glmer(landuse~scale(elevation)+region_type+(rdist|region_code3), family=binomial, data=data_pigeonpear_subset) summary(model10)

model11<-glmer(landuse~scale(trans)+scale(elevation)+(rdist|region_code3), family=binomial, data=data_pigeonpear_subset) summary(model11)

model12<-glmer(landuse~scale(agri)+PA+(rdist|region_code3), family=binomial, data=data_pigeonpear_subset) summary(model12)

model13<-glmer(landuse~scale(trans)+PA+(rdist|region_code3), family=binomial, data=data_pigeonpear_subset) summary(model13)

model14<-glmer(landuse~scale(elevation)+PA+(rdist|region_code3), family=binomial, data=data_pigeonpear_subset) summary(model14)

model15<-glmer(landuse~region_type+PA+(rdist|region_code3), family=binomial, data=data_pigeonpear_subset) summary(model15)

###three variables###

model16<-glmer(landuse~scale(agri)+scale(trans)+region_type+(rdist|region_code3), family=binomial, data=data_pigeonpear_subset) summary(model16)

model17<-glmer(landuse~scale(agri)+scale(elevation)+region_type+(rdist|region_code3), family=binomial, data=data_pigeonpear_subset) summary(model17)

model18<-glmer(landuse~scale(agri)+scale(trans)+scale(elevation)+(rdist|region_code3), family=binomial, data=data_pigeonpear_subset) summary(model18)

model19<-glmer(landuse~scale(trans)+scale(elevation)+region_type+(rdist|region_code3), family=binomial, data=data_pigeonpear_subset) summary(model19)

model20<-glmer(landuse~scale(agri)+scale(trans)+PA+(rdist|region_code3), family=binomial, data=data_pigeonpear_subset) summary(model20)

model21<-glmer(landuse~scale(agri)+scale(elevation)+PA+(rdist|region_code3), family=binomial, data=data_pigeonpear_subset) summary(model21)

model22<-glmer(landuse~scale(agri)+region_type+PA+(rdist|region_code3), family=binomial, data=data_pigeonpear_subset)

summary(model22)

model23<-glmer(landuse~scale(trans)+scale(elevation)+PA+(rdist|region_code3), family=binomial, data=data_pigeonpear_subset) summary(model23)

model24<-glmer(landuse~scale(trans)+region_type+PA+(rdist|region_code3), family=binomial, data=data_pigeonpear_subset) summary(model24)

model25<-glmer(landuse~scale(elevation)+region_type+PA+(rdist|region_code3), family=binomial, data=data_pigeonpear_subset) summary(model25)

###four variables###

model26<-

glmer(landuse~scale(agri)+scale(trans)+scale(elevation)+region_type+(rdist|region_code3), family=binomial, data=data_pigeonpear_subset) summary(model26)

model27<-glmer(landuse~scale(agri)+scale(trans)+scale(elevation)+PA+(rdist|region_code3), family=binomial, data=data_pigeonpear_subset) summary(model27)

model28<-glmer(landuse~scale(agri)+scale(trans)+PA+region_type+(rdist|region_code3), family=binomial, data=data_pigeonpear_subset) summary(model28)

model29<-glmer(landuse~scale(agri)+PA+scale(elevation)+region_type+(rdist|region_code3), family=binomial, data=data_pigeonpear_subset)

summary(model29)

model30<-glmer(landuse~PA+scale(trans)+scale(elevation)+region_type+(rdist|region_code3), family=binomial, data=data_pigeonpear_subset) summary(model30)

###five variables###

model31<-glmer(landuse ~ scale(agri)+scale(trans)+scale(elevation)

+region_type+PA+(rdist|region_code3), family=binomial, data=data_pigeonpear_subset)

###one interaction###

model32<-

glmer(landuse~scale(agri)+scale(trans)+scale(elevation)+region_type+scale(agri):region_type+(r dist|region_code3), family=binomial, data=data_pigeonpear_subset) summary(model32)

model33<-

glmer(landuse~scale(agri)+scale(trans)+scale(elevation)+region_type+scale(trans):region_type+

(rdist|region_code3), family=binomial, data=data_pigeonpear_subset) summary(model33)

model34<-

glmer(landuse~scale(agri)+scale(trans)+scale(elevation)+region_type+scale(elevation):region_ty pe+(rdist|region_code3), family=binomial, data=data_pigeonpear_subset) summary(model34)

model35<-

glmer(landuse~scale(agri)+scale(trans)+scale(elevation)+PA+scale(agri):PA+(rdist|region_code

3), family=binomial, data=data_pigeonpear_subset) summary(model35)

model36<-

glmer(landuse~scale(agri)+scale(trans)+region_type+PA+scale(agri):PA+(rdist|region_code3), family=binomial, data=data_pigeonpear_subset) summary(model36)

model37<-

glmer(landuse~scale(agri)+scale(elevation)+region_type+PA+scale(agri):PA+(rdist|region_code

3), family=binomial, data=data_pigeonpear_subset) summary(model37)

model38<-

glmer(landuse~scale(agri)+scale(trans)+scale(elevation)+PA+scale(trans):PA+(rdist|region_code

3), family=binomial, data=data_pigeonpear_subset) summary(model38)

model39<-

glmer(landuse~scale(agri)+scale(trans)+region_type+PA+scale(trans):PA+(rdist|region_code3), family=binomial, data=data_pigeonpear_subset) summary(model39)

model40<-

glmer(landuse~scale(trans)+scale(elevation)+region_type+PA+scale(trans):PA+(rdist|region_co de3), family=binomial, data=data_pigeonpear_subset) summary(model40)

model41<-

glmer(landuse~scale(agri)+scale(trans)+scale(elevation)+PA+scale(elevation):PA+(rdist|region_ code3), family=binomial, data=data_pigeonpear_subset) summary(model41)

model42<-

glmer(landuse~scale(agri)+scale(elevation)+region_type+PA+scale(elevation):PA+(rdist|region_ code3), family=binomial, data=data_pigeonpear_subset) summary(model42)

model43<-

glmer(landuse~scale(trans)+scale(elevation)+region_type+PA+scale(elevation):PA+(rdist|region

_code3), family=binomial, data=data_pigeonpear_subset) summary(model43)

model44<-

glmer(landuse~scale(agri)+scale(trans)+region_type+PA+region_type:PA+(rdist|region_code3), family=binomial, data=data_pigeonpear_subset) summary(model44)

model45<-

glmer(landuse~scale(agri)+scale(elevation)+region_type+PA+region_type:PA+(rdist|region_cod e3), family=binomial, data=data_pigeonpear_subset) summary(model45)

model46<-

glmer(landuse~scale(trans)+scale(elevation)+region_type+PA+region_type:PA+(rdist|region_co de3), family=binomial, data=data_pigeonpear_subset) summary(model46)

###two interactions###

## with region

model47<-

glmer(landuse~scale(agri)+scale(trans)+scale(elevation)+region_type+scale(agri):region_type+s cale(trans):region_type+(rdist|region_code3), family=binomial, data=data_pigeonpear_subset) summary(model47)

model48<-

glmer(landuse~scale(agri)+scale(trans)+scale(elevation)+region_type+scale(agri):region_type+s cale(elevation):region_type+(rdist|region_code3), family=binomial, data=data_pigeonpear_subset) summary(model48)

model49<-

glmer(landuse~scale(agri)+scale(trans)+scale(elevation)+region_type+scale(trans):region_type+ scale(elevation):region_type+(rdist|region_code3), family=binomial, data=data_pigeonpear_subset) summary(model49)

##with PA model50<-

glmer(landuse~scale(agri)+scale(trans)+scale(elevation)+PA+scale(agri):PA+scale(trans):PA+(r dist|region_code3), family=binomial, data=data_pigeonpear_subset) summary(model50)

model51<-

glmer(landuse~scale(agri)+scale(trans)+region_type+PA+scale(agri):PA+scale(trans):PA+(rdist| region_code3), family=binomial, data=data_pigeonpear_subset) summary(model51)

model52<-

glmer(landuse~scale(agri)+scale(trans)+scale(elevation)+PA+scale(agri):PA+scale(elevation):P A+(rdist|region_code3), family=binomial, data=data_pigeonpear_subset) summary(model52)

model53<-

glmer(landuse~scale(agri)+scale(elevation)+region_type+PA+scale(agri):PA+scale(elevation):P

A+(rdist|region_code3), family=binomial, data=data_pigeonpear_subset)

summary(model53)

model54<-

glmer(landuse~scale(agri)+scale(trans)+region_type+PA+scale(agri):PA+region_type:PA+(rdist| region_code3), family=binomial, data=data_pigeonpear_subset) summary(model54)

model55<-

glmer(landuse~scale(agri)+scale(elevation)+region_type+PA+scale(agri):PA+region_type:PA+( rdist|region_code3), family=binomial, data=data_pigeonpear_subset) summary(model55)

model56<-

glmer(landuse~scale(agri)+scale(trans)+scale(elevation)+PA+scale(trans):PA+scale(elevation):P

A+(rdist|region_code3), family=binomial, data=data_pigeonpear_subset) summary(model56)

model57<glmer(landuse~scale(trans)+scale(elevation)+region_type+PA+scale(trans):PA+scale(elevation): PA+(rdist|region_code3), family=binomial, data=data_pigeonpear_subset) summary(model57)

model58<-

glmer(landuse~scale(agri)+scale(trans)+region_type+PA+scale(trans):PA+region_type:PA+(rdis t|region_code3), family=binomial, data=data_pigeonpear_subset) summary(model58)

model59<-

glmer(landuse~scale(trans)+scale(elevation)+region_type+PA+scale(trans):PA+region_type:PA

+(rdist|region_code3), family=binomial, data=data_pigeonpear_subset) summary(model59)

##combine two sets model60<-

glmer(landuse~scale(agri)+scale(trans)+region_type+PA+scale(agri):region_type+scale(agri):P

A+(rdist|region_code3), family=binomial, data=data_pigeonpear_subset) summary(model60)

model61<-

glmer(landuse~scale(agri)+scale(elevation)+region_type+PA+scale(agri):region_type+scale(agri

):PA+(rdist|region_code3), family=binomial, data=data_pigeonpear_subset) summary(model61)

model62<-

glmer(landuse~scale(agri)+scale(trans)+region_type+PA+scale(agri):region_type+scale(trans):P

A+(rdist|region_code3), family=binomial, data=data_pigeonpear_subset) summary(model62)

model63<-

glmer(landuse~scale(agri)+scale(elevation)+region_type+PA+scale(agri):region_type+scale(elev ation):PA+(rdist|region_code3), family=binomial, data=data_pigeonpear_subset) summary(model63)

model64<-

glmer(landuse~scale(agri)+scale(trans)+region_type+PA+scale(agri):region_type+region_type:P

A+(rdist|region_code3), family=binomial, data=data_pigeonpear_subset) summary(model64)

model65<-

glmer(landuse~scale(agri)+scale(elevation)+region_type+PA+scale(agri):region_type+region_ty pe:PA+(rdist|region_code3), family=binomial, data=data_pigeonpear_subset) summary(model65)

model66<-

glmer(landuse~scale(agri)+scale(trans)+region_type+PA+scale(trans):region_type+scale(agri):P

A+(rdist|region_code3), family=binomial, data=data_pigeonpear_subset) summary(model66)

model67<glmer(landuse~scale(agri)+scale(trans)+region_type+PA+scale(trans):region_type+scale(trans): PA+(rdist|region_code3), family=binomial, data=data_pigeonpear_subset) summary(model67)

model68<-

glmer(landuse~scale(elevation)+scale(trans)+region_type+PA+scale(trans):region_type+scale(tr ans):PA+(rdist|region_code3), family=binomial, data=data_pigeonpear_subset) summary(model68)

model69<-

glmer(landuse~scale(trans)+scale(elevation)+region_type+PA+scale(trans):region_type+scale(el evation):PA+(rdist|region_code3), family=binomial, data=data_pigeonpear_subset) summary(model69)

model70<glmer(landuse~scale(agri)+scale(trans)+region_type+PA+scale(trans):region_type+region_type: PA+(rdist|region_code3), family=binomial, data=data_pigeonpear_subset) summary(model70)

model71<-

glmer(landuse~scale(trans)+scale(elevation)+region_type+PA+scale(trans):region_type+region_ type:PA+(rdist|region_code3), family=binomial, data=data_pigeonpear_subset) summary(model71)

model72<-

glmer(landuse~scale(agri)+scale(elevation)+region_type+PA+scale(elevation):region_type+scal e(agri):PA+(rdist|region_code3), family=binomial, data=data_pigeonpear_subset) summary(model72)

model73<-

glmer(landuse~scale(trans)+scale(elevation)+region_type+PA+scale(elevation):region_type+sca le(trans):PA+(rdist|region_code3), family=binomial, data=data_pigeonpear_subset) summary(model73)

model74<-

glmer(landuse~scale(agri)+scale(elevation)+region_type+PA+scale(elevation):region_type+scal e(elevation):PA+(rdist|region_code3), family=binomial, data=data_pigeonpear_subset) summary(model74)

model75<-

glmer(landuse~scale(trans)+scale(elevation)+region_type+PA+scale(elevation):region_type+sca le(elevation):PA+(rdist|region_code3), family=binomial, data=data_pigeonpear_subset) summary(model75)

model76<-

glmer(landuse~scale(agri)+scale(elevation)+region_type+PA+scale(elevation):region_type+regi on_type:PA+(rdist|region_code3), family=binomial, data=data_pigeonpear_subset) summary(model76)

model77<-

glmer(landuse~scale(trans)+scale(elevation)+region_type+PA+scale(elevation):region_type+reg ion_type:PA+(rdist|region_code3), family=binomial, data=data_pigeonpear_subset) summary(model77)

###three interactions###

model78<-

glmer(landuse~scale(agri)+scale(trans)+scale(elevation)+region_type+scale(agri):region_type+s cale(elevation):region_type+scale(trans):region_type+(rdist|region_code3), family=binomial, data=data_pigeonpear_subset)

summary(model78)

model79<-

glmer(landuse~scale(agri)+scale(trans)+region_type+PA+scale(agri):region_type+scale(agri):P

A+scale(trans):region_type+(rdist|region_code3), family=binomial, data=data_pigeonpear_subset) summary(model79)

model80<-

glmer(landuse~scale(agri)+scale(trans)+region_type+PA+scale(agri):region_type+scale(trans):P

A+scale(trans):region_type+(rdist|region_code3), family=binomial, data=data_pigeonpear_subset) summary(model80)

model81<-

glmer(landuse~scale(agri)+scale(trans)+region_type+PA+scale(agri):region_type+region_type:P

A+scale(trans):region_type+(rdist|region_code3), family=binomial, data=data_pigeonpear_subset) summary(model81)

model82<-

glmer(landuse~scale(agri)+scale(elevation)+region_type+PA+scale(agri):region_type+scale(agri

):PA+scale(elevation):region_type+(rdist|region_code3), family=binomial, data=data_pigeonpear_subset) summary(model82)

model83<-

glmer(landuse~scale(agri)+scale(elevation)+region_type+PA+scale(agri):region_type+scale(elev ation):PA+scale(elevation):region_type+(rdist|region_code3), family=binomial, data=data_pigeonpear_subset) summary(model83)

model84<-

glmer(landuse~scale(agri)+scale(elevation)+region_type+PA+scale(agri):region_type+region_ty pe:PA+scale(elevation):region_type+(rdist|region_code3), family=binomial, data=data_pigeonpear_subset) summary(model84)

model85<-

glmer(landuse~scale(trans)+scale(elevation)+region_type+PA+scale(trans):region_type+scale(tr ans):PA+scale(elevation):region_type+(rdist|region_code3), family=binomial, data=data_pigeonpear_subset) summary(model85)

model86<-

glmer(landuse~scale(trans)+scale(elevation)+region_type+PA+scale(trans):region_type+scale(el evation):PA+scale(elevation):region_type+(rdist|region_code3), family=binomial, data=data_pigeonpear_subset) summary(model86)

model87<-

glmer(landuse~scale(trans)+scale(elevation)+region_type+PA+scale(trans):region_type+region_ type:PA+scale(elevation):region_type+(rdist|region_code3), family=binomial, data=data_pigeonpear_subset) summary(model87)

###four interactions### model88<-

glmer(landuse~scale(agri)+scale(trans)+region_type+PA+scale(agri):region_type+scale(trans):re gion_type+scale(agri):PA+scale(trans):PA+(rdist|region_code3), family=binomial, data=data_pigeonpear_subset) summary(model88)

model89<-

glmer(landuse~scale(agri)+scale(trans)+region_type+PA+scale(agri):region_type+scale(trans):re gion_type+scale(agri):PA+region_type:PA+(rdist|region_code3), family=binomial, data=data_pigeonpear_subset) summary(model89)

model90<-

glmer(landuse~scale(agri)+scale(trans)+region_type+PA+scale(agri):region_type+scale(trans):re gion_type+scale(trans):PA+region_type:PA+(rdist|region_code3), family=binomial, data=data_pigeonpear_subset) summary(model90)

model91<-

glmer(landuse~scale(agri)+scale(elevation)+region_type+PA+scale(agri):region_type+scale(elev ation):region_type+scale(agri):PA+scale(elevation):PA+(rdist|region_code3), family=binomial, data=data_pigeonpear_subset) summary(model91)

model92<-

glmer(landuse~scale(agri)+scale(elevation)+region_type+PA+scale(agri):region_type+scale(elev ation):region_type+scale(agri):PA+region_type:PA+(rdist|region_code3), family=binomial, data=data_pigeonpear_subset) summary(model92)

model93<-

glmer(landuse~scale(agri)+scale(elevation)+region_type+PA+scale(agri):region_type+scale(elev ation):region_type+region_type:PA+scale(elevation):PA+(rdist|region_code3), family=binomial, data=data_pigeonpear_subset) summary(model93)

model94<-

glmer(landuse~scale(trans)+scale(elevation)+region_type+PA+scale(trans):region_type+scale(el evation):region_type+scale(trans):PA+scale(elevation):PA+(rdist|region_code3), family=binomial, data=data_pigeonpear_subset) summary(model94)

model95<-

glmer(landuse~scale(trans)+scale(elevation)+region_type+PA+scale(trans):region_type+scale(el evation):region_type+scale(trans):PA+region_type:PA+(rdist|region_code3), family=binomial, data=data_pigeonpear_subset) summary(model95)

model96<-

glmer(landuse~scale(trans)+scale(elevation)+region_type+PA+scale(trans):region_type+scale(el evation):region_type+region_type:PA+scale(elevation):PA+(rdist|region_code3), family=binomial, data=data_pigeonpear_subset) summary(model96)

###try five interactions### model97<-

glmer(landuse~scale(agri)+scale(trans)+region_type+PA+scale(agri):region_type+scale(trans):re gion_type+scale(agri):PA+scale(trans):PA+region_type:PA+(rdist|region_code3), family=binomial, data=data_pigeonpear_subset) summary(model97)

model98<-

glmer(landuse~scale(agri)+scale(elevation)+region_type+PA+scale(agri):region_type+scale(elev ation):region_type+scale(agri):PA+scale(elevation):PA+region_type:PA+(rdist|region_code3), family=binomial, data=data_pigeonpear_subset) summary(model98)

model99<-

glmer(landuse~scale(trans)+scale(elevation)+region_type+PA+scale(trans):region_type+scale(el evation):region_type+scale(trans):PA+scale(elevation):PA+region_type:PA+(rdist|region_code3

), family=binomial, data=data_pigeonpear_subset) summary(model99)

##select the best model

library(MuMIn)

models_sel_pigeonpear<-

model.sel(model_null,model1,model2,model3,model4,model5,model6,model7, model8, model9, model10,model12, model13, model14,

model15,model16,model19,model20,model22,model24,model37,model39,rank="AIC") sink(file="models_sel_pigeonpear.txt")

models_sel_pigeonpear

sink()

##dignostic plots library(arm) binnedplot(predict(model37, type="response"), resid(model37, type="response"))

library(effects) plot(allEffects(model37)) allEffects(model37)

sink(file="alleffects(model37).txt") allEffects(model37) sink()

sink("summary(model37).txt") summary(model37) sink() ####################prediction with actual yield ###############################

#1. Model baseline scenario, just put the actual yield in pred_pigeonpear <- predict(model37, data_pigeonpear_subset, re.form=NULL, type="response") head(pred_pigeonpear)

write.csv(pred_pigeonpear, "pred_pigeonpear.csv")

##########################prediction with potential yield ###################### ##get potential yield data

data_all<-read.csv("Appendix_S4_Supplementary_Data.csv",header=T)

data_pigeonpea<-data_all[c(1:23313),]

data_pigeonpea_subset<-data_pigeonpea[data_pigeonpea$land_use!=2,]

data_pigeonpea_subset$land_use <- as.factor(data_pigeonpea_subset$land_use) data_pigeonpea_subset$region_code1 <- as.factor(data_pigeonpea_subset$region_code1) data_pigeonpea_subset$region_code2 <- as.factor(data_pigeonpea_subset$region_code2) data_pigeonpea_subset$region_code3 <- as.factor(data_pigeonpea_subset$region_code3) data_pigeonpea_subset$resid<-as.factor(data_pigeonpea_subset$resid) data_pigeonpea_subset$PA<- as.factor(data_pigeonpea_subset$PA)

colnames(data_pigeonpea_subset) <- c("landuse", "agri", "trans", "elevation",

"region_type","region_code1","region_code2","region_code3","rdist", "resid","PA","ID") head(data_pigeonpea_subset)

#2. Scenario: actual yield being replaced by potential yield data pred_pigeonpea <- predict(model37, data_pigeonpea_subset, re.form=NULL, type="response") head(pred_pigeonpea)

write.csv(pred_pigeonpea, "pred_pigeonpea.csv")

#3. Scenario A: agr increase 50%

pigeonpea_y <- cbind(data_pigeonpea_subset$landuse, (data_pigeonpea_subset$agri)*1.5 , data_pigeonpea_subset[,3:12])

colnames(pigeonpea_y) <- c("landuse", "agri", "trans","elevation","region_type","region_code1","region_code2","region_code3","rdist","resid","PA","ID")

summary(pigeonpea_y)

pred_pigeonpea<-predict(model37, pigeonpea_y, re.form=NULL, type="response"))

write.csv(pred_pigeonpea, "pred_pigeonpea_agri.csv")

#4. Scenario B: transport costs increase by 35%

pigeonpea_trans_y <- cbind(data_pigeonpea_subset[,1:2], data_pigeonpea_subset[,3]*1.35, data_pigeonpea_subset[,4:12]) colnames(pigeonpea_trans_y) <- c("landuse", "agri",

"trans","elevation","region_type","region_code1","region_code2","region_code3","rdist","resid",

"PA","ID") summary(pigeonpea_trans_y)

pred_trans_pigeonpea<-predict(model37, pigeonpea_trans_y, re.form=NULL, type="response")

write.csv(pred_trans_pigeonpea, "pred_pigeonpea_trans.csv")

#5. Scenario C: political stability has total conversion

region_pigeonpea <- data_pigeonpea_subset

summary(region_pigeonpea) region_pigeonpea[,5] <- "Division"

region_pigeonpea[,5] <- as.factor(region_pigeonpea[,5])

pred_region_pigeonpea <- predict(model37, region_pigeonpea, re.form=NULL, type="response") head(pred_region_pigeonpea)

write.csv(pred_region_pigeonpea, "pred_pigeonpea_stability.csv")

#6. Scenario D: worst case. agri increase 50%, and stability has total conversion pigeonpea_worstcase<-pigeonpea_y

pigeonpea_worstcase[,5]<-"Division"

pred_worstcase_pigeonpea<-predict(model37, pigeonpea_worstcase, re.form=NULL, type="response")

write.csv(pred_worstcase_pigeonpea, "pred_pigeonpea_worstcase.csv")

rm(list=ls())

##############################################################################

##################model land use with characteristics of cassava#######################

setwd("D:\\Myanmar project\\Resubmission 2\\Code and data (submit)")

data_all_raman<-read.csv("Appendix_S3_Supplementary_Data.csv",header=T)

data_cassavar<-data_all_raman[c(93253:116565),]

data_cassavar_subset<-data_cassavar[data_cassavar$land_use!=2,]

#convert to factors data_cassavar_subset$land_use <- as.factor(data_cassavar_subset$land_use) data_cassavar_subset$region_code1 <- as.factor(data_cassavar_subset$region_code1) data_cassavar_subset$region_code2 <- as.factor(data_cassavar_subset$region_code2) data_cassavar_subset$region_code3 <- as.factor(data_cassavar_subset$region_code3) data_cassavar_subset$resid<-as.factor(data_cassavar_subset$resid) data_cassavar_subset$PA<- as.factor(data_cassavar_subset$PA)

colnames(data_cassavar_subset) <- c("landuse", "agri", "trans", "elevation",

"region_type","region_code1","region_code2","region_code3","rdist", "resid","PA","ID") head(data_cassavar_subset)

library(lme4)

##check collinearity

library(car)

model_vif <- glm(landuse ~ scale(agri)+scale(trans)+scale(elevation) +region_type+PA, family=binomial, data=data_cassavar_subset) summary(model_vif)

vif(model_vif)

###try to fix the random effects first

model_r1 <- glmer(landuse ~ scale(agri)+scale(trans)+scale(elevation) +region_type+PA+

(1|region_code1), family=binomial, data=data_cassavar_subset) summary(model_r1)

model_r2 <- glmer(landuse ~ scale(agri)+scale(trans) +scale(elevation)+region_type+PA+

(1|region_code2), family=binomial, data=data_cassavar_subset) summary(model_r2)

model_r3 <- glmer(landuse ~ scale(agri)+scale(trans)+scale(elevation) +region_type+PA+ (1|region_code3), family=binomial, data=data_cassavar_subset) summary(model_r3)

AIC(model_r1, model_r2, model_r3)

#choose region_code3 with smallest AIC

model_r4 <- glmer(landuse ~ scale(agri)+scale(trans)+scale(elevation) +region_type+PA+

(rdist|region_code3), family=binomial, data=data_cassavar_subset) summary(model_r4)

AIC(model_r3,model_r4)

###one variable### model_null<-glmer(landuse~1+(rdist|region_code3), family=binomial, data=data_cassavar_subset) summary(model_null)

model1<-glmer(landuse~scale(agri)+(rdist|region_code3), family=binomial, data=data_cassavar_subset) summary(model1)

model2<-glmer(landuse~scale(trans)+(rdist|region_code3), family=binomial, data=data_cassavar_subset) summary(model2) model3<-glmer(landuse~scale(elevation)+(rdist|region_code3), family=binomial, data=data_cassavar_subset) summary(model3)

model4<-glmer(landuse~region_type+(rdist|region_code3), family=binomial, data=data_cassavar_subset) summary(model4)

model5<-glmer(landuse~PA+(rdist|region_code3), family=binomial, data=data_cassavar_subset) summary(model5)

###two variables###

model6<-glmer(landuse~scale(agri)+scale(trans)+(rdist|region_code3), family=binomial, data=data_cassavar_subset) summary(model6)

model7<-glmer(landuse~scale(agri)+scale(elevation)+(rdist|region_code3), family=binomial, data=data_cassavar_subset) summary(model7)

model8<-glmer(landuse~scale(agri)+region_type+(rdist|region_code3), family=binomial, data=data_cassavar_subset) summary(model8)

model9<-glmer(landuse~scale(trans)+region_type+(rdist|region_code3), family=binomial, data=data_cassavar_subset) summary(model9)

model10<-glmer(landuse~scale(elevation)+region_type+(rdist|region_code3), family=binomial, data=data_cassavar_subset)

summary(model10)

model11<-glmer(landuse~scale(trans)+scale(elevation)+(rdist|region_code3), family=binomial, data=data_cassavar_subset) summary(model11)

model12<-glmer(landuse~scale(agri)+PA+(rdist|region_code3), family=binomial, data=data_cassavar_subset) summary(model12)

model13<-glmer(landuse~scale(trans)+PA+(rdist|region_code3), family=binomial, data=data_cassavar_subset) summary(model13)

model14<-glmer(landuse~scale(elevation)+PA+(rdist|region_code3), family=binomial, data=data_cassavar_subset) summary(model14)

model15<-glmer(landuse~region_type+PA+(rdist|region_code3), family=binomial, data=data_cassavar_subset) summary(model15)

###three variables###

model16<-glmer(landuse~scale(agri)+scale(trans)+region_type+(rdist|region_code3), family=binomial, data=data_cassavar_subset) summary(model16) model17<-glmer(landuse~scale(agri)+scale(elevation)+region_type+(rdist|region_code3), family=binomial, data=data_cassavar_subset) summary(model17)

model18<-glmer(landuse~scale(agri)+scale(trans)+scale(elevation)+(rdist|region_code3), family=binomial, data=data_cassavar_subset) summary(model18)

model19<-glmer(landuse~scale(trans)+scale(elevation)+region_type+(rdist|region_code3), family=binomial, data=data_cassavar_subset) summary(model19)

model20<-glmer(landuse~scale(agri)+scale(trans)+PA+(rdist|region_code3), family=binomial, data=data_cassavar_subset) summary(model20)

model21<-glmer(landuse~scale(agri)+scale(elevation)+PA+(rdist|region_code3), family=binomial, data=data_cassavar_subset) summary(model21)

model22<-glmer(landuse~scale(agri)+region_type+PA+(rdist|region_code3), family=binomial, data=data_cassavar_subset) summary(model22)

model23<-glmer(landuse~scale(trans)+scale(elevation)+PA+(rdist|region_code3), family=binomial, data=data_cassavar_subset) summary(model23)

model24<-glmer(landuse~scale(trans)+region_type+PA+(rdist|region_code3), family=binomial, data=data_cassavar_subset) summary(model24) model25<-glmer(landuse~scale(elevation)+region_type+PA+(rdist|region_code3), family=binomial, data=data_cassavar_subset) summary(model25)

###four variables###

model26<-

glmer(landuse~scale(agri)+scale(trans)+scale(elevation)+region_type+(rdist|region_code3), family=binomial, data=data_cassavar_subset) summary(model26)

model27<-glmer(landuse~scale(agri)+scale(trans)+scale(elevation)+PA+(rdist|region_code3), family=binomial, data=data_cassavar_subset) summary(model27)

model28<-glmer(landuse~scale(agri)+scale(trans)+PA+region_type+(rdist|region_code3), family=binomial, data=data_cassavar_subset) summary(model28)

model29<-glmer(landuse~scale(agri)+PA+scale(elevation)+region_type+(rdist|region_code3), family=binomial, data=data_cassavar_subset) summary(model29)

model30<-glmer(landuse~PA+scale(trans)+scale(elevation)+region_type+(rdist|region_code3), family=binomial, data=data_cassavar_subset) summary(model30)

###five variables###

model31<-glmer(landuse ~ scale(agri)+scale(trans)+scale(elevation)

+region_type+PA+(rdist|region_code3), family=binomial, data=data_cassavar_subset)

###one interaction###

model32<-

glmer(landuse~scale(agri)+scale(trans)+scale(elevation)+region_type+scale(agri):region_type+(r dist|region_code3), family=binomial, data=data_cassavar_subset) summary(model32)

model33<-

glmer(landuse~scale(agri)+scale(trans)+scale(elevation)+region_type+scale(trans):region_type+

(rdist|region_code3), family=binomial, data=data_cassavar_subset) summary(model33)

model34<-

glmer(landuse~scale(agri)+scale(trans)+scale(elevation)+region_type+scale(elevation):region_ty pe+(rdist|region_code3), family=binomial, data=data_cassavar_subset) summary(model34)

model35<-

glmer(landuse~scale(agri)+scale(trans)+scale(elevation)+PA+scale(agri):PA+(rdist|region_code

3), family=binomial, data=data_cassavar_subset) summary(model35)

model36<-

glmer(landuse~scale(agri)+scale(trans)+region_type+PA+scale(agri):PA+(rdist|region_code3), family=binomial, data=data_cassavar_subset) summary(model36)

model37<-

glmer(landuse~scale(agri)+scale(elevation)+region_type+PA+scale(agri):PA+(rdist|region_code 3), family=binomial, data=data_cassavar_subset)

summary(model37)

model38<-

glmer(landuse~scale(agri)+scale(trans)+scale(elevation)+PA+scale(trans):PA+(rdist|region_code

3), family=binomial, data=data_cassavar_subset) summary(model38)

model39<-

glmer(landuse~scale(agri)+scale(trans)+region_type+PA+scale(trans):PA+(rdist|region_code3), family=binomial, data=data_cassavar_subset) summary(model39)

model40<-

glmer(landuse~scale(trans)+scale(elevation)+region_type+PA+scale(trans):PA+(rdist|region_co de3), family=binomial, data=data_cassavar_subset) summary(model40)

model41<-

glmer(landuse~scale(agri)+scale(trans)+scale(elevation)+PA+scale(elevation):PA+(rdist|region_ code3), family=binomial, data=data_cassavar_subset) summary(model41)

model42<-

glmer(landuse~scale(agri)+scale(elevation)+region_type+PA+scale(elevation):PA+(rdist|region_ code3), family=binomial, data=data_cassavar_subset) summary(model42)

model43<-

glmer(landuse~scale(trans)+scale(elevation)+region_type+PA+scale(elevation):PA+(rdist|region _code3), family=binomial, data=data_cassavar_subset)

summary(model43)

model44<-

glmer(landuse~scale(agri)+scale(trans)+region_type+PA+region_type:PA+(rdist|region_code3), family=binomial, data=data_cassavar_subset) summary(model44)

model45<-

glmer(landuse~scale(agri)+scale(elevation)+region_type+PA+region_type:PA+(rdist|region_cod e3), family=binomial, data=data_cassavar_subset) summary(model45)

model46<-

glmer(landuse~scale(trans)+scale(elevation)+region_type+PA+region_type:PA+(rdist|region_co de3), family=binomial, data=data_cassavar_subset) summary(model46)

###two interactions###

## with region

model47<-

glmer(landuse~scale(agri)+scale(trans)+scale(elevation)+region_type+scale(agri):region_type+s cale(trans):region_type+(rdist|region_code3), family=binomial, data=data_cassavar_subset) summary(model47)

model48<-

glmer(landuse~scale(agri)+scale(trans)+scale(elevation)+region_type+scale(agri):region_type+s cale(elevation):region_type+(rdist|region_code3), family=binomial, data=data_cassavar_subset) summary(model48)

model49<-

glmer(landuse~scale(agri)+scale(trans)+scale(elevation)+region_type+scale(trans):region_type+ scale(elevation):region_type+(rdist|region_code3), family=binomial, data=data_cassavar_subset) summary(model49)

##with PA model50<-

glmer(landuse~scale(agri)+scale(trans)+scale(elevation)+PA+scale(agri):PA+scale(trans):PA+(r dist|region_code3), family=binomial, data=data_cassavar_subset) summary(model50)

model51<-

glmer(landuse~scale(agri)+scale(trans)+region_type+PA+scale(agri):PA+scale(trans):PA+(rdist| region_code3), family=binomial, data=data_cassavar_subset) summary(model51)

model52<-

glmer(landuse~scale(agri)+scale(trans)+scale(elevation)+PA+scale(agri):PA+scale(elevation):P A+(rdist|region_code3), family=binomial, data=data_cassavar_subset) summary(model52)

model53<-

glmer(landuse~scale(agri)+scale(elevation)+region_type+PA+scale(agri):PA+scale(elevation):P

A+(rdist|region_code3), family=binomial, data=data_cassavar_subset) summary(model53)

model54<-

glmer(landuse~scale(agri)+scale(trans)+region_type+PA+scale(agri):PA+region_type:PA+(rdist| region_code3), family=binomial, data=data_cassavar_subset) summary(model54)

model55<-

glmer(landuse~scale(agri)+scale(elevation)+region_type+PA+scale(agri):PA+region_type:PA+( rdist|region_code3), family=binomial, data=data_cassavar_subset) summary(model55)

model56<-

glmer(landuse~scale(agri)+scale(trans)+scale(elevation)+PA+scale(trans):PA+scale(elevation):P

A+(rdist|region_code3), family=binomial, data=data_cassavar_subset) summary(model56)

model57<glmer(landuse~scale(trans)+scale(elevation)+region_type+PA+scale(trans):PA+scale(elevation): PA+(rdist|region_code3), family=binomial, data=data_cassavar_subset) summary(model57)

model58<-

glmer(landuse~scale(agri)+scale(trans)+region_type+PA+scale(trans):PA+region_type:PA+(rdis t|region_code3), family=binomial, data=data_cassavar_subset) summary(model58)

model59<-

glmer(landuse~scale(trans)+scale(elevation)+region_type+PA+scale(trans):PA+region_type:PA

+(rdist|region_code3), family=binomial, data=data_cassavar_subset) summary(model59)

##combine two sets model60<-

glmer(landuse~scale(agri)+scale(trans)+region_type+PA+scale(agri):region_type+scale(agri):P

A+(rdist|region_code3), family=binomial, data=data_cassavar_subset) summary(model60)

model61<-

glmer(landuse~scale(agri)+scale(elevation)+region_type+PA+scale(agri):region_type+scale(agri

):PA+(rdist|region_code3), family=binomial, data=data_cassavar_subset) summary(model61)

model62<-

glmer(landuse~scale(agri)+scale(trans)+region_type+PA+scale(agri):region_type+scale(trans):P

A+(rdist|region_code3), family=binomial, data=data_cassavar_subset) summary(model62)

model63<-

glmer(landuse~scale(agri)+scale(elevation)+region_type+PA+scale(agri):region_type+scale(elev ation):PA+(rdist|region_code3), family=binomial, data=data_cassavar_subset) summary(model63)

model64<-

glmer(landuse~scale(agri)+scale(trans)+region_type+PA+scale(agri):region_type+region_type:P

A+(rdist|region_code3), family=binomial, data=data_cassavar_subset) summary(model64)

model65<-

glmer(landuse~scale(agri)+scale(elevation)+region_type+PA+scale(agri):region_type+region_ty pe:PA+(rdist|region_code3), family=binomial, data=data_cassavar_subset) summary(model65)

model66<-

glmer(landuse~scale(agri)+scale(trans)+region_type+PA+scale(trans):region_type+scale(agri):P

A+(rdist|region_code3), family=binomial, data=data_cassavar_subset) summary(model66)

model67<glmer(landuse~scale(agri)+scale(trans)+region_type+PA+scale(trans):region_type+scale(trans):

PA+(rdist|region_code3), family=binomial, data=data_cassavar_subset)

summary(model67)

model68<-

glmer(landuse~scale(elevation)+scale(trans)+region_type+PA+scale(trans):region_type+scale(tr ans):PA+(rdist|region_code3), family=binomial, data=data_cassavar_subset) summary(model68)

model69<-

glmer(landuse~scale(trans)+scale(elevation)+region_type+PA+scale(trans):region_type+scale(el evation):PA+(rdist|region_code3), family=binomial, data=data_cassavar_subset) summary(model69)

model70<glmer(landuse~scale(agri)+scale(trans)+region_type+PA+scale(trans):region_type+region_type: PA+(rdist|region_code3), family=binomial, data=data_cassavar_subset) summary(model70)

model71<-

glmer(landuse~scale(trans)+scale(elevation)+region_type+PA+scale(trans):region_type+region_ type:PA+(rdist|region_code3), family=binomial, data=data_cassavar_subset) summary(model71)

model72<-

glmer(landuse~scale(agri)+scale(elevation)+region_type+PA+scale(elevation):region_type+scal e(agri):PA+(rdist|region_code3), family=binomial, data=data_cassavar_subset) summary(model72)

model73<-

glmer(landuse~scale(trans)+scale(elevation)+region_type+PA+scale(elevation):region_type+sca le(trans):PA+(rdist|region_code3), family=binomial, data=data_cassavar_subset) summary(model73)

model74<-

glmer(landuse~scale(agri)+scale(elevation)+region_type+PA+scale(elevation):region_type+scal e(elevation):PA+(rdist|region_code3), family=binomial, data=data_cassavar_subset) summary(model74)

model75<-

glmer(landuse~scale(trans)+scale(elevation)+region_type+PA+scale(elevation):region_type+sca le(elevation):PA+(rdist|region_code3), family=binomial, data=data_cassavar_subset) summary(model75)

model76<-

glmer(landuse~scale(agri)+scale(elevation)+region_type+PA+scale(elevation):region_type+regi on_type:PA+(rdist|region_code3), family=binomial, data=data_cassavar_subset) summary(model76)

model77<-

glmer(landuse~scale(trans)+scale(elevation)+region_type+PA+scale(elevation):region_type+reg ion_type:PA+(rdist|region_code3), family=binomial, data=data_cassavar_subset) summary(model77)

###three interactions###

model78<-

glmer(landuse~scale(agri)+scale(trans)+scale(elevation)+region_type+scale(agri):region_type+s cale(elevation):region_type+scale(trans):region_type+(rdist|region_code3), family=binomial, data=data_cassavar_subset) summary(model78)

model79<-

glmer(landuse~scale(agri)+scale(trans)+region_type+PA+scale(agri):region_type+scale(agri):P A+scale(trans):region_type+(rdist|region_code3), family=binomial, data=data_cassavar_subset) summary(model79)

model80<-

glmer(landuse~scale(agri)+scale(trans)+region_type+PA+scale(agri):region_type+scale(trans):P A+scale(trans):region_type+(rdist|region_code3), family=binomial, data=data_cassavar_subset) summary(model80)

model81<-

glmer(landuse~scale(agri)+scale(trans)+region_type+PA+scale(agri):region_type+region_type:P A+scale(trans):region_type+(rdist|region_code3), family=binomial, data=data_cassavar_subset) summary(model81)

model82<-

glmer(landuse~scale(agri)+scale(elevation)+region_type+PA+scale(agri):region_type+scale(agri

):PA+scale(elevation):region_type+(rdist|region_code3), family=binomial, data=data_cassavar_subset) summary(model82)

model83<-

glmer(landuse~scale(agri)+scale(elevation)+region_type+PA+scale(agri):region_type+scale(elev ation):PA+scale(elevation):region_type+(rdist|region_code3), family=binomial, data=data_cassavar_subset) summary(model83)

model84<-

glmer(landuse~scale(agri)+scale(elevation)+region_type+PA+scale(agri):region_type+region_ty pe:PA+scale(elevation):region_type+(rdist|region_code3), family=binomial, data=data_cassavar_subset) summary(model84)

model85<-

glmer(landuse~scale(trans)+scale(elevation)+region_type+PA+scale(trans):region_type+scale(tr ans):PA+scale(elevation):region_type+(rdist|region_code3), family=binomial, data=data_cassavar_subset) summary(model85)

model86<-

glmer(landuse~scale(trans)+scale(elevation)+region_type+PA+scale(trans):region_type+scale(el evation):PA+scale(elevation):region_type+(rdist|region_code3), family=binomial, data=data_cassavar_subset) summary(model86)

model87<-

glmer(landuse~scale(trans)+scale(elevation)+region_type+PA+scale(trans):region_type+region_ type:PA+scale(elevation):region_type+(rdist|region_code3), family=binomial, data=data_cassavar_subset) summary(model87)

###four interactions### model88<-

glmer(landuse~scale(agri)+scale(trans)+region_type+PA+scale(agri):region_type+scale(trans):re gion_type+scale(agri):PA+scale(trans):PA+(rdist|region_code3), family=binomial, data=data_cassavar_subset) summary(model88)

model89<-

glmer(landuse~scale(agri)+scale(trans)+region_type+PA+scale(agri):region_type+scale(trans):re gion_type+scale(agri):PA+region_type:PA+(rdist|region_code3), family=binomial, data=data_cassavar_subset) summary(model89)

model90<-

glmer(landuse~scale(agri)+scale(trans)+region_type+PA+scale(agri):region_type+scale(trans):re gion_type+scale(trans):PA+region_type:PA+(rdist|region_code3), family=binomial, data=data_cassavar_subset) summary(model90)

model91<-

glmer(landuse~scale(agri)+scale(elevation)+region_type+PA+scale(agri):region_type+scale(elev ation):region_type+scale(agri):PA+scale(elevation):PA+(rdist|region_code3), family=binomial, data=data_cassavar_subset) summary(model91)

model92<-

glmer(landuse~scale(agri)+scale(elevation)+region_type+PA+scale(agri):region_type+scale(elev ation):region_type+scale(agri):PA+region_type:PA+(rdist|region_code3), family=binomial, data=data_cassavar_subset) summary(model92)

model93<-

glmer(landuse~scale(agri)+scale(elevation)+region_type+PA+scale(agri):region_type+scale(elev ation):region_type+region_type:PA+scale(elevation):PA+(rdist|region_code3), family=binomial, data=data_cassavar_subset) summary(model93)

model94<-

glmer(landuse~scale(trans)+scale(elevation)+region_type+PA+scale(trans):region_type+scale(el evation):region_type+scale(trans):PA+scale(elevation):PA+(rdist|region_code3), family=binomial, data=data_cassavar_subset) summary(model94)

model95<-

glmer(landuse~scale(trans)+scale(elevation)+region_type+PA+scale(trans):region_type+scale(el evation):region_type+scale(trans):PA+region_type:PA+(rdist|region_code3), family=binomial, data=data_cassavar_subset) summary(model95)

model96<-

glmer(landuse~scale(trans)+scale(elevation)+region_type+PA+scale(trans):region_type+scale(el evation):region_type+region_type:PA+scale(elevation):PA+(rdist|region_code3), family=binomial, data=data_cassavar_subset) summary(model96)

###try five interactions### model97<-

glmer(landuse~scale(agri)+scale(trans)+region_type+PA+scale(agri):region_type+scale(trans):re gion_type+scale(agri):PA+scale(trans):PA+region_type:PA+(rdist|region_code3), family=binomial, data=data_cassavar_subset) summary(model97)

model98<-

glmer(landuse~scale(agri)+scale(elevation)+region_type+PA+scale(agri):region_type+scale(elev ation):region_type+scale(agri):PA+scale(elevation):PA+region_type:PA+(rdist|region_code3), family=binomial, data=data_cassavar_subset) summary(model98)

model99<-

glmer(landuse~scale(trans)+scale(elevation)+region_type+PA+scale(trans):region_type+scale(el evation):region_type+scale(trans):PA+scale(elevation):PA+region_type:PA+(rdist|region_code3

), family=binomial, data=data_cassavar_subset) summary(model99)

##select the best model

library(MuMIn)

models_sel_cassavar<-

model.sel(model_null,model1,model2,model3,model4,model5,model6,model7, model8, model9, model10, model11,model12, model13, model14,

model15,model16,model17,model19,model20,model21,model22,model24,model28,model29, model37,model45,rank="AIC") sink(file="models_sel_cassavar.txt")

models_sel_cassavar

sink()

##diagnostic plots library(arm) binnedplot(predict(model19, type="response"), resid(model19, type="response"))

library(effects) plot(allEffects(model19)) allEffects(model19)

sink(file="alleffects(model19).txt") allEffects(model19) sink()

sink("summary(model19).txt") summary(model19)

sink()

####################prediction with actual yield ###############################

#1. Model baseline scenario, just put the actual yield in pred_cassavar <- predict(model19, data_cassavar_subset_new, re.form=NULL, type="response") head(pred_cassavar)

write.csv(pred_cassavar, "pred_cassavar.csv")

##########################prediction with potential yield ######################

##get potential yield data data_all<-read.csv("Appendix_S4_Supplementary_Data.csv",header=T)

data_cassava<-data_all[c(93253:116565),]

data_cassava_subset<-data_maize[data_cassava$land_use!=2,]

data_cassava_subset$land_use <- as.factor(data_cassava_subset$land_use) data_cassava_subset$region_code1 <- as.factor(data_cassava_subset$region_code1) data_cassava_subset$region_code2 <- as.factor(data_cassava_subset$region_code2) data_cassava_subset$region_code3 <- as.factor(data_cassava_subset$region_code3) data_cassava_subset$resid<-as.factor(data_cassava_subset$resid) data_cassava_subset$PA<- as.factor(data_cassava_subset$PA)

colnames(data_cassava_subset) <- c("landuse", "agri", "trans", "elevation",

"region_type","region_code1","region_code2","region_code3","rdist", "resid","PA","ID") head(data_cassava_subset)

#2. Scenario: actual yield being replaced by potential yield data pred_cassava <- predict(model19, data_cassava_subset, re.form=NULL, type="response") head(pred_cassava) write.csv(pred_cassava, "pred_cassava.csv")

#3. Scenario A: agr increase 50%

cassava_y <- cbind(data_cassava_subset$landuse, (data_cassava_subset$agri)*1.5 , data_cassava_subset[,3:12])

colnames(cassava_y) <- c("landuse", "agri", "trans","elevation","region_type","region_code1","region_code2","region_code3","rdist","resid","PA","ID")

summary(cassava_y)

pred_cassava<-predict(model19, cassava_y, re.form=NULL, type="response"))

write.csv(pred_cassava, "pred_cassava_agri.csv")

#4. Scenario B: transport costs increase by 35%

cassava_trans_y <- cbind(data_cassava_subset[,1:2], data_cassava_subset[,3]*1.35, data_cassava_subset[,4:12]) colnames(cassava_trans_y) <- c("landuse", "agri",

"trans","elevation","region_type","region_code1","region_code2","region_code3","rdist","resid",

"PA","ID") summary(cassava_trans_y)

pred_trans_cassava<-predict(model19, cassava_trans_y, re.form=NULL, type="response")

write.csv(pred_trans_cassava, "pred_cassava_trans.csv")

#5. Scenario C: political stability has total conversion

region_cassava <- data_cassava_subset

summary(region_cassava) region_cassava[,5] <- "Division"

region_cassava[,5] <- as.factor(region_cassava[,5])

pred_region_cassava <- predict(model19, region_cassava, re.form=NULL, type="response") head(pred_region_cassava)

write.csv(pred_region_cassava, "pred_cassava_stability.csv")

#6. Scenario D: worst case. agri increase 50%, and stability has total conversion cassava_worstcase<-cassava_y

cassava_worstcase[,5]<-"Division"

pred_worstcase_cassava<-predict(model19, cassava_worstcase, re.form=NULL, type="response")

write.csv(pred_worstcase_cassava, "pred_cassava_worstcase.csv")

rm(list=ls())

##############################################################################

##################model land use with characteristics of potato########################

setwd("D:\\Myanmar project\\Resubmission 2\\Code and data (submit)")

data_all_raman<-read.csv("Appendix_S3_Supplementary_Data.csv",header=T) data_potator<-data_all_raman[c(116566:139878),]

data_potator_subset<-data_potator[data_potator$land_use!=2,]

#convert to factors data_potator_subset$land_use <- as.factor(data_potator_subset$land_use) data_potator_subset$region_code1 <- as.factor(data_potator_subset$region_code1) data_potator_subset$region_code2 <- as.factor(data_potator_subset$region_code2) data_potator_subset$region_code3 <- as.factor(data_potator_subset$region_code3) data_potator_subset$resid<-as.factor(data_potator_subset$resid) data_potator_subset$PA<- as.factor(data_potator_subset$PA)

colnames(data_potator_subset) <- c("landuse", "agri", "trans", "elevation",

"region_type","region_code1","region_code2","region_code3","rdist", "resid","PA","ID") head(data_potator_subset)

library(lme4)

##check collinearity

library(car)

model_vif <- glm(landuse ~ scale(agri)+scale(trans)+scale(elevation) +region_type+PA, family=binomial, data=data_potator_subset) summary(model_vif)

vif(model_vif)

###try to fix the random effects first

model_r1 <- glmer(landuse ~ scale(agri)+scale(trans)+scale(elevation) +region_type+PA+

(1|region_code1), family=binomial, data=data_potator_subset) summary(model_r1)

model_r2 <- glmer(landuse ~ scale(agri)+scale(trans) +scale(elevation)+region_type+PA+

(1|region_code2), family=binomial, data=data_potator_subset) summary(model_r2)

model_r3 <- glmer(landuse ~ scale(agri)+scale(trans)+scale(elevation) +region_type+PA+

(1|region_code3), family=binomial, data=data_potator_subset) summary(model_r3)

AIC(model_r1, model_r2, model_r3)

#choose region_code3 with smallest AIC

model_r4 <- glmer(landuse ~ scale(agri)+scale(trans)+scale(elevation) +region_type+PA+

(rdist|region_code3), family=binomial, data=data_potator_subset) summary(model_r4)

##choose model_r4 with samllest AIC ################################################################# ###one variable###

model_null<-glmer(landuse~1+(rdist|region_code3), family=binomial, data=data_potator_subset) summary(model_null)

model1<-glmer(landuse~scale(agri)+(rdist|region_code3), family=binomial, data=data_potator_subset) summary(model1)

model2<-glmer(landuse~scale(trans)+(rdist|region_code3), family=binomial, data=data_potator_subset) summary(model2)

model3<-glmer(landuse~scale(elevation)+(rdist|region_code3), family=binomial, data=data_potator_subset) summary(model3)

model4<-glmer(landuse~region_type+(rdist|region_code3), family=binomial, data=data_potator_subset) summary(model4)

model5<-glmer(landuse~PA+(rdist|region_code3), family=binomial, data=data_potator_subset) summary(model5)

###two variables###

model6<-glmer(landuse~scale(agri)+scale(trans)+(rdist|region_code3), family=binomial, data=data_potator_subset) summary(model6)

model7<-glmer(landuse~scale(agri)+scale(elevation)+(rdist|region_code3), family=binomial, data=data_potator_subset) summary(model7)

model8<-glmer(landuse~scale(agri)+region_type+(rdist|region_code3), family=binomial, data=data_potator_subset) summary(model8)

model9<-glmer(landuse~scale(trans)+region_type+(rdist|region_code3), family=binomial, data=data_potator_subset) summary(model9)

model10<-glmer(landuse~scale(elevation)+region_type+(rdist|region_code3), family=binomial, data=data_potator_subset) summary(model10)

model11<-glmer(landuse~scale(trans)+scale(elevation)+(rdist|region_code3), family=binomial, data=data_potator_subset) summary(model11)

model12<-glmer(landuse~scale(agri)+PA+(rdist|region_code3), family=binomial, data=data_potator_subset) summary(model12)

model13<-glmer(landuse~scale(trans)+PA+(rdist|region_code3), family=binomial, data=data_potator_subset) summary(model13) model14<-glmer(landuse~scale(elevation)+PA+(rdist|region_code3), family=binomial, data=data_potator_subset) summary(model14)

model15<-glmer(landuse~region_type+PA+(rdist|region_code3), family=binomial, data=data_potator_subset) summary(model15)

###three variables###

model16<-glmer(landuse~scale(agri)+scale(trans)+region_type+(rdist|region_code3), family=binomial, data=data_potator_subset) summary(model16)

model17<-glmer(landuse~scale(agri)+scale(elevation)+region_type+(rdist|region_code3), family=binomial, data=data_potator_subset) summary(model17)

model18<-glmer(landuse~scale(agri)+scale(trans)+scale(elevation)+(rdist|region_code3), family=binomial, data=data_potator_subset) summary(model18)

model19<-glmer(landuse~scale(trans)+scale(elevation)+region_type+(rdist|region_code3), family=binomial, data=data_potator_subset) summary(model19)

model20<-glmer(landuse~scale(agri)+scale(trans)+PA+(rdist|region_code3), family=binomial, data=data_potator_subset)

summary(model20)

model21<-glmer(landuse~scale(agri)+scale(elevation)+PA+(rdist|region_code3), family=binomial, data=data_potator_subset) summary(model21)

model22<-glmer(landuse~scale(agri)+region_type+PA+(rdist|region_code3), family=binomial, data=data_potator_subset) summary(model22)

model23<-glmer(landuse~scale(trans)+scale(elevation)+PA+(rdist|region_code3), family=binomial, data=data_potator_subset) summary(model23)

model24<-glmer(landuse~scale(trans)+region_type+PA+(rdist|region_code3), family=binomial, data=data_potator_subset) summary(model24)

model25<-glmer(landuse~scale(elevation)+region_type+PA+(rdist|region_code3), family=binomial, data=data_potator_subset) summary(model25)

###four variables###

model26<-

glmer(landuse~scale(agri)+scale(trans)+scale(elevation)+region_type+(rdist|region_code3), family=binomial, data=data_potator_subset) summary(model26)

model27<-glmer(landuse~scale(agri)+scale(trans)+scale(elevation)+PA+(rdist|region_code3), family=binomial, data=data_potator_subset)

summary(model27)

model28<-glmer(landuse~scale(agri)+scale(trans)+PA+region_type+(rdist|region_code3), family=binomial, data=data_potator_subset) summary(model28)

model29<-glmer(landuse~scale(agri)+PA+scale(elevation)+region_type+(rdist|region_code3), family=binomial, data=data_potator_subset) summary(model29)

model30<-glmer(landuse~PA+scale(trans)+scale(elevation)+region_type+(rdist|region_code3), family=binomial, data=data_potator_subset) summary(model30)

###five variables###

model31<-glmer(landuse ~ scale(agri)+scale(trans)+scale(elevation)

+region_type+PA+(rdist|region_code3), family=binomial, data=data_potator_subset)

###one interaction###

model32<-

glmer(landuse~scale(agri)+scale(trans)+scale(elevation)+region_type+scale(agri):region_type+(r dist|region_code3), family=binomial, data=data_potator_subset) summary(model32)

model33<-

glmer(landuse~scale(agri)+scale(trans)+scale(elevation)+region_type+scale(trans):region_type+

(rdist|region_code3), family=binomial, data=data_potator_subset) summary(model33)

model34<-

glmer(landuse~scale(agri)+scale(trans)+scale(elevation)+region_type+scale(elevation):region_ty pe+(rdist|region_code3), family=binomial, data=data_potator_subset) summary(model34)

model35<-

glmer(landuse~scale(agri)+scale(trans)+scale(elevation)+PA+scale(agri):PA+(rdist|region_code

3), family=binomial, data=data_potator_subset) summary(model35)

model36<-

glmer(landuse~scale(agri)+scale(trans)+region_type+PA+scale(agri):PA+(rdist|region_code3), family=binomial, data=data_potator_subset) summary(model36)

model37<-

glmer(landuse~scale(agri)+scale(elevation)+region_type+PA+scale(agri):PA+(rdist|region_code

3), family=binomial, data=data_potator_subset) summary(model37)

model38<-

glmer(landuse~scale(agri)+scale(trans)+scale(elevation)+PA+scale(trans):PA+(rdist|region_code

3), family=binomial, data=data_potator_subset) summary(model38)

model39<-

glmer(landuse~scale(agri)+scale(trans)+region_type+PA+scale(trans):PA+(rdist|region_code3), family=binomial, data=data_potator_subset) summary(model39)

model40<-

glmer(landuse~scale(trans)+scale(elevation)+region_type+PA+scale(trans):PA+(rdist|region_co de3), family=binomial, data=data_potator_subset) summary(model40)

model41<-

glmer(landuse~scale(agri)+scale(trans)+scale(elevation)+PA+scale(elevation):PA+(rdist|region_ code3), family=binomial, data=data_potator_subset) summary(model41)

model42<-

glmer(landuse~scale(agri)+scale(elevation)+region_type+PA+scale(elevation):PA+(rdist|region_ code3), family=binomial, data=data_potator_subset) summary(model42)

model43<-

glmer(landuse~scale(trans)+scale(elevation)+region_type+PA+scale(elevation):PA+(rdist|region

_code3), family=binomial, data=data_potator_subset) summary(model43)

model44<-

glmer(landuse~scale(agri)+scale(trans)+region_type+PA+region_type:PA+(rdist|region_code3), family=binomial, data=data_potator_subset) summary(model44)

model45<-

glmer(landuse~scale(agri)+scale(elevation)+region_type+PA+region_type:PA+(rdist|region_cod e3), family=binomial, data=data_potator_subset) summary(model45)

model46<-

glmer(landuse~scale(trans)+scale(elevation)+region_type+PA+region_type:PA+(rdist|region_co de3), family=binomial, data=data_potator_subset) summary(model46)

###two interactions###

## with region

model47<-

glmer(landuse~scale(agri)+scale(trans)+scale(elevation)+region_type+scale(agri):region_type+s cale(trans):region_type+(rdist|region_code3), family=binomial, data=data_potator_subset) summary(model47)

model48<-

glmer(landuse~scale(agri)+scale(trans)+scale(elevation)+region_type+scale(agri):region_type+s cale(elevation):region_type+(rdist|region_code3), family=binomial, data=data_potator_subset) summary(model48)

model49<-

glmer(landuse~scale(agri)+scale(trans)+scale(elevation)+region_type+scale(trans):region_type+ scale(elevation):region_type+(rdist|region_code3), family=binomial, data=data_potator_subset) summary(model49)

##with PA model50<-

glmer(landuse~scale(agri)+scale(trans)+scale(elevation)+PA+scale(agri):PA+scale(trans):PA+(r dist|region_code3), family=binomial, data=data_potator_subset) summary(model50)

model51<-

glmer(landuse~scale(agri)+scale(trans)+region_type+PA+scale(agri):PA+scale(trans):PA+(rdist| region_code3), family=binomial, data=data_potator_subset)

summary(model51)

model52<-

glmer(landuse~scale(agri)+scale(trans)+scale(elevation)+PA+scale(agri):PA+scale(elevation):P

A+(rdist|region_code3), family=binomial, data=data_potator_subset) summary(model52)

model53<-

glmer(landuse~scale(agri)+scale(elevation)+region_type+PA+scale(agri):PA+scale(elevation):P

A+(rdist|region_code3), family=binomial, data=data_potator_subset) summary(model53)

model54<-

glmer(landuse~scale(agri)+scale(trans)+region_type+PA+scale(agri):PA+region_type:PA+(rdist| region_code3), family=binomial, data=data_potator_subset) summary(model54)

model55<-

glmer(landuse~scale(agri)+scale(elevation)+region_type+PA+scale(agri):PA+region_type:PA+( rdist|region_code3), family=binomial, data=data_potator_subset) summary(model55)

model56<-

glmer(landuse~scale(agri)+scale(trans)+scale(elevation)+PA+scale(trans):PA+scale(elevation):P

A+(rdist|region_code3), family=binomial, data=data_potator_subset) summary(model56)

model57<glmer(landuse~scale(trans)+scale(elevation)+region_type+PA+scale(trans):PA+scale(elevation): PA+(rdist|region_code3), family=binomial, data=data_potator_subset) summary(model57)

model58<-

glmer(landuse~scale(agri)+scale(trans)+region_type+PA+scale(trans):PA+region_type:PA+(rdis t|region_code3), family=binomial, data=data_potator_subset) summary(model58)

model59<-

glmer(landuse~scale(trans)+scale(elevation)+region_type+PA+scale(trans):PA+region_type:PA

+(rdist|region_code3), family=binomial, data=data_potator_subset) summary(model59)

##combine two sets model60<-

glmer(landuse~scale(agri)+scale(trans)+region_type+PA+scale(agri):region_type+scale(agri):P

A+(rdist|region_code3), family=binomial, data=data_potator_subset) summary(model60)

model61<-

glmer(landuse~scale(agri)+scale(elevation)+region_type+PA+scale(agri):region_type+scale(agri

):PA+(rdist|region_code3), family=binomial, data=data_potator_subset) summary(model61)

model62<-

glmer(landuse~scale(agri)+scale(trans)+region_type+PA+scale(agri):region_type+scale(trans):P A+(rdist|region_code3), family=binomial, data=data_potator_subset) summary(model62)

model63<-

glmer(landuse~scale(agri)+scale(elevation)+region_type+PA+scale(agri):region_type+scale(elev ation):PA+(rdist|region_code3), family=binomial, data=data_potator_subset) summary(model63)

model64<-

glmer(landuse~scale(agri)+scale(trans)+region_type+PA+scale(agri):region_type+region_type:P

A+(rdist|region_code3), family=binomial, data=data_potator_subset) summary(model64)

model65<-

glmer(landuse~scale(agri)+scale(elevation)+region_type+PA+scale(agri):region_type+region_ty pe:PA+(rdist|region_code3), family=binomial, data=data_potator_subset) summary(model65)

model66<-

glmer(landuse~scale(agri)+scale(trans)+region_type+PA+scale(trans):region_type+scale(agri):P

A+(rdist|region_code3), family=binomial, data=data_potator_subset) summary(model66)

model67<glmer(landuse~scale(agri)+scale(trans)+region_type+PA+scale(trans):region_type+scale(trans): PA+(rdist|region_code3), family=binomial, data=data_potator_subset) summary(model67)

model68<-

glmer(landuse~scale(elevation)+scale(trans)+region_type+PA+scale(trans):region_type+scale(tr ans):PA+(rdist|region_code3), family=binomial, data=data_potator_subset) summary(model68)

model69<-

glmer(landuse~scale(trans)+scale(elevation)+region_type+PA+scale(trans):region_type+scale(el evation):PA+(rdist|region_code3), family=binomial, data=data_potator_subset) summary(model69)

model70<glmer(landuse~scale(agri)+scale(trans)+region_type+PA+scale(trans):region_type+region_type:

PA+(rdist|region_code3), family=binomial, data=data_potator_subset)

summary(model70)

model71<-

glmer(landuse~scale(trans)+scale(elevation)+region_type+PA+scale(trans):region_type+region_ type:PA+(rdist|region_code3), family=binomial, data=data_potator_subset) summary(model71)

model72<-

glmer(landuse~scale(agri)+scale(elevation)+region_type+PA+scale(elevation):region_type+scal e(agri):PA+(rdist|region_code3), family=binomial, data=data_potator_subset) summary(model72)

model73<-

glmer(landuse~scale(trans)+scale(elevation)+region_type+PA+scale(elevation):region_type+sca le(trans):PA+(rdist|region_code3), family=binomial, data=data_potator_subset) summary(model73)

model74<-

glmer(landuse~scale(agri)+scale(elevation)+region_type+PA+scale(elevation):region_type+scal e(elevation):PA+(rdist|region_code3), family=binomial, data=data_potator_subset) summary(model74)

model75<-

glmer(landuse~scale(trans)+scale(elevation)+region_type+PA+scale(elevation):region_type+sca le(elevation):PA+(rdist|region_code3), family=binomial, data=data_potator_subset) summary(model75)

model76<-

glmer(landuse~scale(agri)+scale(elevation)+region_type+PA+scale(elevation):region_type+regi on_type:PA+(rdist|region_code3), family=binomial, data=data_potator_subset) summary(model76)

model77<-

glmer(landuse~scale(trans)+scale(elevation)+region_type+PA+scale(elevation):region_type+reg ion_type:PA+(rdist|region_code3), family=binomial, data=data_potator_subset) summary(model77)

###three interactions###

model78<-

glmer(landuse~scale(agri)+scale(trans)+scale(elevation)+region_type+scale(agri):region_type+s cale(elevation):region_type+scale(trans):region_type+(rdist|region_code3), family=binomial, data=data_potator_subset) summary(model78)

model79<-

glmer(landuse~scale(agri)+scale(trans)+region_type+PA+scale(agri):region_type+scale(agri):P A+scale(trans):region_type+(rdist|region_code3), family=binomial, data=data_potator_subset) summary(model79)

model80<-

glmer(landuse~scale(agri)+scale(trans)+region_type+PA+scale(agri):region_type+scale(trans):P A+scale(trans):region_type+(rdist|region_code3), family=binomial, data=data_potator_subset) summary(model80)

model81<-

glmer(landuse~scale(agri)+scale(trans)+region_type+PA+scale(agri):region_type+region_type:P A+scale(trans):region_type+(rdist|region_code3), family=binomial, data=data_potator_subset) summary(model81)

model82<-

glmer(landuse~scale(agri)+scale(elevation)+region_type+PA+scale(agri):region_type+scale(agri

):PA+scale(elevation):region_type+(rdist|region_code3), family=binomial, data=data_potator_subset) summary(model82)

model83<-

glmer(landuse~scale(agri)+scale(elevation)+region_type+PA+scale(agri):region_type+scale(elev ation):PA+scale(elevation):region_type+(rdist|region_code3), family=binomial, data=data_potator_subset) summary(model83)

model84<-

glmer(landuse~scale(agri)+scale(elevation)+region_type+PA+scale(agri):region_type+region_ty pe:PA+scale(elevation):region_type+(rdist|region_code3), family=binomial, data=data_potator_subset) summary(model84)

model85<-

glmer(landuse~scale(trans)+scale(elevation)+region_type+PA+scale(trans):region_type+scale(tr ans):PA+scale(elevation):region_type+(rdist|region_code3), family=binomial, data=data_potator_subset) summary(model85)

model86<-

glmer(landuse~scale(trans)+scale(elevation)+region_type+PA+scale(trans):region_type+scale(el evation):PA+scale(elevation):region_type+(rdist|region_code3), family=binomial, data=data_potator_subset) summary(model86)

model87<-

glmer(landuse~scale(trans)+scale(elevation)+region_type+PA+scale(trans):region_type+region_ type:PA+scale(elevation):region_type+(rdist|region_code3), family=binomial, data=data_potator_subset) summary(model87)

###four interactions### model88<-

glmer(landuse~scale(agri)+scale(trans)+region_type+PA+scale(agri):region_type+scale(trans):re gion_type+scale(agri):PA+scale(trans):PA+(rdist|region_code3), family=binomial, data=data_potator_subset) summary(model88)

model89<-

glmer(landuse~scale(agri)+scale(trans)+region_type+PA+scale(agri):region_type+scale(trans):re gion_type+scale(agri):PA+region_type:PA+(rdist|region_code3), family=binomial, data=data_potator_subset) summary(model89)

model90<-

glmer(landuse~scale(agri)+scale(trans)+region_type+PA+scale(agri):region_type+scale(trans):re gion_type+scale(trans):PA+region_type:PA+(rdist|region_code3), family=binomial, data=data_potator_subset) summary(model90)

model91<-

glmer(landuse~scale(agri)+scale(elevation)+region_type+PA+scale(agri):region_type+scale(elev ation):region_type+scale(agri):PA+scale(elevation):PA+(rdist|region_code3), family=binomial, data=data_potator_subset) summary(model91)

model92<-

glmer(landuse~scale(agri)+scale(elevation)+region_type+PA+scale(agri):region_type+scale(elev ation):region_type+scale(agri):PA+region_type:PA+(rdist|region_code3), family=binomial, data=data_potator_subset) summary(model92)

model93<-

glmer(landuse~scale(agri)+scale(elevation)+region_type+PA+scale(agri):region_type+scale(elev ation):region_type+region_type:PA+scale(elevation):PA+(rdist|region_code3), family=binomial, data=data_potator_subset) summary(model93)

model94<-

glmer(landuse~scale(trans)+scale(elevation)+region_type+PA+scale(trans):region_type+scale(el evation):region_type+scale(trans):PA+scale(elevation):PA+(rdist|region_code3), family=binomial, data=data_potator_subset) summary(model94)

model95<-

glmer(landuse~scale(trans)+scale(elevation)+region_type+PA+scale(trans):region_type+scale(el evation):region_type+scale(trans):PA+region_type:PA+(rdist|region_code3), family=binomial, data=data_potator_subset) summary(model95)

model96<-

glmer(landuse~scale(trans)+scale(elevation)+region_type+PA+scale(trans):region_type+scale(el evation):region_type+region_type:PA+scale(elevation):PA+(rdist|region_code3), family=binomial, data=data_potator_subset) summary(model96)

###try five interactions### model97<-

glmer(landuse~scale(agri)+scale(trans)+region_type+PA+scale(agri):region_type+scale(trans):re gion_type+scale(agri):PA+scale(trans):PA+region_type:PA+(rdist|region_code3), family=binomial, data=data_potator_subset) summary(model97)

model98<-

glmer(landuse~scale(agri)+scale(elevation)+region_type+PA+scale(agri):region_type+scale(elev ation):region_type+scale(agri):PA+scale(elevation):PA+region_type:PA+(rdist|region_code3), family=binomial, data=data_potator_subset) summary(model98)

model99<-

glmer(landuse~scale(trans)+scale(elevation)+region_type+PA+scale(trans):region_type+scale(el evation):region_type+scale(trans):PA+scale(elevation):PA+region_type:PA+(rdist|region_code3

), family=binomial, data=data_potator_subset) summary(model99)

##select the best model library(MuMIn)

models_sel_potator<-

model.sel(model_null,model1,model2,model3,model4,model5,model6,model7, model8, model9, model10, model11,model12, model13, model14,

model15,model16,model17,model18,model20,model21,model22,model24,model28,model29, model30,model40,model41,model52,rank="AIC") sink(file="models_sel_potator.txt")

models_sel_potator

sink()

##dignostic plot library(arm) binnedplot(predict(model41, type="response"), resid(model41, type="response"))

library(effects) plot(allEffects(model41)) allEffects(model41)

sink(file="alleffects(model41).txt") allEffects(model41)

sink()

sink("summary(model41).txt") summary(model41)

sink()

library(arm) binnedplot(predict(model52, type="response"), resid(model52, type="response"))

library(effects) plot(allEffects(model52)) allEffects(model52)

sink(file="alleffects(model52).txt") allEffects(model52) sink()

sink("summary(model52).txt") summary(model52)

sink()

##model average mod.avg <- summary(model.avg(models_sel_potator,subset=delta <2,fit=TRUE)) sink("model_average.txt") mod.avg sink()

avg<-model.avg(models_sel_potator,subset=delta <2,fit=TRUE)

####################prediction with actual yield ###############################

#1. Model baseline scenario, just put the actual yield in pred_potator <- predict(avg, data_potator_subset, re.form=NULL, type="response") head(pred_potator)

write.csv(pred_potator, "pred_potator.csv")

##########################prediction with potential yield ######################

##get potential yield data data_all<-read.csv("Appendix_S4_Supplementary_Data.csv",header=T)

data_potato<-data_all[c(116566:139878),]

data_potato_subset<-data_potato[data_potato$land_use!=2,]

data_potato_subset$land_use <- as.factor(data_potato_subset$land_use) data_potato_subset$region_code1 <- as.factor(data_potato_subset$region_code1) data_potato_subset$region_code2 <- as.factor(data_potato_subset$region_code2) data_potato_subset$region_code3 <- as.factor(data_potato_subset$region_code3) data_potato_subset$resid<-as.factor(data_potato_subset$resid) data_potato_subset$PA<- as.factor(data_potato_subset$PA)

colnames(data_potato_subset) <- c("landuse", "agri", "trans", "elevation",

"region_type","region_code1","region_code2","region_code3","rdist", "resid","PA","ID") head(data_potato_subset)

#2. Scenario: actual yield being replaced by potential yield data pred_potato <- predict(avg, data_potato_subset, re.form=NULL, type="response") head(pred_potato)

write.csv(pred_potato, "pred_potato.csv")

#3. Scenario A: agr increase 50%

potato_y <- cbind(data_potato_subset$landuse, (data_potato_subset$agri)*1.5 , data_potato_subset[,3:12])

colnames(potato_y) <- c("landuse", "agri", "trans","elevation","region_type","region_code1","region_code2","region_code3","rdist","resid","PA","ID")

summary(potato_y)

pred_potato<-predict(avg, potato_y, re.form=NULL, type="response"))

write.csv(pred_potato, "pred_potato_agri.csv")

#4. Scenario B: transport costs increase by 35%

potato_trans_y <- cbind(data_potato_subset[,1:2], data_potato_subset[,3]*1.35, data_potato_subset[,4:12]) colnames(potato_trans_y) <- c("landuse", "agri",

"trans","elevation","region_type","region_code1","region_code2","region_code3","rdist","resid",

"PA","ID") summary(potato_trans_y)

pred_trans_potato<-predict(avg, potato_trans_y, re.form=NULL, type="response")

write.csv(pred_trans_potato, "pred_potato_trans.csv")

#5. Scenario C: political stability has total conversion

region_potato <- data_potato_subset

summary(region_potato) region_potato[,5] <- "Division"

region_potato[,5] <- as.factor(region_potato[,5])

pred_region_potato <- predict(avg, region_potato, re.form=NULL, type="response") head(pred_region_potato)

write.csv(pred_region_potato, "pred_potato_stability.csv")

#6. Scenario D: worst case. agri increase 50%, and stability has total conversion potato_worstcase<-potato_y

potato_worstcase[,5]<-"Division"

pred_worstcase_potato<-predict(avg, potato_worstcase, re.form=NULL, type="response")

write.csv(pred_worstcase_potato, "pred_potato_worstcase.csv")

rm(list=ls())

##############################################################################

###################model land use with characteristics of rubber#######################

setwd("D:\\Myanmar project\\Resubmission 2\\Code and data (submit)")

data_all_raman<-read.csv("Appendix_S3_Supplementary_Data.csv",header=T)

data_rubber<-data_all_raman[c(139879:163191),]

data_rubber_subset<-data_rubber[data_rubber$land_use!=2,]

#convert to factors data_rubber_subset$land_use <- as.factor(data_rubber_subset$land_use) data_rubber_subset$region_code1 <- as.factor(data_rubber_subset$region_code1) data_rubber_subset$region_code2 <- as.factor(data_rubber_subset$region_code2) data_rubber_subset$region_code3 <- as.factor(data_rubber_subset$region_code3) data_rubber_subset$resid<-as.factor(data_rubber_subset$resid) data_rubber_subset$PA<- as.factor(data_rubber_subset$PA)

colnames(data_rubber_subset) <- c("landuse", "agri", "trans", "elevation",

"region_type","region_code1","region_code2","region_code3","rdist", "resid","PA","ID") head(data_rubber_subset)

library(lme4)

##check collinearity

library(car)

model_vif <- glm(landuse ~ scale(agri)+scale(trans)+scale(elevation) +region_type+PA, family=binomial, data=data_rubberr_subset) summary(model_vif)

vif(model_vif)

###try to fix the random effects first

model_r1 <- glmer(landuse ~ scale(agri)+scale(trans)+scale(elevation) +region_type+PA+

(1|region_code1), family=binomial, data=data_rubber_subset) summary(model_r1)

model_r2 <- glmer(landuse ~ scale(agri)+scale(trans) +scale(elevation)+region_type+PA+

(1|region_code2), family=binomial, data=data_rubber_subset) summary(model_r2)

model_r3 <- glmer(landuse ~ scale(agri)+scale(trans)+scale(elevation) +region_type+PA+

(1|region_code3), family=binomial, data=data_rubber_subset) summary(model_r3)

AIC(model_r1, model_r2, model_r3)

#choose region_code3 with smallest AIC

model_r4 <- glmer(landuse ~ scale(agri)+scale(trans)+scale(elevation) +region_type+PA+

(rdist|region_code3), family=binomial, data=data_rubber_subset) summary(model_r4)

AIC(model_r3,model_r4)

############################################################################## ######

###one variable### model_null<-glmer(landuse~1+(rdist|region_code3), family=binomial, data=data_rubber_subset) summary(model_null)

model1<-glmer(landuse~scale(agri)+(rdist|region_code3), family=binomial, data=data_rubber_subset) summary(model1)

model2<-glmer(landuse~scale(trans)+(rdist|region_code3), family=binomial, data=data_rubber_subset) summary(model2) model3<-glmer(landuse~scale(elevation)+(rdist|region_code3), family=binomial, data=data_rubber_subset) summary(model3)

model4<-glmer(landuse~region_type+(rdist|region_code3), family=binomial, data=data_rubber_subset) summary(model4)

model5<-glmer(landuse~PA+(rdist|region_code3), family=binomial, data=data_rubber_subset) summary(model5)

###two variables###

model6<-glmer(landuse~scale(agri)+scale(trans)+(rdist|region_code3), family=binomial, data=data_rubber_subset) summary(model6)

model7<-glmer(landuse~scale(agri)+scale(elevation)+(rdist|region_code3), family=binomial, data=data_rubber_subset) summary(model7)

model8<-glmer(landuse~scale(agri)+region_type+(rdist|region_code3), family=binomial, data=data_rubber_subset) summary(model8)

model9<-glmer(landuse~scale(trans)+region_type+(rdist|region_code3), family=binomial, data=data_rubber_subset) summary(model9) model10<-glmer(landuse~scale(elevation)+region_type+(rdist|region_code3), family=binomial, data=data_rubber_subset) summary(model10)

model11<-glmer(landuse~scale(trans)+scale(elevation)+(rdist|region_code3), family=binomial, data=data_rubber_subset) summary(model11)

model12<-glmer(landuse~scale(agri)+PA+(rdist|region_code3), family=binomial, data=data_rubber_subset) summary(model12)

model13<-glmer(landuse~scale(trans)+PA+(rdist|region_code3), family=binomial, data=data_rubber_subset) summary(model13)

model14<-glmer(landuse~scale(elevation)+PA+(rdist|region_code3), family=binomial, data=data_rubber_subset) summary(model14)

model15<-glmer(landuse~region_type+PA+(rdist|region_code3), family=binomial, data=data_rubber_subset) summary(model15)

###three variables###

model16<-glmer(landuse~scale(agri)+scale(trans)+region_type+(rdist|region_code3), family=binomial, data=data_rubber_subset) summary(model16) model17<-glmer(landuse~scale(agri)+scale(elevation)+region_type+(rdist|region_code3), family=binomial, data=data_rubber_subset) summary(model17)

model18<-glmer(landuse~scale(agri)+scale(trans)+scale(elevation)+(rdist|region_code3), family=binomial, data=data_rubber_subset) summary(model18)

model19<-glmer(landuse~scale(trans)+scale(elevation)+region_type+(rdist|region_code3), family=binomial, data=data_rubber_subset) summary(model19)

model20<-glmer(landuse~scale(agri)+scale(trans)+PA+(rdist|region_code3), family=binomial, data=data_rubber_subset) summary(model20)

model21<-glmer(landuse~scale(agri)+scale(elevation)+PA+(rdist|region_code3), family=binomial, data=data_rubber_subset) summary(model21)

model22<-glmer(landuse~scale(agri)+region_type+PA+(rdist|region_code3), family=binomial, data=data_rubber_subset) summary(model22)

model23<-glmer(landuse~scale(trans)+scale(elevation)+PA+(rdist|region_code3), family=binomial, data=data_rubber_subset) summary(model23) model24<-glmer(landuse~scale(trans)+region_type+PA+(rdist|region_code3), family=binomial, data=data_rubber_subset) summary(model24)

model25<-glmer(landuse~scale(elevation)+region_type+PA+(rdist|region_code3), family=binomial, data=data_rubber_subset) summary(model25)

###four variables###

model26<-

glmer(landuse~scale(agri)+scale(trans)+scale(elevation)+region_type+(rdist|region_code3), family=binomial, data=data_rubber_subset) summary(model26)

model27<-glmer(landuse~scale(agri)+scale(trans)+scale(elevation)+PA+(rdist|region_code3), family=binomial, data=data_rubber_subset) summary(model27)

model28<-glmer(landuse~scale(agri)+scale(trans)+PA+region_type+(rdist|region_code3), family=binomial, data=data_rubber_subset) summary(model28)

model29<-glmer(landuse~scale(agri)+PA+scale(elevation)+region_type+(rdist|region_code3), family=binomial, data=data_rubber_subset) summary(model29)

model30<-glmer(landuse~PA+scale(trans)+scale(elevation)+region_type+(rdist|region_code3), family=binomial, data=data_rubber_subset) summary(model30)

###five variables###

model31<-glmer(landuse ~ scale(agri)+scale(trans)+scale(elevation)

+region_type+PA+(rdist|region_code3), family=binomial, data=data_rubber_subset)

###one interaction###

model32<-

glmer(landuse~scale(agri)+scale(trans)+scale(elevation)+region_type+scale(agri):region_type+(r dist|region_code3), family=binomial, data=data_rubber_subset) summary(model32)

model33<-

glmer(landuse~scale(agri)+scale(trans)+scale(elevation)+region_type+scale(trans):region_type+

(rdist|region_code3), family=binomial, data=data_rubber_subset) summary(model33)

model34<-

glmer(landuse~scale(agri)+scale(trans)+scale(elevation)+region_type+scale(elevation):region_ty pe+(rdist|region_code3), family=binomial, data=data_rubber_subset) summary(model34)

model35<-

glmer(landuse~scale(agri)+scale(trans)+scale(elevation)+PA+scale(agri):PA+(rdist|region_code

3), family=binomial, data=data_rubber_subset) summary(model35)

model36<-

glmer(landuse~scale(agri)+scale(trans)+region_type+PA+scale(agri):PA+(rdist|region_code3), family=binomial, data=data_rubber_subset) summary(model36)

model37<-

glmer(landuse~scale(agri)+scale(elevation)+region_type+PA+scale(agri):PA+(rdist|region_code

3), family=binomial, data=data_rubber_subset) summary(model37)

model38<-

glmer(landuse~scale(agri)+scale(trans)+scale(elevation)+PA+scale(trans):PA+(rdist|region_code

3), family=binomial, data=data_rubber_subset) summary(model38)

model39<-

glmer(landuse~scale(agri)+scale(trans)+region_type+PA+scale(trans):PA+(rdist|region_code3), family=binomial, data=data_rubber_subset) summary(model39)

model40<-

glmer(landuse~scale(trans)+scale(elevation)+region_type+PA+scale(trans):PA+(rdist|region_co de3), family=binomial, data=data_rubber_subset) summary(model40)

model41<-

glmer(landuse~scale(agri)+scale(trans)+scale(elevation)+PA+scale(elevation):PA+(rdist|region_ code3), family=binomial, data=data_rubber_subset) summary(model41)

model42<-

glmer(landuse~scale(agri)+scale(elevation)+region_type+PA+scale(elevation):PA+(rdist|region_ code3), family=binomial, data=data_rubber_subset) summary(model42)

model43<-

glmer(landuse~scale(trans)+scale(elevation)+region_type+PA+scale(elevation):PA+(rdist|region

_code3), family=binomial, data=data_rubber_subset) summary(model43)

model44<-

glmer(landuse~scale(agri)+scale(trans)+region_type+PA+region_type:PA+(rdist|region_code3), family=binomial, data=data_rubber_subset) summary(model44)

model45<-

glmer(landuse~scale(agri)+scale(elevation)+region_type+PA+region_type:PA+(rdist|region_cod e3), family=binomial, data=data_rubber_subset) summary(model45)

model46<-

glmer(landuse~scale(trans)+scale(elevation)+region_type+PA+region_type:PA+(rdist|region_co de3), family=binomial, data=data_rubber_subset) summary(model46)

###two interactions###

## with region

model47<-

glmer(landuse~scale(agri)+scale(trans)+scale(elevation)+region_type+scale(agri):region_type+s cale(trans):region_type+(rdist|region_code3), family=binomial, data=data_rubber_subset) summary(model47)

model48<-

glmer(landuse~scale(agri)+scale(trans)+scale(elevation)+region_type+scale(agri):region_type+s cale(elevation):region_type+(rdist|region_code3), family=binomial, data=data_rubber_subset) summary(model48)

model49<-

glmer(landuse~scale(agri)+scale(trans)+scale(elevation)+region_type+scale(trans):region_type+ scale(elevation):region_type+(rdist|region_code3), family=binomial, data=data_rubber_subset) summary(model49)

##with PA model50<-

glmer(landuse~scale(agri)+scale(trans)+scale(elevation)+PA+scale(agri):PA+scale(trans):PA+(r dist|region_code3), family=binomial, data=data_rubber_subset) summary(model50)

model51<-

glmer(landuse~scale(agri)+scale(trans)+region_type+PA+scale(agri):PA+scale(trans):PA+(rdist| region_code3), family=binomial, data=data_rubber_subset) summary(model51)

model52<-

glmer(landuse~scale(agri)+scale(trans)+scale(elevation)+PA+scale(agri):PA+scale(elevation):P

A+(rdist|region_code3), family=binomial, data=data_rubber_subset) summary(model52)

model53<-

glmer(landuse~scale(agri)+scale(elevation)+region_type+PA+scale(agri):PA+scale(elevation):P

A+(rdist|region_code3), family=binomial, data=data_rubber_subset) summary(model53)

model54<-

glmer(landuse~scale(agri)+scale(trans)+region_type+PA+scale(agri):PA+region_type:PA+(rdist| region_code3), family=binomial, data=data_rubber_subset) summary(model54)

model55<-

glmer(landuse~scale(agri)+scale(elevation)+region_type+PA+scale(agri):PA+region_type:PA+( rdist|region_code3), family=binomial, data=data_rubber_subset) summary(model55)

model56<-

glmer(landuse~scale(agri)+scale(trans)+scale(elevation)+PA+scale(trans):PA+scale(elevation):P A+(rdist|region_code3), family=binomial, data=data_rubber_subset) summary(model56)

model57<glmer(landuse~scale(trans)+scale(elevation)+region_type+PA+scale(trans):PA+scale(elevation): PA+(rdist|region_code3), family=binomial, data=data_rubber_subset) summary(model57)

model58<-

glmer(landuse~scale(agri)+scale(trans)+region_type+PA+scale(trans):PA+region_type:PA+(rdis t|region_code3), family=binomial, data=data_rubber_subset) summary(model58)

model59<-

glmer(landuse~scale(trans)+scale(elevation)+region_type+PA+scale(trans):PA+region_type:PA

+(rdist|region_code3), family=binomial, data=data_rubber_subset) summary(model59)

##combine two sets model60<-

glmer(landuse~scale(agri)+scale(trans)+region_type+PA+scale(agri):region_type+scale(agri):P

A+(rdist|region_code3), family=binomial, data=data_rubber_subset) summary(model60)

model61<-

glmer(landuse~scale(agri)+scale(elevation)+region_type+PA+scale(agri):region_type+scale(agri

):PA+(rdist|region_code3), family=binomial, data=data_rubber_subset) summary(model61)

model62<-

glmer(landuse~scale(agri)+scale(trans)+region_type+PA+scale(agri):region_type+scale(trans):P

A+(rdist|region_code3), family=binomial, data=data_rubber_subset) summary(model62)

model63<-

glmer(landuse~scale(agri)+scale(elevation)+region_type+PA+scale(agri):region_type+scale(elev ation):PA+(rdist|region_code3), family=binomial, data=data_rubber_subset) summary(model63)

model64<-

glmer(landuse~scale(agri)+scale(trans)+region_type+PA+scale(agri):region_type+region_type:P

A+(rdist|region_code3), family=binomial, data=data_rubber_subset) summary(model64)

model65<-

glmer(landuse~scale(agri)+scale(elevation)+region_type+PA+scale(agri):region_type+region_ty pe:PA+(rdist|region_code3), family=binomial, data=data_rubber_subset) summary(model65)

model66<-

glmer(landuse~scale(agri)+scale(trans)+region_type+PA+scale(trans):region_type+scale(agri):P

A+(rdist|region_code3), family=binomial, data=data_rubber_subset) summary(model66)

model67<glmer(landuse~scale(agri)+scale(trans)+region_type+PA+scale(trans):region_type+scale(trans):

PA+(rdist|region_code3), family=binomial, data=data_rubber_subset)

summary(model67)

model68<-

glmer(landuse~scale(elevation)+scale(trans)+region_type+PA+scale(trans):region_type+scale(tr ans):PA+(rdist|region_code3), family=binomial, data=data_rubber_subset) summary(model68)

model69<-

glmer(landuse~scale(trans)+scale(elevation)+region_type+PA+scale(trans):region_type+scale(el evation):PA+(rdist|region_code3), family=binomial, data=data_rubber_subset) summary(model69)

model70<glmer(landuse~scale(agri)+scale(trans)+region_type+PA+scale(trans):region_type+region_type: PA+(rdist|region_code3), family=binomial, data=data_rubber_subset) summary(model70)

model71<-

glmer(landuse~scale(trans)+scale(elevation)+region_type+PA+scale(trans):region_type+region_ type:PA+(rdist|region_code3), family=binomial, data=data_rubber_subset) summary(model71)

model72<-

glmer(landuse~scale(agri)+scale(elevation)+region_type+PA+scale(elevation):region_type+scal e(agri):PA+(rdist|region_code3), family=binomial, data=data_rubber_subset) summary(model72)

model73<-

glmer(landuse~scale(trans)+scale(elevation)+region_type+PA+scale(elevation):region_type+sca le(trans):PA+(rdist|region_code3), family=binomial, data=data_rubber_subset) summary(model73)

model74<-

glmer(landuse~scale(agri)+scale(elevation)+region_type+PA+scale(elevation):region_type+scal e(elevation):PA+(rdist|region_code3), family=binomial, data=data_rubber_subset) summary(model74)

model75<-

glmer(landuse~scale(trans)+scale(elevation)+region_type+PA+scale(elevation):region_type+sca le(elevation):PA+(rdist|region_code3), family=binomial, data=data_rubber_subset) summary(model75)

model76<-

glmer(landuse~scale(agri)+scale(elevation)+region_type+PA+scale(elevation):region_type+regi on_type:PA+(rdist|region_code3), family=binomial, data=data_rubber_subset) summary(model76)

model77<-

glmer(landuse~scale(trans)+scale(elevation)+region_type+PA+scale(elevation):region_type+reg ion_type:PA+(rdist|region_code3), family=binomial, data=data_rubber_subset) summary(model77)

###three interactions###

model78<-

glmer(landuse~scale(agri)+scale(trans)+scale(elevation)+region_type+scale(agri):region_type+s cale(elevation):region_type+scale(trans):region_type+(rdist|region_code3), family=binomial, data=data_rubber_subset) summary(model78)

model79<-

glmer(landuse~scale(agri)+scale(trans)+region_type+PA+scale(agri):region_type+scale(agri):P A+scale(trans):region_type+(rdist|region_code3), family=binomial, data=data_rubber_subset) summary(model79)

model80<-

glmer(landuse~scale(agri)+scale(trans)+region_type+PA+scale(agri):region_type+scale(trans):P A+scale(trans):region_type+(rdist|region_code3), family=binomial, data=data_rubber_subset) summary(model80)

model81<-

glmer(landuse~scale(agri)+scale(trans)+region_type+PA+scale(agri):region_type+region_type:P A+scale(trans):region_type+(rdist|region_code3), family=binomial, data=data_rubber_subset) summary(model81)

model82<-

glmer(landuse~scale(agri)+scale(elevation)+region_type+PA+scale(agri):region_type+scale(agri

):PA+scale(elevation):region_type+(rdist|region_code3), family=binomial, data=data_rubber_subset) summary(model82)

model83<-

glmer(landuse~scale(agri)+scale(elevation)+region_type+PA+scale(agri):region_type+scale(elev ation):PA+scale(elevation):region_type+(rdist|region_code3), family=binomial, data=data_rubber_subset) summary(model83)

model84<-

glmer(landuse~scale(agri)+scale(elevation)+region_type+PA+scale(agri):region_type+region_ty pe:PA+scale(elevation):region_type+(rdist|region_code3), family=binomial, data=data_rubber_subset) summary(model84)

model85<-

glmer(landuse~scale(trans)+scale(elevation)+region_type+PA+scale(trans):region_type+scale(tr ans):PA+scale(elevation):region_type+(rdist|region_code3), family=binomial, data=data_rubber_subset) summary(model85)

model86<-

glmer(landuse~scale(trans)+scale(elevation)+region_type+PA+scale(trans):region_type+scale(el evation):PA+scale(elevation):region_type+(rdist|region_code3), family=binomial, data=data_rubber_subset) summary(model86)

model87<-

glmer(landuse~scale(trans)+scale(elevation)+region_type+PA+scale(trans):region_type+region_ type:PA+scale(elevation):region_type+(rdist|region_code3), family=binomial, data=data_rubber_subset) summary(model87)

###four interactions### model88<-

glmer(landuse~scale(agri)+scale(trans)+region_type+PA+scale(agri):region_type+scale(trans):re gion_type+scale(agri):PA+scale(trans):PA+(rdist|region_code3), family=binomial, data=data_rubber_subset) summary(model88)

model89<-

glmer(landuse~scale(agri)+scale(trans)+region_type+PA+scale(agri):region_type+scale(trans):re gion_type+scale(agri):PA+region_type:PA+(rdist|region_code3), family=binomial, data=data_rubber_subset) summary(model89)

model90<-

glmer(landuse~scale(agri)+scale(trans)+region_type+PA+scale(agri):region_type+scale(trans):re gion_type+scale(trans):PA+region_type:PA+(rdist|region_code3), family=binomial, data=data_rubber_subset) summary(model90)

model91<-

glmer(landuse~scale(agri)+scale(elevation)+region_type+PA+scale(agri):region_type+scale(elev ation):region_type+scale(agri):PA+scale(elevation):PA+(rdist|region_code3), family=binomial, data=data_rubber_subset) summary(model91)

model92<-

glmer(landuse~scale(agri)+scale(elevation)+region_type+PA+scale(agri):region_type+scale(elev ation):region_type+scale(agri):PA+region_type:PA+(rdist|region_code3), family=binomial, data=data_rubber_subset) summary(model92)

model93<-

glmer(landuse~scale(agri)+scale(elevation)+region_type+PA+scale(agri):region_type+scale(elev ation):region_type+region_type:PA+scale(elevation):PA+(rdist|region_code3), family=binomial, data=data_rubber_subset) summary(model93)

model94<-

glmer(landuse~scale(trans)+scale(elevation)+region_type+PA+scale(trans):region_type+scale(el evation):region_type+scale(trans):PA+scale(elevation):PA+(rdist|region_code3), family=binomial, data=data_rubber_subset) summary(model94)

model95<-

glmer(landuse~scale(trans)+scale(elevation)+region_type+PA+scale(trans):region_type+scale(el evation):region_type+scale(trans):PA+region_type:PA+(rdist|region_code3), family=binomial, data=data_rubber_subset) summary(model95)

model96<-

glmer(landuse~scale(trans)+scale(elevation)+region_type+PA+scale(trans):region_type+scale(el evation):region_type+region_type:PA+scale(elevation):PA+(rdist|region_code3), family=binomial, data=data_rubber_subset) summary(model96)

###try five interactions### model97<-

glmer(landuse~scale(agri)+scale(trans)+region_type+PA+scale(agri):region_type+scale(trans):re gion_type+scale(agri):PA+scale(trans):PA+region_type:PA+(rdist|region_code3), family=binomial, data=data_rubber_subset) summary(model97)

model98<-

glmer(landuse~scale(agri)+scale(elevation)+region_type+PA+scale(agri):region_type+scale(elev ation):region_type+scale(agri):PA+scale(elevation):PA+region_type:PA+(rdist|region_code3), family=binomial, data=data_rubber_subset) summary(model98)

model99<-

glmer(landuse~scale(trans)+scale(elevation)+region_type+PA+scale(trans):region_type+scale(el evation):region_type+scale(trans):PA+scale(elevation):PA+region_type:PA+(rdist|region_code3

), family=binomial, data=data_rubber_subset) summary(model99)

##select the best model

library(MuMIn)

models_sel_rubber<- model.sel(model_null,model1,model2,model3,model4,model5,model7, model8, model9, model10, model11,model12, model13, model14,

model15,model19,model21,model22,model23,model24,model29,model61,rank="AIC") sink(file="models_sel_rubber.txt")

models_sel_rubber

sink()

##dignostic plots library(arm) binnedplot(predict(model61, type="response"), resid(model61, type="response")) ##heteroscedasticity issue

library(effects) plot(allEffects(model61)) allEffects(model61)

sink(file="alleffects(model61).txt") allEffects(model61) sink()

sink("summary(model61).txt") summary(model61) sink()

rm(list=ls())

##############################################################################

#################model land use with characteristics of sugarcane###################### setwd("D:\\Myanmar project\\Resubmission 2\\Code and data (submit)")

data_all_raman<-read.csv("Appendix_S3_Supplementary_Data.csv",header=T)

data_sugarcaner<-data_all_raman[c(163192:186504),]

data_sugarcaner_subset<-data_sugarcaner[data_sugarcaner$land_use!=2,]

#convert to factors data_sugarcaner_subset$land_use <- as.factor(data_sugarcaner_subset$land_use) data_sugarcaner_subset$region_code1 <- as.factor(data_sugarcaner_subset$region_code1) data_sugarcaner_subset$region_code2 <- as.factor(data_sugarcaner_subset$region_code2) data_sugarcaner_subset$region_code3 <- as.factor(data_sugarcaner_subset$region_code3) data_sugarcaner_subset$resid<-as.factor(data_sugarcaner_subset$resid) data_sugarcaner_subset$PA<- as.factor(data_sugarcaner_subset$PA) colnames(data_sugarcaner_subset) <- c("landuse", "agri", "trans", "elevation",

"region_type","region_code1","region_code2","region_code3","rdist", "resid","PA","ID") head(data_sugarcaner_subset)

library(lme4)

##check collinearity

library(car)

model_vif <- glm(landuse ~ scale(agri)+scale(trans)+scale(elevation) +region_type+PA, family=binomial, data=data_sugarcaner_subset) summary(model_vif)

vif(model_vif)

###try to fix the random effects first

model_r1 <- glmer(landuse ~ scale(agri)+scale(trans)+scale(elevation) +region_type+PA+

(1|region_code1), family=binomial, data=data_sugarcaner_subset) summary(model_r1)

model_r2 <- glmer(landuse ~ scale(agri)+scale(trans) +scale(elevation)+region_type+PA+

(1|region_code2), family=binomial, data=data_sugarcaner_subset) summary(model_r2)

model_r3 <- glmer(landuse ~ scale(agri)+scale(trans)+scale(elevation) +region_type+PA+

(1|region_code3), family=binomial, data=data_sugarcaner_subset) summary(model_r3)

AIC(model_r1, model_r2, model_r3)

#choose region_code3 with smallest AIC

model_r4 <- glmer(landuse ~ scale(agri)+scale(trans)+scale(elevation) +region_type+PA+

(rdist|region_code3), family=binomial, data=data_sugarcaner_subset) summary(model_r4)

AIC(model_r3,model_r4)

##choose model_r4 with samllest AIC

############################################################################

###one variable### model_null<-glmer(landuse~1+(rdist|region_code3), family=binomial, data=data_sugarcaner_subset) summary(model_null)

model1<-glmer(landuse~scale(agri)+(rdist|region_code3), family=binomial, data=data_sugarcaner_subset) summary(model1)

model2<-glmer(landuse~scale(trans)+(rdist|region_code3), family=binomial, data=data_sugarcaner_subset) summary(model2)

model3<-glmer(landuse~scale(elevation)+(rdist|region_code3), family=binomial, data=data_sugarcaner_subset) summary(model3)

model4<-glmer(landuse~region_type+(rdist|region_code3), family=binomial, data=data_sugarcaner_subset) summary(model4)

model5<-glmer(landuse~PA+(rdist|region_code3), family=binomial, data=data_sugarcaner_subset) summary(model5)

###two variables###

model6<-glmer(landuse~scale(agri)+scale(trans)+(rdist|region_code3), family=binomial, data=data_sugarcaner_subset) summary(model6)

model7<-glmer(landuse~scale(agri)+scale(elevation)+(rdist|region_code3), family=binomial, data=data_sugarcaner_subset) summary(model7)

model8<-glmer(landuse~scale(agri)+region_type+(rdist|region_code3), family=binomial, data=data_sugarcaner_subset) summary(model8)

model9<-glmer(landuse~scale(trans)+region_type+(rdist|region_code3), family=binomial, data=data_sugarcaner_subset) summary(model9)

model10<-glmer(landuse~scale(elevation)+region_type+(rdist|region_code3), family=binomial, data=data_sugarcaner_subset) summary(model10)

model11<-glmer(landuse~scale(trans)+scale(elevation)+(rdist|region_code3), family=binomial, data=data_sugarcaner_subset) summary(model11)

model12<-glmer(landuse~scale(agri)+PA+(rdist|region_code3), family=binomial, data=data_sugarcaner_subset) summary(model12)

model13<-glmer(landuse~scale(trans)+PA+(rdist|region_code3), family=binomial, data=data_sugarcaner_subset) summary(model13)

model14<-glmer(landuse~scale(elevation)+PA+(rdist|region_code3), family=binomial, data=data_sugarcaner_subset) summary(model14)

model15<-glmer(landuse~region_type+PA+(rdist|region_code3), family=binomial, data=data_sugarcaner_subset) summary(model15)

###three variables###

model16<-glmer(landuse~scale(agri)+scale(trans)+region_type+(rdist|region_code3), family=binomial, data=data_sugarcaner_subset) summary(model16)

model17<-glmer(landuse~scale(agri)+scale(elevation)+region_type+(rdist|region_code3), family=binomial, data=data_sugarcaner_subset) summary(model17)

model18<-glmer(landuse~scale(agri)+scale(trans)+scale(elevation)+(rdist|region_code3), family=binomial, data=data_sugarcaner_subset) summary(model18) model19<-glmer(landuse~scale(trans)+scale(elevation)+region_type+(rdist|region_code3), family=binomial, data=data_sugarcaner_subset) summary(model19)

model20<-glmer(landuse~scale(agri)+scale(trans)+PA+(rdist|region_code3), family=binomial, data=data_sugarcaner_subset) summary(model20)

model21<-glmer(landuse~scale(agri)+scale(elevation)+PA+(rdist|region_code3), family=binomial, data=data_sugarcaner_subset) summary(model21)

model22<-glmer(landuse~scale(agri)+region_type+PA+(rdist|region_code3), family=binomial, data=data_sugarcaner_subset) summary(model22)

model23<-glmer(landuse~scale(trans)+scale(elevation)+PA+(rdist|region_code3), family=binomial, data=data_sugarcaner_subset) summary(model23)

model24<-glmer(landuse~scale(trans)+region_type+PA+(rdist|region_code3), family=binomial, data=data_sugarcaner_subset) summary(model24)

model25<-glmer(landuse~scale(elevation)+region_type+PA+(rdist|region_code3), family=binomial, data=data_sugarcaner_subset) summary(model25)

###four variables###

model26<-

glmer(landuse~scale(agri)+scale(trans)+scale(elevation)+region_type+(rdist|region_code3), family=binomial, data=data_sugarcaner_subset) summary(model26)

model27<-glmer(landuse~scale(agri)+scale(trans)+scale(elevation)+PA+(rdist|region_code3), family=binomial, data=data_sugarcaner_subset) summary(model27)

model28<-glmer(landuse~scale(agri)+scale(trans)+PA+region_type+(rdist|region_code3), family=binomial, data=data_sugarcaner_subset) summary(model28)

model29<-glmer(landuse~scale(agri)+PA+scale(elevation)+region_type+(rdist|region_code3), family=binomial, data=data_sugarcaner_subset) summary(model29)

model30<-glmer(landuse~PA+scale(trans)+scale(elevation)+region_type+(rdist|region_code3), family=binomial, data=data_sugarcaner_subset) summary(model30)

###five variables###

model31<-glmer(landuse ~ scale(agri)+scale(trans)+scale(elevation)

+region_type+PA+(rdist|region_code3), family=binomial, data=data_sugarcaner_subset)

###one interaction###

model32<-

glmer(landuse~scale(agri)+scale(trans)+scale(elevation)+region_type+scale(agri):region_type+(r dist|region_code3), family=binomial, data=data_sugarcaner_subset)

summary(model32)

model33<-

glmer(landuse~scale(agri)+scale(trans)+scale(elevation)+region_type+scale(trans):region_type+

(rdist|region_code3), family=binomial, data=data_sugarcaner_subset) summary(model33)

model34<-

glmer(landuse~scale(agri)+scale(trans)+scale(elevation)+region_type+scale(elevation):region_ty pe+(rdist|region_code3), family=binomial, data=data_sugarcaner_subset) summary(model34)

model35<-

glmer(landuse~scale(agri)+scale(trans)+scale(elevation)+PA+scale(agri):PA+(rdist|region_code

3), family=binomial, data=data_sugarcaner_subset) summary(model35)

model36<-

glmer(landuse~scale(agri)+scale(trans)+region_type+PA+scale(agri):PA+(rdist|region_code3), family=binomial, data=data_sugarcaner_subset) summary(model36)

model37<-

glmer(landuse~scale(agri)+scale(elevation)+region_type+PA+scale(agri):PA+(rdist|region_code

3), family=binomial, data=data_sugarcaner_subset) summary(model37)

model38<-

glmer(landuse~scale(agri)+scale(trans)+scale(elevation)+PA+scale(trans):PA+(rdist|region_code

3), family=binomial, data=data_sugarcaner_subset) summary(model38)

model39<-

glmer(landuse~scale(agri)+scale(trans)+region_type+PA+scale(trans):PA+(rdist|region_code3), family=binomial, data=data_sugarcaner_subset) summary(model39)

model40<-

glmer(landuse~scale(trans)+scale(elevation)+region_type+PA+scale(trans):PA+(rdist|region_co de3), family=binomial, data=data_sugarcaner_subset) summary(model40)

model41<-

glmer(landuse~scale(agri)+scale(trans)+scale(elevation)+PA+scale(elevation):PA+(rdist|region_ code3), family=binomial, data=data_sugarcaner_subset) summary(model41)

model42<-

glmer(landuse~scale(agri)+scale(elevation)+region_type+PA+scale(elevation):PA+(rdist|region_ code3), family=binomial, data=data_sugarcaner_subset) summary(model42)

model43<-

glmer(landuse~scale(trans)+scale(elevation)+region_type+PA+scale(elevation):PA+(rdist|region

_code3), family=binomial, data=data_sugarcaner_subset) summary(model43)

model44<-

glmer(landuse~scale(agri)+scale(trans)+region_type+PA+region_type:PA+(rdist|region_code3), family=binomial, data=data_sugarcaner_subset) summary(model44)

model45<-

glmer(landuse~scale(agri)+scale(elevation)+region_type+PA+region_type:PA+(rdist|region_cod e3), family=binomial, data=data_sugarcaner_subset) summary(model45)

model46<-

glmer(landuse~scale(trans)+scale(elevation)+region_type+PA+region_type:PA+(rdist|region_co de3), family=binomial, data=data_sugarcaner_subset) summary(model46)

tapply(fitted(model46),list(data_sugarcaner_subset$region_type,data_sugarcaner_subset$PA),m ean)

model46_test<-

glmer(landuse~scale(trans)+scale(elevation)+PA+region_type+PA:region_type+(rdist|region_co de3), family=binomial, data=data_sugarcaner_subset) summary(model46_test) plot(allEffects(model46_test))

###two interactions###

## with region

model47<-

glmer(landuse~scale(agri)+scale(trans)+scale(elevation)+region_type+scale(agri):region_type+s cale(trans):region_type+(rdist|region_code3), family=binomial, data=data_sugarcaner_subset) summary(model47)

model48<-

glmer(landuse~scale(agri)+scale(trans)+scale(elevation)+region_type+scale(agri):region_type+s cale(elevation):region_type+(rdist|region_code3), family=binomial, data=data_sugarcaner_subset) summary(model48)

model49<-

glmer(landuse~scale(agri)+scale(trans)+scale(elevation)+region_type+scale(trans):region_type+ scale(elevation):region_type+(rdist|region_code3), family=binomial, data=data_sugarcaner_subset) summary(model49)

##with PA model50<-

glmer(landuse~scale(agri)+scale(trans)+scale(elevation)+PA+scale(agri):PA+scale(trans):PA+(r dist|region_code3), family=binomial, data=data_sugarcaner_subset) summary(model50)

model51<-

glmer(landuse~scale(agri)+scale(trans)+region_type+PA+scale(agri):PA+scale(trans):PA+(rdist| region_code3), family=binomial, data=data_sugarcaner_subset) summary(model51)

model52<-

glmer(landuse~scale(agri)+scale(trans)+scale(elevation)+PA+scale(agri):PA+scale(elevation):P

A+(rdist|region_code3), family=binomial, data=data_sugarcaner_subset) summary(model52)

model53<-

glmer(landuse~scale(agri)+scale(elevation)+region_type+PA+scale(agri):PA+scale(elevation):P

A+(rdist|region_code3), family=binomial, data=data_sugarcaner_subset) summary(model53)

model54<-

glmer(landuse~scale(agri)+scale(trans)+region_type+PA+scale(agri):PA+region_type:PA+(rdist| region_code3), family=binomial, data=data_sugarcaner_subset) summary(model54)

model55<-

glmer(landuse~scale(agri)+scale(elevation)+region_type+PA+scale(agri):PA+region_type:PA+( rdist|region_code3), family=binomial, data=data_sugarcaner_subset) summary(model55)

model56<-

glmer(landuse~scale(agri)+scale(trans)+scale(elevation)+PA+scale(trans):PA+scale(elevation):P

A+(rdist|region_code3), family=binomial, data=data_sugarcaner_subset) summary(model56)

model57<glmer(landuse~scale(trans)+scale(elevation)+region_type+PA+scale(trans):PA+scale(elevation): PA+(rdist|region_code3), family=binomial, data=data_sugarcaner_subset) summary(model57)

model58<-

glmer(landuse~scale(agri)+scale(trans)+region_type+PA+scale(trans):PA+region_type:PA+(rdis t|region_code3), family=binomial, data=data_sugarcaner_subset) summary(model58)

model59<-

glmer(landuse~scale(trans)+scale(elevation)+region_type+PA+scale(trans):PA+region_type:PA

+(rdist|region_code3), family=binomial, data=data_sugarcaner_subset) summary(model59)

##combine two sets model60<-

glmer(landuse~scale(agri)+scale(trans)+region_type+PA+scale(agri):region_type+scale(agri):P

A+(rdist|region_code3), family=binomial, data=data_sugarcaner_subset) summary(model60)

model61<-

glmer(landuse~scale(agri)+scale(elevation)+region_type+PA+scale(agri):region_type+scale(agri

):PA+(rdist|region_code3), family=binomial, data=data_sugarcaner_subset) summary(model61)

model62<-

glmer(landuse~scale(agri)+scale(trans)+region_type+PA+scale(agri):region_type+scale(trans):P

A+(rdist|region_code3), family=binomial, data=data_sugarcaner_subset) summary(model62)

model63<-

glmer(landuse~scale(agri)+scale(elevation)+region_type+PA+scale(agri):region_type+scale(elev ation):PA+(rdist|region_code3), family=binomial, data=data_sugarcaner_subset) summary(model63)

model64<-

glmer(landuse~scale(agri)+scale(trans)+region_type+PA+scale(agri):region_type+region_type:P A+(rdist|region_code3), family=binomial, data=data_sugarcaner_subset) summary(model64)

model65<-

glmer(landuse~scale(agri)+scale(elevation)+region_type+PA+scale(agri):region_type+region_ty pe:PA+(rdist|region_code3), family=binomial, data=data_sugarcaner_subset) summary(model65)

model66<-

glmer(landuse~scale(agri)+scale(trans)+region_type+PA+scale(trans):region_type+scale(agri):P

A+(rdist|region_code3), family=binomial, data=data_sugarcaner_subset) summary(model66)

model67<glmer(landuse~scale(agri)+scale(trans)+region_type+PA+scale(trans):region_type+scale(trans):

PA+(rdist|region_code3), family=binomial, data=data_sugarcaner_subset)

summary(model67)

model68<-

glmer(landuse~scale(elevation)+scale(trans)+region_type+PA+scale(trans):region_type+scale(tr ans):PA+(rdist|region_code3), family=binomial, data=data_sugarcaner_subset) summary(model68)

model69<-

glmer(landuse~scale(trans)+scale(elevation)+region_type+PA+scale(trans):region_type+scale(el evation):PA+(rdist|region_code3), family=binomial, data=data_sugarcaner_subset) summary(model69)

model70<glmer(landuse~scale(agri)+scale(trans)+region_type+PA+scale(trans):region_type+region_type: PA+(rdist|region_code3), family=binomial, data=data_sugarcaner_subset) summary(model70)

model71<-

glmer(landuse~scale(trans)+scale(elevation)+region_type+PA+scale(trans):region_type+region_ type:PA+(rdist|region_code3), family=binomial, data=data_sugarcaner_subset) summary(model71)

model72<-

glmer(landuse~scale(agri)+scale(elevation)+region_type+PA+scale(elevation):region_type+scal e(agri):PA+(rdist|region_code3), family=binomial, data=data_sugarcaner_subset) summary(model72)

model73<-

glmer(landuse~scale(trans)+scale(elevation)+region_type+PA+scale(elevation):region_type+sca le(trans):PA+(rdist|region_code3), family=binomial, data=data_sugarcaner_subset) summary(model73)

model74<-

glmer(landuse~scale(agri)+scale(elevation)+region_type+PA+scale(elevation):region_type+scal e(elevation):PA+(rdist|region_code3), family=binomial, data=data_sugarcaner_subset) summary(model74)

model75<-

glmer(landuse~scale(trans)+scale(elevation)+region_type+PA+scale(elevation):region_type+sca le(elevation):PA+(rdist|region_code3), family=binomial, data=data_sugarcaner_subset) summary(model75)

model76<-

glmer(landuse~scale(agri)+scale(elevation)+region_type+PA+scale(elevation):region_type+regi on_type:PA+(rdist|region_code3), family=binomial, data=data_sugarcaner_subset) summary(model76)

model77<-

glmer(landuse~scale(trans)+scale(elevation)+region_type+PA+scale(elevation):region_type+reg ion_type:PA+(rdist|region_code3), family=binomial, data=data_sugarcaner_subset) summary(model77)

###three interactions###

model78<-

glmer(landuse~scale(agri)+scale(trans)+scale(elevation)+region_type+scale(agri):region_type+s cale(elevation):region_type+scale(trans):region_type+(rdist|region_code3), family=binomial, data=data_sugarcaner_subset) summary(model78)

model79<-

glmer(landuse~scale(agri)+scale(trans)+region_type+PA+scale(agri):region_type+scale(agri):P

A+scale(trans):region_type+(rdist|region_code3), family=binomial, data=data_sugarcaner_subset) summary(model79)

model80<-

glmer(landuse~scale(agri)+scale(trans)+region_type+PA+scale(agri):region_type+scale(trans):P

A+scale(trans):region_type+(rdist|region_code3), family=binomial, data=data_sugarcaner_subset) summary(model80)

model81<-

glmer(landuse~scale(agri)+scale(trans)+region_type+PA+scale(agri):region_type+region_type:P

A+scale(trans):region_type+(rdist|region_code3), family=binomial, data=data_sugarcaner_subset) summary(model81)

model82<-

glmer(landuse~scale(agri)+scale(elevation)+region_type+PA+scale(agri):region_type+scale(agri

):PA+scale(elevation):region_type+(rdist|region_code3), family=binomial, data=data_sugarcaner_subset) summary(model82)

model83<-

glmer(landuse~scale(agri)+scale(elevation)+region_type+PA+scale(agri):region_type+scale(elev ation):PA+scale(elevation):region_type+(rdist|region_code3), family=binomial, data=data_sugarcaner_subset) summary(model83)

model84<-

glmer(landuse~scale(agri)+scale(elevation)+region_type+PA+scale(agri):region_type+region_ty pe:PA+scale(elevation):region_type+(rdist|region_code3), family=binomial, data=data_sugarcaner_subset) summary(model84)

model85<-

glmer(landuse~scale(trans)+scale(elevation)+region_type+PA+scale(trans):region_type+scale(tr ans):PA+scale(elevation):region_type+(rdist|region_code3), family=binomial, data=data_sugarcaner_subset) summary(model85)

model86<-

glmer(landuse~scale(trans)+scale(elevation)+region_type+PA+scale(trans):region_type+scale(el evation):PA+scale(elevation):region_type+(rdist|region_code3), family=binomial, data=data_sugarcaner_subset) summary(model86)

model87<-

glmer(landuse~scale(trans)+scale(elevation)+region_type+PA+scale(trans):region_type+region_ type:PA+scale(elevation):region_type+(rdist|region_code3), family=binomial, data=data_sugarcaner_subset) summary(model87)

###four interactions### model88<-

glmer(landuse~scale(agri)+scale(trans)+region_type+PA+scale(agri):region_type+scale(trans):re gion_type+scale(agri):PA+scale(trans):PA+(rdist|region_code3), family=binomial, data=data_sugarcaner_subset) summary(model88)

model89<-

glmer(landuse~scale(agri)+scale(trans)+region_type+PA+scale(agri):region_type+scale(trans):re gion_type+scale(agri):PA+region_type:PA+(rdist|region_code3), family=binomial, data=data_sugarcaner_subset) summary(model89)

model90<-

glmer(landuse~scale(agri)+scale(trans)+region_type+PA+scale(agri):region_type+scale(trans):re gion_type+scale(trans):PA+region_type:PA+(rdist|region_code3), family=binomial, data=data_sugarcaner_subset) summary(model90)

model91<-

glmer(landuse~scale(agri)+scale(elevation)+region_type+PA+scale(agri):region_type+scale(elev ation):region_type+scale(agri):PA+scale(elevation):PA+(rdist|region_code3), family=binomial, data=data_sugarcaner_subset) summary(model91)

model92<-

glmer(landuse~scale(agri)+scale(elevation)+region_type+PA+scale(agri):region_type+scale(elev ation):region_type+scale(agri):PA+region_type:PA+(rdist|region_code3), family=binomial, data=data_sugarcaner_subset) summary(model92)

model93<-

glmer(landuse~scale(agri)+scale(elevation)+region_type+PA+scale(agri):region_type+scale(elev ation):region_type+region_type:PA+scale(elevation):PA+(rdist|region_code3), family=binomial, data=data_sugarcaner_subset) summary(model93)

model94<-

glmer(landuse~scale(trans)+scale(elevation)+region_type+PA+scale(trans):region_type+scale(el evation):region_type+scale(trans):PA+scale(elevation):PA+(rdist|region_code3), family=binomial, data=data_sugarcaner_subset) summary(model94)

model95<-

glmer(landuse~scale(trans)+scale(elevation)+region_type+PA+scale(trans):region_type+scale(el evation):region_type+scale(trans):PA+region_type:PA+(rdist|region_code3), family=binomial, data=data_sugarcaner_subset) summary(model95)

model96<-

glmer(landuse~scale(trans)+scale(elevation)+region_type+PA+scale(trans):region_type+scale(el evation):region_type+region_type:PA+scale(elevation):PA+(rdist|region_code3), family=binomial, data=data_sugarcaner_subset) summary(model96)

###try five interactions### model97<-

glmer(landuse~scale(agri)+scale(trans)+region_type+PA+scale(agri):region_type+scale(trans):re gion_type+scale(agri):PA+scale(trans):PA+region_type:PA+(rdist|region_code3), family=binomial, data=data_sugarcaner_subset) summary(model97)

model98<-

glmer(landuse~scale(agri)+scale(elevation)+region_type+PA+scale(agri):region_type+scale(elev ation):region_type+scale(agri):PA+scale(elevation):PA+region_type:PA+(rdist|region_code3), family=binomial, data=data_sugarcaner_subset) summary(model98)

model99<-

glmer(landuse~scale(trans)+scale(elevation)+region_type+PA+scale(trans):region_type+scale(el evation):region_type+scale(trans):PA+scale(elevation):PA+region_type:PA+(rdist|region_code3

), family=binomial, data=data_sugarcaner_subset) summary(model99)

##select the best model library(MuMIn)

models_sel_sugarcaner<-

model.sel(model_null,model1,model2,model3,model4,model5,model6,model7, model8, model9, model10, model11,model12, model13, model14,

model15,model16,model18,model20,model21,model22,model23,model24,model28, model35,model40,model46,rank="AIC") sink(file="models_sel_sugarcaner.txt")

models_sel_sugarcaner

sink()

models_sel_sugarcaner

sink()

##dignostic plots library(arm) binnedplot(predict(model35, type="response"), resid(model35, type="response"))

library(effects) plot(allEffects(model35)) allEffects(model35)

sink(file="alleffects(model35).txt") allEffects(model35) sink()

sink("summary(model35).txt") summary(model35)

sink()

####################prediction with actual yield ###############################

#1. Model baseline scenario, just put the actual yield in pred_sugarcaner <- predict(model35, data_sugarcaner_subset, re.form=NULL, type="response") head(pred_sugarcaner)

write.csv(pred_sugarcaner, "pred_sugarcaner.csv")

##########################prediction with potential yield ###################### ##get potential yield data

data_all<-read.csv("Appendix_S4_Supplementary_Data.csv",header=T)

data_sugarcane<-data_all[c(163192:186504),]

data_sugarcane_subset<-data_sugarcane[data_sugarcane$land_use!=2,]

data_sugarcane_subset$land_use <- as.factor(data_sugarcane_subset$land_use) data_sugarcane_subset$region_code1 <- as.factor(data_sugarcane_subset$region_code1) data_sugarcane_subset$region_code2 <- as.factor(data_sugarcane_subset$region_code2) data_sugarcane_subset$region_code3 <- as.factor(data_sugarcane_subset$region_code3) data_sugarcane_subset$resid<-as.factor(data_sugarcane_subset$resid) data_sugarcane_subset$PA<- as.factor(data_sugarcane_subset$PA)

colnames(data_sugarcane_subset) <- c("landuse", "agri", "trans", "elevation",

"region_type","region_code1","region_code2","region_code3","rdist", "resid","PA","ID") head(data_sugarcane_subset)

#2. Scenario: actual yield being replaced by potential yield data pred_sugarcane <- predict(model35, data_sugarcane_subset, re.form=NULL, type="response") head(pred_sugarcane)

write.csv(pred_sugarcane, "pred_sugarcane.csv")

#3. Scenario A: agr increase 50%

sugarcane_y <- cbind(data_sugarcane_subset$landuse, (data_sugarcane_subset$agri)*1.5 , data_sugarcane_subset[,3:12])

colnames(sugarcane_y) <- c("landuse", "agri", "trans","elevation","region_type","region_code1","region_code2","region_code3","rdist","resid","PA","ID")

summary(sugarcane_y)

pred_sugarcane<-predict(model35, sugarcane_y, re.form=NULL, type="response"))

write.csv(pred_sugarcane, "pred_sugarcane_agri.csv")

#4. Scenario B: transport costs increase by 35%

sugarcane_trans_y <- cbind(data_sugarcane_subset[,1:2], data_sugarcane_subset[,3]*1.35, data_sugarcane_subset[,4:12]) colnames(sugarcane_trans_y) <- c("landuse", "agri",

"trans","elevation","region_type","region_code1","region_code2","region_code3","rdist","resid",

"PA","ID") summary(sugarcane_trans_y)

pred_trans_sugarcane<-predict(model35, sugarcane_trans_y, re.form=NULL, type="response")

write.csv(pred_trans_sugarcane, "pred_sugarcane_trans.csv")

#5. Scenario C: political stability has total conversion

region_sugarcane <- data_sugarcane_subset

summary(region_sugarcane) region_sugarcane[,5] <- "Division"

region_sugarcane[,5] <- as.factor(region_sugarcane[,5])

pred_region_sugarcane <- predict(model35, region_sugarcane, re.form=NULL, type="response") head(pred_region_sugarcane)

write.csv(pred_region_sugarcane, "pred_sugarcane_stability.csv")

#6. Scenario D: worst case. agri increase 50%, and stability has total conversion sugarcane_worstcase<-sugarcane_y

sugarcane_worstcase[,5]<-"Division"

pred_worstcase_sugarcane<-predict(model35, sugarcane_worstcase, re.form=NULL, type="response")

write.csv(pred_worstcase_sugarcane, "pred_sugarcane_worstcase.csv")

rm(list=ls())

##############################################################################

##################model land use with characteristics of tobacco#######################

setwd("D:\\Myanmar project\\Resubmission 2\\Code and data (submit)")

data_all_raman<-read.csv("Appendix_S3_Supplementary_Data.csv",header=T)

data_tobaccor<-data_all_raman[c(186505:209817),]

data_tobaccor_subset<-data_tobaccor[data_tobaccor$land_use!=2,]

#convert to factors data_tobaccor_subset$land_use <- as.factor(data_tobaccor_subset$land_use) data_tobaccor_subset$region_code1 <- as.factor(data_tobaccor_subset$region_code1) data_tobaccor_subset$region_code2 <- as.factor(data_tobaccor_subset$region_code2) data_tobaccor_subset$region_code3 <- as.factor(data_tobaccor_subset$region_code3) data_tobaccor_subset$resid<-as.factor(data_tobaccor_subset$resid) data_tobaccor_subset$PA<- as.factor(data_tobaccor_subset$PA)

colnames(data_tobaccor_subset) <- c("landuse", "agri", "trans", "elevation",

"region_type","region_code1","region_code2","region_code3","rdist", "resid","PA","ID") head(data_tobaccor_subset)

library(lme4)

##check collinearity

library(car)

model_vif <- glm(landuse ~ scale(agri)+scale(trans)+scale(elevation) +region_type+PA, family=binomial, data=data_tobaccor_subset) summary(model_vif)

vif(model_vif)

###try to fix the random effects first

model_r1 <- glmer(landuse ~ scale(agri)+scale(trans)+scale(elevation) +region_type+PA+

(1|region_code1), family=binomial, data=data_tobaccor_subset) summary(model_r1)

model_r2 <- glmer(landuse ~ scale(agri)+scale(trans) +scale(elevation)+region_type+PA+

(1|region_code2), family=binomial, data=data_tobaccor_subset) summary(model_r2)

model_r3 <- glmer(landuse ~ scale(agri)+scale(trans)+scale(elevation) +region_type+PA+ (1|region_code3), family=binomial, data=data_tobaccor_subset) summary(model_r3)

AIC(model_r1, model_r2, model_r3)

#choose region_code3 with smallest AIC

model_r4 <- glmer(landuse ~ scale(agri)+scale(trans)+scale(elevation) +region_type+PA+

(rdist|region_code3), family=binomial, data=data_tobaccor_subset) summary(model_r4)

AIC(model_r3,model_r4)

#############################################################################

###one variable### model_null<-glmer(landuse~1+(rdist|region_code3), family=binomial, data=data_tobaccor_subset) summary(model_null)

model1<-glmer(landuse~scale(agri)+(rdist|region_code3), family=binomial, data=data_tobaccor_subset) summary(model1)

model2<-glmer(landuse~scale(trans)+(rdist|region_code3), family=binomial, data=data_tobaccor_subset) summary(model2) model3<-glmer(landuse~scale(elevation)+(rdist|region_code3), family=binomial, data=data_tobaccor_subset) summary(model3)

model4<-glmer(landuse~region_type+(rdist|region_code3), family=binomial, data=data_tobaccor_subset) summary(model4)

model5<-glmer(landuse~PA+(rdist|region_code3), family=binomial, data=data_tobaccor_subset) summary(model5)

###two variables###

model6<-glmer(landuse~scale(agri)+scale(trans)+(rdist|region_code3), family=binomial, data=data_tobaccor_subset) summary(model6)

model7<-glmer(landuse~scale(agri)+scale(elevation)+(rdist|region_code3), family=binomial, data=data_tobaccor_subset) summary(model7)

model8<-glmer(landuse~scale(agri)+region_type+(rdist|region_code3), family=binomial, data=data_tobaccor_subset) summary(model8)

model9<-glmer(landuse~scale(trans)+region_type+(rdist|region_code3), family=binomial, data=data_tobaccor_subset) summary(model9) model10<-glmer(landuse~scale(elevation)+region_type+(rdist|region_code3), family=binomial, data=data_tobaccor_subset) summary(model10)

model11<-glmer(landuse~scale(trans)+scale(elevation)+(rdist|region_code3), family=binomial, data=data_tobaccor_subset) summary(model11)

model12<-glmer(landuse~scale(agri)+PA+(rdist|region_code3), family=binomial, data=data_tobaccor_subset) summary(model12)

model13<-glmer(landuse~scale(trans)+PA+(rdist|region_code3), family=binomial, data=data_tobaccor_subset) summary(model13)

model14<-glmer(landuse~scale(elevation)+PA+(rdist|region_code3), family=binomial, data=data_tobaccor_subset) summary(model14)

model15<-glmer(landuse~region_type+PA+(rdist|region_code3), family=binomial, data=data_tobaccor_subset) summary(model15)

###three variables###

model16<-glmer(landuse~scale(agri)+scale(trans)+region_type+(rdist|region_code3), family=binomial, data=data_tobaccor_subset) summary(model16) model17<-glmer(landuse~scale(agri)+scale(elevation)+region_type+(rdist|region_code3), family=binomial, data=data_tobaccor_subset) summary(model17)

model18<-glmer(landuse~scale(agri)+scale(trans)+scale(elevation)+(rdist|region_code3), family=binomial, data=data_tobaccor_subset) summary(model18)

model19<-glmer(landuse~scale(trans)+scale(elevation)+region_type+(rdist|region_code3), family=binomial, data=data_tobaccor_subset) summary(model19)

model20<-glmer(landuse~scale(agri)+scale(trans)+PA+(rdist|region_code3), family=binomial, data=data_tobaccor_subset) summary(model20)

model21<-glmer(landuse~scale(agri)+scale(elevation)+PA+(rdist|region_code3), family=binomial, data=data_tobaccor_subset) summary(model21)

model22<-glmer(landuse~scale(agri)+region_type+PA+(rdist|region_code3), family=binomial, data=data_tobaccor_subset) summary(model22)

model23<-glmer(landuse~scale(trans)+scale(elevation)+PA+(rdist|region_code3), family=binomial, data=data_tobaccor_subset) summary(model23)

model24<-glmer(landuse~scale(trans)+region_type+PA+(rdist|region_code3), family=binomial, data=data_tobaccor_subset) summary(model24) model25<-glmer(landuse~scale(elevation)+region_type+PA+(rdist|region_code3), family=binomial, data=data_tobaccor_subset) summary(model25)

###four variables###

model26<-

glmer(landuse~scale(agri)+scale(trans)+scale(elevation)+region_type+(rdist|region_code3), family=binomial, data=data_tobaccor_subset) summary(model26)

model27<-glmer(landuse~scale(agri)+scale(trans)+scale(elevation)+PA+(rdist|region_code3), family=binomial, data=data_tobaccor_subset) summary(model27)

model28<-glmer(landuse~scale(agri)+scale(trans)+PA+region_type+(rdist|region_code3), family=binomial, data=data_tobaccor_subset) summary(model28)

model29<-glmer(landuse~scale(agri)+PA+scale(elevation)+region_type+(rdist|region_code3), family=binomial, data=data_tobaccor_subset) summary(model29)

model30<-glmer(landuse~PA+scale(trans)+scale(elevation)+region_type+(rdist|region_code3), family=binomial, data=data_tobaccor_subset) summary(model30)

###five variables###

model31<-glmer(landuse ~ scale(agri)+scale(trans)+scale(elevation)

+region_type+PA+(rdist|region_code3), family=binomial, data=data_tobaccor_subset)

###one interaction###

model32<-

glmer(landuse~scale(agri)+scale(trans)+scale(elevation)+region_type+scale(agri):region_type+(r dist|region_code3), family=binomial, data=data_tobaccor_subset) summary(model32)

model33<-

glmer(landuse~scale(agri)+scale(trans)+scale(elevation)+region_type+scale(trans):region_type+

(rdist|region_code3), family=binomial, data=data_tobaccor_subset) summary(model33)

model34<-

glmer(landuse~scale(agri)+scale(trans)+scale(elevation)+region_type+scale(elevation):region_ty pe+(rdist|region_code3), family=binomial, data=data_tobaccor_subset) summary(model34)

model35<-

glmer(landuse~scale(agri)+scale(trans)+scale(elevation)+PA+scale(agri):PA+(rdist|region_code

3), family=binomial, data=data_tobaccor_subset) summary(model35)

model36<-

glmer(landuse~scale(agri)+scale(trans)+region_type+PA+scale(agri):PA+(rdist|region_code3), family=binomial, data=data_tobaccor_subset) summary(model36)

model37<-

glmer(landuse~scale(agri)+scale(elevation)+region_type+PA+scale(agri):PA+(rdist|region_code 3), family=binomial, data=data_tobaccor_subset)

summary(model37)

model38<-

glmer(landuse~scale(agri)+scale(trans)+scale(elevation)+PA+scale(trans):PA+(rdist|region_code

3), family=binomial, data=data_tobaccor_subset) summary(model38)

model39<-

glmer(landuse~scale(agri)+scale(trans)+region_type+PA+scale(trans):PA+(rdist|region_code3), family=binomial, data=data_tobaccor_subset) summary(model39)

model40<-

glmer(landuse~scale(trans)+scale(elevation)+region_type+PA+scale(trans):PA+(rdist|region_co de3), family=binomial, data=data_tobaccor_subset) summary(model40)

model41<-

glmer(landuse~scale(agri)+scale(trans)+scale(elevation)+PA+scale(elevation):PA+(rdist|region_ code3), family=binomial, data=data_tobaccor_subset) summary(model41)

model42<-

glmer(landuse~scale(agri)+scale(elevation)+region_type+PA+scale(elevation):PA+(rdist|region_ code3), family=binomial, data=data_tobaccor_subset) summary(model42)

model43<-

glmer(landuse~scale(trans)+scale(elevation)+region_type+PA+scale(elevation):PA+(rdist|region _code3), family=binomial, data=data_tobaccor_subset)

summary(model43)

model44<-

glmer(landuse~scale(agri)+scale(trans)+region_type+PA+region_type:PA+(rdist|region_code3), family=binomial, data=data_tobaccor_subset) summary(model44)

model45<-

glmer(landuse~scale(agri)+scale(elevation)+region_type+PA+region_type:PA+(rdist|region_cod e3), family=binomial, data=data_tobaccor_subset) summary(model45)

model46<-

glmer(landuse~scale(trans)+scale(elevation)+region_type+PA+region_type:PA+(rdist|region_co de3), family=binomial, data=data_tobaccor_subset) summary(model46)

###two interactions###

## with region

model47<-

glmer(landuse~scale(agri)+scale(trans)+scale(elevation)+region_type+scale(agri):region_type+s cale(trans):region_type+(rdist|region_code3), family=binomial, data=data_tobaccor_subset) summary(model47)

model48<-

glmer(landuse~scale(agri)+scale(trans)+scale(elevation)+region_type+scale(agri):region_type+s cale(elevation):region_type+(rdist|region_code3), family=binomial, data=data_tobaccor_subset) summary(model48)

model49<-

glmer(landuse~scale(agri)+scale(trans)+scale(elevation)+region_type+scale(trans):region_type+ scale(elevation):region_type+(rdist|region_code3), family=binomial, data=data_tobaccor_subset) summary(model49)

##with PA model50<-

glmer(landuse~scale(agri)+scale(trans)+scale(elevation)+PA+scale(agri):PA+scale(trans):PA+(r dist|region_code3), family=binomial, data=data_tobaccor_subset) summary(model50)

model51<-

glmer(landuse~scale(agri)+scale(trans)+region_type+PA+scale(agri):PA+scale(trans):PA+(rdist| region_code3), family=binomial, data=data_tobaccor_subset) summary(model51)

model52<-

glmer(landuse~scale(agri)+scale(trans)+scale(elevation)+PA+scale(agri):PA+scale(elevation):P

A+(rdist|region_code3), family=binomial, data=data_tobaccor_subset) summary(model52)

model53<-

glmer(landuse~scale(agri)+scale(elevation)+region_type+PA+scale(agri):PA+scale(elevation):P

A+(rdist|region_code3), family=binomial, data=data_tobaccor_subset) summary(model53)

model54<-

glmer(landuse~scale(agri)+scale(trans)+region_type+PA+scale(agri):PA+region_type:PA+(rdist| region_code3), family=binomial, data=data_tobaccor_subset) summary(model54)

model55<-

glmer(landuse~scale(agri)+scale(elevation)+region_type+PA+scale(agri):PA+region_type:PA+( rdist|region_code3), family=binomial, data=data_tobaccor_subset) summary(model55)

model56<-

glmer(landuse~scale(agri)+scale(trans)+scale(elevation)+PA+scale(trans):PA+scale(elevation):P

A+(rdist|region_code3), family=binomial, data=data_tobaccor_subset) summary(model56)

model57<glmer(landuse~scale(trans)+scale(elevation)+region_type+PA+scale(trans):PA+scale(elevation): PA+(rdist|region_code3), family=binomial, data=data_tobaccor_subset) summary(model57)

model58<-

glmer(landuse~scale(agri)+scale(trans)+region_type+PA+scale(trans):PA+region_type:PA+(rdis t|region_code3), family=binomial, data=data_tobaccor_subset) summary(model58)

model59<-

glmer(landuse~scale(trans)+scale(elevation)+region_type+PA+scale(trans):PA+region_type:PA

+(rdist|region_code3), family=binomial, data=data_tobaccor_subset) summary(model59)

##combine two sets model60<-

glmer(landuse~scale(agri)+scale(trans)+region_type+PA+scale(agri):region_type+scale(agri):P

A+(rdist|region_code3), family=binomial, data=data_tobaccor_subset) summary(model60)

model61<-

glmer(landuse~scale(agri)+scale(elevation)+region_type+PA+scale(agri):region_type+scale(agri

):PA+(rdist|region_code3), family=binomial, data=data_tobaccor_subset) summary(model61)

model62<-

glmer(landuse~scale(agri)+scale(trans)+region_type+PA+scale(agri):region_type+scale(trans):P

A+(rdist|region_code3), family=binomial, data=data_tobaccor_subset) summary(model62)

model63<-

glmer(landuse~scale(agri)+scale(elevation)+region_type+PA+scale(agri):region_type+scale(elev ation):PA+(rdist|region_code3), family=binomial, data=data_tobaccor_subset) summary(model63)

model64<-

glmer(landuse~scale(agri)+scale(trans)+region_type+PA+scale(agri):region_type+region_type:P

A+(rdist|region_code3), family=binomial, data=data_tobaccor_subset) summary(model64)

model65<-

glmer(landuse~scale(agri)+scale(elevation)+region_type+PA+scale(agri):region_type+region_ty pe:PA+(rdist|region_code3), family=binomial, data=data_tobaccor_subset) summary(model65)

model66<-

glmer(landuse~scale(agri)+scale(trans)+region_type+PA+scale(trans):region_type+scale(agri):P

A+(rdist|region_code3), family=binomial, data=data_tobaccor_subset) summary(model66)

model67<glmer(landuse~scale(agri)+scale(trans)+region_type+PA+scale(trans):region_type+scale(trans):

PA+(rdist|region_code3), family=binomial, data=data_tobaccor_subset)

summary(model67)

model68<-

glmer(landuse~scale(elevation)+scale(trans)+region_type+PA+scale(trans):region_type+scale(tr ans):PA+(rdist|region_code3), family=binomial, data=data_tobaccor_subset) summary(model68)

model69<-

glmer(landuse~scale(trans)+scale(elevation)+region_type+PA+scale(trans):region_type+scale(el evation):PA+(rdist|region_code3), family=binomial, data=data_tobaccor_subset) summary(model69)

model70<glmer(landuse~scale(agri)+scale(trans)+region_type+PA+scale(trans):region_type+region_type: PA+(rdist|region_code3), family=binomial, data=data_tobaccor_subset) summary(model70)

model71<-

glmer(landuse~scale(trans)+scale(elevation)+region_type+PA+scale(trans):region_type+region_ type:PA+(rdist|region_code3), family=binomial, data=data_tobaccor_subset) summary(model71)

model72<-

glmer(landuse~scale(agri)+scale(elevation)+region_type+PA+scale(elevation):region_type+scal e(agri):PA+(rdist|region_code3), family=binomial, data=data_tobaccor_subset) summary(model72)

model73<-

glmer(landuse~scale(trans)+scale(elevation)+region_type+PA+scale(elevation):region_type+sca le(trans):PA+(rdist|region_code3), family=binomial, data=data_tobaccor_subset) summary(model73)

model74<-

glmer(landuse~scale(agri)+scale(elevation)+region_type+PA+scale(elevation):region_type+scal e(elevation):PA+(rdist|region_code3), family=binomial, data=data_tobaccor_subset) summary(model74)

model75<-

glmer(landuse~scale(trans)+scale(elevation)+region_type+PA+scale(elevation):region_type+sca le(elevation):PA+(rdist|region_code3), family=binomial, data=data_tobaccor_subset) summary(model75)

model76<-

glmer(landuse~scale(agri)+scale(elevation)+region_type+PA+scale(elevation):region_type+regi on_type:PA+(rdist|region_code3), family=binomial, data=data_tobaccor_subset) summary(model76)

model77<-

glmer(landuse~scale(trans)+scale(elevation)+region_type+PA+scale(elevation):region_type+reg ion_type:PA+(rdist|region_code3), family=binomial, data=data_tobaccor_subset) summary(model77)

###three interactions###

model78<-

glmer(landuse~scale(agri)+scale(trans)+scale(elevation)+region_type+scale(agri):region_type+s cale(elevation):region_type+scale(trans):region_type+(rdist|region_code3), family=binomial, data=data_tobaccor_subset) summary(model78)

model79<-

glmer(landuse~scale(agri)+scale(trans)+region_type+PA+scale(agri):region_type+scale(agri):P A+scale(trans):region_type+(rdist|region_code3), family=binomial, data=data_tobaccor_subset) summary(model79)

model80<-

glmer(landuse~scale(agri)+scale(trans)+region_type+PA+scale(agri):region_type+scale(trans):P A+scale(trans):region_type+(rdist|region_code3), family=binomial, data=data_tobaccor_subset) summary(model80)

model81<-

glmer(landuse~scale(agri)+scale(trans)+region_type+PA+scale(agri):region_type+region_type:P A+scale(trans):region_type+(rdist|region_code3), family=binomial, data=data_tobaccor_subset) summary(model81)

model82<-

glmer(landuse~scale(agri)+scale(elevation)+region_type+PA+scale(agri):region_type+scale(agri

):PA+scale(elevation):region_type+(rdist|region_code3), family=binomial, data=data_tobaccor_subset) summary(model82)

model83<-

glmer(landuse~scale(agri)+scale(elevation)+region_type+PA+scale(agri):region_type+scale(elev ation):PA+scale(elevation):region_type+(rdist|region_code3), family=binomial, data=data_tobaccor_subset) summary(model83)

model84<-

glmer(landuse~scale(agri)+scale(elevation)+region_type+PA+scale(agri):region_type+region_ty pe:PA+scale(elevation):region_type+(rdist|region_code3), family=binomial, data=data_tobaccor_subset) summary(model84)

model85<-

glmer(landuse~scale(trans)+scale(elevation)+region_type+PA+scale(trans):region_type+scale(tr ans):PA+scale(elevation):region_type+(rdist|region_code3), family=binomial, data=data_tobaccor_subset) summary(model85)

model86<-

glmer(landuse~scale(trans)+scale(elevation)+region_type+PA+scale(trans):region_type+scale(el evation):PA+scale(elevation):region_type+(rdist|region_code3), family=binomial, data=data_tobaccor_subset) summary(model86)

model87<-

glmer(landuse~scale(trans)+scale(elevation)+region_type+PA+scale(trans):region_type+region_ type:PA+scale(elevation):region_type+(rdist|region_code3), family=binomial, data=data_tobaccor_subset) summary(model87)

###four interactions### model88<-

glmer(landuse~scale(agri)+scale(trans)+region_type+PA+scale(agri):region_type+scale(trans):re gion_type+scale(agri):PA+scale(trans):PA+(rdist|region_code3), family=binomial, data=data_tobaccor_subset) summary(model88)

model89<-

glmer(landuse~scale(agri)+scale(trans)+region_type+PA+scale(agri):region_type+scale(trans):re gion_type+scale(agri):PA+region_type:PA+(rdist|region_code3), family=binomial, data=data_tobaccor_subset) summary(model89)

model90<-

glmer(landuse~scale(agri)+scale(trans)+region_type+PA+scale(agri):region_type+scale(trans):re gion_type+scale(trans):PA+region_type:PA+(rdist|region_code3), family=binomial, data=data_tobaccor_subset) summary(model90)

model91<-

glmer(landuse~scale(agri)+scale(elevation)+region_type+PA+scale(agri):region_type+scale(elev ation):region_type+scale(agri):PA+scale(elevation):PA+(rdist|region_code3), family=binomial, data=data_tobaccor_subset) summary(model91)

model92<-

glmer(landuse~scale(agri)+scale(elevation)+region_type+PA+scale(agri):region_type+scale(elev ation):region_type+scale(agri):PA+region_type:PA+(rdist|region_code3), family=binomial, data=data_tobaccor_subset) summary(model92)

model93<-

glmer(landuse~scale(agri)+scale(elevation)+region_type+PA+scale(agri):region_type+scale(elev ation):region_type+region_type:PA+scale(elevation):PA+(rdist|region_code3), family=binomial, data=data_tobaccor_subset) summary(model93)

model94<-

glmer(landuse~scale(trans)+scale(elevation)+region_type+PA+scale(trans):region_type+scale(el evation):region_type+scale(trans):PA+scale(elevation):PA+(rdist|region_code3), family=binomial, data=data_tobaccor_subset) summary(model94)

model95<-

glmer(landuse~scale(trans)+scale(elevation)+region_type+PA+scale(trans):region_type+scale(el evation):region_type+scale(trans):PA+region_type:PA+(rdist|region_code3), family=binomial, data=data_tobaccor_subset) summary(model95)

model96<-

glmer(landuse~scale(trans)+scale(elevation)+region_type+PA+scale(trans):region_type+scale(el evation):region_type+region_type:PA+scale(elevation):PA+(rdist|region_code3), family=binomial, data=data_tobaccor_subset) summary(model96)

###try five interactions### model97<-

glmer(landuse~scale(agri)+scale(trans)+region_type+PA+scale(agri):region_type+scale(trans):re gion_type+scale(agri):PA+scale(trans):PA+region_type:PA+(rdist|region_code3), family=binomial, data=data_tobaccor_subset) summary(model97)

model98<-

glmer(landuse~scale(agri)+scale(elevation)+region_type+PA+scale(agri):region_type+scale(elev ation):region_type+scale(agri):PA+scale(elevation):PA+region_type:PA+(rdist|region_code3), family=binomial, data=data_tobaccor_subset) summary(model98)

model99<-

glmer(landuse~scale(trans)+scale(elevation)+region_type+PA+scale(trans):region_type+scale(el evation):region_type+scale(trans):PA+scale(elevation):PA+region_type:PA+(rdist|region_code3

), family=binomial, data=data_tobaccor_subset) summary(model99)

##select the best model library(MuMIn)

models_sel_tobaccor<-

model.sel(model_null,model1,model2,model3,model4,model5,model6,model7, model8, model9, model10, model11,model12, model13, model14,

model15,model16,model17,model18,model20,model21,model22,model23,model24,model27,mo del28, model41,model42,model59,rank="AIC") sink(file="models_sel_tobaccor.txt")

models_sel_tobaccor

sink()

##dignostic plots library(arm) binnedplot(predict(model41, type="response"), resid(model41, type="response"))

library(effects) plot(allEffects(model41)) allEffects(model41)

sink(file="alleffects(model41).txt") allEffects(model41) sink()

sink("summary(model41).txt") summary(model41)

sink()

####################prediction with actual yield ###############################

#1. Model baseline scenario, just put the actual yield in pred_tobaccor <- predict(model41, data_tobaccor_subset, re.form=NULL, type="response") head(pred_tobaccor)

write.csv(pred_tobaccor, "pred_tobaccor.csv")

##########################prediction with potential yield ###################### ##get potential yield data

data_all<-read.csv("Appendix_S4_Supplementary_Data.csv",header=T)

data_tobacco<-data_all[c(186505:209817),]

data_tobacco_subset<-data_tobacco[data_tobacco$land_use!=2,]

data_tobacco_subset$land_use <- as.factor(data_tobacco_subset$land_use) data_tobacco_subset$region_code1 <- as.factor(data_tobacco_subset$region_code1) data_tobacco_subset$region_code2 <- as.factor(data_tobacco_subset$region_code2) data_tobacco_subset$region_code3 <- as.factor(data_tobacco_subset$region_code3) data_tobacco_subset$resid<-as.factor(data_tobacco_subset$resid) data_tobacco_subset$PA<- as.factor(data_tobacco_subset$PA)

colnames(data_tobacco_subset) <- c("landuse", "agri", "trans", "elevation",

"region_type","region_code1","region_code2","region_code3","rdist", "resid","PA","ID") head(data_tobacco_subset)

#2. Scenario: actual yield being replaced by potential yield data pred_tobacco <- predict(model41, data_tobacco_subset, re.form=NULL, type="response") head(pred_tobacco)

write.csv(pred_tobacco, "pred_tobacco.csv")

#3. Scenario A: agr increase 50%

tobacco_y <- cbind(data_tobacco_subset$landuse, (data_tobacco_subset$agri)*1.5 , data_tobacco_subset[,3:12])

colnames(tobacco_y) <- c("landuse", "agri", "trans","elevation","region_type","region_code1","region_code2","region_code3","rdist","resid","PA","ID")

summary(tobacco_y)

pred_tobacco<-predict(model41, tobacco_y, re.form=NULL, type="response"))

write.csv(pred_tobacco, "pred_tobacco_agri.csv")

#4. Scenario B: transport costs increase by 35%

tobacco_trans_y <- cbind(data_tobacco_subset[,1:2], data_tobacco_subset[,3]*1.35, data_tobacco_subset[,4:12]) colnames(tobacco_trans_y) <- c("landuse", "agri",

"trans","elevation","region_type","region_code1","region_code2","region_code3","rdist","resid",

"PA","ID") summary(tobacco_trans_y)

pred_trans_tobacco<-predict(model41, tobacco_trans_y, re.form=NULL, type="response")

write.csv(pred_trans_tobacco, "pred_tobacco_trans.csv")

#5. Scenario C: political stability has total conversion

region_tobacco <- data_tobacco_subset

summary(region_tobacco) region_tobacco[,5] <- "Division"

region_tobacco[,5] <- as.factor(region_tobacco[,5])

pred_region_tobacco <- predict(model41, region_tobacco, re.form=NULL, type="response") head(pred_region_tobacco)

write.csv(pred_region_tobacco, "pred_tobacco_stability.csv")

#6. Scenario D: worst case. agri increase 50%, and stability has total conversion tobacco_worstcase<-tobacco_y

tobacco_worstcase[,5]<-"Division"

pred_worstcase_tobacco<-predict(model41, tobacco_worstcase, re.form=NULL, type="response")

write.csv(pred_worstcase_tobacco, "pred_tobacco_worstcase.csv")

rm(list=ls())

############################################################################## #################model land use with characteristics of groundnut######################

setwd("D:\\Myanmar project\\Resubmission 2\\Code and data (submit)")

data_all_raman<-read.csv("Appendix_S3_Supplementary_Data.csv",header=T)

data_groundnutr<-data_all_raman[c(209818:233130),]

data_groundnutr_subset<-data_groundnutr[data_groundnutr$land_use!=2,]

#convert to factors data_groundnutr_subset$land_use <- as.factor(data_groundnutr_subset$land_use) data_groundnutr_subset$region_code1 <- as.factor(data_groundnutr_subset$region_code1) data_groundnutr_subset$region_code2 <- as.factor(data_groundnutr_subset$region_code2) data_groundnutr_subset$region_code3 <- as.factor(data_groundnutr_subset$region_code3) data_groundnutr_subset$resid<-as.factor(data_groundnutr_subset$resid) data_groundnutr_subset$PA<- as.factor(data_groundnutr_subset$PA) colnames(data_groundnutr_subset) <- c("landuse", "agri", "trans", "elevation",

"region_type","region_code1","region_code2","region_code3","rdist", "resid","PA","ID") head(data_groundnutr_subset)

library(lme4)

##check collinearity

library(car)

model_vif <- glm(landuse ~ scale(agri)+scale(trans)+scale(elevation) +region_type+PA, family=binomial, data=data_groundnutr_subset) summary(model_vif)

vif(model_vif)

###try to fix the random effects first

model_r1 <- glmer(landuse ~ scale(agri)+scale(trans)+scale(elevation) +region_type+PA+

(1|region_code1), family=binomial, data=data_groundnutr_subset) summary(model_r1)

model_r2 <- glmer(landuse ~ scale(agri)+scale(trans) +scale(elevation)+region_type+PA+

(1|region_code2), family=binomial, data=data_groundnutr_subset) summary(model_r2)

model_r3 <- glmer(landuse ~ scale(agri)+scale(trans)+scale(elevation) +region_type+PA+

(1|region_code3), family=binomial, data=data_groundnutr_subset) summary(model_r3)

AIC(model_r1, model_r2, model_r3)

#choose region_code3 with smallest AIC

model_r4 <- glmer(landuse ~ scale(agri)+scale(trans)+scale(elevation) +region_type+PA+

(rdist|region_code3), family=binomial, data=data_groundnutr_subset) summary(model_r4)

###one variable###

model_null<-glmer(landuse~1+(rdist|region_code3), family=binomial, data=data_groundnutr_subset) summary(model_null)

model1<-glmer(landuse~scale(agri)+(rdist|region_code3), family=binomial, data=data_groundnutr_subset) summary(model1)

model2<-glmer(landuse~scale(trans)+(rdist|region_code3), family=binomial, data=data_groundnutr_subset) summary(model2)

model3<-glmer(landuse~scale(elevation)+(rdist|region_code3), family=binomial, data=data_groundnutr_subset) summary(model3)

model4<-glmer(landuse~region_type+(rdist|region_code3), family=binomial, data=data_groundnutr_subset) summary(model4)

model5<-glmer(landuse~PA+(rdist|region_code3), family=binomial, data=data_groundnutr_subset) summary(model5)

###two variables### model6<-glmer(landuse~scale(agri)+scale(trans)+(rdist|region_code3), family=binomial, data=data_groundnutr_subset) summary(model6)

model7<-glmer(landuse~scale(agri)+scale(elevation)+(rdist|region_code3), family=binomial, data=data_groundnutr_subset) summary(model7)

model8<-glmer(landuse~scale(agri)+region_type+(rdist|region_code3), family=binomial, data=data_groundnutr_subset) summary(model8)

model9<-glmer(landuse~scale(trans)+region_type+(rdist|region_code3), family=binomial, data=data_groundnutr_subset) summary(model9)

model10<-glmer(landuse~scale(elevation)+region_type+(rdist|region_code3), family=binomial, data=data_groundnutr_subset) summary(model10)

model11<-glmer(landuse~scale(trans)+scale(elevation)+(rdist|region_code3), family=binomial, data=data_groundnutr_subset) summary(model11)

model12<-glmer(landuse~scale(agri)+PA+(rdist|region_code3), family=binomial, data=data_groundnutr_subset) summary(model12)

model13<-glmer(landuse~scale(trans)+PA+(rdist|region_code3), family=binomial, data=data_groundnutr_subset) summary(model13) model14<-glmer(landuse~scale(elevation)+PA+(rdist|region_code3), family=binomial, data=data_groundnutr_subset) summary(model14)

model15<-glmer(landuse~region_type+PA+(rdist|region_code3), family=binomial, data=data_groundnutr_subset) summary(model15)

###three variables###

model16<-glmer(landuse~scale(agri)+scale(trans)+region_type+(rdist|region_code3), family=binomial, data=data_groundnutr_subset) summary(model16)

model17<-glmer(landuse~scale(agri)+scale(elevation)+region_type+(rdist|region_code3), family=binomial, data=data_groundnutr_subset) summary(model17)

model18<-glmer(landuse~scale(agri)+scale(trans)+scale(elevation)+(rdist|region_code3), family=binomial, data=data_groundnutr_subset) summary(model18)

model19<-glmer(landuse~scale(trans)+scale(elevation)+region_type+(rdist|region_code3), family=binomial, data=data_groundnutr_subset) summary(model19)

model20<-glmer(landuse~scale(agri)+scale(trans)+PA+(rdist|region_code3), family=binomial, data=data_groundnutr_subset)

summary(model20)

model21<-glmer(landuse~scale(agri)+scale(elevation)+PA+(rdist|region_code3), family=binomial, data=data_groundnutr_subset) summary(model21)

model22<-glmer(landuse~scale(agri)+region_type+PA+(rdist|region_code3), family=binomial, data=data_groundnutr_subset) summary(model22)

model23<-glmer(landuse~scale(trans)+scale(elevation)+PA+(rdist|region_code3), family=binomial, data=data_groundnutr_subset) summary(model23)

model24<-glmer(landuse~scale(trans)+region_type+PA+(rdist|region_code3), family=binomial, data=data_groundnutr_subset) summary(model24)

model25<-glmer(landuse~scale(elevation)+region_type+PA+(rdist|region_code3), family=binomial, data=data_groundnutr_subset) summary(model25)

###four variables###

model26<-

glmer(landuse~scale(agri)+scale(trans)+scale(elevation)+region_type+(rdist|region_code3), family=binomial, data=data_groundnutr_subset) summary(model26)

model27<-glmer(landuse~scale(agri)+scale(trans)+scale(elevation)+PA+(rdist|region_code3), family=binomial, data=data_groundnutr_subset)

summary(model27)

model28<-glmer(landuse~scale(agri)+scale(trans)+PA+region_type+(rdist|region_code3), family=binomial, data=data_groundnutr_subset) summary(model28)

model29<-glmer(landuse~scale(agri)+PA+scale(elevation)+region_type+(rdist|region_code3), family=binomial, data=data_groundnutr_subset) summary(model29)

model30<-glmer(landuse~PA+scale(trans)+scale(elevation)+region_type+(rdist|region_code3), family=binomial, data=data_groundnutr_subset) summary(model30)

###five variables###

model31<-glmer(landuse ~ scale(agri)+scale(trans)+scale(elevation)

+region_type+PA+(rdist|region_code3), family=binomial, data=data_groundnutr_subset)

###one interaction###

model32<-

glmer(landuse~scale(agri)+scale(trans)+scale(elevation)+region_type+scale(agri):region_type+(r dist|region_code3), family=binomial, data=data_groundnutr_subset) summary(model32)

model33<-

glmer(landuse~scale(agri)+scale(trans)+scale(elevation)+region_type+scale(trans):region_type+

(rdist|region_code3), family=binomial, data=data_groundnutr_subset) summary(model33)

model34<-

glmer(landuse~scale(agri)+scale(trans)+scale(elevation)+region_type+scale(elevation):region_ty pe+(rdist|region_code3), family=binomial, data=data_groundnutr_subset) summary(model34)

model35<-

glmer(landuse~scale(agri)+scale(trans)+scale(elevation)+PA+scale(agri):PA+(rdist|region_code

3), family=binomial, data=data_groundnutr_subset) summary(model35)

model36<-

glmer(landuse~scale(agri)+scale(trans)+region_type+PA+scale(agri):PA+(rdist|region_code3), family=binomial, data=data_groundnutr_subset) summary(model36)

model37<-

glmer(landuse~scale(agri)+scale(elevation)+region_type+PA+scale(agri):PA+(rdist|region_code

3), family=binomial, data=data_groundnutr_subset) summary(model37)

model38<-

glmer(landuse~scale(agri)+scale(trans)+scale(elevation)+PA+scale(trans):PA+(rdist|region_code

3), family=binomial, data=data_groundnutr_subset) summary(model38)

model39<-

glmer(landuse~scale(agri)+scale(trans)+region_type+PA+scale(trans):PA+(rdist|region_code3), family=binomial, data=data_groundnutr_subset) summary(model39)

model40<-

glmer(landuse~scale(trans)+scale(elevation)+region_type+PA+scale(trans):PA+(rdist|region_co de3), family=binomial, data=data_groundnutr_subset) summary(model40)

model41<-

glmer(landuse~scale(agri)+scale(trans)+scale(elevation)+PA+scale(elevation):PA+(rdist|region_ code3), family=binomial, data=data_groundnutr_subset) summary(model41)

model42<-

glmer(landuse~scale(agri)+scale(elevation)+region_type+PA+scale(elevation):PA+(rdist|region_ code3), family=binomial, data=data_groundnutr_subset) summary(model42)

model43<-

glmer(landuse~scale(trans)+scale(elevation)+region_type+PA+scale(elevation):PA+(rdist|region

_code3), family=binomial, data=data_groundnutr_subset) summary(model43)

model44<-

glmer(landuse~scale(agri)+scale(trans)+region_type+PA+region_type:PA+(rdist|region_code3), family=binomial, data=data_groundnutr_subset) summary(model44)

model45<-

glmer(landuse~scale(agri)+scale(elevation)+region_type+PA+region_type:PA+(rdist|region_cod e3), family=binomial, data=data_groundnutr_subset) summary(model45)

model46<-

glmer(landuse~scale(trans)+scale(elevation)+region_type+PA+region_type:PA+(rdist|region_co de3), family=binomial, data=data_groundnutr_subset) summary(model46)

###two interactions###

## with region

model47<-

glmer(landuse~scale(agri)+scale(trans)+scale(elevation)+region_type+scale(agri):region_type+s cale(trans):region_type+(rdist|region_code3), family=binomial, data=data_groundnutr_subset) summary(model47)

model48<-

glmer(landuse~scale(agri)+scale(trans)+scale(elevation)+region_type+scale(agri):region_type+s cale(elevation):region_type+(rdist|region_code3), family=binomial, data=data_groundnutr_subset) summary(model48)

model49<-

glmer(landuse~scale(agri)+scale(trans)+scale(elevation)+region_type+scale(trans):region_type+ scale(elevation):region_type+(rdist|region_code3), family=binomial, data=data_groundnutr_subset) summary(model49)

##with PA model50<-

glmer(landuse~scale(agri)+scale(trans)+scale(elevation)+PA+scale(agri):PA+scale(trans):PA+(r dist|region_code3), family=binomial, data=data_groundnutr_subset) summary(model50)

model51<-

glmer(landuse~scale(agri)+scale(trans)+region_type+PA+scale(agri):PA+scale(trans):PA+(rdist| region_code3), family=binomial, data=data_groundnutr_subset) summary(model51)

model52<-

glmer(landuse~scale(agri)+scale(trans)+scale(elevation)+PA+scale(agri):PA+scale(elevation):P

A+(rdist|region_code3), family=binomial, data=data_groundnutr_subset) summary(model52)

model53<-

glmer(landuse~scale(agri)+scale(elevation)+region_type+PA+scale(agri):PA+scale(elevation):P

A+(rdist|region_code3), family=binomial, data=data_groundnutr_subset) summary(model53)

model54<-

glmer(landuse~scale(agri)+scale(trans)+region_type+PA+scale(agri):PA+region_type:PA+(rdist| region_code3), family=binomial, data=data_groundnutr_subset) summary(model54)

model55<-

glmer(landuse~scale(agri)+scale(elevation)+region_type+PA+scale(agri):PA+region_type:PA+( rdist|region_code3), family=binomial, data=data_groundnutr_subset) summary(model55)

model56<-

glmer(landuse~scale(agri)+scale(trans)+scale(elevation)+PA+scale(trans):PA+scale(elevation):P

A+(rdist|region_code3), family=binomial, data=data_groundnutr_subset) summary(model56)

model57<glmer(landuse~scale(trans)+scale(elevation)+region_type+PA+scale(trans):PA+scale(elevation):

PA+(rdist|region_code3), family=binomial, data=data_groundnutr_subset)

summary(model57)

model58<-

glmer(landuse~scale(agri)+scale(trans)+region_type+PA+scale(trans):PA+region_type:PA+(rdis t|region_code3), family=binomial, data=data_groundnutr_subset) summary(model58)

model59<-

glmer(landuse~scale(trans)+scale(elevation)+region_type+PA+scale(trans):PA+region_type:PA

+(rdist|region_code3), family=binomial, data=data_groundnutr_subset) summary(model59)

##combine two sets model60<-

glmer(landuse~scale(agri)+scale(trans)+region_type+PA+scale(agri):region_type+scale(agri):P A+(rdist|region_code3), family=binomial, data=data_groundnutr_subset) summary(model60)

model61<-

glmer(landuse~scale(agri)+scale(elevation)+region_type+PA+scale(agri):region_type+scale(agri

):PA+(rdist|region_code3), family=binomial, data=data_groundnutr_subset) summary(model61)

model62<-

glmer(landuse~scale(agri)+scale(trans)+region_type+PA+scale(agri):region_type+scale(trans):P

A+(rdist|region_code3), family=binomial, data=data_groundnutr_subset) summary(model62)

model63<-

glmer(landuse~scale(agri)+scale(elevation)+region_type+PA+scale(agri):region_type+scale(elev ation):PA+(rdist|region_code3), family=binomial, data=data_groundnutr_subset) summary(model63)

model64<-

glmer(landuse~scale(agri)+scale(trans)+region_type+PA+scale(agri):region_type+region_type:P

A+(rdist|region_code3), family=binomial, data=data_groundnutr_subset) summary(model64)

model65<-

glmer(landuse~scale(agri)+scale(elevation)+region_type+PA+scale(agri):region_type+region_ty pe:PA+(rdist|region_code3), family=binomial, data=data_groundnutr_subset) summary(model65)

model66<-

glmer(landuse~scale(agri)+scale(trans)+region_type+PA+scale(trans):region_type+scale(agri):P

A+(rdist|region_code3), family=binomial, data=data_groundnutr_subset) summary(model66)

model67<glmer(landuse~scale(agri)+scale(trans)+region_type+PA+scale(trans):region_type+scale(trans): PA+(rdist|region_code3), family=binomial, data=data_groundnutr_subset) summary(model67)

model68<-

glmer(landuse~scale(elevation)+scale(trans)+region_type+PA+scale(trans):region_type+scale(tr ans):PA+(rdist|region_code3), family=binomial, data=data_groundnutr_subset) summary(model68)

model69<-

glmer(landuse~scale(trans)+scale(elevation)+region_type+PA+scale(trans):region_type+scale(el evation):PA+(rdist|region_code3), family=binomial, data=data_groundnutr_subset) summary(model69)

model70<glmer(landuse~scale(agri)+scale(trans)+region_type+PA+scale(trans):region_type+region_type: PA+(rdist|region_code3), family=binomial, data=data_groundnutr_subset) summary(model70)

model71<-

glmer(landuse~scale(trans)+scale(elevation)+region_type+PA+scale(trans):region_type+region_ type:PA+(rdist|region_code3), family=binomial, data=data_groundnutr_subset) summary(model71)

model72<-

glmer(landuse~scale(agri)+scale(elevation)+region_type+PA+scale(elevation):region_type+scal e(agri):PA+(rdist|region_code3), family=binomial, data=data_groundnutr_subset) summary(model72)

model73<-

glmer(landuse~scale(trans)+scale(elevation)+region_type+PA+scale(elevation):region_type+sca le(trans):PA+(rdist|region_code3), family=binomial, data=data_groundnutr_subset) summary(model73)

model74<-

glmer(landuse~scale(agri)+scale(elevation)+region_type+PA+scale(elevation):region_type+scal e(elevation):PA+(rdist|region_code3), family=binomial, data=data_groundnutr_subset) summary(model74)

model75<-

glmer(landuse~scale(trans)+scale(elevation)+region_type+PA+scale(elevation):region_type+sca le(elevation):PA+(rdist|region_code3), family=binomial, data=data_groundnutr_subset) summary(model75)

model76<-

glmer(landuse~scale(agri)+scale(elevation)+region_type+PA+scale(elevation):region_type+regi on_type:PA+(rdist|region_code3), family=binomial, data=data_groundnutr_subset) summary(model76)

model77<-

glmer(landuse~scale(trans)+scale(elevation)+region_type+PA+scale(elevation):region_type+reg ion_type:PA+(rdist|region_code3), family=binomial, data=data_groundnutr_subset) summary(model77)

###three interactions###

model78<-

glmer(landuse~scale(agri)+scale(trans)+scale(elevation)+region_type+scale(agri):region_type+s cale(elevation):region_type+scale(trans):region_type+(rdist|region_code3), family=binomial, data=data_groundnutr_subset) summary(model78)

model79<-

glmer(landuse~scale(agri)+scale(trans)+region_type+PA+scale(agri):region_type+scale(agri):P

A+scale(trans):region_type+(rdist|region_code3), family=binomial, data=data_groundnutr_subset) summary(model79)

model80<-

glmer(landuse~scale(agri)+scale(trans)+region_type+PA+scale(agri):region_type+scale(trans):P

A+scale(trans):region_type+(rdist|region_code3), family=binomial, data=data_groundnutr_subset) summary(model80)

model81<-

glmer(landuse~scale(agri)+scale(trans)+region_type+PA+scale(agri):region_type+region_type:P

A+scale(trans):region_type+(rdist|region_code3), family=binomial, data=data_groundnutr_subset) summary(model81)

model82<-

glmer(landuse~scale(agri)+scale(elevation)+region_type+PA+scale(agri):region_type+scale(agri

):PA+scale(elevation):region_type+(rdist|region_code3), family=binomial, data=data_groundnutr_subset) summary(model82)

model83<-

glmer(landuse~scale(agri)+scale(elevation)+region_type+PA+scale(agri):region_type+scale(elev ation):PA+scale(elevation):region_type+(rdist|region_code3), family=binomial, data=data_groundnutr_subset) summary(model83)

model84<-

glmer(landuse~scale(agri)+scale(elevation)+region_type+PA+scale(agri):region_type+region_ty pe:PA+scale(elevation):region_type+(rdist|region_code3), family=binomial, data=data_groundnutr_subset) summary(model84)

model85<-

glmer(landuse~scale(trans)+scale(elevation)+region_type+PA+scale(trans):region_type+scale(tr ans):PA+scale(elevation):region_type+(rdist|region_code3), family=binomial, data=data_groundnutr_subset) summary(model85)

model86<-

glmer(landuse~scale(trans)+scale(elevation)+region_type+PA+scale(trans):region_type+scale(el evation):PA+scale(elevation):region_type+(rdist|region_code3), family=binomial, data=data_groundnutr_subset) summary(model86)

model87<-

glmer(landuse~scale(trans)+scale(elevation)+region_type+PA+scale(trans):region_type+region_ type:PA+scale(elevation):region_type+(rdist|region_code3), family=binomial, data=data_groundnutr_subset)

summary(model87)

###four interactions### model88<-

glmer(landuse~scale(agri)+scale(trans)+region_type+PA+scale(agri):region_type+scale(trans):re gion_type+scale(agri):PA+scale(trans):PA+(rdist|region_code3), family=binomial, data=data_groundnutr_subset) summary(model88)

model89<-

glmer(landuse~scale(agri)+scale(trans)+region_type+PA+scale(agri):region_type+scale(trans):re gion_type+scale(agri):PA+region_type:PA+(rdist|region_code3), family=binomial, data=data_groundnutr_subset) summary(model89)

model90<-

glmer(landuse~scale(agri)+scale(trans)+region_type+PA+scale(agri):region_type+scale(trans):re gion_type+scale(trans):PA+region_type:PA+(rdist|region_code3), family=binomial, data=data_groundnutr_subset) summary(model90)

model91<-

glmer(landuse~scale(agri)+scale(elevation)+region_type+PA+scale(agri):region_type+scale(elev ation):region_type+scale(agri):PA+scale(elevation):PA+(rdist|region_code3), family=binomial, data=data_groundnutr_subset) summary(model91)

model92<-

glmer(landuse~scale(agri)+scale(elevation)+region_type+PA+scale(agri):region_type+scale(elev ation):region_type+scale(agri):PA+region_type:PA+(rdist|region_code3), family=binomial, data=data_groundnutr_subset) summary(model92)

model93<-

glmer(landuse~scale(agri)+scale(elevation)+region_type+PA+scale(agri):region_type+scale(elev ation):region_type+region_type:PA+scale(elevation):PA+(rdist|region_code3), family=binomial, data=data_groundnutr_subset) summary(model93)

model94<-

glmer(landuse~scale(trans)+scale(elevation)+region_type+PA+scale(trans):region_type+scale(el evation):region_type+scale(trans):PA+scale(elevation):PA+(rdist|region_code3), family=binomial, data=data_groundnutr_subset) summary(model94)

model95<-

glmer(landuse~scale(trans)+scale(elevation)+region_type+PA+scale(trans):region_type+scale(el evation):region_type+scale(trans):PA+region_type:PA+(rdist|region_code3), family=binomial, data=data_groundnutr_subset) summary(model95)

model96<-

glmer(landuse~scale(trans)+scale(elevation)+region_type+PA+scale(trans):region_type+scale(el evation):region_type+region_type:PA+scale(elevation):PA+(rdist|region_code3), family=binomial, data=data_groundnutr_subset) summary(model96)

###try five interactions### model97<-

glmer(landuse~scale(agri)+scale(trans)+region_type+PA+scale(agri):region_type+scale(trans):re gion_type+scale(agri):PA+scale(trans):PA+region_type:PA+(rdist|region_code3), family=binomial, data=data_groundnutr_subset) summary(model97)

model98<-

glmer(landuse~scale(agri)+scale(elevation)+region_type+PA+scale(agri):region_type+scale(elev ation):region_type+scale(agri):PA+scale(elevation):PA+region_type:PA+(rdist|region_code3), family=binomial, data=data_groundnutr_subset) summary(model98)

model99<-

glmer(landuse~scale(trans)+scale(elevation)+region_type+PA+scale(trans):region_type+scale(el evation):region_type+scale(trans):PA+scale(elevation):PA+region_type:PA+(rdist|region_code3

), family=binomial, data=data_groundnutr_subset) summary(model99)

##Select the best model

library(MuMIn)

models_sel_groundnutr<-

model.sel(model_null,model1,model2,model3,model4,model5,model6,model7, model8, model9, model10, model11,model12, model13, model14,

model15,model16,model17,model18,model19,model20,model21,model22,model23,model24,mo del26,model27,model28,model29,

model40,rank="AIC") sink(file="models_sel_groundnutr.txt")

models_sel_groundnutr

sink()

##dignostic plot library(arm) binnedplot(predict(model27, type="response"), resid(model27, type="response")) library(effects) plot(allEffects(model27)) allEffects(model27)

sink(file="alleffects(model27).txt") allEffects(model27) sink()

sink("summary(model27).txt") summary(model27) sink()

####################prediction with actual yield ###############################

#1.Model baseline scenario, just put the actual yield in pred_groundnutr <- predict(model27, data_groundnutr_subset, re.form=NULL, type="response") head(pred_groundnutr)

write.csv(pred_groundnutr, "pred_groundnutr.csv")

##########################prediction with potential yield ######################

##get potential yield data data_all<-read.csv("Appendix_S4_Supplementary_Data.csv",header=T)

data_groundnut<-data_all[c(209818:233130),]

data_groundnut_subset<-data_groundnut[data_groundnut$land_use!=2,] data_groundnut_subset$land_use <- as.factor(data_groundnut_subset$land_use) data_groundnut_subset$region_code1 <- as.factor(data_groundnut_subset$region_code1) data_groundnut_subset$region_code2 <- as.factor(data_groundnut_subset$region_code2) data_groundnut_subset$region_code3 <- as.factor(data_groundnut_subset$region_code3) data_groundnut_subset$resid<-as.factor(data_groundnut_subset$resid) data_groundnut_subset$PA<- as.factor(data_groundnut_subset$PA)

colnames(data_groundnut_subset) <- c("landuse", "agri", "trans", "elevation",

"region_type","region_code1","region_code2","region_code3","rdist", "resid","PA","ID") head(data_groundnut_subset)

#2. Scenario: actual yield being replaced by potential yield data pred_groundnut <- predict(model27, data_groundnut_subset, re.form=NULL, type="response") head(pred_groundnut)

write.csv(pred_groundnut, "pred_groundnut.csv")

#3. Scenario A: agr increase 50%

groundnut_y <- cbind(data_groundnut_subset$landuse, (data_groundnut_subset$agri)*1.5 , data_groundnut_subset[,3:12])

colnames(groundnut_y) <- c("landuse", "agri", "trans","elevation","region_type","region_code1","region_code2","region_code3","rdist","resid","PA","ID")

summary(groundnut_y)

pred_groundnut<-predict(model27, groundnut_y, re.form=NULL, type="response"))

write.csv(pred_groundnut, "pred_groundnut_agri.csv")

#4. Scenario B: transport costs increase by 35%

groundnut_trans_y <- cbind(data_groundnut_subset[,1:2], data_groundnut_subset[,3]*1.35, data_groundnut_subset[,4:12]) colnames(groundnut_trans_y) <- c("landuse", "agri",

"trans","elevation","region_type","region_code1","region_code2","region_code3","rdist","resid",

"PA","ID") summary(groundnut_trans_y)

pred_trans_groundnut<-predict(model27, groundnut_trans_y, re.form=NULL, type="response")

write.csv(pred_trans_groundnut, "pred_groundnut_trans.csv")

#5. Scenario C: political stability has total conversion

region_groundnut <- data_groundnut_subset

summary(region_groundnut) region_groundnut[,5] <- "Division"

region_groundnut[,5] <- as.factor(region_groundnut[,5])

pred_region_groundnut <- predict(model27, region_groundnut, re.form=NULL, type="response") head(pred_region_groundnut)

write.csv(pred_region_groundnut, "pred_groundnut_stability.csv")

#6. Scenario D: worst case. agri increase 50%, and stability has total conversion groundnut_worstcase<-groundnut_y

groundnut_worstcase[,5]<-"Division"

pred_worstcase_groundnut<-predict(model27, groundnut_worstcase, re.form=NULL, type="response")

write.csv(pred_worstcase_groundnut, "pred_groundnut_worstcase.csv")

rm(list=ls())

##############################################################################

##################model land use with characteristics of sesame#######################

setwd("D:\\Myanmar project\\Resubmission 2\\Code and data (submit)")

data_all_raman<-read.csv("Appendix_S3_Supplementary_Data.csv",header=T)

data_sesame<-data_all_raman[c(233131:256443),] data_sesame_subset<-data_sesame[data_sesame$land_use!=2,]

#convert to factors data_sesame_subset$land_use <- as.factor(data_sesame_subset$land_use) data_sesame_subset$region_code1 <- as.factor(data_sesame_subset$region_code1) data_sesame_subset$region_code2 <- as.factor(data_sesame_subset$region_code2) data_sesame_subset$region_code3 <- as.factor(data_sesame_subset$region_code3) data_sesame_subset$resid<-as.factor(data_sesame_subset$resid) data_sesame_subset$PA<- as.factor(data_sesame_subset$PA)

colnames(data_sesame_subset) <- c("landuse", "agri", "trans", "elevation",

"region_type","region_code1","region_code2","region_code3","rdist", "resid","PA","ID") head(data_sesame_subset)

library(lme4)

##check collinearity

library(car)

model_vif <- glm(landuse ~ scale(agri)+scale(trans)+scale(elevation) +region_type+PA, family=binomial, data=data_sesamer_subset) summary(model_vif)

vif(model_vif)

###try to fix the random effects first

model_r1 <- glmer(landuse ~ scale(agri)+scale(trans)+scale(elevation) +region_type+PA+

(1|region_code1), family=binomial, data=data_sesame_subset) summary(model_r1)

model_r2 <- glmer(landuse ~ scale(agri)+scale(trans) +scale(elevation)+region_type+PA+

(1|region_code2), family=binomial, data=data_sesame_subset) summary(model_r2)

model_r3 <- glmer(landuse ~ scale(agri)+scale(trans)+scale(elevation) +region_type+PA+

(1|region_code3), family=binomial, data=data_sesame_subset) summary(model_r3)

AIC(model_r1, model_r2, model_r3)

model_r4 <- glmer(landuse ~ scale(agri)+scale(trans)+scale(elevation) +region_type+PA+

(rdist|region_code3), family=binomial, data=data_sesame_subset) summary(model_r4)

AIC(model_r3,model_r4)

##choose model_r4 with samllest AIC ############################################################################

###one variable### model_null<-glmer(landuse~1+(rdist|region_code3), family=binomial, data=data_sesame_subset) summary(model_null)

model1<-glmer(landuse~scale(agri)+(rdist|region_code3), family=binomial, data=data_sesame_subset) summary(model1)

model2<-glmer(landuse~scale(trans)+(rdist|region_code3), family=binomial, data=data_sesame_subset) summary(model2)

model3<-glmer(landuse~scale(elevation)+(rdist|region_code3), family=binomial, data=data_sesame_subset) summary(model3)

model4<-glmer(landuse~region_type+(rdist|region_code3), family=binomial, data=data_sesame_subset) summary(model4)

model5<-glmer(landuse~PA+(rdist|region_code3), family=binomial, data=data_sesame_subset) summary(model5)

###two variables###

model6<-glmer(landuse~scale(agri)+scale(trans)+(rdist|region_code3), family=binomial, data=data_sesame_subset) summary(model6)

model7<-glmer(landuse~scale(agri)+scale(elevation)+(rdist|region_code3), family=binomial, data=data_sesame_subset) summary(model7)

model8<-glmer(landuse~scale(agri)+region_type+(rdist|region_code3), family=binomial, data=data_sesame_subset) summary(model8)

model9<-glmer(landuse~scale(trans)+region_type+(rdist|region_code3), family=binomial, data=data_sesame_subset) summary(model9)

model10<-glmer(landuse~scale(elevation)+region_type+(rdist|region_code3), family=binomial, data=data_sesame_subset) summary(model10)

model11<-glmer(landuse~scale(trans)+scale(elevation)+(rdist|region_code3), family=binomial, data=data_sesame_subset) summary(model11)

model12<-glmer(landuse~scale(agri)+PA+(rdist|region_code3), family=binomial, data=data_sesame_subset) summary(model12)

model13<-glmer(landuse~scale(trans)+PA+(rdist|region_code3), family=binomial, data=data_sesame_subset) summary(model13) model14<-glmer(landuse~scale(elevation)+PA+(rdist|region_code3), family=binomial, data=data_sesame_subset) summary(model14)

model15<-glmer(landuse~region_type+PA+(rdist|region_code3), family=binomial, data=data_sesame_subset) summary(model15)

###three variables###

model16<-glmer(landuse~scale(agri)+scale(trans)+region_type+(rdist|region_code3), family=binomial, data=data_sesame_subset) summary(model16)

model17<-glmer(landuse~scale(agri)+scale(elevation)+region_type+(rdist|region_code3), family=binomial, data=data_sesame_subset) summary(model17)

model18<-glmer(landuse~scale(agri)+scale(trans)+scale(elevation)+(rdist|region_code3), family=binomial, data=data_sesame_subset) summary(model18)

model19<-glmer(landuse~scale(trans)+scale(elevation)+region_type+(rdist|region_code3), family=binomial, data=data_sesame_subset) summary(model19)

model20<-glmer(landuse~scale(agri)+scale(trans)+PA+(rdist|region_code3), family=binomial, data=data_sesame_subset)

summary(model20)

model21<-glmer(landuse~scale(agri)+scale(elevation)+PA+(rdist|region_code3), family=binomial, data=data_sesame_subset) summary(model21)

model22<-glmer(landuse~scale(agri)+region_type+PA+(rdist|region_code3), family=binomial, data=data_sesame_subset) summary(model22)

model23<-glmer(landuse~scale(trans)+scale(elevation)+PA+(rdist|region_code3), family=binomial, data=data_sesame_subset) summary(model23)

model24<-glmer(landuse~scale(trans)+region_type+PA+(rdist|region_code3), family=binomial, data=data_sesame_subset) summary(model24)

model25<-glmer(landuse~scale(elevation)+region_type+PA+(rdist|region_code3), family=binomial, data=data_sesame_subset) summary(model25)

###four variables###

model26<-

glmer(landuse~scale(agri)+scale(trans)+scale(elevation)+region_type+(rdist|region_code3), family=binomial, data=data_sesame_subset) summary(model26)

model27<-glmer(landuse~scale(agri)+scale(trans)+scale(elevation)+PA+(rdist|region_code3), family=binomial, data=data_sesame_subset)

summary(model27)

model28<-glmer(landuse~scale(agri)+scale(trans)+PA+region_type+(rdist|region_code3), family=binomial, data=data_sesame_subset) summary(model28)

model29<-glmer(landuse~scale(agri)+PA+scale(elevation)+region_type+(rdist|region_code3), family=binomial, data=data_sesame_subset) summary(model29)

model30<-glmer(landuse~PA+scale(trans)+scale(elevation)+region_type+(rdist|region_code3), family=binomial, data=data_sesame_subset) summary(model30)

###five variables###

model31<-glmer(landuse ~ scale(agri)+scale(trans)+scale(elevation)

+region_type+PA+(rdist|region_code3), family=binomial, data=data_sesame_subset)

###one interaction###

model32<-

glmer(landuse~scale(agri)+scale(trans)+scale(elevation)+region_type+scale(agri):region_type+(r dist|region_code3), family=binomial, data=data_sesame_subset) summary(model32)

model33<-

glmer(landuse~scale(agri)+scale(trans)+scale(elevation)+region_type+scale(trans):region_type+

(rdist|region_code3), family=binomial, data=data_sesame_subset) summary(model33)

model34<-

glmer(landuse~scale(agri)+scale(trans)+scale(elevation)+region_type+scale(elevation):region_ty pe+(rdist|region_code3), family=binomial, data=data_sesame_subset) summary(model34)

model35<-

glmer(landuse~scale(agri)+scale(trans)+scale(elevation)+PA+scale(agri):PA+(rdist|region_code

3), family=binomial, data=data_sesame_subset) summary(model35)

model36<-

glmer(landuse~scale(agri)+scale(trans)+region_type+PA+scale(agri):PA+(rdist|region_code3), family=binomial, data=data_sesame_subset) summary(model36)

model37<-

glmer(landuse~scale(agri)+scale(elevation)+region_type+PA+scale(agri):PA+(rdist|region_code

3), family=binomial, data=data_sesame_subset) summary(model37)

model38<-

glmer(landuse~scale(agri)+scale(trans)+scale(elevation)+PA+scale(trans):PA+(rdist|region_code

3), family=binomial, data=data_sesame_subset) summary(model38)

model39<-

glmer(landuse~scale(agri)+scale(trans)+region_type+PA+scale(trans):PA+(rdist|region_code3), family=binomial, data=data_sesame_subset) summary(model39)

model40<-

glmer(landuse~scale(trans)+scale(elevation)+region_type+PA+scale(trans):PA+(rdist|region_co de3), family=binomial, data=data_sesame_subset) summary(model40)

model41<-

glmer(landuse~scale(agri)+scale(trans)+scale(elevation)+PA+scale(elevation):PA+(rdist|region_ code3), family=binomial, data=data_sesame_subset) summary(model41)

model42<-

glmer(landuse~scale(agri)+scale(elevation)+region_type+PA+scale(elevation):PA+(rdist|region_ code3), family=binomial, data=data_sesame_subset) summary(model42)

model43<-

glmer(landuse~scale(trans)+scale(elevation)+region_type+PA+scale(elevation):PA+(rdist|region

_code3), family=binomial, data=data_sesame_subset) summary(model43)

model44<-

glmer(landuse~scale(agri)+scale(trans)+region_type+PA+region_type:PA+(rdist|region_code3), family=binomial, data=data_sesame_subset) summary(model44)

model45<-

glmer(landuse~scale(agri)+scale(elevation)+region_type+PA+region_type:PA+(rdist|region_cod e3), family=binomial, data=data_sesame_subset) summary(model45)

model46<-

glmer(landuse~scale(trans)+scale(elevation)+region_type+PA+region_type:PA+(rdist|region_co de3), family=binomial, data=data_sesame_subset) summary(model46)

###two interactions###

## with region

model47<-

glmer(landuse~scale(agri)+scale(trans)+scale(elevation)+region_type+scale(agri):region_type+s cale(trans):region_type+(rdist|region_code3), family=binomial, data=data_sesame_subset) summary(model47)

model48<-

glmer(landuse~scale(agri)+scale(trans)+scale(elevation)+region_type+scale(agri):region_type+s cale(elevation):region_type+(rdist|region_code3), family=binomial, data=data_sesame_subset) summary(model48)

model49<-

glmer(landuse~scale(agri)+scale(trans)+scale(elevation)+region_type+scale(trans):region_type+ scale(elevation):region_type+(rdist|region_code3), family=binomial, data=data_sesame_subset) summary(model49)

##with PA model50<-

glmer(landuse~scale(agri)+scale(trans)+scale(elevation)+PA+scale(agri):PA+scale(trans):PA+(r dist|region_code3), family=binomial, data=data_sesame_subset) summary(model50)

model51<-

glmer(landuse~scale(agri)+scale(trans)+region_type+PA+scale(agri):PA+scale(trans):PA+(rdist| region_code3), family=binomial, data=data_sesame_subset)

summary(model51)

model52<-

glmer(landuse~scale(agri)+scale(trans)+scale(elevation)+PA+scale(agri):PA+scale(elevation):P

A+(rdist|region_code3), family=binomial, data=data_sesame_subset) summary(model52)

model53<-

glmer(landuse~scale(agri)+scale(elevation)+region_type+PA+scale(agri):PA+scale(elevation):P

A+(rdist|region_code3), family=binomial, data=data_sesame_subset) summary(model53)

model54<-

glmer(landuse~scale(agri)+scale(trans)+region_type+PA+scale(agri):PA+region_type:PA+(rdist| region_code3), family=binomial, data=data_sesame_subset) summary(model54)

model55<-

glmer(landuse~scale(agri)+scale(elevation)+region_type+PA+scale(agri):PA+region_type:PA+( rdist|region_code3), family=binomial, data=data_sesame_subset) summary(model55)

model56<-

glmer(landuse~scale(agri)+scale(trans)+scale(elevation)+PA+scale(trans):PA+scale(elevation):P

A+(rdist|region_code3), family=binomial, data=data_sesame_subset) summary(model56)

model57<glmer(landuse~scale(trans)+scale(elevation)+region_type+PA+scale(trans):PA+scale(elevation): PA+(rdist|region_code3), family=binomial, data=data_sesame_subset) summary(model57)

model58<-

glmer(landuse~scale(agri)+scale(trans)+region_type+PA+scale(trans):PA+region_type:PA+(rdis t|region_code3), family=binomial, data=data_sesame_subset) summary(model58)

model59<-

glmer(landuse~scale(trans)+scale(elevation)+region_type+PA+scale(trans):PA+region_type:PA

+(rdist|region_code3), family=binomial, data=data_sesame_subset) summary(model59)

##combine two sets model60<-

glmer(landuse~scale(agri)+scale(trans)+region_type+PA+scale(agri):region_type+scale(agri):P

A+(rdist|region_code3), family=binomial, data=data_sesame_subset) summary(model60)

model61<-

glmer(landuse~scale(agri)+scale(elevation)+region_type+PA+scale(agri):region_type+scale(agri

):PA+(rdist|region_code3), family=binomial, data=data_sesame_subset) summary(model61)

model62<-

glmer(landuse~scale(agri)+scale(trans)+region_type+PA+scale(agri):region_type+scale(trans):P

A+(rdist|region_code3), family=binomial, data=data_sesame_subset) summary(model62)

model63<-

glmer(landuse~scale(agri)+scale(elevation)+region_type+PA+scale(agri):region_type+scale(elev ation):PA+(rdist|region_code3), family=binomial, data=data_sesame_subset) summary(model63)

model64<-

glmer(landuse~scale(agri)+scale(trans)+region_type+PA+scale(agri):region_type+region_type:P

A+(rdist|region_code3), family=binomial, data=data_sesame_subset) summary(model64)

model65<-

glmer(landuse~scale(agri)+scale(elevation)+region_type+PA+scale(agri):region_type+region_ty pe:PA+(rdist|region_code3), family=binomial, data=data_sesame_subset) summary(model65)

model66<-

glmer(landuse~scale(agri)+scale(trans)+region_type+PA+scale(trans):region_type+scale(agri):P

A+(rdist|region_code3), family=binomial, data=data_sesame_subset) summary(model66)

model67<glmer(landuse~scale(agri)+scale(trans)+region_type+PA+scale(trans):region_type+scale(trans): PA+(rdist|region_code3), family=binomial, data=data_sesame_subset) summary(model67)

model68<-

glmer(landuse~scale(elevation)+scale(trans)+region_type+PA+scale(trans):region_type+scale(tr ans):PA+(rdist|region_code3), family=binomial, data=data_sesame_subset) summary(model68)

model69<-

glmer(landuse~scale(trans)+scale(elevation)+region_type+PA+scale(trans):region_type+scale(el evation):PA+(rdist|region_code3), family=binomial, data=data_sesame_subset) summary(model69)

model70<glmer(landuse~scale(agri)+scale(trans)+region_type+PA+scale(trans):region_type+region_type:

PA+(rdist|region_code3), family=binomial, data=data_sesame_subset)

summary(model70)

model71<-

glmer(landuse~scale(trans)+scale(elevation)+region_type+PA+scale(trans):region_type+region_ type:PA+(rdist|region_code3), family=binomial, data=data_sesame_subset) summary(model71)

model72<-

glmer(landuse~scale(agri)+scale(elevation)+region_type+PA+scale(elevation):region_type+scal e(agri):PA+(rdist|region_code3), family=binomial, data=data_sesame_subset) summary(model72)

model73<-

glmer(landuse~scale(trans)+scale(elevation)+region_type+PA+scale(elevation):region_type+sca le(trans):PA+(rdist|region_code3), family=binomial, data=data_sesame_subset) summary(model73)

model74<-

glmer(landuse~scale(agri)+scale(elevation)+region_type+PA+scale(elevation):region_type+scal e(elevation):PA+(rdist|region_code3), family=binomial, data=data_sesame_subset) summary(model74)

model75<-

glmer(landuse~scale(trans)+scale(elevation)+region_type+PA+scale(elevation):region_type+sca le(elevation):PA+(rdist|region_code3), family=binomial, data=data_sesame_subset) summary(model75)

model76<-

glmer(landuse~scale(agri)+scale(elevation)+region_type+PA+scale(elevation):region_type+regi on_type:PA+(rdist|region_code3), family=binomial, data=data_sesame_subset) summary(model76)

model77<-

glmer(landuse~scale(trans)+scale(elevation)+region_type+PA+scale(elevation):region_type+reg ion_type:PA+(rdist|region_code3), family=binomial, data=data_sesame_subset) summary(model77)

###three interactions###

model78<-

glmer(landuse~scale(agri)+scale(trans)+scale(elevation)+region_type+scale(agri):region_type+s cale(elevation):region_type+scale(trans):region_type+(rdist|region_code3), family=binomial, data=data_sesame_subset) summary(model78)

model79<-

glmer(landuse~scale(agri)+scale(trans)+region_type+PA+scale(agri):region_type+scale(agri):P A+scale(trans):region_type+(rdist|region_code3), family=binomial, data=data_sesame_subset) summary(model79)

model80<-

glmer(landuse~scale(agri)+scale(trans)+region_type+PA+scale(agri):region_type+scale(trans):P A+scale(trans):region_type+(rdist|region_code3), family=binomial, data=data_sesame_subset) summary(model80)

model81<-

glmer(landuse~scale(agri)+scale(trans)+region_type+PA+scale(agri):region_type+region_type:P A+scale(trans):region_type+(rdist|region_code3), family=binomial, data=data_sesame_subset) summary(model81)

model82<-

glmer(landuse~scale(agri)+scale(elevation)+region_type+PA+scale(agri):region_type+scale(agri

):PA+scale(elevation):region_type+(rdist|region_code3), family=binomial, data=data_sesame_subset) summary(model82)

model83<-

glmer(landuse~scale(agri)+scale(elevation)+region_type+PA+scale(agri):region_type+scale(elev ation):PA+scale(elevation):region_type+(rdist|region_code3), family=binomial, data=data_sesame_subset) summary(model83)

model84<-

glmer(landuse~scale(agri)+scale(elevation)+region_type+PA+scale(agri):region_type+region_ty pe:PA+scale(elevation):region_type+(rdist|region_code3), family=binomial, data=data_sesame_subset) summary(model84)

model85<-

glmer(landuse~scale(trans)+scale(elevation)+region_type+PA+scale(trans):region_type+scale(tr ans):PA+scale(elevation):region_type+(rdist|region_code3), family=binomial, data=data_sesame_subset) summary(model85)

model86<-

glmer(landuse~scale(trans)+scale(elevation)+region_type+PA+scale(trans):region_type+scale(el evation):PA+scale(elevation):region_type+(rdist|region_code3), family=binomial, data=data_sesame_subset) summary(model86)

model87<-

glmer(landuse~scale(trans)+scale(elevation)+region_type+PA+scale(trans):region_type+region_ type:PA+scale(elevation):region_type+(rdist|region_code3), family=binomial, data=data_sesame_subset) summary(model87)

###four interactions### model88<-

glmer(landuse~scale(agri)+scale(trans)+region_type+PA+scale(agri):region_type+scale(trans):re gion_type+scale(agri):PA+scale(trans):PA+(rdist|region_code3), family=binomial, data=data_sesame_subset) summary(model88)

model89<-

glmer(landuse~scale(agri)+scale(trans)+region_type+PA+scale(agri):region_type+scale(trans):re gion_type+scale(agri):PA+region_type:PA+(rdist|region_code3), family=binomial, data=data_sesame_subset) summary(model89)

model90<-

glmer(landuse~scale(agri)+scale(trans)+region_type+PA+scale(agri):region_type+scale(trans):re gion_type+scale(trans):PA+region_type:PA+(rdist|region_code3), family=binomial, data=data_sesame_subset) summary(model90)

model91<-

glmer(landuse~scale(agri)+scale(elevation)+region_type+PA+scale(agri):region_type+scale(elev ation):region_type+scale(agri):PA+scale(elevation):PA+(rdist|region_code3), family=binomial, data=data_sesame_subset) summary(model91)

model92<-

glmer(landuse~scale(agri)+scale(elevation)+region_type+PA+scale(agri):region_type+scale(elev ation):region_type+scale(agri):PA+region_type:PA+(rdist|region_code3), family=binomial, data=data_sesame_subset) summary(model92)

model93<-

glmer(landuse~scale(agri)+scale(elevation)+region_type+PA+scale(agri):region_type+scale(elev ation):region_type+region_type:PA+scale(elevation):PA+(rdist|region_code3), family=binomial, data=data_sesame_subset) summary(model93)

model94<-

glmer(landuse~scale(trans)+scale(elevation)+region_type+PA+scale(trans):region_type+scale(el evation):region_type+scale(trans):PA+scale(elevation):PA+(rdist|region_code3), family=binomial, data=data_sesame_subset) summary(model94)

model95<-

glmer(landuse~scale(trans)+scale(elevation)+region_type+PA+scale(trans):region_type+scale(el evation):region_type+scale(trans):PA+region_type:PA+(rdist|region_code3), family=binomial, data=data_sesame_subset) summary(model95)

model96<-

glmer(landuse~scale(trans)+scale(elevation)+region_type+PA+scale(trans):region_type+scale(el evation):region_type+region_type:PA+scale(elevation):PA+(rdist|region_code3), family=binomial, data=data_sesame_subset) summary(model96)

###try five interactions### model97<-

glmer(landuse~scale(agri)+scale(trans)+region_type+PA+scale(agri):region_type+scale(trans):re gion_type+scale(agri):PA+scale(trans):PA+region_type:PA+(rdist|region_code3), family=binomial, data=data_sesame_subset) summary(model97)

model98<-

glmer(landuse~scale(agri)+scale(elevation)+region_type+PA+scale(agri):region_type+scale(elev ation):region_type+scale(agri):PA+scale(elevation):PA+region_type:PA+(rdist|region_code3), family=binomial, data=data_sesame_subset) summary(model98)

model99<-

glmer(landuse~scale(trans)+scale(elevation)+region_type+PA+scale(trans):region_type+scale(el evation):region_type+scale(trans):PA+scale(elevation):PA+region_type:PA+(rdist|region_code3

), family=binomial, data=data_sesame_subset) summary(model99)

##issue of quasi-perfect separation

rm(list=ls())

##############################################################################

#################model land use with characteristics of sunflower######################

setwd("D:\\Myanmar project\\Resubmission 2\\Code and data (submit)") data_all_raman<-read.csv("Appendix_S3_Supplementary_Data.csv",header=T)

data_sunflowerr<-data_all_raman[c(256444:279756),]

data_sunflowerr_subset<-data_sunflowerr[data_sunflowerr$land_use!=2,]

#convert to factors data_sunflowerr_subset$land_use <- as.factor(data_sunflowerr_subset$land_use) data_sunflowerr_subset$region_code1 <- as.factor(data_sunflowerr_subset$region_code1) data_sunflowerr_subset$region_code2 <- as.factor(data_sunflowerr_subset$region_code2) data_sunflowerr_subset$region_code3 <- as.factor(data_sunflowerr_subset$region_code3) data_sunflowerr_subset$resid<-as.factor(data_sunflowerr_subset$resid) data_sunflowerr_subset$PA<- as.factor(data_sunflowerr_subset$PA) colnames(data_sunflowerr_subset) <- c("landuse", "agri", "trans", "elevation",

"region_type","region_code1","region_code2","region_code3","rdist", "resid","PA","ID") head(data_sunflowerr_subset)

library(lme4)

##check collinearity

library(car)

model_vif <- glm(landuse ~ scale(agri)+scale(trans)+scale(elevation) +region_type+PA, family=binomial, data=data_sunflowerr_subset) summary(model_vif)

vif(model_vif)

###try to fix the random effects first

model_r1 <- glmer(landuse ~ scale(agri)+scale(trans)+scale(elevation) +region_type+PA+

(1|region_code1), family=binomial, data=data_sunflowerr_subset) summary(model_r1)

model_r2 <- glmer(landuse ~ scale(agri)+scale(trans) +scale(elevation)+region_type+PA+

(1|region_code2), family=binomial, data=data_sunflowerr_subset) summary(model_r2)

model_r3 <- glmer(landuse ~ scale(agri)+scale(trans)+scale(elevation) +region_type+PA+

(1|region_code3), family=binomial, data=data_sunflowerr_subset) summary(model_r3)

AIC(model_r1, model_r2, model_r3)

#choose region_code3 with smallest AIC

model_r4 <- glmer(landuse ~ scale(agri)+scale(trans)+scale(elevation) +region_type+PA+

(rdist|region_code3), family=binomial, data=data_sunflowerr_subset) summary(model_r4)

##choose model_r4 with samllest AIC

##############################################################################

###one variable### model_null<-glmer(landuse~1+(rdist|region_code3), family=binomial, data=data_sunflowerr_subset) summary(model_null)

model1<-glmer(landuse~scale(agri)+(rdist|region_code3), family=binomial, data=data_sunflowerr_subset) summary(model1) model2<-glmer(landuse~scale(trans)+(rdist|region_code3), family=binomial, data=data_sunflowerr_subset) summary(model2)

model3<-glmer(landuse~scale(elevation)+(rdist|region_code3), family=binomial, data=data_sunflowerr_subset) summary(model3)

model4<-glmer(landuse~region_type+(rdist|region_code3), family=binomial, data=data_sunflowerr_subset) summary(model4)

model5<-glmer(landuse~PA+(rdist|region_code3), family=binomial, data=data_sunflowerr_subset) summary(model5)

###two variables###

model6<-glmer(landuse~scale(agri)+scale(trans)+(rdist|region_code3), family=binomial, data=data_sunflowerr_subset) summary(model6)

model7<-glmer(landuse~scale(agri)+scale(elevation)+(rdist|region_code3), family=binomial, data=data_sunflowerr_subset) summary(model7)

model8<-glmer(landuse~scale(agri)+region_type+(rdist|region_code3), family=binomial, data=data_sunflowerr_subset) summary(model8) model9<-glmer(landuse~scale(trans)+region_type+(rdist|region_code3), family=binomial, data=data_sunflowerr_subset) summary(model9)

model10<-glmer(landuse~scale(elevation)+region_type+(rdist|region_code3), family=binomial, data=data_sunflowerr_subset) summary(model10)

model11<-glmer(landuse~scale(trans)+scale(elevation)+(rdist|region_code3), family=binomial, data=data_sunflowerr_subset) summary(model11)

model12<-glmer(landuse~scale(agri)+PA+(rdist|region_code3), family=binomial, data=data_sunflowerr_subset) summary(model12)

model13<-glmer(landuse~scale(trans)+PA+(rdist|region_code3), family=binomial, data=data_sunflowerr_subset) summary(model13)

model14<-glmer(landuse~scale(elevation)+PA+(rdist|region_code3), family=binomial, data=data_sunflowerr_subset) summary(model14)

model15<-glmer(landuse~region_type+PA+(rdist|region_code3), family=binomial, data=data_sunflowerr_subset) summary(model15)

###three variables###

model16<-glmer(landuse~scale(agri)+scale(trans)+region_type+(rdist|region_code3), family=binomial, data=data_sunflowerr_subset) summary(model16)

model17<-glmer(landuse~scale(agri)+scale(elevation)+region_type+(rdist|region_code3), family=binomial, data=data_sunflowerr_subset) summary(model17)

model18<-glmer(landuse~scale(agri)+scale(trans)+scale(elevation)+(rdist|region_code3), family=binomial, data=data_sunflowerr_subset) summary(model18)

model19<-glmer(landuse~scale(trans)+scale(elevation)+region_type+(rdist|region_code3), family=binomial, data=data_sunflowerr_subset) summary(model19)

model20<-glmer(landuse~scale(agri)+scale(trans)+PA+(rdist|region_code3), family=binomial, data=data_sunflowerr_subset) summary(model20)

model21<-glmer(landuse~scale(agri)+scale(elevation)+PA+(rdist|region_code3), family=binomial, data=data_sunflowerr_subset) summary(model21)

model22<-glmer(landuse~scale(agri)+region_type+PA+(rdist|region_code3), family=binomial, data=data_sunflowerr_subset) summary(model22)

model23<-glmer(landuse~scale(trans)+scale(elevation)+PA+(rdist|region_code3), summary(model23)

model24<-glmer(landuse~scale(trans)+region_type+PA+(rdist|region_code3), family=binomial, data=data_sunflowerr_subset) summary(model24)

model25<-glmer(landuse~scale(elevation)+region_type+PA+(rdist|region_code3), family=binomial, data=data_sunflowerr_subset) summary(model25)

###four variables###

model26<-

glmer(landuse~scale(agri)+scale(trans)+scale(elevation)+region_type+(rdist|region_code3), family=binomial, data=data_sunflowerr_subset) summary(model26)

model27<-glmer(landuse~scale(agri)+scale(trans)+scale(elevation)+PA+(rdist|region_code3), family=binomial, data=data_sunflowerr_subset) summary(model27)

model28<-glmer(landuse~scale(agri)+scale(trans)+PA+region_type+(rdist|region_code3), family=binomial, data=data_sunflowerr_subset) summary(model28)

model29<-glmer(landuse~scale(agri)+PA+scale(elevation)+region_type+(rdist|region_code3), family=binomial, data=data_sunflowerr_subset) summary(model29)

model30<-glmer(landuse~PA+scale(trans)+scale(elevation)+region_type+(rdist|region_code3), summary(model30)

###five variables###

model31<-glmer(landuse ~ scale(agri)+scale(trans)+scale(elevation)

+region_type+PA+(rdist|region_code3), family=binomial, data=data_sunflowerr_subset)

###one interaction###

model32<-

glmer(landuse~scale(agri)+scale(trans)+scale(elevation)+region_type+scale(agri):region_type+(r dist|region_code3), family=binomial, data=data_sunflowerr_subset) summary(model32)

model33<-

glmer(landuse~scale(agri)+scale(trans)+scale(elevation)+region_type+scale(trans):region_type+

(rdist|region_code3), family=binomial, data=data_sunflowerr_subset) summary(model33)

model34<-

glmer(landuse~scale(agri)+scale(trans)+scale(elevation)+region_type+scale(elevation):region_ty pe+(rdist|region_code3), family=binomial, data=data_sunflowerr_subset) summary(model34)

model35<-

glmer(landuse~scale(agri)+scale(trans)+scale(elevation)+PA+scale(agri):PA+(rdist|region_code

3), family=binomial, data=data_sunflowerr_subset) summary(model35)

model36<-

glmer(landuse~scale(agri)+scale(trans)+region_type+PA+scale(agri):PA+(rdist|region_code3),

summary(model36)

model37<-

glmer(landuse~scale(agri)+scale(elevation)+region_type+PA+scale(agri):PA+(rdist|region_code

3), family=binomial, data=data_sunflowerr_subset) summary(model37)

model38<-

glmer(landuse~scale(agri)+scale(trans)+scale(elevation)+PA+scale(trans):PA+(rdist|region_code

3), family=binomial, data=data_sunflowerr_subset) summary(model38)

model39<-

glmer(landuse~scale(agri)+scale(trans)+region_type+PA+scale(trans):PA+(rdist|region_code3), family=binomial, data=data_sunflowerr_subset) summary(model39)

model40<-

glmer(landuse~scale(trans)+scale(elevation)+region_type+PA+scale(trans):PA+(rdist|region_co de3), family=binomial, data=data_sunflowerr_subset) summary(model40)

model41<-

glmer(landuse~scale(agri)+scale(trans)+scale(elevation)+PA+scale(elevation):PA+(rdist|region_ code3), family=binomial, data=data_sunflowerr_subset) summary(model41)

model42<-

glmer(landuse~scale(agri)+scale(elevation)+region_type+PA+scale(elevation):PA+(rdist|region_ code3), family=binomial, data=data_sunflowerr_subset)

summary(model42)

model43<-

glmer(landuse~scale(trans)+scale(elevation)+region_type+PA+scale(elevation):PA+(rdist|region

_code3), family=binomial, data=data_sunflowerr_subset) summary(model43)

model44<-

glmer(landuse~scale(agri)+scale(trans)+region_type+PA+region_type:PA+(rdist|region_code3), family=binomial, data=data_sunflowerr_subset) summary(model44)

model45<-

glmer(landuse~scale(agri)+scale(elevation)+region_type+PA+region_type:PA+(rdist|region_cod e3), family=binomial, data=data_sunflowerr_subset) summary(model45)

model46<-

glmer(landuse~scale(trans)+scale(elevation)+region_type+PA+region_type:PA+(rdist|region_co de3), family=binomial, data=data_sunflowerr_subset) summary(model46)

###two interactions###

## with region

model47<-

glmer(landuse~scale(agri)+scale(trans)+scale(elevation)+region_type+scale(agri):region_type+s cale(trans):region_type+(rdist|region_code3), family=binomial, data=data_sunflowerr_subset) summary(model47)

model48<-

glmer(landuse~scale(agri)+scale(trans)+scale(elevation)+region_type+scale(agri):region_type+s cale(elevation):region_type+(rdist|region_code3), family=binomial, data=data_sunflowerr_subset) summary(model48)

model49<-

glmer(landuse~scale(agri)+scale(trans)+scale(elevation)+region_type+scale(trans):region_type+ scale(elevation):region_type+(rdist|region_code3), family=binomial, data=data_sunflowerr_subset) summary(model49)

##with PA model50<-

glmer(landuse~scale(agri)+scale(trans)+scale(elevation)+PA+scale(agri):PA+scale(trans):PA+(r dist|region_code3), family=binomial, data=data_sunflowerr_subset) summary(model50)

model51<-

glmer(landuse~scale(agri)+scale(trans)+region_type+PA+scale(agri):PA+scale(trans):PA+(rdist| region_code3), family=binomial, data=data_sunflowerr_subset) summary(model51)

model52<-

glmer(landuse~scale(agri)+scale(trans)+scale(elevation)+PA+scale(agri):PA+scale(elevation):P

A+(rdist|region_code3), family=binomial, data=data_sunflowerr_subset) summary(model52)

model53<-

glmer(landuse~scale(agri)+scale(elevation)+region_type+PA+scale(agri):PA+scale(elevation):P A+(rdist|region_code3), family=binomial, data=data_sunflowerr_subset) summary(model53)

model54<-

glmer(landuse~scale(agri)+scale(trans)+region_type+PA+scale(agri):PA+region_type:PA+(rdist| region_code3), family=binomial, data=data_sunflowerr_subset) summary(model54)

model55<-

glmer(landuse~scale(agri)+scale(elevation)+region_type+PA+scale(agri):PA+region_type:PA+( rdist|region_code3), family=binomial, data=data_sunflowerr_subset) summary(model55)

model56<-

glmer(landuse~scale(agri)+scale(trans)+scale(elevation)+PA+scale(trans):PA+scale(elevation):P

A+(rdist|region_code3), family=binomial, data=data_sunflowerr_subset) summary(model56)

model57<glmer(landuse~scale(trans)+scale(elevation)+region_type+PA+scale(trans):PA+scale(elevation): PA+(rdist|region_code3), family=binomial, data=data_sunflowerr_subset) summary(model57)

model58<-

glmer(landuse~scale(agri)+scale(trans)+region_type+PA+scale(trans):PA+region_type:PA+(rdis t|region_code3), family=binomial, data=data_sunflowerr_subset) summary(model58)

model59<-

glmer(landuse~scale(trans)+scale(elevation)+region_type+PA+scale(trans):PA+region_type:PA

+(rdist|region_code3), family=binomial, data=data_sunflowerr_subset) summary(model59)

##combine two sets

model60<-

glmer(landuse~scale(agri)+scale(trans)+region_type+PA+scale(agri):region_type+scale(agri):P

A+(rdist|region_code3), family=binomial, data=data_sunflowerr_subset) summary(model60)

model61<-

glmer(landuse~scale(agri)+scale(elevation)+region_type+PA+scale(agri):region_type+scale(agri

):PA+(rdist|region_code3), family=binomial, data=data_sunflowerr_subset) summary(model61)

model62<-

glmer(landuse~scale(agri)+scale(trans)+region_type+PA+scale(agri):region_type+scale(trans):P

A+(rdist|region_code3), family=binomial, data=data_sunflowerr_subset) summary(model62)

model63<-

glmer(landuse~scale(agri)+scale(elevation)+region_type+PA+scale(agri):region_type+scale(elev ation):PA+(rdist|region_code3), family=binomial, data=data_sunflowerr_subset) summary(model63)

model64<-

glmer(landuse~scale(agri)+scale(trans)+region_type+PA+scale(agri):region_type+region_type:P

A+(rdist|region_code3), family=binomial, data=data_sunflowerr_subset) summary(model64)

model65<-

glmer(landuse~scale(agri)+scale(elevation)+region_type+PA+scale(agri):region_type+region_ty pe:PA+(rdist|region_code3), family=binomial, data=data_sunflowerr_subset) summary(model65)

model66<-

glmer(landuse~scale(agri)+scale(trans)+region_type+PA+scale(trans):region_type+scale(agri):P

A+(rdist|region_code3), family=binomial, data=data_sunflowerr_subset)

summary(model66)

model67<glmer(landuse~scale(agri)+scale(trans)+region_type+PA+scale(trans):region_type+scale(trans): PA+(rdist|region_code3), family=binomial, data=data_sunflowerr_subset) summary(model67)

model68<-

glmer(landuse~scale(elevation)+scale(trans)+region_type+PA+scale(trans):region_type+scale(tr ans):PA+(rdist|region_code3), family=binomial, data=data_sunflowerr_subset) summary(model68)

model69<-

glmer(landuse~scale(trans)+scale(elevation)+region_type+PA+scale(trans):region_type+scale(el evation):PA+(rdist|region_code3), family=binomial, data=data_sunflowerr_subset) summary(model69)

model70<glmer(landuse~scale(agri)+scale(trans)+region_type+PA+scale(trans):region_type+region_type: PA+(rdist|region_code3), family=binomial, data=data_sunflowerr_subset) summary(model70)

model71<-

glmer(landuse~scale(trans)+scale(elevation)+region_type+PA+scale(trans):region_type+region_ type:PA+(rdist|region_code3), family=binomial, data=data_sunflowerr_subset) summary(model71)

model72<-

glmer(landuse~scale(agri)+scale(elevation)+region_type+PA+scale(elevation):region_type+scal e(agri):PA+(rdist|region_code3), family=binomial, data=data_sunflowerr_subset) summary(model72)

model73<-

glmer(landuse~scale(trans)+scale(elevation)+region_type+PA+scale(elevation):region_type+sca le(trans):PA+(rdist|region_code3), family=binomial, data=data_sunflowerr_subset) summary(model73)

model74<-

glmer(landuse~scale(agri)+scale(elevation)+region_type+PA+scale(elevation):region_type+scal e(elevation):PA+(rdist|region_code3), family=binomial, data=data_sunflowerr_subset) summary(model74)

model75<-

glmer(landuse~scale(trans)+scale(elevation)+region_type+PA+scale(elevation):region_type+sca le(elevation):PA+(rdist|region_code3), family=binomial, data=data_sunflowerr_subset) summary(model75)

model76<-

glmer(landuse~scale(agri)+scale(elevation)+region_type+PA+scale(elevation):region_type+regi on_type:PA+(rdist|region_code3), family=binomial, data=data_sunflowerr_subset) summary(model76)

model77<-

glmer(landuse~scale(trans)+scale(elevation)+region_type+PA+scale(elevation):region_type+reg ion_type:PA+(rdist|region_code3), family=binomial, data=data_sunflowerr_subset) summary(model77)

###three interactions###

model78<-

glmer(landuse~scale(agri)+scale(trans)+scale(elevation)+region_type+scale(agri):region_type+s cale(elevation):region_type+scale(trans):region_type+(rdist|region_code3), family=binomial, data=data_sunflowerr_subset) summary(model78)

model79<-

glmer(landuse~scale(agri)+scale(trans)+region_type+PA+scale(agri):region_type+scale(agri):P

A+scale(trans):region_type+(rdist|region_code3), family=binomial, data=data_sunflowerr_subset) summary(model79)

model80<-

glmer(landuse~scale(agri)+scale(trans)+region_type+PA+scale(agri):region_type+scale(trans):P

A+scale(trans):region_type+(rdist|region_code3), family=binomial, data=data_sunflowerr_subset) summary(model80)

model81<-

glmer(landuse~scale(agri)+scale(trans)+region_type+PA+scale(agri):region_type+region_type:P

A+scale(trans):region_type+(rdist|region_code3), family=binomial, data=data_sunflowerr_subset) summary(model81)

model82<-

glmer(landuse~scale(agri)+scale(elevation)+region_type+PA+scale(agri):region_type+scale(agri

):PA+scale(elevation):region_type+(rdist|region_code3), family=binomial, data=data_sunflowerr_subset) summary(model82)

model83<-

glmer(landuse~scale(agri)+scale(elevation)+region_type+PA+scale(agri):region_type+scale(elev ation):PA+scale(elevation):region_type+(rdist|region_code3), family=binomial, data=data_sunflowerr_subset) summary(model83)

model84<-

glmer(landuse~scale(agri)+scale(elevation)+region_type+PA+scale(agri):region_type+region_ty pe:PA+scale(elevation):region_type+(rdist|region_code3), family=binomial, data=data_sunflowerr_subset)

summary(model84)

model85<-

glmer(landuse~scale(trans)+scale(elevation)+region_type+PA+scale(trans):region_type+scale(tr ans):PA+scale(elevation):region_type+(rdist|region_code3), family=binomial, data=data_sunflowerr_subset) summary(model85)

model86<-

glmer(landuse~scale(trans)+scale(elevation)+region_type+PA+scale(trans):region_type+scale(el evation):PA+scale(elevation):region_type+(rdist|region_code3), family=binomial, data=data_sunflowerr_subset) summary(model86)

model87<-

glmer(landuse~scale(trans)+scale(elevation)+region_type+PA+scale(trans):region_type+region_ type:PA+scale(elevation):region_type+(rdist|region_code3), family=binomial, data=data_sunflowerr_subset) summary(model87)

###four interactions### model88<-

glmer(landuse~scale(agri)+scale(trans)+region_type+PA+scale(agri):region_type+scale(trans):re gion_type+scale(agri):PA+scale(trans):PA+(rdist|region_code3), family=binomial, data=data_sunflowerr_subset) summary(model88)

model89<-

glmer(landuse~scale(agri)+scale(trans)+region_type+PA+scale(agri):region_type+scale(trans):re gion_type+scale(agri):PA+region_type:PA+(rdist|region_code3), family=binomial, data=data_sunflowerr_subset) summary(model89)

model90<-

glmer(landuse~scale(agri)+scale(trans)+region_type+PA+scale(agri):region_type+scale(trans):re gion_type+scale(trans):PA+region_type:PA+(rdist|region_code3), family=binomial, data=data_sunflowerr_subset) summary(model90)

model91<-

glmer(landuse~scale(agri)+scale(elevation)+region_type+PA+scale(agri):region_type+scale(elev ation):region_type+scale(agri):PA+scale(elevation):PA+(rdist|region_code3), family=binomial, data=data_sunflowerr_subset) summary(model91)

model92<-

glmer(landuse~scale(agri)+scale(elevation)+region_type+PA+scale(agri):region_type+scale(elev ation):region_type+scale(agri):PA+region_type:PA+(rdist|region_code3), family=binomial, data=data_sunflowerr_subset) summary(model92)

model93<-

glmer(landuse~scale(agri)+scale(elevation)+region_type+PA+scale(agri):region_type+scale(elev ation):region_type+region_type:PA+scale(elevation):PA+(rdist|region_code3), family=binomial, data=data_sunflowerr_subset) summary(model93)

model94<-

glmer(landuse~scale(trans)+scale(elevation)+region_type+PA+scale(trans):region_type+scale(el evation):region_type+scale(trans):PA+scale(elevation):PA+(rdist|region_code3), family=binomial, data=data_sunflowerr_subset) summary(model94)

model95<-

glmer(landuse~scale(trans)+scale(elevation)+region_type+PA+scale(trans):region_type+scale(el evation):region_type+scale(trans):PA+region_type:PA+(rdist|region_code3), family=binomial, data=data_sunflowerr_subset)

summary(model95)

model96<-

glmer(landuse~scale(trans)+scale(elevation)+region_type+PA+scale(trans):region_type+scale(el evation):region_type+region_type:PA+scale(elevation):PA+(rdist|region_code3), family=binomial, data=data_sunflowerr_subset) summary(model96)

###try five interactions### model97<-

glmer(landuse~scale(agri)+scale(trans)+region_type+PA+scale(agri):region_type+scale(trans):re gion_type+scale(agri):PA+scale(trans):PA+region_type:PA+(rdist|region_code3), family=binomial, data=data_sunflowerr_subset) summary(model97)

model98<-

glmer(landuse~scale(agri)+scale(elevation)+region_type+PA+scale(agri):region_type+scale(elev ation):region_type+scale(agri):PA+scale(elevation):PA+region_type:PA+(rdist|region_code3), family=binomial, data=data_sunflowerr_subset) summary(model98)

model99<-

glmer(landuse~scale(trans)+scale(elevation)+region_type+PA+scale(trans):region_type+scale(el evation):region_type+scale(trans):PA+scale(elevation):PA+region_type:PA+(rdist|region_code3

), family=binomial, data=data_sunflowerr_subset) summary(model99)

##select the best model

library(MuMIn)

models_sel_sunflowerr<-

model.sel(model_null,model1,model2,model3,model4,model5,model6,model7, model8, model9, model10, model11,model12, model13, model14,

model15,model16,model17,model18,model19,model20,model22,model23,model24,model28,mo del30,model35,rank="AIC")

sink(file="models_sel_sunflowerr.txt")

models_sel_sunflowerr

sink()

##dignostic plots library(arm) binnedplot(predict(model35, type="response"), resid(model35, type="response"))

library(effects) plot(allEffects(model35)) allEffects(model35)

sink(file="alleffects(model35).txt") allEffects(model35) sink()

sink("summary(model35).txt") summary(model35)

sink()

####################prediction with actual yield ###############################

#1. Model baseline scenario, just put the actual yield in pred_sunflowerr <- predict(model35, data_sunflowerr_subset, re.form=NULL, type="response") head(pred_sunflowerr)

write.csv(pred_sunflowerr, "pred_sunflowerr.csv")

##########################prediction with potential yield ######################

##get potential yield data data_all<-read.csv("Appendix_S4_Supplementary_Data.csv",header=T)

data_sunflower<-data_all[c(256444:279256),]

data_sunflower_subset<-data_sunflower[data_sunflower$land_use!=2,]

data_sunflower_subset$land_use <- as.factor(data_sunflower_subset$land_use) data_sunflower_subset$region_code1 <- as.factor(data_sunflower_subset$region_code1) data_sunflower_subset$region_code2 <- as.factor(data_sunflower_subset$region_code2) data_sunflower_subset$region_code3 <- as.factor(data_sunflower_subset$region_code3) data_sunflower_subset$resid<-as.factor(data_sunflower_subset$resid) data_sunflower_subset$PA<- as.factor(data_sunflower_subset$PA)

colnames(data_sunflower_subset) <- c("landuse", "agri", "trans", "elevation",

"region_type","region_code1","region_code2","region_code3","rdist", "resid","PA","ID") head(data_sunflower_subset)

#2. Scenario: actual yield being replaced by potential yield data pred_sunflower <- predict(model35, data_sunflower_subset, re.form=NULL, type="response") head(pred_sunflower)

write.csv(pred_sunflower, "pred_sunflower.csv")

#3. Scenario A: agr increase 50%

sunflower_y <- cbind(data_sunflower_subset$landuse, (data_sunflower_subset$agri)*1.5 , data_sunflower_subset[,3:12])

colnames(sunflower_y) <- c("landuse", "agri", "trans","elevation","region_type","region_code1","region_code2","region_code3","rdist","resid","PA","ID")

summary(sunflower_y)

pred_sunflower<-predict(model35, sunflower_y, re.form=NULL, type="response"))

write.csv(pred_sunflower, "pred_sunflower_agri.csv")

#4. Scenario B: transport costs increase by 35%

sunflower_trans_y <- cbind(data_sunflower_subset[,1:2], data_sunflower_subset[,3]*1.35, data_sunflower_subset[,4:12]) colnames(sunflower_trans_y) <- c("landuse", "agri",

"trans","elevation","region_type","region_code1","region_code2","region_code3","rdist","resid",

"PA","ID") summary(sunflower_trans_y)

pred_trans_sunflower<-predict(model35, sunflower_trans_y, re.form=NULL, type="response")

write.csv(pred_trans_sunflower, "pred_sunflower_trans.csv")

#5. Scenario C: political stability has total conversion

region_sunflower <- data_sunflower_subset

summary(region_sunflower) region_sunflower[,5] <- "Division"

region_sunflower[,5] <- as.factor(region_sunflower[,5])

pred_region_sunflower <- predict(model35, region_sunflower, re.form=NULL, type="response") head(pred_region_sunflower)

write.csv(pred_region_sunflower, "pred_sunflower_stability.csv")

#6. Scenario D: worst case. agri increase 50%, and stability has total conversion sunflower_worstcase<-sunflower_y

sunflower_worstcase[,5]<-"Division"

pred_worstcase_sunflower<-predict(model35, sunflower_worstcase, re.form=NULL, type="response")

write.csv(pred_worstcase_sunflower, "pred_sunflower_worstcase.csv")

rm(list=ls())

##############################################################################

##################model land use with characteristics of oilpalm#######################

setwd("D:\\Myanmar project\\Resubmission 2\\Code and data (submit)")

data_all_raman<-read.csv("Appendix_S3_Supplementary_Data.csv",header=T)

data_oilpalmr<-data_all_raman[c(279757:303069),]

data_oilpalmr_subset<-data_oilpalmr[data_oilpalmr$land_use!=2,]

#convert to factors data_oilpalmr_subset$land_use <- as.factor(data_oilpalmr_subset$land_use) data_oilpalmr_subset$region_code1 <- as.factor(data_oilpalmr_subset$region_code1) data_oilpalmr_subset$region_code2 <- as.factor(data_oilpalmr_subset$region_code2) data_oilpalmr_subset$region_code3 <- as.factor(data_oilpalmr_subset$region_code3) data_oilpalmr_subset$resid<-as.factor(data_oilpalmr_subset$resid) data_oilpalmr_subset$PA<- as.factor(data_oilpalmr_subset$PA)

colnames(data_oilpalmr_subset) <- c("landuse", "agri", "trans", "elevation",

"region_type","region_code1","region_code2","region_code3","rdist", "resid","PA","ID") head(data_oilpalmr_subset)

library(lme4)

##check collinearity

library(car)

model_vif <- glm(landuse ~ scale(agri)+scale(trans)+scale(elevation) +region_type+PA, family=binomial, data=data_oilpalmr_subset) summary(model_vif)

vif(model_vif)

##remove trans because of collinearity##

###try to fix the random effects first

model_r1 <- glmer(landuse ~ scale(agri)+scale(elevation) +region_type+PA+

(1|region_code1), family=binomial, data=data_oilpalmr_subset) summary(model_r1)

model_r2 <- glmer(landuse ~ scale(agri)+scale(elevation)+region_type+PA+ (1|region_code2), family=binomial, data=data_oilpalmr_subset) summary(model_r2)

model_r3 <- glmer(landuse ~ scale(agri)+scale(elevation) +region_type+PA+

(1|region_code3), family=binomial, data=data_oilpalmr_subset) summary(model_r3)

model_r4 <- glmer(landuse ~ scale(agri)+scale(elevation) +region_type+PA+ (rdist|region_code3), family=binomial, data=data_oilpalmr_subset) summary(model_r4)

AIC(model_r1,model_r2,model_r3,model_r4)

##choose model_r4 with samllest AIC

###one variable### model_null<-glmer(landuse~1+(rdist|region_code3), family=binomial, data=data_oilpalmr_subset) summary(model_null)

model1<-glmer(landuse~scale(agri)+(rdist|region_code3), family=binomial, data=data_oilpalmr_subset) summary(model1) model3<-glmer(landuse~scale(elevation)+(rdist|region_code3), family=binomial, data=data_oilpalmr_subset) summary(model3)

model4<-glmer(landuse~region_type+(rdist|region_code3), family=binomial, data=data_oilpalmr_subset) summary(model4)

model5<-glmer(landuse~PA+(rdist|region_code3), family=binomial, data=data_oilpalmr_subset) summary(model5)

###two variables###

model7<-glmer(landuse~scale(agri)+scale(elevation)+(rdist|region_code3), family=binomial, data=data_oilpalmr_subset) summary(model7)

model8<-glmer(landuse~scale(agri)+region_type+(rdist|region_code3), family=binomial, data=data_oilpalmr_subset) summary(model8)

model10<-glmer(landuse~scale(elevation)+region_type+(rdist|region_code3), family=binomial, data=data_oilpalmr_subset) summary(model10)

model12<-glmer(landuse~scale(agri)+PA+(rdist|region_code3), family=binomial, data=data_oilpalmr_subset)

summary(model12)

model14<-glmer(landuse~scale(elevation)+PA+(rdist|region_code3), family=binomial, data=data_oilpalmr_subset) summary(model14)

model15<-glmer(landuse~region_type+PA+(rdist|region_code3), family=binomial, data=data_oilpalmr_subset) summary(model15)

###three variables###

model17<-glmer(landuse~scale(agri)+scale(elevation)+region_type+(rdist|region_code3), family=binomial, data=data_oilpalmr_subset) summary(model17)

model21<-glmer(landuse~scale(agri)+scale(elevation)+PA+(rdist|region_code3), family=binomial, data=data_oilpalmr_subset) summary(model21)

model22<-glmer(landuse~scale(agri)+region_type+PA+(rdist|region_code3), family=binomial, data=data_oilpalmr_subset) summary(model22) model25<-glmer(landuse~scale(elevation)+region_type+PA+(rdist|region_code3), family=binomial, data=data_oilpalmr_subset) summary(model25)

###four variables###

model29<-glmer(landuse~scale(agri)+PA+scale(elevation)+region_type+(rdist|region_code3), family=binomial, data=data_oilpalmr_subset) summary(model29)

###one interaction###

model32<-

glmer(landuse~scale(agri)+scale(trans)+scale(elevation)+region_type+scale(agri):region_type+(r dist|region_code3), family=binomial, data=data_oilpalmr_subset) summary(model32)

model34<-

glmer(landuse~scale(agri)+scale(trans)+scale(elevation)+region_type+scale(elevation):region_ty pe+(rdist|region_code3), family=binomial, data=data_oilpalmr_subset) summary(model34)

model37<-

glmer(landuse~scale(agri)+scale(elevation)+region_type+PA+scale(agri):PA+(rdist|region_code

3), family=binomial, data=data_oilpalmr_subset) summary(model37)

model42<-

glmer(landuse~scale(agri)+scale(elevation)+region_type+PA+scale(elevation):PA+(rdist|region_ code3), family=binomial, data=data_oilpalmr_subset) summary(model42)

model45<-

glmer(landuse~scale(agri)+scale(elevation)+region_type+PA+region_type:PA+(rdist|region_cod e3), family=binomial, data=data_oilpalmr_subset) summary(model45)

###two interactions###

## with region

##with PA

model53<-

glmer(landuse~scale(agri)+scale(elevation)+region_type+PA+scale(agri):PA+scale(elevation):P

A+(rdist|region_code3), family=binomial, data=data_oilpalmr_subset) summary(model53)

model55<-

glmer(landuse~scale(agri)+scale(elevation)+region_type+PA+scale(agri):PA+region_type:PA+( rdist|region_code3), family=binomial, data=data_oilpalmr_subset) summary(model55)

##combine two sets

model61<-

glmer(landuse~scale(agri)+scale(elevation)+region_type+PA+scale(agri):region_type+scale(agri

):PA+(rdist|region_code3), family=binomial, data=data_oilpalmr_subset) summary(model61)

model63<-

glmer(landuse~scale(agri)+scale(elevation)+region_type+PA+scale(agri):region_type+scale(elev ation):PA+(rdist|region_code3), family=binomial, data=data_oilpalmr_subset) summary(model63)

model65<-

glmer(landuse~scale(agri)+scale(elevation)+region_type+PA+scale(agri):region_type+region_ty pe:PA+(rdist|region_code3), family=binomial, data=data_oilpalmr_subset) summary(model65)

model72<-

glmer(landuse~scale(agri)+scale(elevation)+region_type+PA+scale(elevation):region_type+scal e(agri):PA+(rdist|region_code3), family=binomial, data=data_oilpalmr_subset) summary(model72)

model74<-

glmer(landuse~scale(agri)+scale(elevation)+region_type+PA+scale(elevation):region_type+scal e(elevation):PA+(rdist|region_code3), family=binomial, data=data_oilpalmr_subset) summary(model74)

model76<-

glmer(landuse~scale(agri)+scale(elevation)+region_type+PA+scale(elevation):region_type+regi on_type:PA+(rdist|region_code3), family=binomial, data=data_oilpalmr_subset) summary(model76)

###three interactions###

model82<-

glmer(landuse~scale(agri)+scale(elevation)+region_type+PA+scale(agri):region_type+scale(agri

):PA+scale(elevation):region_type+(rdist|region_code3), family=binomial, data=data_oilpalmr_subset) summary(model82)

model83<-

glmer(landuse~scale(agri)+scale(elevation)+region_type+PA+scale(agri):region_type+scale(elev ation):PA+scale(elevation):region_type+(rdist|region_code3), family=binomial, data=data_oilpalmr_subset) summary(model83)

model84<-

glmer(landuse~scale(agri)+scale(elevation)+region_type+PA+scale(agri):region_type+region_ty pe:PA+scale(elevation):region_type+(rdist|region_code3), family=binomial, data=data_oilpalmr_subset) summary(model84)

###four interactions###

model91<-

glmer(landuse~scale(agri)+scale(elevation)+region_type+PA+scale(agri):region_type+scale(elev ation):region_type+scale(agri):PA+scale(elevation):PA+(rdist|region_code3), family=binomial, data=data_oilpalmr_subset) summary(model91)

model92<-

glmer(landuse~scale(agri)+scale(elevation)+region_type+PA+scale(agri):region_type+scale(elev ation):region_type+scale(agri):PA+region_type:PA+(rdist|region_code3), family=binomial, data=data_oilpalmr_subset) summary(model92)

model93<-

glmer(landuse~scale(agri)+scale(elevation)+region_type+PA+scale(agri):region_type+scale(elev ation):region_type+region_type:PA+scale(elevation):PA+(rdist|region_code3), family=binomial, data=data_oilpalmr_subset) summary(model93)

###try five interactions###

model98<-

glmer(landuse~scale(agri)+scale(elevation)+region_type+PA+scale(agri):region_type+scale(elev ation):region_type+scale(agri):PA+scale(elevation):PA+region_type:PA+(rdist|region_code3), family=binomial, data=data_oilpalmr_subset) summary(model98)

##select the best model library(MuMIn)

models_sel_oilpalmr<- model.sel(model_null,model1,model3,model4,model5,model7, model8, model10, model12, model14, model15,model17,model21,model22,model29, model42, rank="AIC") sink(file="models_sel_oilpalmr.txt")

models_sel_oilpalmr

sink()

##dignostic plots

library(arm) binnedplot(predict(model42, type="response"), resid(model42, type="response"))

library(effects) plot(allEffects(model42)) allEffects(model42)

sink(file="alleffects(model42).txt") allEffects(model42) sink()

sink("summary(model42).txt") summary(model42)

sink()

####################prediction with actual yield ###############################

#1. Model baseline scenario, just put the actual yield in pred_oilpalmr <- predict(model42, data_oilpalmr_subset, re.form=NULL, type="response") head(pred_oilpalmr)

write.csv(pred_oilpalmr, "pred_oilpalmr.csv")

##########################prediction with potential yield ######################

##get potential yield data data_all<-read.csv("Appendix_S4_Supplementary_Data.csv",header=T) data_oilpalm<-data_all[c(279757:303069),]

data_oilpalm_subset<-data_oilpalm[data_oilpalm$land_use!=2,]

data_oilpalm_subset$land_use <- as.factor(data_oilpalm_subset$land_use) data_oilpalm_subset$region_code1 <- as.factor(data_oilpalm_subset$region_code1) data_oilpalm_subset$region_code2 <- as.factor(data_oilpalm_subset$region_code2) data_oilpalm_subset$region_code3 <- as.factor(data_oilpalm_subset$region_code3) data_oilpalm_subset$resid<-as.factor(data_oilpalm_subset$resid) data_oilpalm_subset$PA<- as.factor(data_oilpalm_subset$PA)

colnames(data_oilpalm_subset) <- c("landuse", "agri", "trans", "elevation",

"region_type","region_code1","region_code2","region_code3","rdist", "resid","PA","ID") head(data_oilpalm_subset)

#2. Scenario: actual yield being replaced by potential yield data pred_oilpalm <- predict(model42, data_oilpalm_subset, re.form=NULL, type="response") head(pred_oilpalm)

write.csv(pred_oilpalm, "pred_oilpalm.csv")

#3. Scenario A: agr increase 50%

oilpalm_y <- cbind(data_oilpalm_subset$landuse, (data_oilpalm_subset$agri)*1.5 , data_oilpalm_subset[,3:12])

colnames(oilpalm_y) <- c("landuse", "agri", "trans","elevation","region_type","region_code1","region_code2","region_code3","rdist","resid","PA","ID")

summary(oilpalm_y)

pred_oilpalm<-predict(model42, oilpalm_y, re.form=NULL, type="response"))

write.csv(pred_oilpalm, "pred_oilpalm_agri.csv")

#4. Scenario B: transport costs increase by 35%

oilpalm_trans_y <- cbind(data_oilpalm_subset[,1:2], data_oilpalm_subset[,3]*1.35, data_oilpalm_subset[,4:12]) colnames(oilpalm_trans_y) <- c("landuse", "agri",

"trans","elevation","region_type","region_code1","region_code2","region_code3","rdist","resid",

"PA","ID") summary(oilpalm_trans_y)

pred_trans_oilpalm<-predict(model42, oilpalm_trans_y, re.form=NULL, type="response")

write.csv(pred_trans_oilpalm, "pred_oilpalm_trans.csv")

#5. Scenario C: political stability has total conversion

region_oilpalm <- data_oilpalm_subset

summary(region_oilpalm) region_oilpalm[,5] <- "Division"

region_oilpalm[,5] <- as.factor(region_oilpalm[,5])

pred_region_oilpalm <- predict(model42, region_oilpalm, re.form=NULL, type="response") head(pred_region_oilpalm)

write.csv(pred_region_oilpalm, "pred_oilpalm_stability.csv")

#6. Scenario D: worst case. agri increase 50%, and stability has total conversion oilpalm_worstcase<-oilpalm_y

oilpalm_worstcase[,5]<-"Division"

pred_worstcase_oilpalm<-predict(model42, oilpalm_worstcase, re.form=NULL, type="response")

write.csv(pred_worstcase_oilpalm, "pred_oilpalm_worstcase.csv")

rm(list=ls())
